# Factors influencing choice of health system access level in China: A systematic review

**DOI:** 10.1371/journal.pone.0201887

**Published:** 2018-08-10

**Authors:** Yun Liu, Qingxia Kong, Shasha Yuan, Joris van de Klundert

**Affiliations:** 1 Erasmus School of Health Policy & Management, Erasmus University Rotterdam, Rotterdam, The Netherlands; 2 Rotterdam School of Management, Erasmus University Rotterdam, Rotterdam, The Netherlands; 3 Institute of Medical Information and Library, Chinese Academy of Medical Sciences & Peking Union Medical College, Beijing, China; 4 Prince Mohammad Bin Salman College of Business & Entrepreneurship, King Abdullah Economic City, Kingdom of Saudi Arabia; National Institute of Health, ITALY

## Abstract

**Objective:**

In China, patients increasingly choose to access already severely overcrowded higher level hospitals, leaving lower level facilities with low utilization rates. This situation undermines the effectiveness and efficiency of the health system. The situation tends to worsen despite policy measures aimed at improvement. We systematically review the factors affecting patient choice to synthesize scientific understanding of health system access in China. The review provides an evidence base for measures to direct patient flow towards lower level facilities.

**Methods:**

We screened the peer-reviewed literature published from April 2009 to January 2016 that investigates Chinese patients’ choice of health care facilities at different levels and assessed 45 studies in total. We applied two structured forms to extract data on each study’s characteristics, methodology, and factors.

**Results of data synthesis:**

The results identified four factor types: 1) patient, 2) provider, 3) context and 4) composite: combined patient, provider, and/or context attributes. Patient factors are mentioned the most, but the evidence on patient factors is often inconclusive. Evidence suggests that the provider factors ‘drug variety’ and ‘equipment’, and composite factor ‘perceived quality’, push patients from lower levels towards higher levels.

**Conclusion:**

Underuse of primary care facilities and overcrowding of higher level facilities will likely be amplified by current demographic trends. Evidence suggests that improving drug availability, equipment and perceived quality of primary care services can improve the situation. Well-designed research that considers the interactions between factors is called for to better inform future interventions.

## Introduction

Since the turn of the millennium, the Chinese government has made unprecedented investments to improve its health system. Government spending on health care has grown tenfold to a total budget of 1,243 billion RMB in 2016 [1]. By November 2016, the number of hospitals was increased to 29,000 and the number of primary care facilities amounted to 930,000 [2]. Supply-side growth, however, continues to be outpaced by the growth in demand, particularly for higher level hospitals [3]. The resulting overcrowding in higher level hospitals and low utilization of primary care facilities undermine the effectiveness and efficiency of the health system [4–7]. Here we review the scientific evidence for factors that influence the patient’s choice of health care access level, as a step toward developing evidence-based interventions to improve patient flow.

**Fig 1:**
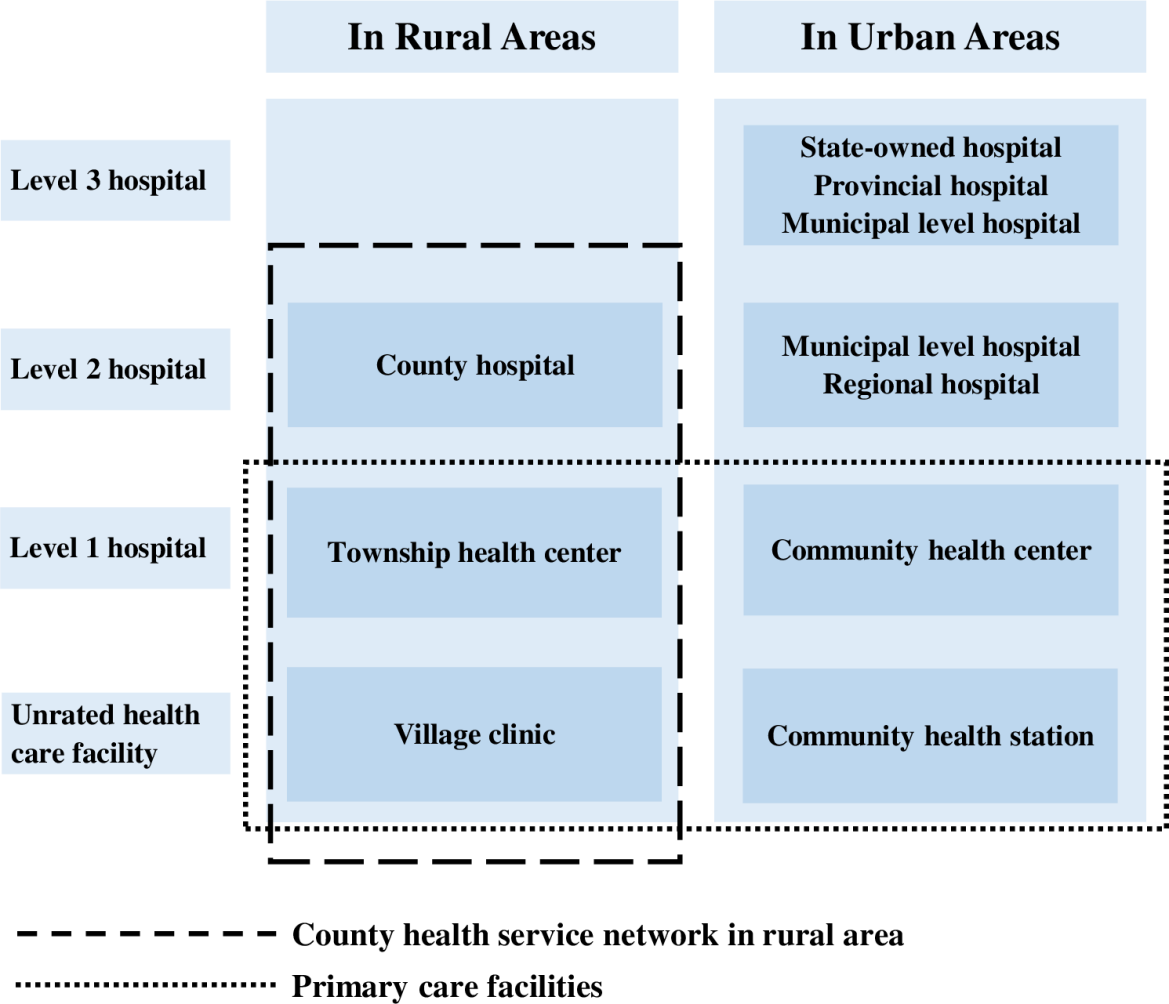
The three-level hospital system plus primary care facilities in China.

The Chinese health system defines hospitals as “medical institutions having more than 20 beds” and distinguished the hospital system in “3 levels and 10 classes of hospital system” [8,9] as shown in Fig 1. The general population is free to choose health care facilities without being restricted by a gatekeeping mechanism [10]. In rural areas, township health centers (THCs) and village clinics offer grass roots primary care and public health services. In urban areas, these services are provided by community health centers (CHCs) and community health stations [5,11].

In the first 11 months of 2016, the number of primary care visits decreased by 0.6% to 3.93 billion [12], thus sustaining the low utilization rates of lower level facilities [6]. Over the same period, the number of hospital visits increased by 5.6% compared to 2015, to a total of 2.89 billion [12]. Moreover, patients in China increasingly access the health system at hospitals on level 2 and 3 [3], which has resulted in overcrowding of level 3 hospitals particularly. This is further illustrated by the “three longs and one short” phenomenon [13]: long waiting time for registration, long waiting time to prepay the charges, long waiting time for the appointment with a doctor, but a short appointment duration. This situation has generated great patient discontent [14] and caused deterioration of the patient-doctor relationship [15].

The situation and corresponding challenges to effectiveness and efficiency may be further amplified by future societal developments such as increased welfare, expanded health insurance coverage, rapid urbanization, and aging of the population [16,17]. Therefore, in order to develop a sustainable, cost-effective health system, ongoing Chinese health system reforms target strengthening primary care facilities and directing patients toward the lower levels of care. Examples are the introduction of gradient reimbursement schemes [4,7,18] and the continuously increasing resources spending on primary care infrastructure [7,19].

Scientific understanding of the effect of such interventions is limited [12–14] and this effect depends highly on the influence on the access choices of the population. While some empirical [20,21] and theoretical studies [22–24] address this topic, scientific research focused on the influence of reform interventions on access choices is scarce. Moreover, the difficulty that actual reforms have in effectively directing access choice indicate that currently available theory and evidence may be insufficient to inform policy making. The apparent complexity of the relationships between reform intervention and access choice or health-seeking behavior calls for an empirical evidence base, which can facilitate the design and implementation of more effective interventions and help researchers develop empirically grounded theory. With these objectives, we present a systematic review of empirical evidence on factors influencing access level choice.

## Methods

We conducted this systematic review in accordance with National Health Service Centre for Reviews and Dissemination Guidance for undertaking reviews in health care [25] (see S1 Appendix). We used the Preferred Reporting Items for Systematic Reviews and Meta-Analyses (PRISMA) [26] for reporting purposes.

### Search strategy

We searched Embase, Medline, Web of Science, and Pubmed for English language articles, and three large Chinese databases (CNKI, VIP and Wanfang) for articles in Chinese. As the new round of health reform starting in April 2009 [4] brought considerable change, we sought articles that investigated Chinese patients’ choice of health care access levels between April 2009 and January 2016. The detailed search strategies (see S1 Text) were executed by a medical librarian and the first author.

### Study selection

The following inclusion criteria were applied during study selection: (1) primary empirical studies; (2) research aimed at identifying factors that influence patients’ choice of health care facility access level, and how these factors affect the choice of level; (3) data collected after April of 2009; (4) study population is Chinese residents; (5) written in English or Chinese language; (6) published in a peer-reviewed journal.

Two authors (YL and one other, either QK or SY) screened each record independently. The first round of study selection was to screen titles and abstracts of primarily identified articles based on the inclusion criteria. In the case of disagreement between reviewers, the articles were included. In the second round, the full text of each selected article was assessed for eligibility using the inclusion criteria. Eligibility assessment discrepancies were discussed until consensus was reached. Twice, we found two articles reporting analysis of the same data. In both cases, we combined the findings and presented them under the earliest included article (reducing the number of studies from 47 to 45).

### Data extraction

We developed a first form to extract the characteristics of each study by following the broad format of PICO (Population, Intervention, Comparison and Outcomes) guideline [25], and made necessary adaptations to the study characteristics by adding more information of interest. We then developed a second form to extract findings regarding the factors mentioned in each study. Factors were labeled by type (patient, provider and context); we also allowed new factor types. When including studies that considered patient choice with respect to provider facilities rather than the level of the provider facilities, we considered the facility level only.

Some included studies use qualitative methods, others use quantitative methods, and a third subset uses mixed methods. We thus conducted a narrative synthesis, which is a systematic review methodology that appropriately accommodates the heterogeneity of the included articles [25]. For the quantitative results, we extracted only the information regarding associations reported as significant.

For each of the factors and choices reported, we extracted whether they were stated (e.g. in interviews or questionnaires) or revealed (e.g. on actual visits) given that revealed factors and choices may be considered to provide stronger evidence than stated factors and choices [27]. Therefore, we distinguished four evidence types: a revealed factor for a revealed choice (RR), a stated factor for a revealed choice (RS), a stated factor for a stated choice (SS), and a revealed factor for a stated choice (SR). We provide further insight into the workings of each factor by identifying whether it positively or negatively affected choice for a certain level. To this purpose, we speak of attraction when a factor is positively associated with choice for a certain level, and of repulsion when the association is negative.

When synthesizing the data, we firstly considered whether the evidence reported in the studies was conclusive or inconclusive. Evidence is classified as conclusive if the research methods employed provide an unambiguous answer to the stated empirical research question (e.g. the hypothesis is accepted) [28]. If the results of the included studies contradict each other, the review classifies them as inconsistent. Otherwise, they are considered to be consistent.

### Quality assessment

We appraised the methodological quality of the studies using the validated, widely used Method Appraisal Tool (MMAT) [29,30]. This tool has four specific criteria for each study type. The overall quality score of each article is presented by the number of criteria it meets [31].

## Results

### Characteristics of the included studies and quality assessment

**Fig 2:**
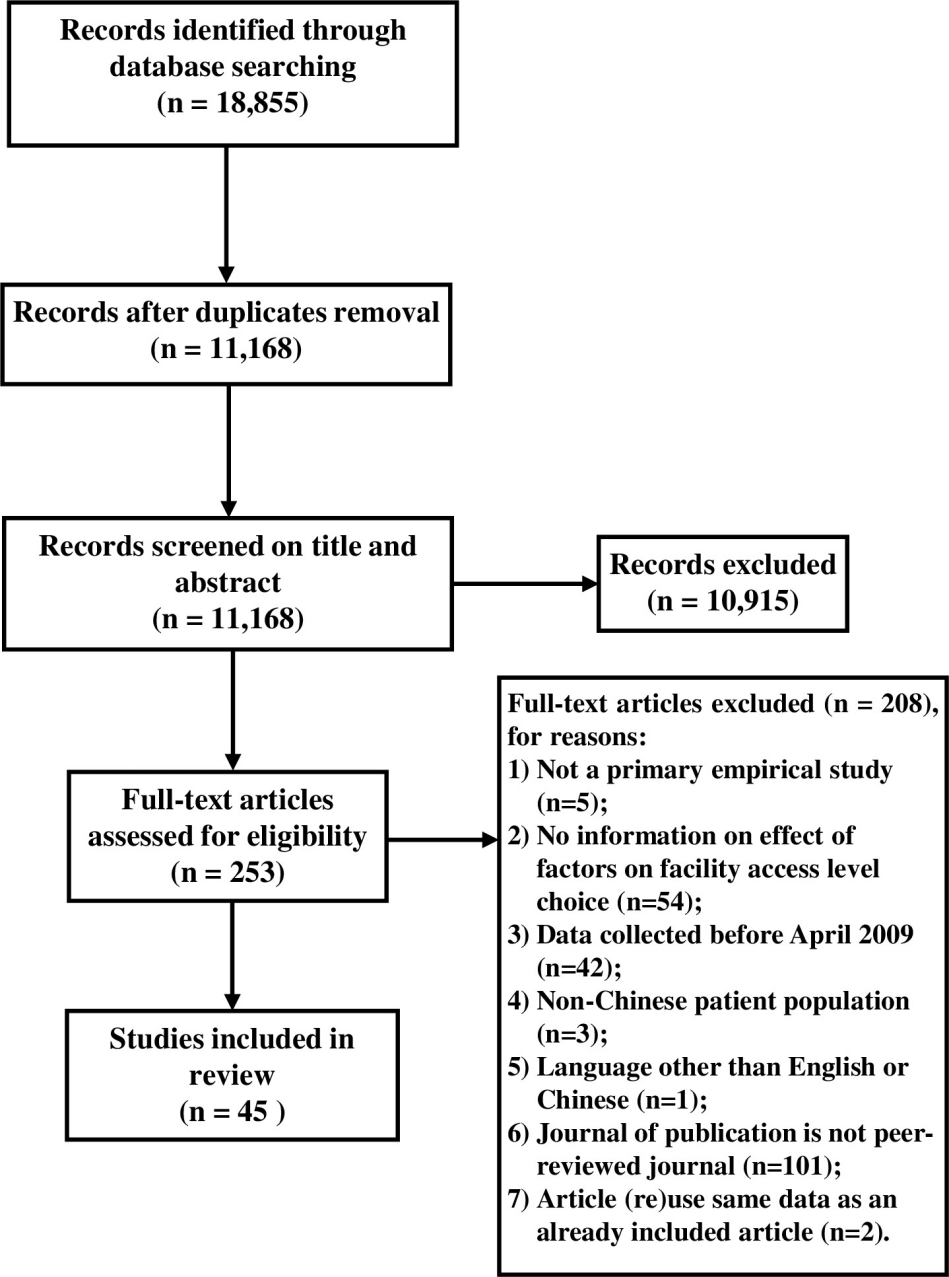
PRISMA 2009 flow diagram.

As shown in Fig 2, we initially retrieved a total of 18,855 records. After removing duplicates and applying the inclusion criteria, we were left with a final set of 45 articles [23,24,32–74]. Table 1 shows the basic information of these articles and the results of the quality assessment.

**Table 1:** Overview of included studies.

Study	Study design	Data collection method	Respondents^a^	Sample site	Sample size	Study quality^b^	Evidence revealed or stated^c^
Cheng et al. 2015 [53]	Cross-sectional study using mixed methods	Interview	P, O	NA	1,917 individuals	**	SR
Jing et al. 2015 [33]	Longitudinal study using mixed methods	Patient registration data, questionnaire, focus group interview, literature review	P, O	Shanghai	314 individuals (questionnaires), 80 individuals (interviews)	**	RR
Jing et al. 2015 [34]	Cross-sectional study	Questionnaire	P	Shanghai	1,200 individuals	****	SS, SR
Kuang et al. 2015 [65]	Cross-sectional study	Survey including PCAT questions	P	Guangdong	1,645 individuals	***	RR
Liu et al. 2014 [66]	Longitudinal study	Survey	P	Sichuan	976 individuals	***	RR
Tang 2012 [67]	Cross-sectional study	Residence household survey	O	Nationwide	4,853 individuals	***	RR
Zeng et al. 2015 [68]	Cross-sectional study	Survey	O	Guangdong	736 individuals	****	SR
Zhou 2014 [54]	Cross-sectional study using qualitative methods	Interview and patient registration data	P, O	Zhejiang and Yunnan	80 health workers, 80 service users	****	SS
Dong et al. 2014 [35]	Cross-sectional study	Questionnaire, residence household survey	P, O	Nationwide	88,482 individuals	***	RR
Yang et al. 2014 [69]	Cross-sectional study	Survey	P	Guangdong	51,501 individuals	***	SS, SR
Zhou et al. 2014 [70]	Cross-sectional study	Survey	O	Guangdong	12,800 individuals	***	SS, SR
Li et al. 2014 [36]	Cross-sectional study	Questionnaire	P	Guangdong	787 individuals	***	RR
Wang et al. 2012 [55]	Cross-sectional study	Interview	O	Shandong, Shanxi, Henan, Shannxi, Gansu, Ningxia, and Inner Mongolia	15,698 individuals	****	RR
Zhang et al. 2011 [56]	Longitudinal study	Interview, regular hospital reports	P	Beijing	NA	***	RR
Jiang et al. 2013 [57]	Cross-sectional study	Interview	O	NA	2,093 individuals	****	SR
Powell-Jackson et al. 2015 [32]	Cluster randomized experiment embedded in quasi-experimental study	Questionnaire	O	Ningxia	54,143 individuals	***	RR
Wang et al. 2014 [37]	Cross-sectional study	Questionnaire	O	Guangdong	162,464 individuals	***	RR
Zhang et al. 2014 [63]	Longitudinal study	Patient registration data	P	Jiangsu	14,169 individuals	***	RR
He et al. 2014 [38]	Cross-sectional study	Questionnaire	P	Jilin	12,862 individuals	****	RR, RS
Bao 2013 [39]	Cross-sectional study	Questionnaire	O	Shanxi	668 individuals	****	RS
Wang et al. 2011 [40]	Cross-sectional study	Questionnaire	P	Shandong	850 individuals	***	SR
Ji et al. 2015 [41]	Cross-sectional study	Questionnaire	P	Beijing	2,632 individuals	***	RR
Zhao and Zhang 2012 [71]	Cross-sectional study	Residence household survey	O	Beijing	2,556 individuals	***	RR
Guo et al. 2012 [42]	Cross-sectional study	Questionnaire	O	Shandong	2,274 individuals	**	SR
Chen et al. 2013 [23]	Cross-sectional study	Questionnaire	P	Beijing, Henan, Chongqing, and Anhui	3,792 individuals	***	SR
Jin et al. 2011 [43]	Cross-sectional study	Questionnaire	P	Shandong	3,500 individuals	***	SS
Huang et al. 2012 [44]	Cross-sectional study	Questionnaire	O	NA	6,024 individuals	****	RR, RS
Li et al. 2015 [45]	Cross-sectional study	Questionnaire	O	Guangdong	435 individuals	***	SS, SR
He et al. 2011 [58]	Longitudinal study using mixed methods	Medical insurance registration data, focus group interview	P, O	Anhui	NA	**	RR
Zhou et al. 2011 [25]	Cross-sectional study	Interview	P	Guangdong	661 individuals	****	RR
Xia et al. 2015 [46]	Cross-sectional study	Questionnaire	O	Sichuan	307 individuals	***	SS, SR
Yao et al. 2014 [47]	Cross-sectional study	Questionnaire	P	Guangdong	1,464 individuals	***	RS, SR
Gong and Cao 2011 [48]	Cross-sectional study	Questionnaire	O	Shandong	2,274 individuals	****	SR
Zhang et al. 2014 [49]	Cross-sectional study	Questionnaire	O	Xinjiang	768 individuals	***	SS, SR
Zeng et al. 2012 [64]	Longitudinal study	Patient registration data	P	Guangdong	NA	*	RR
Wang et al. 2012 [72]	Cross-sectional study	Survey	O	Zhejiang	274 individuals	****	SS, SR
Wang et al. 2014 [50]	Cross-sectional study	Questionnaire	O	Sichuan	4,201 individuals	****	RR, RS
Tian et al. 2012 [59]	Longitudinal study using mixed methods	Medical insurance registration data, focus group interview	P, O	Yunnan	NA	**	RR
Luo et al. 2015 [60]	Longitudinal study using mixed methods	Medical insurance registration data, focus group interview, literature review	P, O	Hubei	NA	**	RR
Xie et al. 2010 [51]	Cross-sectional study	Questionnaire	O	Jiangsu	397 individuals	***	SS, SR
Guo et al. 2015 [61]	Longitudinal study	Medical insurance registration data, focus group interview	P, O	Heilongjiang	NA	***	RR
Chen et al. 2013 [62]	Longitudinal study	Medical insurance registration data, interview	P, O	Shandong	4,571 Individuals, 15 medical Institutions	***	RR
Wei and Xiao 2014 [73]	Cross-sectional study	Survey	P, O	Anhui	498 individuals	***	SR
Zhuang et al. 2011 [52]	Cross-sectional study	Questionnaire	O	Guangdong	40,053 individuals	****	SR
Ma et al. 2015 [74]	Cross-sectional study	Questionnaire	O	Zhejiang	952 individuals	***	SS

^a^ P = patients or service users; O = general population.

^b^ The MMAT score is 25% (*) when 1 criterion is met; 50% (**) when 2 criteria are met; 75% when 3 criteria are met (***); and 100% when 4 criteria are met (****).

^c^ RR = revealed factor for revealed choice; RS = stated factor for revealed choice; SS = stated factor for stated choice; SR = revealed factor for stated choice.

**Fig 3:**
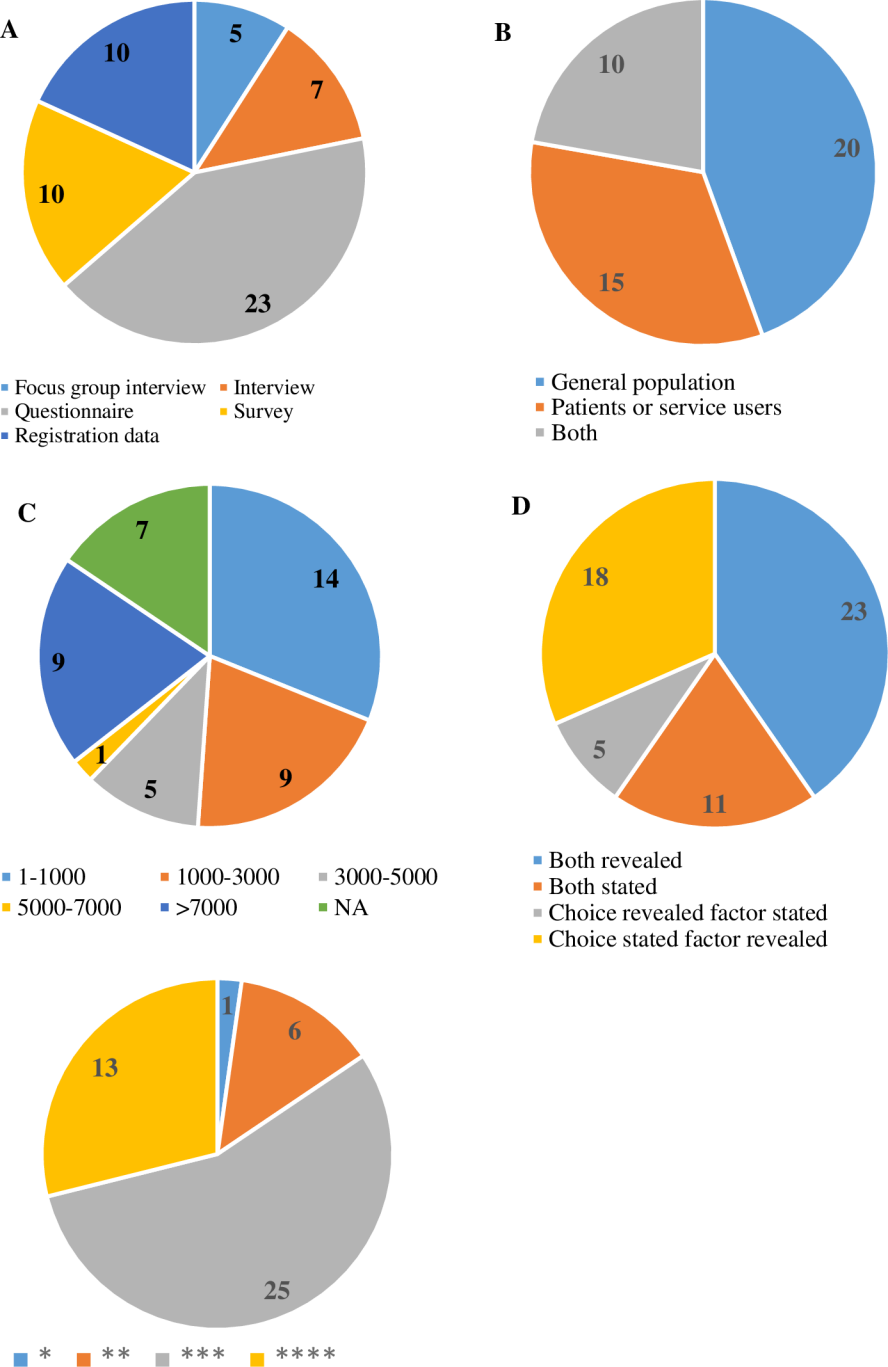
Summary of study characteristics. (A) Distribution of data sources. (B) Distribution of respondent types. (C) Distribution of sample sizes. (D) Evidence types. (E) Distribution of quality assessment scores. *The number in each slice of the pie chart indicates the number of studies with the corresponding attribute of interest.

**Fig 4:**
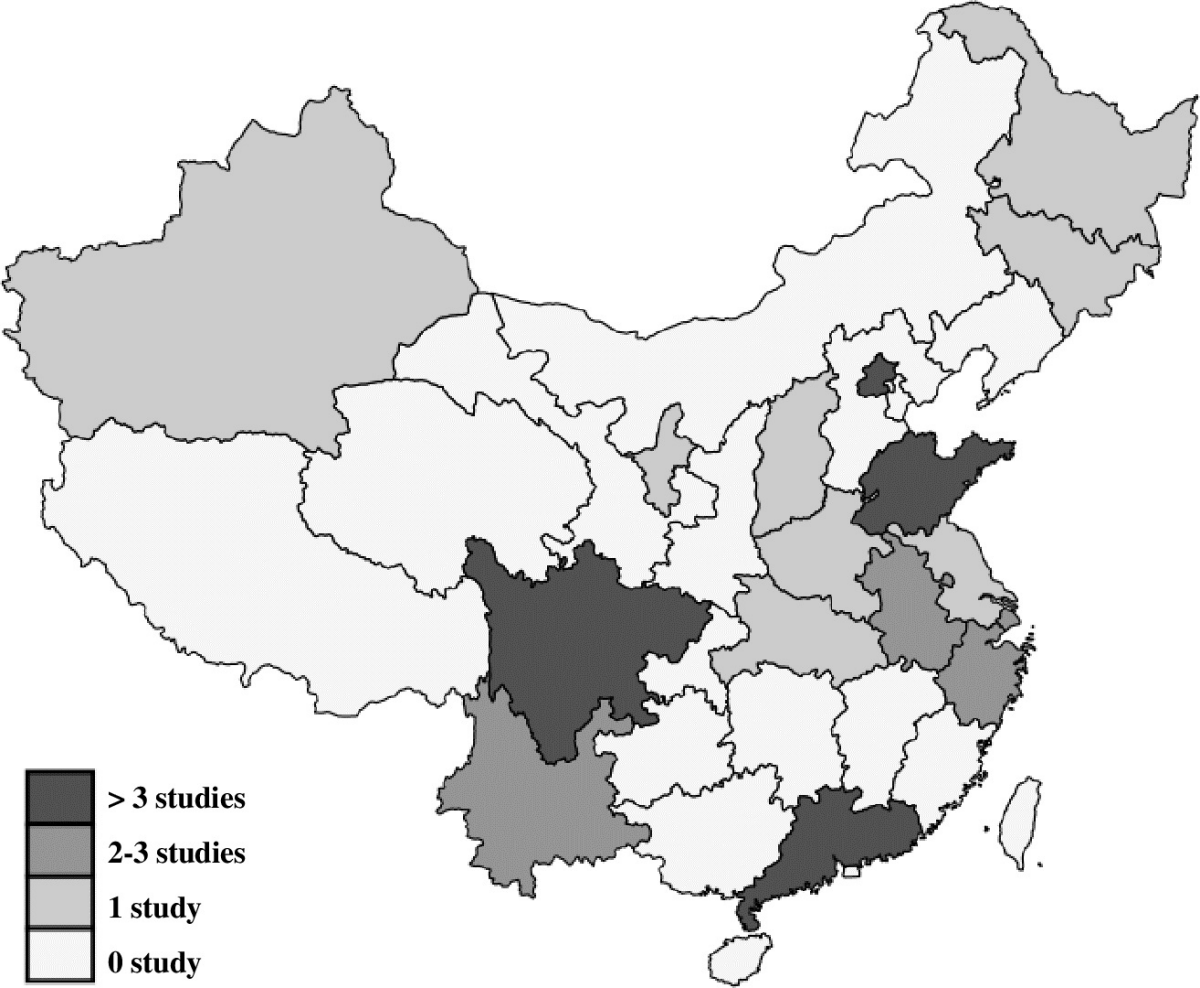
Geographic distribution of study sites except for the studies conducted nationwide (n = 2) or without indication of location (n = 4). Caption credit: The map of mainland China in Fig 4 was created using Stata software (StataCorp. 2017. Stata Statistical Software: Release 15. College Station, TX: StataCorp LLC).

For ease of exposition, Figs 3 and 4 summarizes the characteristics of the studies. Except for one quasi-experimental study, all studies are observational (n = 44). The data are collected mostly from questionnaires (n = 23). Other data sources include interviews (n = 12), registration databases (n = 10) and combinations of questionnaires and interviews (n = 10). The number of studies that take the general population as respondents (n = 20) is slightly larger than those with patients or service users as respondents (n = 15). 10 studies have both types of respondents. The reported sample size varied from 80 to 162,464. 14 Studies have a sample size of less than 1,000 individuals.

A majority of the studies reports results on revealed factors, either for revealed choices (n = 23), or for stated choices (n = 18). 11 Studies report stated factors for stated choices and five studies report stated factors for revealed choices. The most frequently studied provinces are Guangdong (n = 11), Shandong (n = 6), Beijing (n = 4) and Sichuan (n = 4; including Chongqing). The MMAT quality score was 100% for 13 studies, 75% for 25 studies, 50% for six studies and 25% for one study.

### Identified factors influencing patient’s choice

The factors identified in the studies are presented with brief notes in Table 2, and in detail in Table 3 and S2 Text. We found 15 patient factors, nine provider factors, and four context factors. In addition, we found six factors of a new type, which we call ‘composite factors’. These include attributes of more than one of the other three types of factors.

**Table 2:** Identified factors with brief explanations.

Factors	Explanation
**Patient factors**	
**Age**	Age
**Health insurance status**	Health insurance status in terms of enrollment, type and coverage
**Income**	Household income or individual income
**Education**	Education level
**Pre-existing disease**	Onset of pre-existing disease when making choice
**Disease severity**	Disease severity
**Gender**	Gender
**Marriage status**	Marriage status
**Place of residence**	Rural or urban; geographic location in China
**Migration**	If the study sample was migrated from original birth location
**Occupation**	Employment or working place
**Health literacy**	Ability to acquire and utilize health knowledge
**Ethnicity**	Han or minorities
**Life style**	Doing physical exercise
**Anxiety before seeing doctor**	Anxiety before seeing doctor
**Provider factors**	
**Drug**	Drug availability; implementation of essential medicine list
**Medical equipment**	Degree of depreciation of medical equipment
**Service price/cost-effectiveness**	Service price/cost-effectiveness
**Service attitude**	Medical professional’s service attitude
**Service scope**	Variety of services provided by the facility, including the availability of doctors specialized in chronic disease treatment
**Physical environment in facility**	The comfort level of the physical environment in facility
**Medical staff**	Medical skill and personal connection
**Service convenience**	Waiting time, difficulty in getting admitted and convenience of procedure
**Application of health information technology**	Application of health information technology
**Context factors**	
**Capitation/gatekeeping**	In the payment reform, the payment method was changed to capitation
**Freedom of service choice**	Freedom of choosing health care facilities formulated in health insurance policy
**Salary reform on health workers**	Initiation of payment reform on medical staffs
**Public campaign/interaction of social capital**	Exposure to reform publicity campaigns
**Composite factors**	
**Perceived quality of care**	Perceived poor clinical outcome
**Transportation convenience/distance**	Distance from home to facility
**Reimbursement rate/insurance coverage**	Difference in reimbursement rates between higher and lower level facilities
**Previous experience with provider**	Previous medical experience of visiting primary care facilities or receiving inpatient care
**Awareness about the facility**	Awareness of primary level facilities or the roll-out of referral policy
**Disease diagnosis**	Having the purpose of “confirmation of disease diagnosis”

**Table 3:** Studies that identified factors differentiated by evidence type and quality scores.

Factors	Total number of studies that found this factor	Number of studies by evidence type^a^				Number of studies in each scoring category^b^			
		RR	SS	RS	SR	*	**	***	****
**Patient factors**									
**Age**	18	9 [24, 35, 38, 44, 56, 62, 63, 65, 71]	0	0	9 [23, 34, 42, 47, 53, 57, 68, 72, 73]	0	2	9	7
**Health insurance status**	15	9 [24, 37, 38, 41, 44, 55, 60, 61, 71]	2 [54, 69]	0	4 [23, 42, 47, 68]	0	2	7	6
**Income**	13	6 [35, 37, 44, 50, 55, 62]	0	0	7 [42, 45, 47, 57, 69, 72, 73]	0	1	7	5
**Education**	11	4 [37, 38, 44, 71]	0	0	7 [34, 42, 45, 47, 69, 72, 73]	0	1	6	4
**Pre-existing disease**	8	4 [37, 38, 44, 65]	2 [44, 45]	0	3 [46, 68, 70]	0	0	5	3
**Disease severity**	7	3 [44, 56, 63]	3 [45, 46, 49]	0	1 [40]	0	0	6	1
**Gender**	4	3 [24, 61, 63]	0	0	1 [48]	0	0	2	2
**Marriage status**	4	2 [62, 71]	0	0	2 [57, 68]	0	0	2	2
**Place of residence**	4	1 [50]	0	0	3 [47, 57, 69]	0	0	2	2
**Migration**	3	2 [36, 65]	0	0	1 [68]	0	0	2	1
**Occupation**	3	1 [65]	0	0	2 [57, 73]	0	0	1	1
**Health literacy**	2	0	1 [72]	0	1 [69]	0	0	1	1
**Ethnicity**	1	0	0	0	1 [49]	0	0	1	0
**Life style**	1	0	0	0	1 [69]	0	0	1	0
**Anxiety before seeing doctor**	1	1 [67]	0	0	0	0	0	1	0
**Provider factors**									
**Drug**	13	4 [58, 59, 62, 64]	5 [49, 54, 69, 70, 72]	2 [39, 43]	3 [23, 48, 72]	1	2	6	4
**Medical equipment**	8	0	3 [69, 70, 74]	3 [39, 43, 47]	2 [42, 48]	0	1	5	2
**Service price/cost-effectiveness**	7	1 [62]	4 [34, 54, 70, 74]	0	2 [42, 72]	0	1	3	3
**Service attitude**	6	0	4 [34, 51, 69, 70]	1 [47]	1 [48]	0	0	4	2
**Service scope**	3	1 [24]	0	2 [39, 47]	0	0	0	1	2
**Physical environment in facility**	4	0	2 [69, 70, 74]	1 [39]	0	0	0	3	1
**Medical staff**	3	1 [62]	1 [51, 74]	0	0	0	0	3	0
**Service convenience**	2	0	2 [34, 70]	0	0	0	0	1	1
**Applying of health information technology**	2	1 [66]	0	0	1 [69]	0	0	2	0
**Context factors**									
**Capitation/** **gatekeeping**	2	1 [33]	1 [51]	0	0	0	1	1	0
**Freedom of service choice**	2	0	2 [34, 51]	0	0	0	0	1	1
**Salary reform on health workers**	1	0	1 [54]	0	0	0	0	0	1
**Public campaign/** **interaction of social capital**	1	0	0	0	1 [34]	0	0	0	1
**Composite factors**									
**Perceived quality of care**	16	0	7 [34, 51, 52, 54, 69, 70, 74]	6 [38, 39, 43, 44, 47, 50]	3 [23, 42, 48]	0	1	7	8
**Transportation convenience/distance**	9	2 [56, 61]	4 [49, 51, 52, 69, 70]	1 [45]	1 [48]	0	0	6	3
**Reimbursement rate/ insurance coverage**	7	6 [32, 44, 60–63]	0	0	1 [48]	0	1	4	2
**Previous experience with provider**	2	1 [50]	0	0	1 [46]	0	0	1	1
**Awareness about the facility**	2	1 [50]	0	0	1 [51]	0	0	1	1
**Disease diagnosis**	1	0	0	1 [43]	0	0	0	1	0

^a^ RR = revealed factor for revealed choice; RS = stated factor for revealed choice; SS = stated factor for stated choice; SR = revealed factor for stated choice.

^b^ The MMAT score is 25% (*) when 1 criterion is met; 50% (**) when 2 criteria are met; 75% when 3 criteria are met (***); and 100% when 4 criteria are met (****).

The most frequently indicated patient factors are age (n = 18 studies), health insurance status (n = 15 studies), income (n = 13 studies) and education (n = 11 studies). The most often found provider factors include drug availability (n = 13 studies), medical equipment (n = 8 studies), service price/cost-effectiveness (n = 7 studies) and service attitude (n = 6 studies). Context factors were reported less frequently: capitation/gatekeeping (n = 2 studies), freedom of service choice (n = 2 studies), salary reform on health workers (n = 1 study) and public campaign/interaction of social capital (n = 1 study). The most frequently identified composite factors are perceived quality of care (n = 16 studies), transportation convenience/distance (n = 9 studies) and reimbursement rate/insurance coverage (n = 7 studies).

### Effects of identified factors on patient’s choice

Table 4 gives an overview of whether factors attracted or repulsed patients, and for which facility levels. The reader may first notice that the synthesized evidence on patient factors age, insurance status, pre-existing disease, disease severity, gender, marital status, and location of residence is inconclusive. For instance, there is evidence that older people are repulsed by both lower and higher level facilities while female patients are attracted by both lower and higher level facilities.

**Table 4:** Patient factors that create attraction or repulsion to choose lower level or higher level health care facilities.

Factors	Lower level facilities^a^		Higher level facilities^b^	
	Attract	Repulse	Attract	Repulse
**Patient factors**				
**Age**	Older (11)		Older (5)	-
**Insurance status**	Having insurance or knowledge of insurance (6); having New Cooperative Medical Scheme insurance among other types of insurance (3)	Having insurance (4)	-	-
**Income**	-	Higher income (12)	-	Lower income (1)
**Education**	-	-	Higher level (11)	-
**Pre-existing disease**	More onset of diseases in recent 3 months (1); chronic condition (2)	Chronic condition (5)	-	-
**Disease severity**	Perceived minor disease (6)	-	Perceived minor disease (1)	-
**Gender**	Female (1)	-	Female (3)	-
**Marriage status**	Married (1)	-	Married (2); widowed (1)	-
**Place of residence**	Rural area (2)	-	Rural area (1); central and western regions compared to eastern regions (1)	-
**Migration**	Immigrants (2); immigrants with no intention to reside permanently or with fewer than 5 years residency (1)	-	-	-
**Occupation**	Retired people (1); working for governments, worker or peasants (1)	-	Working at large enterprises (1)	-
**Health literacy**	-	Obtaining health knowledge (1)	Having habit of seeking help (1)	-
**Ethnicity**	Han (1)	-	-	-
**Life style**	-	Having habit of doing physical exercise (1)	-	-
**Anxiety before seeing doctor**	-	-	High level (1)	-
**Provider factors**				
**Drug**	Low or unified price of drug on the essential medicine list (5)	Limited drug variety (7)	-	-
**Medical equipment**	-	Obsolete equipment (4)	Better equipment than lower level facilities (2)	-
**Service price/cost-effectiveness**	Lower price and more cost-effective (6)	High price (1)	-	-
**Service attitude**	Good attitude (5)	Bad attitude (1)	-	-
**Service scope**	-	Limited service types (2)	-	-
**Physical environment in facility**	-	Uncomfortable environment (4)	-	-
**Medical staff**	Personal connections with staff (1)	Not acquainted with the staff (1)	-	-
**Service convenience**	Convenience in general and shorter waiting time than higher level facilities (2)	-	-	-
**Application of health information technology**	Application of community health report (2)	-	-	-
**Context factors**				
**Capitation/gatekeeping**	Implementation of capitation and gatekeeping (1)	Complicated procedure of referral (1)	-	-
**Freedom of service choice**	-	Sign contract of designated family doctor prohibits the freedom of service choice (2)	-	-
**Salary reform on health workers**	-	Implementation of fixed salary policy on health workers (1)	-	-
**Public campaign/interaction of social capital**	Exposure to publicity campaign or high score in social interaction of social capital (1)	-	-	-
**Composite factors**				
**Perceived quality of care**	Reliable skill (2)	Perceived low quality of care (14)	-	-
**Transportation convenience/** **distance**	Short distance from home and convenient transportation (7)	-	-	-
**Reimbursement rate/insurance coverage**	Larger reimbursement rate and expanded benefit package at lower level facilities (3)	Enlarged reimbursement rate at lower level facilities (4)	-	-
**Previous experience with provider**	Having previous experience at low level facilities (1)	No inpatient experience (1)	-	-
**Awareness about the facility**	Having knowledge of community health center or township health center (1)	Having no knowledge of community health center or township health center (1)	-	-
**Disease diagnosis**	-	-	Trust higher level facilities for this purpose (1)	-

* Numbers in the parentheses represent the number of studies that found this effect.

^a^ ‘Attract’ refers to evidence that the factor is positively associated with the choice for lower levels, in which case we speak of attraction; ‘Repulse’ refers to evidence that the factor is negatively associated with the choice for a lower level, in which case we speak of repulsion. Empty space represents no evidence was found.

^b^ As under a, but for higher level facilities.

Patient factors positively associated with lower level attraction are: lower education level, retired patients/working for governments/peasants, and patients of the Han ethnicity. Attracting lower level provider factors are lower and unified drug price, service price, and good service attitude. Composite factors and context factors which cause lower level facilities to attract patients are the short distance to home, transportation convenience, implementation of capitation and gatekeeping, previous experience with provider, knowledge about CHCs or THCs, being exposed to publicity campaigns, and high social capital.

Repulsive patient factors for lower level facilities are health knowledge, habit of seeking help from higher level facilities, regular physical exercise, and high anxiety to seeing a doctor. The most repulsive provider factors for low level facilities are limited drug variety, obsolete medical equipment and discomfort. The limited service portfolio of lower level facilities is another repulsing factor. The composite factor perceived poor quality is frequently reported to repulse patients, although some studies report that patients consider lower level facilities to be reliable. Repulsing context factors for level facilities are complexity of the referral procedure, and limited freedom of choice following from general practitioner contracts. The implementation of salary reform at primary level facilities caused them to repulse.

The included studies provide little evidence for factors explicitly addressing access at higher level facilities. Patient factors that attract to higher levels are higher level of education, habit of seeking medical care at higher level facilities, and employment at a large enterprise. The purpose of seeking confirmation of disease diagnosis also stimulated patient flow towards higher level facilities. The most attractive provider factors are drug variety, medical equipment, and physical environment. Other than high price, patient crowding, and difficulty to see a doctor, we found no evidence on repulsion with regard to higher level facilities.

## Discussion

### Main findings and interpretations

We first summarize the evidence on the factors influencing health system access level choice, thus outlining the contribution to the necessary advancement of scientific understanding and development of evidence-based interventions. In the process, we interpret the evidence in relation to previously reported literature and the ongoing reforms. A general reflection on relevant theory and policy is subsequently presented.

Patient factors are the most reported. Interestingly, while the patient factors age, health insurance status, income, education, pre-existing condition, and disease severity received most attention, the evidence for these factors is inconclusive. Thus, based on the review, for instance, we cannot conclude that elderly patients choose primary care more frequently, or less frequently.

The evidence on the factor education is conclusive. Better education is associated with accessing higher levels (as is further supported by the association between health literacy and access at higher levels). The evidence on income level and disease severity is almost conclusive. Most of the studies (12/13) found that people with higher income are more likely to choose higher level facilities. These findings suggest that inequality in the health system access persists [4]. Geography may operate as an underlying factor, as patients from remote rural areas tend to have lower incomes and live further away from higher level facilities [75–77]. Evidence for the patient factor disease severity is also almost conclusive. Five out of six studies investigating disease severity reported that people with perceived minor diseases preferred lower level facilities, while people with more severe conditions preferred high access levels. This might be explained by the limited trust people attach to lower level facilities and might relate to the composite factor perceived quality discussed below.

The provider factors drug variety, equipment, followed by service price, and service attitude received the most attention. Limited drug variety and lack of equipment at lower level facilities cause patients to access higher levels. These findings echo earlier evidence that patients attach much importance to provider factors believed to be associated with effectiveness, i.e. clinical outcomes [22]. In terms of the Structure-Process-Outcome model to explain quality of care developed by Donabedian [78], these factors relate to structures which patients appear to associate with poor outcomes [7] and hence cause lower levels to repulse [79]. From a policy perspective, this suggests that interventions to improve the structure, for instance improving drug variety by extending the essential medicine list, or by investing in equipment, may help to direct patient flows toward the lower levels. The recent encouragement of health authorities to invest in independent regional diagnostic medical imaging centers [80] may result in similar effect.

Factors of the context type that influence patient choice mostly relate to gatekeeping and referral policies. The perceived high complexity of referral procedures, and limited freedom of access choice when registering with a general practitioner cause lower levels to repulse. This suggests that policy interventions to improve ease of referral can help direct patient flows towards lower levels.

This systematic review has produced a new factor type: composite factors, including such factors as perceived quality of care, transportation convenience, travel distance, and reimbursement rate that are frequently reported to influence access choice both in China and elsewhere (e.g., in relation to bypassing nearby facilities [81,82]). Factors are classified as composite when they relate to combinations of patient attributes, provider attributes and/or context attributes.

Current reforms are intended to direct patient flow by changes in coverage and diversifying reimbursement rates [83]. Interestingly, we found that when the reimbursement rate or coverage became more generous, patients tended to choose higher level facilities more frequently, even when lower level reimbursement changes were larger. Apparently, copayment reductions at higher levels have more effect than relatively higher reductions at lower level facilities. This is congruent with patient factor findings where higher income and education are positively associated with access at higher levels. These results may suggest an underlying affordability factor to be at work, causing patients who can afford it to choose access at higher levels. However, our review did not reveal any results on the relationships between factors. Current understanding of (and evidence for) interactions among factors is poor. While this identifies a relevant area for future research, it also calls for modesty when deriving policy implications from this review.

As a more general reflection, our results reveal that most of the evidence is in regard to factors that push patients away from the lower levels (repulsion) and cause them to seek care at higher levels. Lack of drug variety, (obsolete) medical equipment, and perceived poor quality are the most important among such factors. Hence our review indicates that for many Chinese citizens, the lower levels are not the ‘first point of access’ that primary care is intended to be according to the Declaration of Alma Ata [84], which explicitly mentions primary health care to “form an integral part of a country’s health system, of which it is the central function and main focus” and “first level of contact of individuals, the family and community with the national health system”. The identified factors and evidence allow for some corresponding theoretical interpretation for this finding.

Classifying factors as attracting or repulsing relates to push and pull factor theory, as for instance considered by Bansal et al. [85] to explain why people migrate to other countries or switch service providers. While they focus on provider related push and pull factors, their framework also includes other (mooring) factors which relate to the person (patient) and context [86]. Herzberg [87] considers push and pull factors to explain why employees leave their employer organization. He relates the factors to Maslow’s needs hierarchy [88] and considers push factors to be more fundamental as they relate to basic physiological and safety needs.

Building on these related theories, we may interpret provider related factors such as drug variety, equipment, and perceived quality to push patients away from the (default) primary care, because primary care facilities are not trusted to safely address basic patient health needs. It may also explain why disease severity pushes toward higher level facilities, as more severe diseases form a larger threat to basic needs. Moreover it suggests that patients who can afford will often choose access at higher levels, as indicated by the evidence on the factors higher income, education, and reimbursement.

Reasoning along these lines, one may deduce that further economic development, and more generous reimbursement will increase the number of patients who can afford to access higher levels, thus pushing an even larger population away from primary care and to overcrowded high level hospitals. The evidence on the patient flow data in 2016 [12] provided in the introduction supports these arguments. From a policy perspective, this stresses the importance of lower level ability to provide safe health services for fundamental health needs, and to be trusted to refer to when required to address fundamental health needs.

### Limitations

As the context of health policy changes rapidly in China [16,89] and new developments advance rapidly (e.g. encouragement of private hospitals [90] and innovations such as e-consults [91,92]), the validity of some of the evidence provided by this systematic review reduces over time.

Second, most of the evidence is derived from observational designs without adjustment for confounders or consideration of interactions among factors. Hence, our review delivers little evidence that demonstrates causal relationships between factors and choice. Likewise, the designs of the included studies varied considerably, preventing us from presenting synthesized findings on effect sizes, as might be obtained through meta-analysis when enough high quality quantitative studies are available. Obviously, effect sizes forms an important direction for future research as well.

Eastern China is overrepresented in the included studies. This calls for caution when applying the findings nationwide, or in Western Chinese contexts and other under-studied regions. In addition, it calls for further research in other parts of China.

## Conclusions

The present problem in the Chinese health system of overcrowding in higher level hospitals and underuse of lower level facilities is driven by patient access choices. However, current scientific evidence on the factors influencing patient access choices is limited. This systematic review reveals that higher income, higher education, and urbanization are associated with access at high levels. As urbanization and income are increasing in China, as is the education level, our results suggest that current problems may worsen, and may further threaten the effectiveness and efficiency of health services in China.

Patients appear to be pushed towards higher level facilities by the perceived inability of lower level facilities to address basic health needs. This inability is predominantly expressed by the factors lack of drug variety, obsolete equipment and perceived poor quality. From a policy viewpoint, our results suggest that improving lower level structures and quality perceptions of lower level institutions, in combination with a trusted referral system, may promote access at lower levels. This can help the primary care to regain its intended central function and improve the Chinese health system at large.

As the identified evidence is inconsistent for many identified factors, it is likely that contextual factors are not yet well understood, and that interactions between factors play a role. As of yet, these interactions have not received attention. Moreover, effect sizes remain uncertain, and very little evidence exists for western China. Therefore, the scientific evidence base to support policy interventions aiming to promote the utilization of primary care facilities in China deserves extension.

## Supporting information

S1 Text Search strategy. (DOCX)

S2 Text Detail description of identified factors influencing patient’s choice. (DOCX)

S3 Text Background information on the Chinese health system. (DOCX)

S1 Checklist PRISMA 2009 checklist. (DOC)

S1 Appendix National health service centre for reviews and dissemination guidance for undertaking reviews in health care. (PDF)
