# Supplementary material for: Factors influencing choice of health system access level in China: A systematic review
Source: PLoS One. 2018 Aug 10;13(8):e0201887. doi: 10.1371/journal.pone.0201887 (PMC6086423; doi:10.1371/journal.pone.0201887)
Supplement: S1 Text — (DOCX) [file pone.0201887.s001.docx]

**S1 Text**

**Search strategy**

| **Database** | **Hits** |
| --- | --- |
| Embase.com | 1,980 |
| Medline ovid | 2,358 |
| Web of science | 1,609 |
| PubMed | 940 |
| CNKI | 3,492 |
| Wanfang Data | 4,523 |
| VIP | 3,953 |
| **Total** | **18,855** |

**Embase.com**

**https://www.embase.com/#search**

('patient preference'/de OR 'patient decision making'/de OR 'help seeking behavior'/de OR 'patient satisfaction'/de OR 'patient attitude'/de OR 'attitude to health'/de OR ('decision making'/de AND ('patient'/de)) OR ((patient* NEAR/3 (preference* OR choice* OR choose OR satisf* OR seeking OR attitude* OR experience* OR decision* OR decide* OR priorit*)) OR barrier* OR facilitator* OR challenge* OR obstacle*):ab,ti) AND ('health care access'/de OR 'health care delivery'/de OR 'health care utilization'/de OR 'health care need'/de OR 'community care'/de OR 'primary health care'/exp OR 'secondary health care'/exp OR 'tertiary health care'/exp OR 'maternity ward'/de OR 'health center'/de OR 'health care facility'/de OR 'needs assessment'/de OR 'utilization review'/de OR 'community hospital'/de OR 'university hospital'/de OR 'general hospital'/de OR 'ambulatory care'/de OR 'dental clinic'/de OR 'emergency ward'/de OR ((access NEAR/3 point*) OR ((community OR home-based OR specialized*) NEAR/3 (care OR service* OR centre* OR center*)) OR (communit* NEAR/3 hospital*) OR ((care OR healthcare OR service* OR medical OR resource* OR hospital* OR health*) NEAR/3 (access* OR deliver* OR utili* OR underutili* OR overutili* OR use OR misuse OR demand* OR need OR needs )) OR ((primary OR secondary OR tertiary ) NEAR/3 (care OR healthcare OR physician* OR service*)) OR (source NEAR/3 (care* OR healthcare*)) OR (provider NEAR/3 care ) OR ((choice OR choose OR chose OR prefer* OR seeking) NEAR/6 (resource* OR hospital* OR doctor* OR service*)) OR (medical NEAR/3 consum*) OR ((township OR village) NEAR/3 (center* OR centre* OR clinic*)) OR ((maternity OR birthing) NEAR/3 (ward* OR centre* OR center*)) OR (health* NEAR/3 (centre* OR center* OR facilit*)) OR ((universit* OR academic* OR general) NEAR/3 (hospital* OR centre* OR center*)) OR (ambulator* NEAR/3 (care OR facilit*)) OR (dental NEAR/3 (clinic* OR facilit*)) OR gatekeep* OR (health NEAR/3 seeking ) OR (emergency NEAR/3 (ward* OR department*)) OR (General NEAR/3 (Practitioner* OR practice*)) OR (family NEAR/3 doctor*)):ab,ti) AND ('China'/exp OR (China OR (chinese NOT (chinese NEAR/3 (immigrant* OR migrant* OR american* OR canad OR austral*)))):ab,ti,ca,ta,cy,ad OR [chinese]/lim) NOT ([Conference Abstract]/lim OR [Letter]/lim OR [Note]/lim OR [Editorial]/lim)

**Medline ovid**

**http://ovidsp.tx.ovid.com/sp-3.24.1b/ovidweb.cgi**

("Help-Seeking Behavior"/ OR exp "patient satisfaction"/ OR "Attitude to Health"/ OR ("decision making"/ AND ("patients"/)) OR ((patient* ADJ3 (preference* OR choice* OR choose OR satisf* OR seeking OR attitude* OR experience* OR decision* OR decide* OR priorit*)) OR barrier* OR facilitator* OR challenge* OR obstacle*).ab,ti.) AND ("Health Services Accessibility"/ OR "Delivery of Health Care"/ OR "Needs Assessment"/ OR "Community Health Services"/ OR exp "Community Health Nursing"/ OR "Primary Health Care"/ OR "General Practitioners"/ OR exp "General Practice"/ OR "Secondary Care"/ OR "Secondary Care Centers"/ OR "Tertiary Healthcare"/ OR "Tertiary Care Centers"/ OR "Birthing Centers"/ OR "Health Facilities"/ OR "Utilization Review"/ OR "Hospitals, Community"/ OR "Hospitals, University"/ OR "Hospitals, General"/ OR "Ambulatory Care"/ OR "Ambulatory Care Facilities"/ OR "Dental Clinics"/ OR "Emergency Service, Hospital"/ OR ((access ADJ3 point*) OR ((community OR home-based OR specialized*) ADJ3 (care OR service* OR centre* OR center*)) OR (communit* ADJ3 hospital*) OR ((care OR healthcare OR service* OR medical OR resource* OR hospital* OR health*) ADJ3 (access* OR deliver* OR utili* OR underutili* OR overutili* OR "use" OR misuse OR demand* OR need OR needs )) OR ((primary OR secondary OR tertiary ) ADJ3 (care OR healthcare OR physician* OR service*)) OR (source ADJ3 (care* OR healthcare*)) OR (provider ADJ3 care ) OR ((choice OR choose OR chose OR prefer* OR seeking) ADJ6 (resource* OR hospital* OR doctor* OR service*)) OR (medical ADJ3 consum*) OR ((township OR village) ADJ3 (center* OR centre* OR clinic*)) OR ((maternity OR birthing) ADJ3 (ward* OR centre* OR center*)) OR (health* ADJ3 (centre* OR center* OR facilit*)) OR ((universit* OR academic* OR general) ADJ3 (hospital* OR centre* OR center*)) OR (ambulator* ADJ3 (care OR facilit*)) OR (dental ADJ3 (clinic* OR facilit*)) OR gatekeep* OR (health ADJ3 seeking ) OR (emergency ADJ3 (ward* OR department*)) OR (General ADJ3 (Practitioner* OR practice*)) OR (family ADJ3 doctor*)).ab,ti.) AND ("China"/ OR Beijing/ OR tibet/ OR (China OR (chinese NOT (chinese ADJ3 (immigrant* OR migrant* OR american* OR canad OR austral*)))).ab,ti,jn,cp,in OR chinese.la.) NOT (letter OR news OR comment OR editorial OR congresses OR abstracts).pt.

**Web of science**

**http://apps.webofknowledge.com/WOS_GeneralSearch_input.do?product=WOS&search_mode=GeneralSearch&SID=P2qQ7OGwgL7ItGxl1BY&preferencesSaved=**

TS=((((patient* NEAR/2 (preference* OR choice* OR choose OR satisf* OR seeking OR attitude* OR experience* OR decision* OR decide* OR priorit*)) OR barrier* OR facilitator* OR challenge* OR obstacle*)) AND (((access NEAR/2 point*) OR ((community OR home-based OR specialized*) NEAR/2 (care OR service* OR centre* OR center*)) OR (communit* NEAR/2 hospital*) OR ((care OR healthcare OR service* OR medical OR resource* OR hospital* OR health*) NEAR/2 (access* OR deliver* OR utili* OR underutili* OR overutili* OR use OR misuse OR demand* OR need OR needs )) OR ((primary OR secondary OR tertiary ) NEAR/2 (care OR healthcare OR physician* OR service*)) OR (source NEAR/2 (care* OR healthcare*)) OR (provider NEAR/2 care ) OR ((choice OR choose OR chose OR prefer* OR seeking) NEAR/5 (resource* OR hospital* OR doctor* OR service*)) OR (medical NEAR/2 consum*) OR ((township OR village) NEAR/2 (center* OR centre* OR clinic*)) OR ((maternity OR birthing) NEAR/2 (ward* OR centre* OR center*)) OR (health* NEAR/2 (centre* OR center* OR facilit*)) OR ((universit* OR academic* OR general) NEAR/2 (hospital* OR centre* OR center*)) OR (ambulator* NEAR/2 (care OR facilit*)) OR (dental NEAR/2 (clinic* OR facilit*)) OR gatekeep* OR (health NEAR/2 seeking ) OR (emergency NEAR/2 (ward* OR department*)) OR (General NEAR/2 (Practitioner* OR practice*)) OR (family NEAR/2 doctor*))) ) AND (TS=(China OR (chinese NOT (chinese NEAR/3 (immigrant* OR migrant* OR american* OR canad OR austral*)))) OR LA=chinese) AND DT=(article)

**PubMed**

**https://www.ncbi.nlm.nih.gov/pubmed/advanced**

((hospitals[mesh] OR tertiary healthcare[mesh] OR academic centers[mesh] OR secondary care[mesh] OR township health center[tw] OR village clinic[tw] OR ambulatory care facilities[mesh] OR ambulatory care[mesh] OR primary health care[mesh] OR birthing centers[mesh] OR dental facilities[mesh]) AND (needs assessment[mesh] OR utilization[mesh] OR choice behavior[mesh] OR personal satisfaction[mesh] OR utilization review[mesh] OR patient satisfaction[mesh] OR attitude to health[mesh])) OR gatekeeping[mesh] OR health services needs and demand[mesh] OR health services accessibility[mesh] OR health services/utilization[mesh] OR ambulatory care facilities/utilization[mesh] OR amubulatory care/utilization[mesh] OR primary health care/utilization[mesh] OR hospitals/utilization[mesh] OR tertiary healthcare/utilization[mesh] OR academic medical centers/utilization[mesh] OR secondary care/utilization[mesh] AND china[mesh]

**CNKI**

**http://kns.cnki.net/kns/brief/result.aspx?dbprefix=scdb&action=scdbsearch&db_opt=SCDB**

TI=(’医疗’+’初级保健’+‘初级卫生保健’+’医院’+’卫生服务’+’乡镇卫生中心’+‘中心卫生院’+’乡镇卫生院’+’村卫生室’ +’就诊’ +’就医’ +’住院’ +’门诊’ +’首诊’)*(’需求’+’利用’+’使用’+’可及性’+’选择’+’满意度’ +’流向’ +’意愿’)*(’患者’+’病人’+’居民’)

**Wanfang Data**

**http://www.wanfangdata.com/COJ/advanced_search.asp**

(题名:(医疗+初级保健+初级卫生保健+医院+卫生服务+乡镇卫生中心+中心卫生院+乡镇卫生院+村卫生室+就诊+就医+住院+门诊+首诊) AND 题名:(需求+利用+使用+可及性+选择+满意度+流向+意愿) AND 题名:(患者+病人+居民))

**VIP**

**http://lib.cqvip.com/ZK/index.aspx**

(T=医疗 OR T=初级保健 OR T=初级卫生保健 OR T=医院 OR T=卫生服务 OR T=乡镇卫生中心 OR T=中心卫生院 OR T=乡镇卫生院 OR T=村卫生室 OR T=就诊 OR T=就医 OR T=住院 OR T=门诊 OR T=首诊) AND (T=需求 OR T=利用 OR T=使用 OR T=可及性 OR T=选择 OR T=满意度 OR T=流向 OR T=意愿) AND (T=患者 OR T=病人 OR T=居民)

The Chinese search terms that were translated into English:

TI=(’health care’+’primary care’+‘primary health care’+’hospital’+’health service’+’township central health center’+‘central health center’+’township health center’+’village clinic’ +’hospital visit’ +’doctor visit’ +’inpatient’ +’outpatient’ +’first contact’)*(’demand’+’utilization’+’utilze’+’accessibility’+’choice’+’satisfaction’ +’flow’ +’willingness’)*(’patient’+’patients’+’residents’)
