# Supplementary material for: Factors influencing choice of health system access level in China: A systematic review
Source: PLoS One. 2018 Aug 10;13(8):e0201887. doi: 10.1371/journal.pone.0201887 (PMC6086423; doi:10.1371/journal.pone.0201887)
Supplement: S2 Text — (DOCX) [file pone.0201887.s002.docx]

**S2 Text**

**Detail description of identified factors influencing patient’s choice**

**Patient factors**

**Age**

Associations were frequently found between age and choice of health care facilities (n=18), however, different directions of the association were found among these studies. 11 studies [1-11] found the elderly is likely to use low level health care facilities, which include 2 cross-sectional studies that demonstrated older people were more inclined to sign family practice contracts [1,2]. 5 studies [12-16] found the opposite effect, namely that being older is a negative factor for choosing low level facilities. For example, in a sample of chronic disease patients, it was found that older people are more likely to choose general hospital [12]. In a cross-sectional study, the proportion of community health center (CHC) users at different age groups were compared with that in a National Health Service Survey (NHSS), and the results showed that the proportion of the elderly visitors was lower than that estimated by NHSS, which showed that CHC didn’t attract elderly visitors.

More generally, 2 studies concluded that populations from different age groups tend to use certain level of facilities [17,18]. A longitudinal study [17] investigated the effects of modifications on NCMS reimbursement level on outpatient service utilization for chronic patients in rural China. It found that the 10-17 years old group was 4.5% more likely to visit VC than the 18-30 years old group. In the other age groups, the likelihood of visiting VCs increased with age; for THC, compared with patients in the 18-30 years old group, those in the 46-60, 61-80, >80 age groups were 6.63%, 9.23% and 6.37%, respectively less likely to visit THC; but, the 10-17 years old group was 6.62% more likely to visit THC; and the other age groups didn't differ significantly; for county hospitals, compared with patients in the 18–30 age group, those in the 31–45,46-60, 61–80, and >80 years old groups were 3.57%, 7.90%, 10.74% and 9.91%, respectively, less likely to visit county-level hospitals; however, the other age groups did not differ significantly. Another study of patients with communicable diseases [18] found that the >26 year age group - especially 46-55 age group - were more likely to choose THCs over county hospitals.

**Health insurance status**

Significant associations between health insurance status and choice of health care facilities were found in 15 studies [4-9,13,16,19-25]. However, the effects differed substantially among the studies.

6 Studies [5-8,21,22] indicated that having health insurance is a significant positive factor for choosing lower level of facilities. For example, a study [21] found that people who had both outpatient and inpatient insurance coverage had significantly more village-level outpatient visits and township-level outpatient visits, compared to people who had inpatient insurance coverage only. Another study [22] found that lack of medical insurance was positively associated with using secondary care over primary care as usual source of care (USC).

4 Studies indicated that medical insurance was negatively associated to choosing lower level facilities [4,13,16,19]. For example, in a study [19] of the impact of rural health reform in Yunnan and Zhejiang province, patients had to go to township health center (THC) instead of VC even it was inconvenient, because cost at VC was not covered by NCMS. Beside, another 2 studies [24,25] reported that NCMS didn’t guide the patient flow down to lower level facilities, but increased the use of high level facilities, even with the increased reimbursement rate.

In particular, 3 studies [6,9,23] reported differences in impact between different types of insurance. In a study [6] found that patients with NCMS or URBMI were more willing to choose primary health care facilities, compared to patients without health insurance. In a sample of residents from urban areas in Guangdong [9], NCMS enrollees had the highest probability to choose CHC as a first contact among different types of medical insurance. In another study [23] of elderly residents in Beijing, UEBMI or URBMI enrollees were found to be more likely to choose community level facilities and tertiary hospitals for first contact, and NCMS enrollees were more likely to choose community level facilities and secondary hospitals for first contact. In addition, one study found patients with less knowledge about NCMS to be more likely to choose higher level facilities [7].

A study [20] of patients with major non-communicable chronic diseases reported the effect of medical insurance status among different patient groups. For urban patients, under coverage of medical insurance was one reason for initially choosing CHSs, while this was not the case for rural patients.

**Income**

Household income and individual income were repeatedly reported as a factor associated with choice of health care facilities (n=13; [5,7,9-11,14,15,18,20-22,26,27]. The majority of the studies (n=12) found the same direction of this association that income level is negatively associated with the likelihood of choosing low level facilities. One study [20] however found that low income rural patients are less likely to choose CHCs for initial treatment than their high income counterparts (OR=0.742).

**Education**

Education level was repeatedly found to be associated with patients’ choice of health care facilities in 11 studies [2,4,5,7,9-11,16,20,22,26]. All of those studies revealed the same effect of education level that people with higher education level are likely to choose primary facilities or sign contract with general practitioner (GP).

**Pre-existing disease**

8 Studies [1,7,13,16,22,26,28,29] showed the association of existing diseases with patient choice of health care facilities. In a sample of urban residents in Nanchong, Sichuan province [29], it was found that persons with more onsets of diseases in recent 3 months were more likely to choose CHC as first contact, than those who had less diseases onset in recent 3 months. All of the other 7 studies reported the findings related to patients’ existing chronic conditions. Among them, 5 [7,13,16,22,28] showed that chronic condition was negatively associated with using low level facilities. For example, a large cross-sectional survey [22] conducted in Guangdong demonstrated that people with multiple chronic conditions were more likely to choose secondary level facilities (instead of primary level facilities), compared to their counterparts without multi-morbidity. Another study [28] of a large sample of rural elderly in Guangdong found that healthy rural elderly preferred low level facilities (43.8%) more than their counterparts with chronic conditions (37.1%) when they felt unwell (p<0.01), and gave the conclusion that Health-Related Quality of Life (HRQL) and preferred health-seeking institutions among this elderly population were significantly correlated. Furthermore, in a nationwide survey [7] of 6024 rural residents, it was demonstrated that patients with onset of acute disease in the last 2 weeks were relatively more likely to choose low level. However, 2 study [1,26] found the opposite effect for patients suffering from chronic condition, i.e. found to them to less frequently choose CHCs or GP. Kuang et al. (2015) found that patients who choose to frequent Family Practitioners were more likely to have at least one chronic condition (p<0.01). Li et al. (2015) reported that one of the most frequently mentioned reasons for choosing CHC is that patients consider it not necessary to go to higher level hospital for minor diseases or (chronic) conditions.

**Disease severity**

7 studies [3,7,17,26,29-31] reported that patient’s choice were influenced by disease severity, and most of these studies showed that it was negatively associated with choosing low level facilities. For example, a nationwide study [7] investigating the impact of different reimbursement plans on patients’ choice of health care facilities showed that patients who evaluated their health condition as poor were more inclined to choose high level facilities. Similarly, in a longitudinal study of rural outpatients with type II diabetes in the Jiangsu province [17], the findings revealed that the coefficients for the effects of evaluated disease severities and visits to different types of facility were negative and statistically significant. Patients with medium severity and high severity were 12.49% and 32.35%, respectively, less likely to visit VCs than those in the low severity category; in line with this direction, compared to patients with low disease severity, patients with medium and high severity were 4.01% and 6.07% respectively, more likely to visit THCs, and were 5.11% and 19.22%, respectively, more likely to visit county-level hospitals. However, in a study [31] in Urumqi, Xinjiang autonomous region, big hospitals were the most frequent choice of urban residents for minor disease treatment (34.8%). This finding contrasts the result from the survey in the same study that most residents (91.8%) agreed with the principle to “treat minor disease in community; treat severe disease in hospital”.

**Gender**

Gender was also identified as a predisposing factor for the choice of health care facilities [8,17,18,32]. The 3 studies [17,18,32] showed that female patients were more likely to choose higher level facilities compared to their male counterparts. All of these 3 studies were conducted in rural area of Jiangsu [17] and Shandong [18,32] province. Contrary to this finding, however, 1 study [8] found the opposite effect that for patients with chronic conditions in urban Shenzhen, although gender was the least significant factor among age, insurance status, and chronic condition for urban patients with chronic diseases, female patients tend to seek help at CHC compared to male patients.

**Marriage status**

4 Studies [4,13,15,18] reported the associations between marital status and utilization of health care facilities. 2 of the 4 studies [13,15] indicated that being married negatively influences the utilization of low level facilities. Zeng et al. (2015) found that level 3 hospitals attracted more married people, compared to lower level facilities; another study demonstrated that [15] married people were more likely to choose level 3 hospitals. A study [4] done in Beijing showed that widowed people are 24.332 times more likely to choose facilities at level 2 or 3 than their unmarried counterparts. By contrast, a study on patients with communicable diseases [18] found that married people were more likely to choose THCs over county level facilities.

**Place of residence**

A study [27] reported that for first contact, rural residents in Chengdu were more likely to choose higher level facilities compared to urban residents. However, 2 studies [9,15] report the opposite. These 2 studies included samples from 25 provinces and samples from the Guangdong province. One study [20] of patients suffering from NCDs from the Guangdong province showed mixed effects of residing location. It found that in general, rural residents are more likely to seek treatment at level 3 hospitals, and to prefer level 2 hospitals over CHSs at the first sign of illness or for minor health concerns, with the exception of COPD patients. In rural areas, by contrast, patients with all types of NCDs were more likely to visit a CHS first.

Jiang et al. (2013) also found that for outpatient care, people in central (odds ratio 1.950) and western (odds ratio 1.641) regions were relatively more likely to access level 3 hospitals.

**Migrant**

3 Studies [1,13,33] regarded migrants. Two of these studies [1,33] reported the migrated population to be more likely to use low level facilities than local residents. The third study [13] compared the effect on different types of migrant in terms of their migration duration. It found that in, general, migrated population was more likely to choose level 3 hospitals. However, population who didn’t intent to migrate permanently, or with less than 5 year residency in Guangzhou tended to choose THCs or VCs over level 3 facilities.

**Occupation**

Occupation status was found in 3 studies [1,11,15], as being associated with facility choice. One study [1] found a higher proportion of retired patients among the patients with family practice contracts compared to the group without such contracts (59.1%

and 25.0%, respectively). Another study [15] found people working in big enterprises to be significantly more likely to visit level 3 hospitals (odds ratio 1.724) for outpatient treatment, compared to civil servants, while farmers were significantly less likely (odds ratio 0.458). Similar findings were reported by [11], which found that farmers or workers are more likely to go to CHCs.

**Health literacy**

Health literacy also influenced the choice of health care facilities. A study [20] of NCD patients conducted in Guangdong showed that for urban residents, obtaining health knowledge is negatively associated with choosing CHSs. Another study [10] investigated the impact of the zero-margin drug selling policy in Zhejiang. It found that even under the implementation of the zero-margin policy, 10.24% of the respondents continued to choose level 3 hospitals, out of habit.

**Ethnicity**

Only 1 study [31] identified ethnicity as an access level choice factor. The findings suggested that minorities tend to more frequently choose level 3 hospitals and western medicine for treatment of minor diseases, compared to Han Chinese.

**Life style**

Yang et al. (2014) found that urban residents doing physical exercise relatively less frequently choose CHSs;

**Anxiety before seeing doctor**

One study [34] found that people’s anxiety before seeing a doctor might influence their choice behavior. The survey conducted in 17 provinces showed that high anxiety increases the likelihood of choosing high level facilities. In fact, they were able to predict access level choice based on anxiety level.

**Provider factors**

**Drug**

13 Studies [6,10,18-20,28,31,32,35-39] mention drug availability at primary level facilities as a factor influencing patient choice. Limited drug variety was repeatedly mentioned as one reason for not choosing lower level facilities in 7 studies [19,20,28,35-38]. 3 Studies [19,35,36] especially report patients to choose county hospital or municipal/above level facilities over primary level facilities for this reason. Another 3 studies found that higher satisfaction with drug reimbursement rate, drug price, drug variety, and medication rationality [6,32], and more extensive essential medicine lists [18] increased willingness to choose CHC/THC level facilities.

8 Studies [6,10,18,19,31,37-39] especially investigated the implementation of EML. However, the direction of the associations found differed. Positive associations between EML implementation and directing patients to lower level facilities were reported found in five studies [6,10,18,31,38], among which 2 studies [10,31] reported that the main reason for choosing primary level facilities was the lower drug price. The other 3 studies indicated that higher total satisfaction with EML could generate more willingness to choose primary care facilities [6,10,18]. However, 5 studies [10,19,37-39] reported a negative association between EML implementation and choosing primary level facilities. These studies find that patients chose county level facilities, or above, because of the resulting limited drug variety at primary level facilities [19,37,38], and help-seeking habit of going to big hospitals [10].

**Medical equipment**

Medical equipment was repeatedly mentioned in 8 studies [5,9,20,28,32,35,36]+宁波. 6 Studies [9,20,28,35,36] +宁波report that poor quality/obsolete equipment is one of the reasons for not choosing VC/ but higher level facilities. Among them, 2 studies [35,36] reported that patients choose county hospital or municipal/above level facilities over low level facilities because of this reason. If equipment meets patient need, it can motivate patients to seek help from VCs for minor diseases [5]. Higher scores of satisfaction with equipment generates more willingness to choosing VC or THC [32] (14375).

**Service price/cost-effectiveness**

Low price is revealed as a strong reason for choosing CHCs by 6 studies [2,5,10,18,19,28] over higher level facilities. In a sample of residents in Zhejiang province [10], it was reported that average decrease of cost per visit positively impacted redirecting patients to CHCs. A study of rural residents in the Shandong province [10], found that the lower the price at THC in comparison to county level hospitals, the more likely patients were to choose THCs over county hospitals. Another study conducted in urban Shanghai [2] mentioned that good cost-effectiveness is one reason for accepting “first contact in community” (FCC). One study [19] conducted in Zhejiang and Yunnan rural area also found the trade-off between price and perceived quality of health care facilities by patients. It found that when there were only young medical graduates left at VC and THC, patients didn’t trust the medical skill and were more likely to choose the more expensive county hospitals.

Only one study +宁波 reported that the high service price was a reason for not choosing CHC.

**Service attitude**

6 Studies [2,9,20,28,32,40] address the service attitude as a patient choice factor. Three of them [2,28,40] report good service attitude as a reason to choose CHCs. 2 Studies reported that better service attitudes [20] or higher score of satisfaction with service attitude [32] are positively associated with choosing lower level facilities. Yao et al. (2014) report bad service attitudes as a reason to avoid CHCs and instead seek access at level 3 hospitals.

**Service scope**

Service scope was also identified in 3 studies [8,9,35] as a patient choice factor. Another study [8] found patients to be more willing to visit CHCs if the number of physicians specialized in chronic diseases was higher. Another two studies [9,35] reported limited variety of services and lack of health services other than for regular medical care as reasons for not choosing CHC.

**Physical environment of facility**

4 Studies [20,28,35] +宁波report lack of comfort as a reason for not choosing CHS or VC/THC level facilities but to seek access at higher level facilities instead.

**Medical staff**

3 Study [18,40] +宁波mentioned medical staff would be one factor that influenced patients’ choice. Xie et al. (2010) reported that the good relationship and personal connections with doctors was one of the reasons for choosing CHC as first contact place. Chen et al. (2013b) reported that high seniority of medical staff at THC increased the likelihood to choose access at level one rather than level two. However, Ma. et al. (2015) +宁波 reported that patients didn’t want to choose CHC because they were not acquainted with the doctors.

**Service convenience**

Convenience and short waiting time are mentioned as reasons for choosing CHS [28] or accepting FCC [2] over higher level facilities. However, another study [19] conducted in Zhejiang and Yunnan rural areas showed that patients who didn’t trust VCs/THCs chose county hospitals, despite them being much more expensive and crowded, and difficulties with admission and seeing doctors.

**Health Information Technology (HIT)**

A study [20] of patients with major non-communicable chronic diseases in the Guangdong province found that for urban residents, the awareness of taking community health report was positively associated to CHS visit. A study in Wenchuan county (Sichuan province) [41] reported HIT usage to increase referral and decrease the probability of seeking health care outside Wenchuan (i.e. promoted within-county care seeking).

**Context factors**

**Capitation/gatekeeping**

The influence of a reform of capitation as pre-payment method and the gatekeeping role of CHSC on patient flow was analyzed in a study [42] of rural residents in the Pudong area of Shanghai. It found that after the reform, outpatient visits at level 2 hospitals (THC/CH) reduced by 2.3%, and primary care (CHC/VC) use increased by 2.29%. There was no effect on level 3 hospitals. [40] explored the willingness of choosing CHCs as gatekeepers in Nanjing and reported that “the referral procedure at CHC was very inconvenient” as one of the reasons for not initially choosing CHCs.

**Freedom of service choice**

2 Studies [2,40] mentioned the preference for free choice of medical service facilities. Both of the studies were conducted in eastern urban areas and reported limitation of choice freedom as a reason not to sign contracts specifying family doctors at the CHC as gatekeepers.

**Salary reform on health workers**

A study [19] conducted in the rural areas of the Zhejiang and Yunnan provinces discovered that the salary reform indirectly impacted patient choice. The salary reform introduced fixed salaries for township health workers, to compensate for loss of income from drug sales because of the implementation of essential drug list. After the salary reform, the study found that patients more frequently selected the county hospitals instead of the THCs. Patient interviews provided as reasons that 1) doctors at THCs were not interested in treating patients anymore and transferred patients to county hospital, and 2) that the salary reform caused a brain drain of experienced health workers from township level and patients had little confidence in the remaining young medical graduates.

**Public campaign/interaction of social capital**

A study [2] conducted in the urban area of Shanghai explored the factors influencing patient contract choice with GP and found that exposure to GP system publicity campaigns increased the likelihood of signing a contract with GP, as well patient factors such as higher social interaction and social capital.

**Composite factors**

**Perceived quality of care**

16 Studies ([2,5-7,9,16,19,20,27,28,30,32,35,36,40]+宁波 found that the perceived quality or trust on medical skill in low level facilities were significant factor that influenced people’s choice. 13 studies([2,7,9,16,19,20,26,27,29,35,36,40] +宁波report perceived poor medical skills at primary level facilities as a reason for not choosing primary care facilities. For example, a study [19] conducted in the Zhejiang and Yunnan rural areas showed that when there were just a few young medical graduates left at VCs and THCs after the introduction of the essential medicine list, patients lacked trust in primary care and preferred service at expensive, crowded, county hospitals were it was difficult to see doctors and to get admitted. On the other hand, 1 study identified medical skill as a reason for choosing primary level facilities [5,30]. Another study [28] also reported medical skill both as a reason for CHCs and as a reason for not choosing them but to prefer higher level facilities. Another 2 studies [6,32] provide evidence that higher satisfaction with medical skills at low level facilities significantly increased the likelihood of seeking help at primary level facilities.

**Transportation convenience/distance**

The short distance between primary level facilities and home was repeatedly mentioned as a reason for choosing those facilities [7,20,28,31,40]. Furthermore, 2 studies [18,32] conducted in rural area of the Shandong province identified the relative distance to home of county hospitals and THCs as a factor influencing patient choice: if the THC is relatively closer to home (compared to the county hospital), patients were more likely to choose it.

[3] evaluated the effectiveness of urban health reform policies in Beijing and found that CHCs which had the highest increases in the number of visits were located more than 5km from densely populated areas and lacked convenient transportation, while the subset of CHCs with the largest decreases in number of visits mostly resided in densely populated areas close to the urban center with many level 3 hospitals.

**Reimbursement rate/coverage from insurance**

7 Studies present findings on the impact of reimbursement rates on patient choice [7,17,18,24,25,32,43]. One Study [32] conducted in rural area in Shandong province found relatively higher NCMS reimbursement rates as a reason to choose THCs over county hospitals. In another sample of rural residents in Shandong [18], patients were more likely to choose THCs when the difference in reimbursements rates were larger. Another nationwide study [7] analyzed the impact of NCMS on rural resident’s choice of facilities. It found that populations in areas with larger differences tended to more frequently choose lower level facilities and that the likelihood of choosing primary level facilities increased with increasing NCMS reimbursement rate at primary level.

However, one study [43] found that the effect of expanding reimbursement rate didn’t motivate patients to choose lower level facilities. The study was designed to assess the impact of a tiered reimbursement strategy that increased NCMS reimbursement rate at health care facilities, and especially implemented a more generous increase at lower level facilities. The findings suggest that the strategy at lower level facilities increased the likelihood of people using VC; however, the increase did not arise from a substitution away from higher level facilities, but an increase of using formal care. Similarly, in a sample of rural patients with type II diabetes mellitus in the Jiangsu province [17], when the reimbursement rate was increased for outpatient visit on different level facilities, patients were 5.99% more likely to visit THCs and 2.34% more likely to visit county level hospitals, compared to the control group which had no change on the reimbursement rate. The volume of outpatient visit at VCs had no significant change. Another 2 studies [24,25] also found that inpatients access to higher level facilities increased after the implementation of NCMS, even when lower level facilities were more generously reimbursed.

**Previous medical experience**

2 Studies [27,29] reported on the association between previous medical experience and choice of health care facilities. One study [29] found that urban residents with previous experience in community level health care facilities were more willing to choose community level facilities. In the other study [27], no inpatient experience was negatively associated with initial contact at community or township level facilities.

**Awareness about the facility**

Patient choice of health care facilities was also associated with patient knowledge of facilities and patient awareness of referral policies [27,40]. In a study of rural and urban residents in Chengdu, Sichuan province [27], residents that held little knowledge or awareness of community or township level facilities, were more likely to choose higher level facilities. In Nanjing, where the government promoted the first contact at CHC and implemented a gradient reimbursement policy, the urban residents who had higher awareness rate of the referral policy were more willing to visit community health center first, compared to their counterpart that had lower awareness rate (OR=0.598) [40].

**Disease diagnosis**

A study [36] of 3,500 rural patients in a city in the Shandong province reported that 42.74% of patients at the municipal hospital had the purpose of “confirmation of disease diagnosis”, which was significantly lower than the percentage (15.88%) at VC/THC.

**References**

(1) Kuang L, Liang Y, Mei J, Zhao J, Wang Y, Liang H, et al. Family practice and the quality of primary care: a study of Chinese patients in Guangdong Province. Fam Pract 2015 Oct;32(5):557-563.

(2) Jing L, Shu Z, Sun X, Chiu JF, Lou J, Xie C. Factors influencing patients' contract choice with general practitioners in Shanghai: a preliminary study. Asia-Pacific Journal of Public Health 2015 Mar;27(2 Suppl):77S-85S.

(3) Zhang X, Chen L, Mueller K, Yu Q, Liu J, Lin G. Tracking the effectiveness of health care reform in China: A case study of community health centers in a district of Beijing. Health Policy 2011 MAY 2011;100(2-3):181-188.

(4) Zhao Y, Zhang T. Analysis of Influence Factors of Rural Residents`Choice of Selecting Care Providers during Two －Week Prevalence in Tongzhou District of Beijing. Chinese Health Quality Management 2012(02):69-72.

(5) Guo Z, Xu L, Sun L, Liu T, Zhao W, Zhang Q, et al. Analysis on the Behavior of Rural Residents' Choice of Village Clinic in Shandong Province. Chinese Journal of Public Health 2012(05):690-692.

(6) Chen Y, Zhao C, Dai T. The impact to the satisfaction and health seeking intention of out-patients after implementing essential medicine system. Chinese Journal of Health Policy 2013(04):26-30.

(7) Huang X, Wang Q, Gu X, Xiang G, Li T, Mao Z. Research on Influence of New Rural Cooperative Medical Scheme Out-patient Reimbursement Plan on Choosing Medical Institutions. Chinese Health Economics 2012(04):48-51.

(8) Zhou H, Ye C, Zhu B, Wang R, Peng Z, Wang B, et al. Multilevel Model Analysis on Health Seeking Behavior of Patients with Chronic Diseases in Shenzhen City. Chinese Journal of Social Medicine 2011(04):249-251.

(9) Yao W, Lin Y, Zhong W, Wu M, Li J, Chen X. Investigation on the Willingness of Initial visit of Community Residents in Guangdong Province. Soft Science of Health 2014(09):602-606.

(10) Wang X, Zhang M, Hel L, Huang X, Gao J, Zhang L. Analysis of Urban and Rural Residents’ Desire and Hospitalizing Behavior on Drugs Zero -Profit Policy. Chinese Hospital Management 2012(05):10-13.

(11) Wei M, Xiao J. Study on influencing factors and countermeasures analyses of choosing a different medical institution by patients. Chinese Health Service Management 2014(04):259-261.

(12) Cheng SMSN, Zhao J, Bai JMSN, Zang X. Continuity of Care for Older Adults with Chronic Illness in China: An Exploratory Study. Public Health Nursing 2015 July/August;32(4):298-306.

(13) Zeng J, Shi L, Zou X, Chen W, Ling L. Rural-to-Urban Migrants' Experiences with Primary Care under Different Types of Medical Institutions in Guangzhou, China. PLoS ONE [Electronic Resource] 2015;10(10):e0140922.

(14) Dong X, Liu L, Cao S, Yang H, Song F, Yang C, et al. Focus on vulnerable populations and promoting equity in health service utilization--an analysis of visitor characteristics and service utilization of the Chinese community health service. BMC Public Health 2014 2014/;14:503.

(15) Jiang Y, Wang Y, Zhang L, Li Y, Wang X, Ma S. Access to healthcare and medical expenditure for the middle-aged and elderly: observations from China. PLoS One 2013 May 15;8(5):e64589.

(16) He H, Wang C, Zhen Q, Gu Y, Zhong L, Yu Y, et al. Community health service utilization among patients with chronic disease in Jilin province. Chinese Journal of Public Health 2014(10):1247-1249.

(17) Zhang L, Wang Z, Qian D, Ni J. Effects of changes in health insurance reimbursement level on outpatient service utilization of rural diabetics: evidence from Jiangsu Province, China. BMC Health Serv Res 2014 Apr 23;14:185-6963-14-185.

(18) Chen Q, Yin A, Han Z, Liang Z, Wei S. Study on Choice of medical institution of rural inpatients with distributable diseases in Shandong Province. Chinese Health Economics 2013(07):56-58.

(19) Zhou XD, Li L, Hesketh T. Health system reform in rural China: voices of healthworkers and service-users. Soc Sci Med 2014 Sep;117:134-141.

(20) Yang H, Huang X, Zhou Z, Wang HHX, Tong X, Wang Z, et al. Determinants of initial utilization of community healthcare services among patients with major non-communicable chronic diseases in South China. PLoS ONE 2014 2014/12;9(12).

(21) Wang H, Liu Y, Zhu Y, Xue L, Dale M, Sipsma H, et al. Health Insurance Benefit Design and Healthcare Utilization in Northern Rural China. Plos One 2012 NOV 21 2012;7(11):e50395.

(22) Wang HH, Wang JJ, Wong SY, Wong MC, Li FJ, Wang PX, et al. Epidemiology of multimorbidity in China and implications for the healthcare system: cross-sectional survey among 162,464 community household residents in southern China. BMC Med 2014 Oct 23;12:188-014-0188-0.

(23) Ji R, Zhu Z, Wang Y, Liu L, Sun G, Yang J. Demand and Utilization of Community Health Service for Middle and Old Age Residents Registered With Different Types of Medical Insurances in Beijing. Chinese General Practice 2015(16):1968-1971.

(24) Luo F, Yao L, Chen K, Liu Z, Wu S, Li Y, et al. Study on the distribution and medical expense of inpatients under the new medical reform: using a county of Hubei as case study. Chinese Health Economics 2015(02):60-62.

(25) Guo M, Wu Q, Li Y, Hao Y, Huang Z, Jiao M, et al. Analysis of the change tendency of inpatient flow and hospital cost burden among rural residents under new rural cooperative medical scheme. Chinese Hospital Management 2015(01):72-74.

(26) Li L, Mao J, Lin J, Weng Y, Li Y, Zhao J, et al. The Influencing Factors of Medical Care － seeking Intention and the Use of Community Health Service Among Residents in Guangzhou. Chinese General Practice 2015(01):100-104.

(27) Wang Z, Yang H, Gao B, Li N. Behavior of and satisfaction to first medical consultation among community ill residents in Chengdu city during a two-week period. Chinese Journal of Public Health 2014(11):1473-1476.

(28) Zhou Z, Wang C, Yang H, Wang X, Zheng C, Wang J. Health-related quality of life and preferred health-seeking institutions among rural elderly individuals with and without chronic conditions: A population-based study in Guangdong Province, China. BioMed Res Int 2014 /;2014.

(29) Xia X, Ren Y, Yang X, Zhang Y, Wei X. Study on initial diagnosis will and influencing factors in residents' community ——An example in Nanchong. Soft Science of Health 2015(10):619-622.

(30) Wang Y, Ma J, Huang A, Gong S. Investigation on Utilization of Community Health Service and Satisfactory Degree among Community Population in Jinan. Chinese Journal of Public Health Management 2011(04):374-375.

(31) Zhang X, Yao W, Zhao X, Wei H. Investigation of Community Residents' Intention of Visiting Doctor and Community Health Service in Urban Areas of Urumqi. Chinese General Practice 2014(13):1538-1542.

(32) Gong X, Cao X. Analysis of rural residents' willingness of getting care in township hospitals and its influence factors. Chinese Health Service Management 2011(11):854-855.

(33) Li H, Chung RY, Wei X, Mou J, Wong SY, Wong MC, et al. Comparison of perceived quality amongst migrant and local patients using primary health care delivered by community health centres in Shenzhen, China. Bmc Family Practice 2014 APR 29 2014;15:76.

(34) Tang L. The patient's anxiety before seeing a doctor and her/his hospital choice behavior in China. BMC Public Health 2012;12:1121.

(35) Bao L. Investigation on the Utilization of Rural Residents in the Background of the New Rural Cooperative Medical System in Ankang. Medicine and Society 2013(08):9-11.

(36) Jin Q, Han W, Zhang W. Medical demand of participants in new rural CMS and the relating factors. Chinese Rura l Health Service Administra tio n 2011(09):894-896.

(37) He P, Liu B, Sun Q, Zuo G, Li K. Comparative analysis on inpatient flow and medical expenditure of New Rural Cooperative Medical Scheme before and after the essential medicines system reform in Anhui province. Chinese Journal of Health Policy 2011(11):19-24.

(38) Zeng P, Que W, Li Y, Peng S, Ye D, Chen S. National Essential Drug System and Treatment Flow in Community Health Centers of Shajing Street. CHINESE PRIMARY HEALTH CARE 2012(04):26-27.

(39) Tian L, Zhao F, Yang H, Yang L, Yang L. The change tendency of visits among patients with new rural cooperative medical scheme after the implementation of essential medicines system in a city of Yunnan Province. Chinese Journal of Health Policy 2012(11):27-32.

(40) Xie Y, Dai T, Zhu K, Li C. Analysis of residents' willingness to select community doctor as gatekeeper and its determinants. Chinese General Practice 2010(15):1621-1624.

(41) Liu GG, Chen Y, Qin X. Transforming rural health care through information technology: an interventional study in China. Health Policy & Planning 2013 December;29(8):975-985.

(42) Jing L, Bai J, Sun X, Zakus D, Lou J, Li M, et al. NRCMS capitation reform and effect evaluation in Pudong New Area of Shanghai. International Journal of Health Planning & Management 2015;31:131.

(43) Powell-Jackson T, Yip WC, Han W. Realigning demand and supply side incentives to improve primary health care seeking in rural China. Health Econ 2015 Jun;24(6):755-772.
