# Supplementary material for: Factors influencing choice of health system access level in China: A systematic review
Source: PLoS One. 2018 Aug 10;13(8):e0201887. doi: 10.1371/journal.pone.0201887 (PMC6086423; doi:10.1371/journal.pone.0201887)
Supplement: S3 Text — (DOCX) [file pone.0201887.s003.docx]

**S3 Text**

**Background Information on the Chinese Health System**

This appendix describes the two main components of China’s healthcare delivery system, i.e. primary care and hospital care, as well as information on health insurance relevant to patient choice of health system access level. For each of these topics, we present a description of the relevant recent historical developments, and arrive at the current context.

China introduced a 3-tier hospital system in 1989 [1], where hospitals are defined as any ‘medical institution that has more than 20 beds [2]. The 3 tiers, or levels, refer to capacity and competency. Subsequently, each level differentiates 3 classes (A, B and C). Level 1, class C hospitals are rated as the lowest level. Together with an additional super class AAA at level 3, these levels and classes constitute the so-called Chinese “3 levels and 10 classes of hospital system” (as shown in Figure 1).

Hospitals at level 3 include municipal level, provincial level and state-owned hospitals. They provide comprehensive medical and disease prevention services. Moreover, they take the responsibility of medical education and research, and provide technical support to lower level facilities. Level 3 hospitals tend to be located in the bigger cities. Hospitals at level 2 include county level, regional level and some of the municipal level hospitals. They provide medical service and disease prevention service for a certain region. Similar to hospitals at level 3, hospitals at this level are also responsible for providing technical support to lower level facilities and undertaking limited educational tasks. Level 1 hospitals act as the first contact of medical care and disease prevention service for the community. Community health centers (CHCs) in urban areas and township health centers (THCs) in rural areas fall in this level. They are often not referred to as “hospitals” because they also provide basic public health service [2-4].

Rural China has adopted a specific 3-tiered hierarchy, known as the county health service network (see Figure 1). It constitutes of county hospitals, township health centers and village clinics (VC). The village clinics are the primary care facilities (see below). The objective of this network is to ensure access for the entire rural population and to achieve that “minor illness is treated in the village; common disease is treated in the township; serious illness is basically treated at the county level” [5,6].

The main carriers of primary health care service are the basic health care facilities, which include the CHCs and community health stations (CHSs) in urban areas, and THCs and VCs in rural areas [5,6], as shown in Figure 1. The current health reforms intend to develop strong networks between health care facilities at different levels, such as providing technical and management support to facilities at low level and implementing a two-way referral system. However, in the absence of gatekeeping mechanisms, patients can freely choose which facility to visit and often choose level 1 or level 2 hospitals. Moreover, the present fee-for-service models stimulate competition for patients across all levels. Lower level facilities can be reluctant to transfer patients to higher level facilities, and patient referral to lower level facilities by higher level hospitals is rare [7].

**Primary care in China**

There is no explicit definition of primary health care or primary care in China yet. From the development history and related policies, we adopt the distinction of two sets of services: public health service and primary care service [5,6]. Below, we disregard public health services, as it is beyond the scope of our review.

China introduced a primary health care system in 1968, and relied on ‘bare foot’ doctors to deliver it [8]. The bare foot doctors provided timely treatment to peasants, and were paid through the cooperative medical system, to which the peasants contributed. While the bare foot doctors had little access to technology or medication, the coverage of medical care in rural areas reached 84.6% in 1975 [9,10]. This system has been referred to as a successful practice by World Health Organization (WHO), for its access improvements, and has served as an example for other developing countries [8]. Following the 1978 societal and economic reforms, this primary care system has been slowly dismantled. The primary care health system shrunk, and coverage declined to only 39.9% in 1985 [9]. As of the early 1980s, the cooperative medical system collapsed [8,11]. As this eliminated the financial support for the bare foot doctors, their formal role in the health system ceased to exist [8,10,11].

In 1988 and 1991, the Chinese government committed to fulfilling the requirements of the Alma-Ata declaration [12] and to efforts towards “Health for all in 2000” [13]. Hence, a series of policies were introduced to improve the primary health care system. From 1999 to 2002, pilot projects were conducted to renew models of community health service in China, and subsequent implementation resulted in a triple increase of the number of community health facilities in 2008 [14].

Concurrently, new insurance schemes had been developed to resolve the difficulties of medical access in rural areas [15,16]. In 2009, China initiated a next round of health reform, which further prioritized primary care services [16-18]. In a subsequent reform round in 2013, the policy shifted towards “differentiating the primary and non-primary health care service” and “collaboration between primary and non-primary healthcare service” [19].

**Social health insurance system in China**

The social health insurance system in China is mainly composed of basic health insurance schemes, which includes Urban Resident Basic Medical Insurance (URBMI), Urban Employee Basic Medical Insurance (UEBMI) and NCMS [20,21]. In addition, there are public Medical Financial Assistance (MFA) for vulnerable people, and supplementary and commercial health insurance plans run by private sectors for specific or higher level health care demand [20,21]. The launch year and target population of these insurance schemes can be found in Figure 2.The coverage of those 3 main public health insurance schemes (URBMI, UEBMI and NCMS) is approximately 95% of the entire Chinese population [22]. Local agencies of public health insurance administration designate health care facilities in the insurance coverage network. The overall goals of current health reform include strengthening primary level facilities and building a hierarchical medical system [16,17]. As one of the measures, the reimbursement rate at primary level facilities are set to be higher than for higher level facilities [23,24]. In addition, most reimbursement policies focus on inpatient coverage rather than outpatients to achieve the goal of preventing catastrophic health expenditure [22,24,25].

**There is ongoing integration of NCMS and URBMI required by the State Council from January of 2016 in terms of beneficial package and management mechanism. The new name of the integrated insurance is Urban and Rural Residents Basic Medical Insurance [26]*.

|  | **UEBMI** | **URBMI** | **NCMS** | **MFA** | **supplementary and commercial health insurance plans** |
| --- | --- | --- | --- | --- | --- |
| **Launch year** | 1998 | 2007 | 2003 | 2003 | NA |
| **Target population** | Employed urban residents | Non-employed urban residents | Rural registered residents | Vulnerable people | People with specific or higher level health care demand |

Table 1. Social health insurance system in China

**References**

(1) Chinese Ministry of Health. Governing rules for the management and classification of hospitals. 1989.

(2) National Health and Family Planning Commission of the People's Republic of China. Basic standards for medical institutions (for trial). 1994; Available at: <http://www.nhfpc.gov.cn/yzygj/s3577/200804/13cac302fc0a422b80de18612f6d3d9a.shtml>. Accessed 31March, 2017.

(3) Tang L. The patient's anxiety before seeing a doctor and her/his hospital choice behavior in China. BMC Public Health 2012;12:1121.

(4) The State Council. Guidance on the development of urban communities for health services
. 2006; Available at: <http://www.gov.cn/zwgk/2006-02/23/content_208882.htm>. Accessed 24March, 2017.

(5) National Health and Family Planning Commission of the People's Republic of China. What are the functions of primary health service system? 2009; Available at: <http://www.nhfpc.gov.cn/tigs/s9664/200904/89079b7bf1134f72a604b51a982e9796.shtml>. Accessed 274March, 2017.

(6) National Health and Family Planning Commission of the People's Republic of China. What services can be provided by primary health care institutions? 2009; Available at: <http://www.nhfpc.gov.cn/tigs/s9664/200904/81df89f6981842dd9e47d2ff0951bac3.shtml>. Accessed 31March, 2017.

(7) Gong Y, Yin X, Wang Y, Li Y, Qin G, Liu L, et al. Social determinants of community health services utilization among the users in China: a 4-year cross-sectional study. PLoS One 2014 May 22;9(5):e98095.

(8) Zhang D, Unschuld PU. China's barefoot doctor: past, present, and future. Lancet 2008 Nov 29;372(9653):1865-1867.

(9) Zhu NS, Ling ZH, Shen J, Lane JM, Hu SL. Factors associated with the decline of the Cooperative Medical System and barefoot doctors in rural China. Bull World Health Organ 1989;67(4):431-441.

(10) Chen X, Hu T, Lin Z. The rise and decline of the cooperative medical system in rural China. International Journal of Health Services 1993;23(4):731-742.

(11) Feng X, Tang S, Bloom G, Segall M, Gu X. Cooperative medical schemes in contemporary rural China. Soc Sci Med 1995;41(8):1111-1118.

(12) International Conference on Primary Health Care. Declaration of Alma-Ata. 1978; Available at: <http://www.who.int/publications/almaata_declaration_en.pdf?ua=1>. Accessed March27, 2017.

(13) Ministry of Health, the People's Republic of China.  International Conference on Primary Health Care in Alma-Ata in 1978. 2002; Available at: <http://www.moh.gov.cn/zwgkzt/pncws1/200804/31122.shtml>. Accessed 24March, 2017.

(14) Bhattacharyya O, Yin D, Wong ST, Chen B. Evolution of primary care in China 1997–2009. Health Policy 2011;100(2):174-180.

(15) You X, Kobayashi Y. The new cooperative medical scheme in China. Health Policy 2009;91(1):1-9.

(16) Yip W, Hsiao W. China's health care reform: A tentative assessment. China economic review 2009;20(4):613-619.

(17) Liu Q, Wang B, Kong Y, Cheng KK. China's primary health-care reform. Lancet 2011 Jun 18;377(9783):2064-2066.

(18) Li H, Yu W. Enhancing community system in China's recent health reform: An effort to improve equity in essential health care. Health Policy 2011 Feb;99(2):167-173.

(19) The State Council of the People's Republic of China. Opinions on Promoting the Development of Health Service Industry. 2013; Available at: <http://www.gov.cn/xxgk/pub/govpublic/mrlm/201310/t20131018_66502.html>. Accessed 24March, 2017.

(20) Liu K. The Effects of Social Health Insurance Reform on People’s Out-of-Pocket Health Expenditure in China. : Springer; 2016.

(21) Barber SL, Yao L. Health insurance systems in China: a briefing note. World health report 2010.

(22) The State Council Information Office of the People's Republic of China. The Right to Development: China's Philosophy, Practice and Contribution. 2016; Available at: <http://www.gov.cn/zhengce/2016-12/01/content_5141177.htm>. Accessed 24March, 2016.

(23) Yu B, Meng Q, Collins C, Tolhurst R, Tang S, Yan F, et al. How does the New Cooperative Medical Scheme influence health service utilization? A study in two provinces in rural China. BMC health services research 2010;10(1):116.

(24) Meng Q, Xu L, Zhang Y, Qian J, Cai M, Xin Y, et al. Trends in access to health services and financial protection in china between 2003 and 2011: A cross-sectional study. The Lancet Mar 2012;379(9818):805-814.

(25) Meng Q, Fang H, Liu X, Yuan B, Xu J. Consolidating the social health insurance schemes in China: towards an equitable and efficient health system. The Lancet 2015;386(10002):1484-1492.

(26) National Health and Family Planning Commission of the People's Republic of China. Opinions of the State Council on Integrating the Basic Medical Insurance System for Urban and Rural Residents. 2016; Available at: <http://www.nhfpc.gov.cn/tigs/s3577/201601/dd4364b96d704fc2b72220ac5a9cb865.shtml>. Accessed March24, 2017.
