# Supplementary material for: Factors influencing choice of health system access level in China: A systematic review
Source: PLoS One. 2018 Aug 10;13(8):e0201887. doi: 10.1371/journal.pone.0201887 (PMC6086423; doi:10.1371/journal.pone.0201887)
Supplement: S1 Appendix — (PDF) [file pone.0201887.s005.pdf]

Systematic Reviews

CRD's guidance for undertaking reviews in health care

Centre for Reviews and Dissemination

Systematic Reviews

CRD's guidance for undertaking reviews in health care

© Centre for Reviews and Dissemination, University of York, 2008

Published by CRD, University of York

January 2009

ISBN 978-1-900640-47-3

This publication presents independent guidance produced by the Centre for Reviews and Dissemination (CRD). The views expressed in this publication are those of CRD and not necessarily those of the NHS, the NIHR or the Department of Health.

All rights reserved. Reproduction of this book by photocopying or electronic means for non-commercial purposes is permitted. Otherwise, no part of this book may be reproduced, adapted, stored in a retrieval system or transmitted by any means, electronic, mechanical, photocopying, or otherwise without the prior written permission of CRD.

Cover design by yo-yo.uk.com

Prepared and printed by:
York Publishing Services Ltd
64 Hallfield Road
Layerthorpe
York YO31 7ZQ
Tel: 01904 431213
Website: www.yps-publishing.co.uk

Contents

Preface		v
Acknowledgements		ix
Chapter 1	Core principles and methods for conducting a systematic review of health interventions	1
Chapter 2	Systematic reviews of clinical tests	109
Chapter 3	Systematic reviews of public health interventions	157
Chapter 4	Systematic reviews of adverse effects	177
Chapter 5	Systematic reviews of economic evaluations	199
Chapter 6	Incorporating qualitative evidence in or alongside effectiveness reviews	219
APPENDICES:		
Appendix 1	Other review approaches	239
Appendix 2	Example search strategy to identify studies from electronic databases	243
Appendix 3	Documenting the search process	249
Appendix 4	Searching for adverse effects	253
Abbreviations		255
Glossary		261
Index		277

PREFACE

This third edition of the Centre for Reviews and Dissemination (CRD) guidance for undertaking systematic reviews builds on previous editions published in 1996 and 2001. Our guidance continues to be recommended as a source of good practice by agencies such as the National Institute for Health Research Health Technology Assessment (NIHR HTA) programme, and the National Institute for Health and Clinical Excellence (NICE), and has been used widely both nationally and internationally. Our aim is to promote high standards in commissioning and conduct, by providing practical guidance for undertaking systematic reviews evaluating the effects of health interventions.

WHY SYSTEMATIC REVIEWS ARE NEEDED

Health care decisions for individual patients and for public policy should be informed by the best available research evidence. Practitioners and decision-makers are encouraged to make use of the latest research and information about best practice, and to ensure that decisions are demonstrably rooted in this knowledge.[1, 2] However, this can be difficult given the large amounts of information generated by individual studies which may be biased, methodologically flawed, time and context dependent, and can be misinterpreted and misrepresented.[3] Furthermore, individual studies can reach conflicting conclusions. This disparity may be because of biases or differences in the way the studies were designed or conducted, or simply due to the play of chance. In such situations, it is not always clear which results are the most reliable, or which should be used as the basis for practice and policy decisions.[4]

Systematic reviews aim to identify, evaluate and summarise the findings of all relevant individual studies, thereby making the available evidence more accessible to decision-makers. When appropriate, combining the results of several studies gives a more reliable and precise estimate of an intervention's effectiveness than one study alone.[5-8] Systematic reviews adhere to a strict scientific design based on explicit, pre-specified and reproducible methods. Because of this, when carried out well, they provide reliable estimates about the effects of interventions so that conclusions are defensible. As well as setting out what we know about a particular intervention, systematic reviews can also demonstrate where knowledge is lacking.[4, 9] This can then be used to guide future research.[10]

WHAT IS COVERED IN THE GUIDANCE

The methods and steps necessary to conduct a systematic review are presented in a core chapter (*Chapter 1*). Additional issues specific to reviews in more specialised topic areas, such as clinical tests (diagnostic, screening and prognostic), and public health are addressed in separate, complementary chapters (*Chapters 2-3*). We also consider questions relating to harm (*Chapter 4*) costs (*Chapter 5*) and how and why interventions work (*Chapter 6*).

This guide focuses on the methods relating to use of aggregate study level data. Although discussed briefly in relevant sections, individual patient data (IPD) meta-analysis, which is a specific method of systematic review, is not described in detail. The basic principles are outlined in *Appendix 1* and more detailed guidance can be found in the Cochrane Handbook[11] and specialist texts.[12, 13] Similarly, other forms of evidence synthesis including prospective meta-analysis, reviews of reviews, and scoping reviews are beyond the scope of this guidance but are described briefly in *Appendix 1*.

WHO SHOULD USE THIS GUIDE

The guidance has been written for those with an understanding of health research but who are new to systematic reviews; those with some experience but who want to learn more; and for commissioners. We hope that experienced systematic reviewers will also find this guidance of value; for example when planning a review in an area that is unfamiliar or with an expanded scope. This guidance might also be useful to those who need to evaluate the quality of systematic reviews, including, for example, anyone with responsibility for implementing systematic review findings.

Given the purpose of the guidance, the audience it is designed for, and the aim to remain concise, it has been necessary to strike a balance between the wide scope covered and the level of detail and discussion included. In addition to providing references to support statements and discussions, recommended reading of more specialist works such as the Cochrane Handbook,[14] Systematic Reviews in the Social Sciences,[4] and Systematic Reviews in Health Care[15] have been given throughout the text.

HOW TO USE THIS GUIDE

The core methods for carrying out any systematic review are given in *Chapter 1* which can be read from start to finish as an introduction to the review process, followed step by step while undertaking a review, or specific sections can be referred to individually. In view of this, and the sometimes iterative nature of the review process, occasional repetition and cross referencing between sections has been necessary.

Chapters 2-5 provide supplementary information relevant to conducting reviews in more specialised topic areas. To minimize repetition, they simply highlight the differences or additional considerations pertinent to their speciality and should be used in conjunction with the core principles set out in *Chapter 1*. *Chapter 6* provides guidance on the identification, assessment and synthesis of qualitative studies to help explain, interpret and implement the findings from effectiveness reviews. This reflects the growing recognition of the contribution that qualitative research can make to reviews of effectiveness.

For the purposes of space and readability:

The term 'review' is used throughout this guidance and should be taken as a short form for 'systematic review', except where it is explicitly stated that non-systematic reviews are being discussed.

'Review question' is used in the singular even though frequently there may be more than one question or objective set. The same process applies to each and every question.

A glossary of terms has been provided to ensure a clear understanding of the use of those terms in the context of this guidance and to facilitate ease of reference for the reader.

REFERENCES

1. Bullock H, Mountford J, Stanley R. *Better policy-making*. London: Centre for Management and Policy Studies; 2001.

2. Strategic Policy Making Team. *Professional policy making for the twenty first century*. London: Cabinet Office; 1999.

3. Wilson P, Petticrew M, and on behalf of the Medical Research Council's Population Health Sciences Research Network knowledge transfer project team. Why promote the findings of single research studies? *BMJ* 2008;336:722.

4. Petticrew M, Roberts H. *Systematic reviews in the social sciences: a practical guide*. Malden, MA: Blackwell Publishing; 2006.

5. Oxman AD. Meta-statistics: help or hindrance? *ACP J Club* 1993;118:A-13.

6. L'Abbe KA, Detsky AS, O'Rourke K. Meta-analysis in clinical research. *Ann Intern Med* 1987;107:224-33.

7. Thacker SB. Meta-analysis: a quantitative approach to research integration. *JAMA* 1988;259:1685-9.

8. Sacks HS, Berrier J, Reitman D, Acona-Berk VA, Chalmers TC. Meta-analysis of randomized controlled trials. *N Engl J Med* 1987;316:450-5.

9. Petticrew M. Why certain systematic reviews reach uncertain conclusions. *BMJ* 2003;326:756-8.

10. Brown P, Brunnhuber K, Chalkidou K, Chalmers I, Clarke C, Fenton M, et al. How to formulate research recommendations. *BMJ* 2006;333:804-6.

11. Stewart LA, Tierney JF, Clarke M. Chapter 19: Reviews of individual patient data. In: Higgins JPT, Green S, editors. *Cochrane handbook for systematic reviews of interventions. Version 5.0.0 (updated February 2008)*: The Cochrane Collaboration; 2008. Available from: www.cochrane-handbook.org.

12. Stewart LA, Clarke MJ. Practical methodology of meta-analyses (overviews) using updated individual patient data. Cochrane Working Group. *Stat Med* 1995;14:2057-79.

13. Stewart LA, Tierney JF. To IPD or not to IPD? Advantages and disadvantages of systematic reviews using individual patient data. *Eval Health Prof* 2002;25:76-97.

14. Higgins JPT, Green S, (editors). *Cochrane handbook for systematic reviews of interventions. Version 5.0.0 [updated February 2008]*: The Cochrane Collaboration; 2008. Available from: www.cochrane-handbook.org

15. Egger M, Davey Smith G, Altman DG. *Systematic reviews in health care: meta analysis in context*. 2nd ed. London: BMJ Books; 2001.

Acknowledgements

This guidance has been produced as a collaborative effort by the following staff at CRD, building on the work of the original writers of CRD Report 4 (Editions 1 & 2).

Jo Akers, Raquel Aguiar-Ibáñez, Ali Baba-Akbari Sari, Susanne Beynon, Alison Booth, Jane Burch, Duncan Chambers, Dawn Craig, Jane Dalton, Steven Duffy, Alison Eastwood, Debra Fayter, Tiago Fonseca, David Fox, Julie Glanville, Su Golder, Susanne Hempel, Kate Light, Catriona McDaid, Gill Norman, Colin Pierce, Bob Phillips, Stephen Rice, Amber Rithalia, Mark Rodgers, Frances Sharp, Amanda Sowden, Lesley Stewart, Christian Stock, Rebecca Trowman, Ros Wade, Marie Westwood, Paul Wilson, Nerys Woolacott, Gill Worthy and Kath Wright.

CRD would like to thank Doug Altman, Director of the Centre for Statistics in Medicine and Cancer Research UK Medical Statistics Group, at the University of Oxford, for writing the Prognostics section of *Chapter 2*, *Reviews of clinical tests*.

CRD is grateful to the following for their peer review comments on draft versions of various chapters of the guidance:

Doug Altman, Centre for Statistics in Medicine, University of Oxford.

Rebecca Armstrong, Melbourne School of Population Health, University of Melbourne.

Jeffrey Aronson, Department of Primary Health Care, University of Oxford.

Deborah Ashby, Barts and the London School of Medicine and Dentistry, Queen Mary, University of London.

Andrew Booth, Trent RDSU Information Service at ScHARR Library, University of Sheffield.

Patrick Bossuyt, AMC, University of Amsterdam.

Nicky Britten, Institute of Health Service Research, Peninsula Medical School, Universities of Exeter and Plymouth.

Mike Clarke, UK Cochrane Centre, Oxford.

Jon Deeks, Department of Public Health and Epidemiology, University of Birmingham.

Mary Dixon-Woods, Department of Health Sciences, University of Leicester.

Jodie Doyle, Melbourne School of Population Health, University of Melbourne.

Mike Drummond, Centre for Health Economics, University of York.

Zoe Garrett, Centre for Health Technology Evaluation, NICE.

Ruth Garside, Peninsula Medical School, Universities of Exeter and Plymouth.

Julie Hadley, Faculty of Health, Staffordshire University.

Roger Harbord, Department of Social Medicine, University of Bristol.

Angela Harden, EPPI-Centre, University of London.

Andrew Herxheimer, UK Cochrane Centre, Oxford.

Julian Higgins, MRC Biostatistics Unit, Institute of Public Health, Cambridge.

Chris Hyde, ARIF and West Midlands HTA Group, University of Birmingham.

Mike Kelly, Centre for Public Health Excellence, NICE.

Jos Kleijnen, Kleijnen Systematic Reviews.

Carole Lefebvre, UK Cochrane Centre, Oxford.

Yoon Loke, School of Medicine, Health Policy and Practice, University of East Anglia.

Susan Mallett, Centre for Statistics in Medicine, University of Oxford.

Heather McIntosh, Community Health Sciences, University of Edinburgh.

Miranda Mugford, School of Medicine Health Policy and Practice, University of East Anglia.

Mark Petticrew, Public and Environmental Health Research Unit, London School of Hygiene and Tropical Medicine.

Jennie Popay, Division of Health Research, Lancaster University.

Deidre Price, Department of Clinical Pharmacology, University of Oxford.

Gerry Richardson, Centre for Health Economics, University of York.

Jonathan Sterne, Department of Social Medicine, University of Bristol.

Jayne Tierney, MRC Clinical Trials Unit, London.

Luke Vale, Health Services Research Unit, University of Aberdeen.

Liz Waters, Melbourne School of Population Health, University of Melbourne.

Penny Whiting, Department of Social Medicine, University of Bristol.

CHAPTER 1

CORE PRINCIPLES AND METHODS FOR CONDUCTING A SYSTEMATIC REVIEW OF HEALTH INTERVENTIONS

1.1 GETTING STARTED **3**

1.1.1 Is a review required? **3**

1.1.2 The review team **4**

1.1.3 The advisory group **5**

1.2 THE REVIEW PROTOCOL **6**

1.2.1 Introduction **6**

1.2.2 Key areas to cover in a review protocol **6**

1.2.2.1 Background 6

1.2.2.2 Review question and inclusion criteria 6

1.2.2.3 Defining inclusion criteria 10

1.2.2.4 Identifying research evidence 13

1.2.2.5 Study selection 13

1.2.2.6 Data extraction 13

1.2.2.7 Quality assessment 14

1.2.2.8 Data synthesis 14

1.2.2.9 Dissemination 14

1.2.3 Approval of the draft protocol **14**

1.2.4 How to deal with protocol amendments during the review **15**

1.3 UNDERTAKING THE REVIEW **16**

1.3.1 Identifying research evidence for systematic reviews **16**

1.3.1.1 Minimizing publication and language biases 16

1.3.1.2 Searching electronic databases 17

1.3.1.3 Searching other sources 17

1.3.1.4 Constructing the search strategy for electronic databases 19

1.3.1.5 Text mining 20

1.3.1.6 Updating literature searches 20

1.3.1.7 Current awareness 20

1.3.1.8 Managing references 21

1.3.1.9 Obtaining documents 21

1.3.1.10 Documenting the search 21

1.3.2 Study selection **23**

1.3.2.1 Process for study selection 23

1.3.3 Data extraction **28**

1.3.3.1 Design 28

1.3.3.2 Content 28

1.3.3.3 Software 29

1.3.3.4 Piloting data extraction 29

1.3.3.5 Process of data extraction 29

1.3.4 Quality assessment **33**
1.3.4.1 Introduction 33
1.3.4.2 Defining quality 33
1.3.4.3 The impact of study quality on the estimate of effect 42
1.3.4.4 The process of quality assessment in systematic reviews 43
1.3.5 Data synthesis **45**
1.3.5.1 Narrative synthesis 48
1.3.5.2 Quantitative synthesis of comparative studies 54
1.3.6 Report writing **77**
1.3.6.1 General considerations 77
1.3.6.2 Executive summary or abstract 80
1.3.6.3 Formulating the discussion 81
1.3.6.4 Conclusions, implications, recommendations 81
1.3.7 Archiving the review **84**
1.3.8 Disseminating the findings of systematic reviews **85**
1.3.8.1 What is dissemination? 85
1.3.8.2 CRD approach to dissemination 86
REFERENCES **91**

1.1 GETTING STARTED

There are a number of reasons why a new review may be considered. Commissioned calls for evidence synthesis are usually on topics where a gap in knowledge has been identified, prioritised and a question posed. Alternatively the idea for a review may be investigator led, with a topic identified from an area of practice or research interest; such approaches may or may not be funded. Whatever the motivation for undertaking a review the preparation and conduct should be rigorous.

1.1.1 Is a review required?

Before undertaking a systematic review it is necessary to check whether there are already existing or ongoing reviews, and whether a new review is justified. This process should begin by searching the Database of Abstracts of Reviews of Effects (DARE),[1] and the Cochrane Database of Systematic Reviews (CDSR).[2] DARE contains critical appraisals of systematic reviews of the effects of health interventions. CDSR contains the full text of regularly updated systematic reviews of the effects of health care interventions carried out by the Cochrane Collaboration. Other sites to consider searching include, the National Institute for Health and Clinical Excellence (NICE) and the NIHR Health Technology Assessment (NIHR HTA) programme websites. The Campbell Collaboration website[3] contains the Campbell Library of Systematic Reviews giving full details of completed and ongoing systematic reviews in education, crime and justice, and social welfare; and the Evidence for Policy and Practice Information (EPPI) Centre,[4] whose review fields include education, health promotion, social care and welfare, and public health, has a database of systematic and non systematic reviews of public health interventions (DoPHER). It may also be worth looking at sites such as the National Guidelines Clearinghouse (NGC)[5] or the Scottish Intercollegiate Guidelines Network (SIGN),[6] as many guidelines are based on systematic review evidence. Searching the previous year of MEDLINE or other appropriate bibliographic databases may be helpful in identifying recently published reviews.

If an existing review is identified which addresses the question of interest, then the review should be assessed to determine whether it is of sufficient quality to guide policy and practice. In general, a good review should focus on a well-defined question and use appropriate methods. A comprehensive search should have been carried out, clear and appropriate criteria used to select or reject studies, and the process of assessing study quality, extracting and synthesising data should have been unbiased, reproducible and transparent. If these processes are not well-documented, confidence in results and inferences is weakened. The review should report the results of all included studies clearly, highlighting any similarities or differences between studies, and exploring the reasons for any variations.

Critical appraisal can be undertaken with the aid of a checklist[7-10] such as the example outlined in *Box 1.1*. Such checklists focus on identifying flaws in reviews that might bias the results.[8] Quality assessment is important because the effectiveness of interventions may be masked or exaggerated by reviews that are not rigorously conducted. Structured abstracts included in the DARE database[1] provide worked examples of how a checklist can be used to appraise and summarise reviews.

If a high quality review is located, but was completed some time ago, then an update of the review may be justified. Current relevance will need to be assessed and is particularly important in fields where the research is rapidly evolving. Where appropriate, collaboration with the original research team may assist in the update process by providing access to the data they used. However, little research has been conducted on when and how to update systematic reviews and the feasibility and efficiency of the identified approaches is uncertain.[11] If a review is of adequate quality and still relevant, there may be no need to undertake another systematic review.

Where a new systematic review or an update is required, the next step is to establish a review team and possibly an advisory group, to develop the review protocol.

Box 1.1: Critically appraising review articles

- Was the review question clearly defined in terms of population, interventions, comparators, outcomes and study designs (PICOS)?
- Was the search strategy adequate and appropriate? Were there any restrictions on language, publication status or publication date?
- Were preventative steps taken to minimize bias and errors in the study selection process?
- Were appropriate criteria used to assess the quality of the primary studies, and were preventative steps taken to minimize bias and errors in the quality assessment process?
- Were preventative steps taken to minimize bias and errors in the data extraction process?
- Were adequate details presented for each of the primary studies?
- Were appropriate methods used for data synthesis? Were differences between studies assessed? Were the studies pooled, and if so was it appropriate and meaningful to do so?
- Do the authors’ conclusions accurately reflect the evidence that was reviewed?

1.1.2 The review team

The review team will manage and conduct the review and should have a range of skills. Ideally these should include expertise in systematic review methods, information retrieval, the relevant clinical/topic area, statistics, health economics and/or qualitative research methods where appropriate. It is good practice to have a minimum of two researchers involved so that measures to minimize bias and error can be implemented at all stages of the review. Any conflicts of interest should be explicitly noted early in the process, and steps taken to ensure that these do not impact on the review process.

1.1.3 The advisory group

In addition to the team who will undertake the review there may be a number of individuals or groups who are consulted at various stages, including for example health care professionals, patient representatives, service users and experts in research methods. Some funding bodies require the establishment of an advisory group who will comment on the protocol and final report and provide input to ensure that the review has practical relevance to likely end users. Even if this is not the case, and even where the review team is knowledgeable about the area, it is still valuable to have an advisory group whose members can be consulted at key stages.

Engaging with stakeholders who are likely to be involved in implementing the recommendations of the review can help to ensure that the review is relevant to their needs. The particular form of user involvement will be determined by the purpose of the consultation. For example, when considering relevant outcomes for the review, users may suggest particular aspects of quality of life which it would be appropriate to assess. An example of a review which incorporated the views of users to considerable effect is one evaluating interventions to promote smoking cessation in pregnancy, which included outcomes more relevant to users as a result of their involvement.[12] However, consultation is time consuming, and needs to be taken into account in the project timetable. Where reviews have strict time constraints, wide consultation may not be possible.

At an early stage, members of the advisory group should discuss the audiences for whom the review findings are likely to be relevant, helping to start the planning of a dissemination strategy from the beginning of the project.

The review team may also wish to seek more informal advice from other clinical or methodological experts who are not members of the advisory group. Likewise, where an advisory group has not been established, the review team may still seek advice from relevant sources.

Summary: Getting started

- Whatever the motivation for undertaking a review the preparation and conduct should be rigorous.
- A search of resources such as the DARE database should be undertaken to check for existing or ongoing reviews, to ensure a new review is justified.
- A review team should be established to manage and conduct the review. The membership should provide a range of skills, including expertise in systematic review methods, information retrieval, the relevant clinical/topic area, statistics, health economics and/or qualitative research methods where appropriate.
- Formation of an advisory group including, for example, health care professionals, patient representatives, services users and experts in research methods may be a requirement of some funding bodies. In any event, it may be valuable to have an advisory group, whose members can be consulted at key stages.
- The review team may wish to seek advice from a variety of clinical or methodological experts, whether or not an advisory group is convened.

1.2 THE REVIEW PROTOCOL

1.2.1 Introduction

The review protocol sets out the methods to be used in the review. Decisions about the review question, inclusion criteria, search strategy, study selection, data extraction, quality assessment, data synthesis and plans for dissemination should be addressed. Specifying the methods in advance reduces the risk of introducing bias into the review. For example, clear inclusion criteria avoids selecting studies according to whether their results reflect a favoured conclusion.

If modifications to the protocol are required, these should be clearly documented and justified. Modifications may arise from a clearer understanding of the review question, and should not be made because of an awareness of the results of individual studies. Further information is given in *Section 1.2.4 How to deal with protocol amendments during the review*.

Protocol development is often an iterative process that requires communication within the review team and advisory group and sometimes with the funder.

1.2.2 Key areas to cover in a review protocol

This section covers the development of the protocol and the information it should contain. The formulation of the review objectives from the review question and the setting of inclusion criteria are covered in detail here as these must be agreed before starting a review. The search strategy, study selection, data extraction, quality assessment, synthesis and dissemination are also mentioned briefly as they are essential parts of the review protocol. However, to avoid repetition, full details of the issues related to both protocol requirements and carrying out the review are provided in *Section 1.3 Undertaking the review*.

1.2.2.1 Background

The background section should communicate the key contextual factors and conceptual issues relevant to the review question. It should explain why the review is required and provide the rationale underpinning the inclusion criteria and the focus of the review question, for example justifying the choice of interventions to be considered in the review.

1.2.2.2 Review question and inclusion criteria

Systematic reviews should set clear questions, the answers to which will provide meaningful information that can be used to guide decision-making. These should be stated clearly and precisely in the protocol. Questions may be extremely specific or very broad, although if broad, it may be more appropriate to break this down into a series of related more specific questions. For example a review to ‘assess the evidence on the positive and negative effects of population-wide drinking water fluoridation strategies to prevent caries’,[13] was undertaken by addressing five objectives:

Objective 1: What are the effects of fluoridation of drinking water supplies on the incidence of caries?

Objective 2: If water fluoridation is shown to have beneficial effects, what is the effect over and above that offered by the use of alternative interventions and strategies?

Objective 3: Does water fluoridation result in a reduction of caries across social groups and between geographical locations, bringing equity?

Objective 4: Does water fluoridation have negative effects?

Objective 5: Are there differences in the effects of natural and artificial water fluoridation?

Where there are several objectives it may be necessary to prioritise by importance and likelihood of being able to answer the question. It may even be necessary to restrict the scope of the question to a level that is manageable within set resources. For clarity, the singular term 'review question' is used throughout the guidance.

Box 1.2: Example review objective and PICOS elements for a review protocol

Review objective
The objective of this review is to assess the clinical effectiveness of treatments for childhood retinoblastoma.[14]

Participants
Studies of participants diagnosed with retinoblastoma at the age of 18 years or under.

Studies of adults where childhood retinoblastoma was followed up into adulthood.

Studies of mixed diagnoses if outcomes were reported separately for children with retinoblastoma.

Interventions
Any intervention or combination of interventions given for the treatment of retinoblastoma, including (but not restricted to) enucleation, external beam radiotherapy, chemotherapy, brachytherapy, cryotherapy, thermotherapy and photocoagulation.

Outcomes
Any clinical outcome, including (but not restricted to) survival, progression-free survival, tumour response, preservation of the eye, visual acuity, disease remission and adverse effects.

Study design
Randomised controlled trials (RCTs) and controlled trials. However, it is not anticipated that many studies of these designs will be available. Therefore, if information from controlled trials is not available, cohort studies are eligible for inclusion provided that data from a comparison group are reported.

Case series and case reports are excluded from the review owing to the high potential for bias in these study designs. Case–control studies (except where nested as part of a cohort study) and economic evaluations are also excluded.

**P**opulation<br>**I**nterventions<br>**C**omparators<br>**O**utcomes<br>**S**tudy design

The review question can be framed in terms of the population, intervention(s), comparator(s) and outcomes of the studies that will be included in the review. These elements of the review question, together with study design, will then be refined in order to determine the specific inclusion criteria that will be used when selecting studies for the review. Although both the acronyms PICO or PICOS are commonly used, here the term PICOS will be used throughout for consistency. In some situations, not all the elements will be relevant, for example not every review question will specify type of study design to be included. The use of PICOS in the context of reviews incorporating different study designs is discussed in the relevant chapters.

The review question may be presented in general terms, for example, 'What is the best treatment option for retinoblastoma?' More often the actual question is discussed by the review team and an objective, or series of objectives, framed by the population, the intervention and the outcome(s) of interest agreed. For example, 'The objective of this review is to assess the clinical effectiveness of treatments for childhood retinoblastoma.'[14] The PICOS elements for this example are shown in *Box 1.2*.

Population

The included population should be relevant to the population to which the review findings will be applied, and explicit inclusion criteria should be defined in terms of the disease or condition of interest. Any specified restrictions should be clinically justifiable and relevant. Eligibility must usually be applied to the whole study and consideration of how to deal with studies that include a mixed population, some of whom are relevant to the review and some of whom are not, is required. If the inclusion criteria are broad, it may be informative to investigate effectiveness across subgroups of participants. However, in the absence of individual patient data (IPD), or very detailed reporting of data, broken down by participant characteristics, it is unlikely that inclusion can be restricted to particular types of participant or that detailed subgroup analyses will be possible. Where analysis of participant subgroups is planned, this should be specified in the protocol. Examples of factors that may be investigated include participants' gender, age, disease severity, the presence of any co-morbidities, socio-economic status, ethnicity and geographical area.

Interventions and comparators

The nature of the interventions explored in the review may be framed in very broad terms like 'psychosocial interventions' or may be more specific such as 'cognitive behavioural therapy'. Factors usually specified include the precise nature of the intervention (e.g. the method of administration of a drug), the person delivering the intervention (e.g. a community psychiatric nurse versus a non-professional carer) or setting in which the intervention is delivered (e.g. inpatient or outpatient).

Where comparative studies are to be included, the protocol should also specify which comparators are eligible. As with the interventions, comparators should be carefully defined, so that the scope of a term such as 'palliative care' or 'usual care' is clear. The protocol should also specify whether any co-interventions carried out at the same time affect eligibility for inclusion; this applies to both the intervention(s) and the comparator(s).

Outcomes

The success or failure of a therapeutic intervention will usually be assessed in terms of differences in mortality or morbidity in the populations treated. Primary outcomes are likely to include measures of mortality and morbidity but other outcomes may also be of importance, for example measures of quality of life and participants' subjective experiences of pain or physical functioning.

A review should explore a clearly defined set of relevant outcomes and it is important to justify each outcome included. Input from the advisory group and the findings from initial scoping searches and qualitative research may be helpful in deciding which outcomes to include.

The use of surrogate outcomes may be misleading, giving an over or underestimate of the true clinical outcome.[15] Decisions about whether to consider surrogate outcomes should therefore be informed by available evidence about associations between the surrogate (e.g. blood pressure) and the outcome of interest (e.g. stroke). Often, surrogate outcomes are included only where a study also reports a relevant clinical outcome.

The review may also consider the timing of outcome assessment and possible adverse effects of the intervention. If the review is considering cost-effectiveness or economic issues as well as clinical effectiveness, the relevant economic outcomes should also be specified.

Although the review may aim to consider a series of outcomes, it is rare that inclusion would be restricted to only those studies that report all the outcomes of interest. More usually inclusion criteria will require that included studies report the main outcome.

Study design

The types of study included in the review will play a major role in determining the reliability of the results and the validity of estimates of effect is linked to the study design. While some study designs are clearly more robust than others, this should not be the only factor in determining which types of study are eligible for inclusion.[16]

Scoping searches may reveal that there are likely to be only a limited number of relevant randomised studies. In this case researchers have the option of justifying a decision to limit study design, bearing in mind that the identification of gaps in the current evidence base may in itself be a significant finding of the review. Alternatively, they can include quasi-experimental or observational studies. For reviews in some topic areas, these may be the only types of study available. The study design inclusion criteria given as an example in *Box 1.2* have been set to take account of the paucity of experimental studies, as indicated by the scoping searches.

In some cases a range of study designs may be needed to address different questions within the same review. For example, a review seeking to include information on adverse events will often include case-control and/or case-series (see *Chapter 4*) whilst a review incorporating participants' experiences of an intervention is likely to include qualitative studies (see *Chapter 6*). The potential biases from the inclusion of a range of study designs are discussed in *Section 1.3.4 Quality assessment.*

1.2.2.3 Defining inclusion criteria

The inclusion criteria should be set out in the protocol, to ensure that the boundaries of the review question are clearly defined. In the example in *Box 1.2*, the population to be studied was specified in the review question as those with 'childhood' retinoblastoma. In addition to qualifying 'childhood' as under 18, appropriate timeframes for disease progression and treatment and the possibilities of concurrent disease processes have been taken into account. In reviews of interventions relating to other diseases it may be necessary to be more specific about how the disease of interest will be verified, and to specify the disease stage and severity. In the simple example given in *Box 1.2* the key interventions and outcomes of interest are listed.

The nature of the intervention(s) and comparator(s) should be specified in detail. Whilst this may be more straightforward for drug interventions, more complex interventions may require detailed consideration of terms. For example, interventions such as 'stress management' or 'relaxation' may be defined differently by different study authors. Therefore researchers need to be clear about their own definitions and what elements are acceptable. An operational definition describing the content and delivery of the intervention will usually be helpful.

The inclusion criteria should capture all studies of interest. If the criteria are too narrowly defined there is a risk of missing potentially relevant studies and the generalisability of the results may be reduced. On the other hand, if the criteria are too broad the review may contain information which is hard to compare and synthesise.[17,18] Inclusion criteria also need to be practical to apply; if they are too detailed, screening may become overly complicated and time consuming.

Methodological quality

As previously stated, a review should be based on the best quality evidence available (see *Box 1.3*). Whatever the study design(s) included, it should not be assumed that all studies of the same basic design (e.g. RCT) are equally well-conducted. The quality of the included studies should be formally assessed as this will impact on the reliability of the results and therefore on the conclusions drawn. Although quality assessment can sometimes be used to exclude studies that do not meet certain criteria, this is not standard practice and differential quality is more usually assessed at the synthesis stage through sensitivity analysis. For further information see *Section 1.3.4 Quality assessment* and *Section 1.3.5 Data synthesis*.

Box 1.3: Hierarchy of study designs to assess the effects of interventions

This list is not exhaustive, but covers the main study designs. Refer to the glossary for definitions of other study designs. Names and definitions may differ (e.g. randomised controlled trial is often called randomised clinical trial).

(Continued)

Randomised controlled trials
The simplest form of RCT is known as the parallel group trial which randomises eligible participants to two or more groups, treats according to assignment, and compares the groups with respect to outcomes of interest. Participants are allocated to groups using both randomisation (allocation involves the play of chance) and concealment (ensures that the intervention that will be allocated cannot be known in advance). There are different types of randomised study designs, such as:

Randomised cross-over trials
Where all participants receive all the interventions; for example in a two arm cross-over trial, one group receives intervention A before intervention B, and the other group receive intervention B before intervention A. It is the sequence of interventions that is randomised.

Cluster randomised trials
A cluster randomised trial is a trial where clusters of people rather than single individuals are randomised to different interventions. For example, whole clinics or geographical locations may be randomised to receive particular interventions, rather than individuals.

Quasi-experimental studies
The main distinction between randomised and quasi-experimental studies is the way in which participants are allocated to the intervention and control groups; quasi-experimental studies do not use random assignment to create the comparison groups.

Non-randomised controlled studies
Individuals are allocated to a concurrent comparison group, using methods other than randomisation. The lack of concealed randomised allocation increases the risk of selection bias.

Before-and-after study
Comparison of outcomes in study participants before and after the introduction of an intervention. The before-and-after comparisons may be in the same sample of participants or in different samples.

Interrupted time series
Interrupted time series designs are multiple observations over time that are 'interrupted', usually by an intervention or treatment.

Observational studies
A study in which natural variation in interventions or exposure among participants (i.e. not allocated by an investigator) is investigated to explore the effect of the interventions or exposure on health outcomes.

Cohort study
A defined group of participants is followed over time and comparison is made between those who did and did not receive an intervention.

Case-control study
Groups from the same population with (cases) and without (controls) a specific outcome of interest, are compared to evaluate the association between exposure to an intervention and the outcome.

Case series
Description of a number of cases of an intervention and the outcome (without comparison with a control group). These are not comparative studies.

Language

The ideal for most systematic reviews is to include all available relevant evidence. In principle, this includes studies written in any language to avoid the introduction of language bias into the review. Language bias arises because studies with statistically significant results that have been conducted in non-English speaking countries may be more likely to be published in English language journals than those with nonsignificant results.[19] In addition, trials originating in certain countries have been found to have unusually high proportions of positive results.[20]

Thus, if reviews include only studies reported in English, their results and inferences may be biased.[19-21] Even if language bias does not influence summary effect estimates, it is likely to affect precision, because analysis will be based on fewer data.[22] Whenever feasible, all relevant studies should be included regardless of language. However, realistically this is not always possible due to a lack of time, resources and facilities for translation. It is advisable therefore, to identify all non-English language papers, document their existence, but record 'language' as the reason for exclusion in cases where they cannot be dealt with. Although titles and abstracts are translated in many databases, full papers are usually only available in their primary language.

When a decision is made to include non-English language studies, the review question should inform the decision about which languages are chosen, as studies of particular interventions and/or settings are more likely than others to be published in certain languages. An investigation of the inclusion of non-English language reports of RCTs in systematic reviews concluded that language restrictions do not appear to bias the estimates in reviews of conventional interventions, but may bias the results of complementary or alternative medicines.[23] Researchers need to give careful thought as to whether imposing language restrictions may potentially bias the results of their individual review. When non-English language literature is included in a review, its influence on the estimation and precision of effect may be explored in a sensitivity analysis.

Publication type/status

Studies are not always published as full papers in peer-reviewed journals; they may be published as reports, book chapters, conference abstracts, theses or they may be informally reported or remain unpublished. Ideally a review should aim to include all relevant studies, regardless of publication status, in order to avoid publication bias. Publication bias occurs when the publication of a study is influenced by its results, hence inclusion of only published studies may overestimate the intervention effect.[24]

There are practical issues that limit the inclusion of all studies regardless of publication type/status. Unpublished studies are likely to be harder to source, and more difficult to obtain, than published studies. The inclusion of conference abstracts and interim results should be considered, bearing in mind that contact with the study authors may be required to obtain full study details.[25] The effects of including any data from abstracts alone should be carefully considered, since differences often occur between data reported in conference abstracts and their corresponding full reports, although differences in results are seldom large.[26, 27] Also, it can be difficult to appraise study quality from minimal details provided in an abstract. Sensitivity analyses may be carried out to examine the effect of including data from conference abstracts.[28]

The identification of ongoing studies is important for a number of reasons. They may provide a useful starting point for subsequent reviews and updates; they may also improve the quality of conclusions about future research by indicating where new research has already commenced. Information about ongoing studies may be available as 'partially published research' like conference abstracts – these can be classified as ongoing studies which may contribute to future reviews.[29]

1.2.2.4 Identifying research evidence

A preliminary search strategy for identifying relevant research should be included in the protocol. This should specify the databases and additional sources that will be searched, and also the likely search terms to be used. The search strategy should be constructed to take into account PICOS, although the outcome(s) of studies and/or study design are not always used. Incorporating decisions about publication status and language restrictions also needs to be made at this stage. In reviews of one year or more duration, or reviews in rapidly evolving fields, provision for repeating the searches towards the end of the review process should also be considered. In addition it may be useful to carry out current awareness searches to identify relevant papers as they are published. The approach taken will depend on the question and the topic, and also on the available time and resources. It is usual to include in the protocol details of the software that will be used to manage references. Further information is given in *Section 1.3.1 Identifying research evidence for systematic reviews.*

1.2.2.5 Study selection

Study selection is usually conducted in two stages: an initial screening of titles and abstracts against the inclusion criteria to identify potentially relevant papers followed by screening of the full papers identified as possibly relevant in the initial screening. The protocol should specify the process by which decisions on the selection of studies will be made. This should include the number of researchers who will screen titles and abstracts and then full papers, and the method for resolving disagreements about study eligibility. *Section 1.3.2 Study selection* contains more information.

1.2.2.6 Data extraction

The protocol should outline the information that will be extracted from studies identified for inclusion in the review and provide details of any software to be used for recording the data. As with study selection the protocol should state the procedure for data extraction including the number of researchers who will extract the data and how discrepancies will be resolved. The protocol should also specify whether authors of primary studies will be contacted to provide missing or additional data. If foreign language papers are to be included, it may be necessary to specify translation arrangements. Further information is given in *Section 1.3.3 Data extraction.*

1.2.2.7 Quality assessment

The protocol should provide details of the method of study appraisal to be used, including examples of the specific quality criteria. Details of how the study appraisal is to be used should be specified, for example whether the results will inform sensitivity analyses. The protocol should also specify the process for conducting the appraisal of study quality, the number of researchers involved, and how disagreements will be resolved. For a detailed discussion of these issues see *Section 1.3.4 Quality assessment.*

1.2.2.8 Data synthesis

As far as possible, the protocol should specify the strategy for data synthesis. It should state whether a meta-analysis is planned, although whether a planned meta-analysis will ultimately prove possible will depend on the studies and data that are available. As analyses will depend on what data are available, and because it is difficult to anticipate all of the statistical issues that may arise, it can be difficult to pre-specify full details of the planned synthesis. However, the protocol should outline how heterogeneity will be explored and quantified, under what circumstances a meta-analysis would be considered appropriate and whether a fixed or random-effects model or both would be used. Where appropriate, the approach to narrative synthesis should also be outlined. The protocol should also specify the outcomes of interest and what effect measures will be used. Any planned subgroup or sensitivity analyses or investigation of publication bias should also be described. Further information is given in *Section 1.3.5 Data synthesis*.

1.2.2.9 Dissemination

Dissemination of findings is an integral part of the review process and fundamental to ensuring that the essential messages from the review reach the appropriate audiences. It is helpful to consider how the review findings will be disseminated from as early a stage as possible to allow adequate time for planning and development and to ensure that the proposed activities are properly resourced. Details are given in *Section 1.3.8 Disseminating the findings of systematic reviews*.

1.2.3 Approval of the draft protocol

Some commissioning or funding bodies may require that they formally approve the protocol, and will provide input to the draft protocol, in addition to the other stakeholders, such as clinical and methodological experts, patient groups and service users, who may be consulted. For commissioned reviews, even where it is not a specific requirement, it can be useful to communicate with the commissioner at the protocol development stage. This will help to ensure that the protocol meets the commissioning brief or where the review question or the scope of the project has been altered, that this is agreed before work commences.

1.2.4 How to deal with protocol amendments during the review

Sticking rigidly to a protocol when it becomes apparent that a change of direction is required, can result in a review that is not useful to end users. It is possible that consideration of the primary research may raise questions which were not anticipated at the protocol stage. Where this results from a clearer understanding of the review question, it can be appropriate to carry out documented and justified amendments to the protocol. In the report of the review findings it is helpful to distinguish between the initial review question and any subsequent amendments. It is never appropriate to modify the protocol because of awareness of the results of individual studies, as this is likely to introduce bias and affect the validity of the review's conclusions.

Many reviews undergo protocol modification.[30] Where modifications are a possibility, the implications for the review process and workload should be considered carefully. In particular, the likely impact on the literature search should be assessed, as it may require modification and running again. Data extraction forms may also need to be amended, and any data that have already been extracted might require some re-working. Protocol amendments should be documented in a protocol addendum and in the final report of the review.

Summary: The review protocol

- The protocol sets out in advance the methods to be used in the review with the aim of minimizing bias.
- The background section of the protocol should communicate the key contextual and conceptual factors relevant to the review question and provide the justification for the review.
- The protocol should specify the review question.
- Study inclusion and exclusion criteria should be clearly defined using the relevant PICOS elements.
- The protocol should also specify the methods which will be used to:
  - Identify research evidence
  - Select studies for inclusion
  - Data extract included studies
  - Quality assess included studies
  - Synthesise results
  - Disseminate the review findings
- In cases when it becomes apparent that a modification to the protocol is required, protocol amendments should be clearly documented and justified.

1.3 UNDERTAKING THE REVIEW

1.3.1 Identifying research evidence for systematic reviews

This section describes how to undertake a systematic search using a range of methods to identify studies, manage the references retrieved by the searches, obtain documents and write up the search process. Practical examples of constructing search strategies are given in *Appendix 2*, and *Appendix 3* provides examples of how the search should be documented. Issues around the identification of research evidence that are specific to review type such as adverse effects or clinical tests are discussed in the relevant chapters.

Conducting a thorough search to identify relevant studies is a key factor in minimizing bias in the review process. The search process should be as transparent as possible and documented in a way that enables it to be evaluated and reproduced.

Studies can be located using a combination of the following approaches:

- Searching electronic databases
- Visually scanning reference lists from relevant studies
- Handsearching key journals and conference proceedings
- Contacting study authors, experts, manufacturers, and other organisations
- Searching relevant Internet resources
- Citation searching
- Using a project Internet site to canvas for studies

1.3.1.1 Minimizing publication and language biases

Decisions about where and how to search could unintentionally introduce bias into the review, so the team needs to consider, and try to minimize, the possible impact of search limitations. For example, restricting the searching to the use of electronic databases, which consist mainly of references to published journal articles, could result in the review being subject to publication bias as this approach is unlikely to identify studies that have not been published in peer reviewed journals. Wider searching is needed to identify research results circulated as reports or discussion papers. The identification of grey literature, such as unpublished papers, is difficult, but some are included on databases such as NTIS (National Technical Information Service) and HMIC (Health Management Information Consortium). Libraries of specialist research organisations and professional societies may also provide access to collections of grey literature.

Searching databases and registers that include unpublished studies, such as records of ongoing research, conference proceedings and theses, can reduce the impact of publication bias. Conference proceedings provide information on both research in progress and completed research. Conference abstracts are recorded in some major bibliographic databases such as BIOSIS Previews, as well as in dedicated databases such as Index to Scientific and Technical proceedings, ZETOC, and the Conference Papers Index.[31-34] It is also worth consulting catalogues from major libraries, for

example the British Library and the US National Library of Medicine. The abstracts in conference proceedings may only give limited information, and there can be differences between data presented in an abstract and that included in a final report.[35, 36] For these reasons, researchers should try to acquire the full report, if there is one, before considering whether to include the results in a systematic review.

As already discussed, limiting searches to English language papers can introduce language bias. Large bibliographic databases, such as MEDLINE and EMBASE, do include a small number of non-English language journals.[37] Using additional databases such as LILACS (Latin American and Caribbean Health Sciences Literature) that contain collections of non-English language research can minimize potential language bias.

1.3.1.2 Searching electronic databases

The selection of electronic databases to search will depend upon the review topic. Lists of databases are available from libraries and from database providers, such as Dialog and Wolters Kluwer, while subject experts will be familiar with the bibliographic databases in their field.

For reviews of health care interventions, MEDLINE and EMBASE are the databases most commonly used to identify studies. The Cochrane Central Register of Controlled Trials (CENTRAL) includes details of published articles taken from bibliographic databases and other published and unpublished sources.[38] There are other databases with a narrower focus that could be equally appropriate. These include PsycINFO (psychology and psychiatry), AMED (complementary medicine), MANTIS (osteopathy and chiropractic) and CINAHL (nursing and allied health professions). If the topic includes social care there are a range of databases available including ASSIA (Applied Social Sciences Index and Abstracts), CSA Sociological Abstracts, and CSA Social Services Abstracts, that could be used. The databases referred to above are all subject-based but there are others, such as AgeInfo, Ageline and ChildData, that focus on a specific population group that could be relevant to the review topic.

Due to the diversity of questions addressed by systematic reviews, there can be no agreed standard for what constitutes an acceptable search in terms of the number of databases searched. For example, if the review is on a cross-cutting public health topic such as housing and health it is advisable to search a wider range of databases than if the review is of a pharmaceutical intervention for a known health condition (see *Chapter 3, Section 3.3 Identifying research evidence).*

1.3.1.3 Searching other sources

In addition to searching electronic databases, published and unpublished research may also be obtained by using one or more of the following methods.

Scanning reference lists of relevant studies
Browsing the reference lists of papers (both primary studies and reviews) that have been identified by the database searches may identify further studies of interest.

Handsearching key journals

Handsearching involves scanning the content of journals, conference proceedings and abstracts, page by page. It is an important way of identifying very recent publications that have not yet been included and indexed by electronic databases or of including articles from journals that are not indexed by electronic databases.[39] Handsearching can also ensure complete coverage of journal issues, including letters or commentaries, which may not be indexed by databases. It can also compensate for poor or inaccurate database indexing that can result in even the most carefully constructed strategy failing to identify relevant studies. Selecting which journals to handsearch can be done by analysing the results of the database searches to identify the journals that contain the largest number of relevant studies.

Searching trials registers

Trials can be identified by searching one or more of the many trials registers that exist. It can be a particularly useful approach to identifying unpublished or ongoing trials. Many of the registers are available on the Internet and some of the larger ones, such as www.ClinicalTrials.gov and www.who.int/trialsearch/, include the facility to search by drug name or by condition. While some registers are disease specific, others collect together trials from a specific country or region. Pharmaceutical companies may also make information about trials they have conducted available from their websites.

Contacting experts and manufacturers

Research groups and other experts as well as manufacturers may be useful sources of research not identified by the electronic searches, and may also be able to supply information about unpublished or ongoing research. Contacting relevant research centres or specialist libraries is another way of identifying potential studies. While these methods can all be useful, they are also time consuming and offer no guarantee of obtaining relevant information.

After a thorough and systematic search has been conducted, and relevant studies have been identified, topic experts can be asked to check the list to identify any known missing studies.

Searching relevant Internet resources

Internet searching can be a useful means of retrieving grey literature, such as unpublished papers, reports and conference abstracts. Identifying and scanning specific relevant websites will usually be more practical than using a general search engine such as ‘Google’.

Reviews of transport and ‘welfare to work’ programmes have reported how Internet searching of potentially relevant websites was effective in identifying additional studies to those retrieved from databases.[40, 41] It is worth considering using the Internet when investigating a topic area where it is likely that studies have been published informally rather than in a journal indexed in a bibliographic database.

Internet searching should be carried out in as structured a way as possible and the procedure documented (see *Appendix 3*).

Citation searching

Citation searching involves selecting a number of key papers already identified for inclusion in the review and then searching for articles that have cited these papers. This approach should identify a cluster of related, and therefore highly relevant, papers. As this is in effect a search forward through time, citation searching is not suitable for identifying recent papers as they cannot have been referenced by other older papers.

Citation searching used to be limited to using the indexes Science Citation Index Expanded, Social Sciences Citation Index, and Arts & Humanities Citation Index, but other resources (including CINAHL, PsycINFO and Google Scholar) now include cited references in their records so these are also available for citation searching. Using similar services offered by journals such as the BMJ can also be helpful.

Using a project Internet site to canvas for studies

Where it has been agreed that a dedicated website should be set up for the review, for example as part of the overall dissemination strategy, this can be used to canvas for unpublished data/grey literature. Inclusion of an email contact address allows interested parties to submit information about relevant research. Posting the inclusion and exclusion criteria on the website may help to ensure submissions are appropriate. Throughout the review process the website should be continually updated with information about the studies identified. Personal responses should be sent to all respondents and where appropriate submitted material should be included in the library of references. Further details about dedicated project websites can be found in *Section 1.3.8 Disseminating the findings of systematic reviews*.

This approach should probably only be considered for 'high profile' reviews and then it should be as an adjunct to active canvassing for unpublished/grey literature.

1.3.1.4 Constructing the search strategy for electronic databases

Search strategies are explicitly designed to be highly sensitive so as many potentially relevant studies as possible are retrieved. Consequently the searches tend to retrieve a large number of records that do not meet the inclusion criteria. While it is possible to increase the precision of a search strategy, and so reduce the number of irrelevant papers retrieved, this may lead to relevant studies being missed.[42]

Constructing an effective combination of search terms involves breaking down the review question into 'concepts'. Using the Population, Intervention, Comparator, and Outcomes elements from PICOS can help to structure the search, but it is not essential that every element is used. For example it may be better not to use terms for the outcomes since inclusion might mean that the database being searched fails to show relevant studies simply because the outcome is not mentioned prominently enough in the record, even though the study measured it. For each of the elements used, it is important to consider all the possible alternative terms. For example a drug intervention may be known by a generic name and one or more proprietary names. Advice should be sought from the topic experts on the review team and advisory group.

For a detailed discussion of how to structure a search from a review question, including the use of search filters for study design, see *Appendix 2*.

1.3.1.5 Text mining

Text mining is a rapidly developing approach to utilizing the large amount of published text now available. Its potential use in systematic reviews is currently being explored and it may in future be an additional useful way of identifying relevant studies.[43, 44] The aim of text mining is to identify connections between seemingly unrelated facts to generate new ideas or hypotheses. A number of processes are involved in the technique: a) Information Retrieval identifies documents to match a user's query; b) Natural Language Processing provides linguistic data needed to perform c) Information Extraction, the process of automatically obtaining structured data from an unstructured natural language document; and d) Data Mining, the process of identifying patterns in large sets of data.[45, 46] In future this approach may be helpful in automatically screening and ranking large numbers of potentially eligible studies prior to assessment by the researchers.

There are a variety of text mining tools available, for example TerMine and Acromine[47] are tools dealing with term extraction and variation. Also of interest are KLEIO,[48] which provides advanced searching facilities across MEDLINE and FACTA, which finds associated concepts using text analysis.[49] Further information about text mining and the use of these tools can be found on the National Centre for Text Mining website (www.nactem.ac.uk/).

1.3.1.6 Updating literature searches

Depending on the scope and timescale of the review, an update of the literature searches towards the end of the project may be required. If the initial searches were carried out some time before the final analysis is undertaken (e.g. six months) it may be necessary to re-run the searches to ensure that no recent papers are missed. To do this successfully the date the original search was conducted and the years covered by the search must have been recorded.

When doing update searches the update date field should be used rather than the actual date. This ensures that anything added to the database since the original search was conducted will be identified. If the database has added a lot of older material (e.g. from 1967) this will be removed by using the original date limits (e.g. 1990-2008) in combination with the update date field. For databases that do not include an update date field it may be better to run the whole search again and then use reference management software to remove those records that have already been identified and assessed.

1.3.1.7 Current awareness

If a review is covering an area where there is rapid change or if a major study is expected to report its findings in the near future, setting up current awareness alerts can ensure that new papers are identified as soon as they become available. Options for current awareness include e-mail alerts from journals and RSS feeds from databases or websites.

1.3.1.8 Managing references

To ensure the retrieved records are managed efficiently the team should agree working practices. For example, who will screen the references and record decisions about which documents to obtain and how to code these decisions; whether decisions about rejecting or obtaining documents should be made blind to others' decisions; and how to store documents received. In addition, one member of the team should be responsible for identifying and removing duplicate references, ordering inter-library loans, recording the receipt of documents, and following up non-arrivals.

Using bibliographic software such as EndNote, Reference Manager or ProCite to record and manage references will help in documenting the process, streamline document management and make the production of reference lists for reports and journal papers easier. EPPI-Reviewer, a web-based review management programme, also incorporates reference management functions.[4, 50] Alternatively it is possible to construct a database of references using a database package such as Microsoft Access or a word processing package. By creating a 'library' (database) of references, information can be shared by the whole review team, duplicated references can be identified and deleted more easily, and customised fields can be created where ordering decisions can be recorded.[42] Specialised bibliographic management software packages have the facility to import references from electronic databases into the library and interact with word processing packages so bibliographies can be created in a variety of styles.

When an electronic library of references is used, it is important to establish in advance clear rules about which team members can add or amend records in the library, and that consistent terminology is used to record decisions. It is usually preferable to have one person from the team responsible for the library of references.

1.3.1.9 Obtaining documents

Obtaining a large number of papers in a short space of time can be very labour intensive. The procedure for acquiring documents will vary according to organisational arrangements and will depend on issues such as cost, what resources are available, and whether access to an inter-library loan network is available. Most libraries in the United Kingdom will be able to obtain articles from the British Library Document Supply Centre's collection although membership is required and there is a charge per article. Many journals are available in full text on the Internet, although a subscription may be required before articles can be downloaded. It may be cost-effective to travel to a particular library to obtain material if a large number of references are required and are available. The information specialist on the team is likely to know about networks of associated libraries and electronic resources that can be used for obtaining documents.[51]

1.3.1.10 Documenting the search

The search process should be reported in sufficient detail so that it could be re-run at a later date. The easiest way to document the search is to record the process and the results contemporaneously. The decisions reached during development and any changes or amendments made should be recorded and explained. It is important to record all searches, including Internet searches, handsearching and contact with experts.

Providing the full detail of searches helps future researchers to re-run or update the searches and enables readers to evaluate the thoroughness of searching. The write up of the search should include information about the databases and interfaces searched (including the dates covered), full detailed search strategies (including any justifications for date or language restrictions) and the number of records retrieved.

When systematic reviews are reported in journal articles, limits on the word count may make it impossible to provide full details of the searches. In these circumstances as much information as possible should be provided within the available space. For example, 'We searched MEDLINE, EMBASE and CINAHL' is more helpful to the reader than 'We conducted computer searches'. Many journals now have an electronic version of the publication where the full search details can be provided. Alternatively, the published report can include the review team's contact details so full details of the search strategies can be requested. If a detailed report is being written for the commissioners of the review, the full search details should be included.

The use of flow charts to demonstrate how relevant papers are identified is detailed in *Section 1.3.2 Study selection*. Guidance on documenting the different aspects of the searching process is given in *Appendix 3*.

Summary: Identifying research evidence for systematic reviews

- The search for studies should be comprehensive.
- The extent of searching is determined by the research question and the resources available to the research team.
- Thorough searching is best achieved by using a variety of search methods (electronic and manual) and by searching multiple, possibly overlapping resources.
- Most of the searching is likely to take place at the beginning of the review with an update search towards the end.
- Using bibliographic software to record and manage references will help in documenting the process, streamline document management and make the production of reference lists for reports and journal papers easier.
- The search process should be documented in full or details provided of where the strategy can be obtained.

1.3.2 Study selection

Literature searching may result in a large number of potentially eligible records that need to be assessed for inclusion against predetermined criteria, only a small proportion of which may eventually be included in the review. The process for selecting studies should be explicit and conducted in such a way as to minimize the risk of errors and bias. This section explains the steps involved and the issues to be considered when planning and conducting study selection.

1.3.2.1 Process for study selection

The process by which decisions on the selection of studies will be made should be specified in the protocol, including who will carry out each stage and how it will be performed. The aim of selection is to ensure that all relevant studies are included in the review.

It is important that the selection process should minimize biases, which can occur when the decision to include or exclude certain studies may be affected by pre-formed opinions.[52-56] The process for study selection therefore needs to be explicit, objective and minimize the potential for errors of judgement. It should be documented clearly to ensure it is reproducible (see *Figure 1.1*). The selection of studies from electronic databases is usually conducted in two stages:

Stage 1: a first decision is made based on titles and, where available, abstracts. These should be assessed against the predetermined inclusion criteria. If it can be determined that an article does not meet the inclusion criteria then it can be rejected straightaway. It is important to err on the side of over-inclusion during this first stage. The review question and the subsequent specification of the inclusion and exclusion criteria are likely to determine ease of rejection in this first stage. Where the question and criteria are tightly focused then it is usually easier to be confident that the rejected studies are not relevant. Rejected citations fall into two main categories; those that are clearly not relevant and those that address the topic of interest but fail on one or more criteria such as population. For those in the first category it is usually adequate to record as an irrelevant study, without a reason why. For those in the second category it is useful to record why the study failed to meet the inclusion criteria, as this increases the transparency of the selection process. Where abstracts are available the amount and usefulness of the information to the decision-making process often varies according to database and journal. Structured abstracts such as those produced by the BMJ are particularly useful at this stage of the review process.

Stage 2: for studies that appear to meet the inclusion criteria, or in cases when a definite decision cannot be made based on the title and/or abstract alone, the full paper should be obtained for detailed assessment against the inclusion criteria.

Some searching methods provide access to full papers directly, for example handsearching journals and contact with research groups, in which case assessment for inclusion is a one stage process.

Even when explicit inclusion criteria are specified, decisions concerning the inclusion of individual studies can remain subjective. Familiarity with the topic area and an understanding of the definitions being used are usually important.

The reliability of the decision process is increased if all papers are independently assessed by more than one researcher, and the decisions shown to be reproducible. One study found that on average a single researcher is likely to miss 8% of eligible studies, whereas a pair of researchers working independently would capture all eligible studies.[57] Assessment of agreement is particularly important during the pilot phase (described later in this section), when evidence of poor agreement should lead to a revision of the selection criteria or an improvement of their coding. Agreement between assessors (inter-assessor reliability) may be formally assessed mathematically using a Kappa statistic (a measure of chance-corrected agreement).[58]

The process for resolving disagreements between assessors should be specified in the protocol. Many disagreements may be simple oversights, whilst others may be matters of interpretation. These disagreements should be discussed and, where possible, resolved by consensus after referring to the protocol; if necessary a third person may be consulted.

If resources and time allow, the lists of included and excluded studies may be discussed with the advisory group. In addition, these lists can be posted on a dedicated website with a request for feedback on any missing studies, an approach used in a review of water fluoridation.[59] For further information see *Section 1.3.8 Disseminating the findings of systematic reviews*.

Piloting the study selection process

The selection process should be piloted by applying the inclusion criteria to a sample of papers in order to check that they can be reliably interpreted and that they classify the studies appropriately. The pilot phase can be used to refine and clarify the inclusion criteria and ensure that the criteria can be applied consistently by more than one person. Piloting may also give an indication of the likely time needed for the full selection process.

Masking/blinding

Judgements about inclusion may be affected by knowledge of the authorship, institutions, journal titles and year of publication, or the results and conclusions of articles.[60] Blind assessment may be possible by removing such identifying information, but the gain should be offset against the time and effort required to disguise the source of each article. Several studies have found that masking author, institution, journal name and study results is of limited value in study selection.[61, 62] Therefore, the general opinion is that unmasked assessment by two independent researchers is acceptable.

Dealing with lack of information

Sometimes the amount of information reported about a study is insufficient to make a decision about inclusion, and it can be helpful to contact study authors to ask for more details. However, this requires time and resources, and the authors may not reply, particularly if the study is old. If authors are to be contacted it may be advisable to decide in advance how much time will be given to allow them to reply. If contacting authors is not practical then the studies in question could be excluded and listed as 'potentially relevant studies'. If a decision is made to include such studies, the influence on the results of the review can be checked in a sensitivity analysis.

Dealing with duplication

It is important to look for duplicate publications of research results to ensure they are not treated as separate studies in the review. Multiple papers may be published for a number of reasons including: translations; results at different follow-up periods or reporting of different outcomes. However, it is not always easy to identify duplicates as they are often covert (i.e. not cross referenced to one another) and neither authorship nor sample size are reliable criteria for identification of duplication.[63] Estimates of prevalence of duplicate publication range from 1.4% to 28%,[64] and studies have been found to have up to five duplicate reports.[63] Multiple reports from the same study may include identical samples with different outcomes reported or increasing samples with the same outcomes reported.

Multiple reporting can lead to biased results, as studies with significant results are more likely to be published or presented more frequently, leading to an overestimation of treatment effects when findings are combined.[65] When multiple reports of a study are identified these should be treated as a single study but reference made to all the publications. It may be worthwhile comparing multiple publications for any discrepancies, which could be highlighted and the study authors contacted for clarification.

Documenting decisions

It is important to have a record of decisions made for each article. This may be in paper form, attached to paper copies of the articles, or the selection process may be partially or wholly computerised. If the search results are provided in electronic format, they can be imported into a reference management program such as EndNote, Reference Manager or ProCite which stores, displays and enables organisation of the records, and allows basic inclusion decisions to be made and recorded (in custom fields). For more complex selection procedures, where several decisions and comments need to be recorded, a database program such as Microsoft Access may be of use. There are also programs specifically designed for carrying out systematic reviews which include aids for the selection process, such as TrialStat SRS and EPPI-Reviewer.

Reporting study selection

A flow chart showing the number of studies/papers remaining at each stage is a simple and useful way of documenting the study selection process. Recommendations for reporting and presentation of a flow chart when reporting systematic reviews with or without a meta-analysis have been developed by the PRISMA group, formerly the QUOROM group. Publication of these guidelines is forthcoming.[66, 67] In the meantime, the existing QUOROM guidelines for the reporting meta-analysis of RCTs,[9] provide guidance that is equally applicable to all systematic reviews. *Figure 1.1* is an example of a flow chart from a systematic review of treatments for childhood retinoblastoma.[14]

A list of studies excluded from the review should also be reported where possible, giving the reasons for exclusion. This list may be included in the report of the review as an appendix. In general, this list is most informative if it is restricted to 'near misses' (i.e. those studies that only narrowly failed to meet inclusion criteria and that readers might have expected to see included) rather than all the research evidence identified. Decisions to exclude studies may be reached at the title and abstract stage or at the full paper stage.

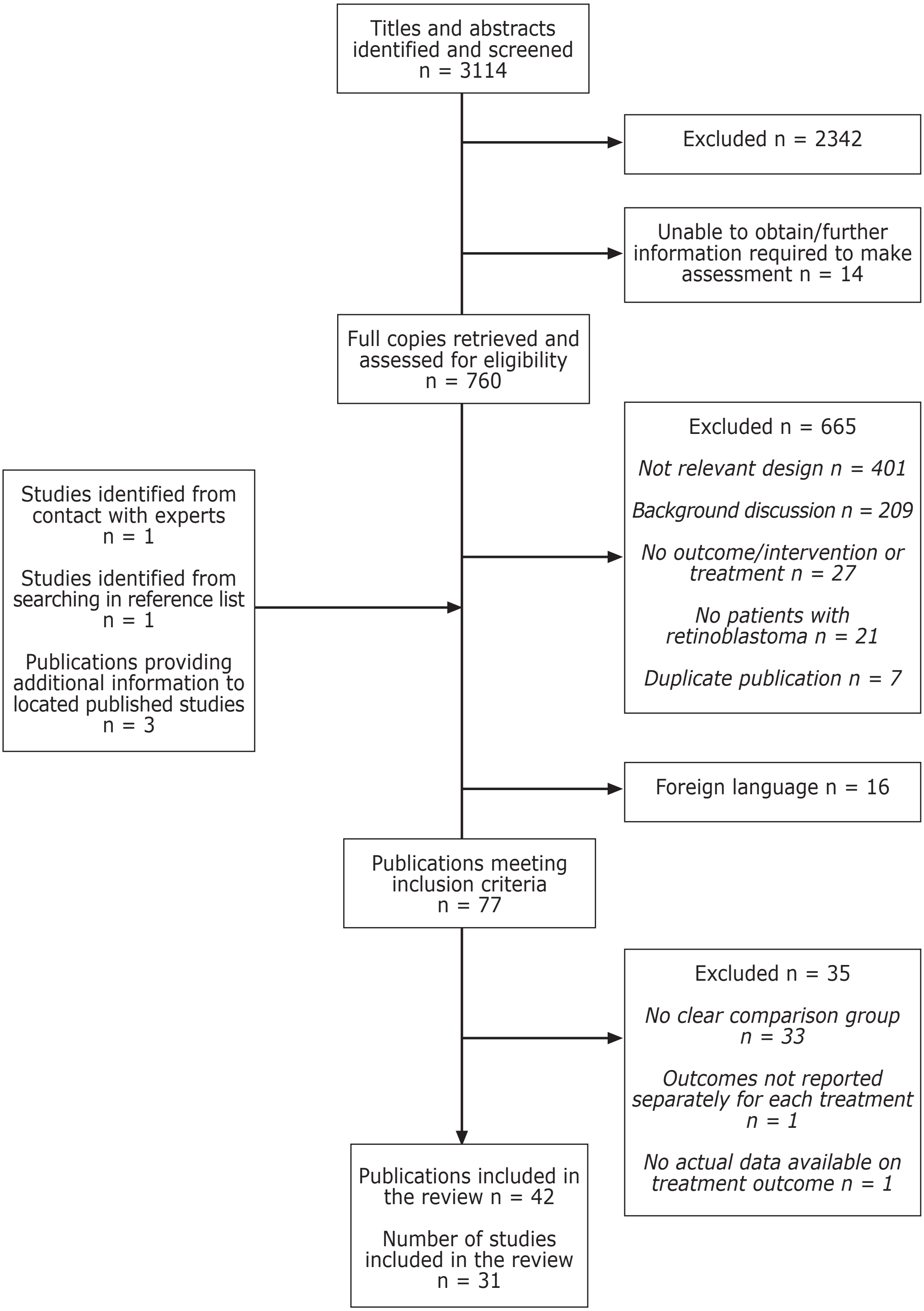

Figure 1.1: Flow chart of study selection process[14]

Summary: Study selection

- In order to minimize bias, studies should be assessed for inclusion using selection criteria that flow directly from the review question and that have been piloted to check that they can be reliably applied.
- Study selection is a staged process involving sifting through the citations located by the search, retrieving full reports of potentially relevant citations and, from their assessment, identifying those studies that fulfil the inclusion criteria.
- Parallel independent assessments should be conducted to minimize the risk of errors. If disagreements occur between assessors, they should be resolved according to a predefined strategy using consensus and arbitration as appropriate.
- The study selection process should be documented, detailing reasons for exclusion of studies that are 'near-misses'.

1.3.3 Data extraction

Data extraction is the process by which researchers obtain the necessary information about study characteristics and findings from the included studies. Data extraction requirements will vary from review to review, and the extraction forms should be tailored to the review question. The first stage of any data extraction is to plan the type of analyses and list the tables that will be included in the report. This will help to identify which data should be extracted. General guidance on the process is given here, but the specific details will clearly depend on the individual review topic.

A sample data extraction form and details of the data extraction process should be included in the review protocol. A common problem at the protocol stage is that there may be limited familiarity with the topic area. This can lead to uncertainties, for example, about comparators and outcome measures. As a result, time can be wasted extracting unnecessary data and difficulties can arise when attempting to utilise and synthesise the data. Sufficient time early in the project should therefore be allocated to developing, piloting and refining the data extraction form.

The extraction of data is linked to assessment of study quality in that both processes are often undertaken at the same time.

Standardised data extraction forms can provide consistency in a systematic review, whilst reducing bias and improving validity and reliability.[68] Use of an electronic form has the added advantage of being able to combine data extraction and data entry into one step, and to facilitate data analysis and the production of results tables for the final report.

1.3.3.1 Design

Integral to the design of the form is the category of data to be extracted. It may be numerical, fixed text such as yes/no, a 'pick list', or free text. However, the number of free text fields should be limited as much as possible to simplify the analysis of data. The form should be unambiguous and easy to use in order to minimize discrepancies. Instructions for completion should be provided and each field should have decision rules about coding data in order to avoid ambiguity and to aid consistent completion. Piloting the form is essential. Paper forms should only be used where access to direct completion of electronic forms is impossible, to reduce risks of error in data transcription.

1.3.3.2 Content

The nature of the data extracted will depend on the type of question being addressed and the types of study available. *Box 1.4* gives an example of some of the information that might be extracted for a comparative study.

The results to be extracted from each individual study may be reported in a variety of ways, and it is often necessary for a researcher to manipulate the available data into a common format. Manipulations of the reported findings are discussed in further detail in *Section 1.3.5 Data synthesis*, but can include using confidence intervals to

determine standard errors or estimating the hazard ratio from a survival curve. Data can be categorised at this stage; however, it is advisable to extract as much of the reported data as is likely to be needed, and categorise at a later stage, so that detailed information is not lost during data extraction.

1.3.3.3 Software

EPPI-Reviewer is a web application that enables researchers to manage all stages of a review in a single location. RevMan and TrialStat SRS are other software packages that can be used in data extraction for systematic reviews. Other tools commonly used include general word processing packages, spreadsheets and databases.

Whichever software package is used, ideally it should have the ability to provide different types of question coding. Some software will also allow researchers to develop quality control mechanisms for minimizing data entry errors, for example, by specifying ranges of valid values.

1.3.3.4 Piloting data extraction

Data extraction forms should be piloted on a sample of included studies to ensure that all the relevant information is captured and that resources are not wasted on extracting data not required. The consistency of the data extracted should be assessed to make sure that those extracting the data are interpreting the forms, and the draft instructions and decision rules about coding data, in the same way. This will help to reduce data extraction errors. The exporting, analysis and outputs of the data extraction forms should also be pilot tested where appropriate, on a small sample of included studies. This will ensure that the exporting of data works correctly and the outputs provide the information required for data analysis and synthesis.

When using databases, piloting is particularly important as it becomes increasingly difficult to make changes once the template has been created and information has been entered into the database. Early production of the expected output is also the best way to check that the correct data structure has been set up.

1.3.3.5 Process of data extraction

Data extraction needs to be as unbiased and reliable as possible, however it is prone to human error and often subjective decisions are required. The number of researchers that will perform data extraction is likely to be influenced by constraints on time and resources. Ideally two researchers should independently perform the data extraction (the level of inter-rater agreement is often measured using a Kappa statistic[58]). As an accepted minimum, one researcher can extract the data with a second researcher independently checking the data extraction forms for accuracy and completeness. This method may result in significantly more errors than two researchers independently performing data extraction but may also take significantly less time.[69] Any disagreements should be noted and resolved by consensus among researchers or by arbitration by an additional independent researcher. A record of corrections or

amendments to data extraction forms should be kept for future reference, particularly where there is genuine ambiguity (internal inconsistency) which cannot be resolved after discussion with the study authors. If using an electronic data extraction form that does not keep a record of amendments, completed forms can be printed and amendments recorded manually, before correcting the electronic version.

As with screening studies for inclusion, blinding researchers to the journal and author details has been recommended.[70, 71] However, this is a time-consuming operation, may not alter the results of a review and is likely to be of limited value.[61]

Box 1.4 Example information requirements for data extraction

General information

Researcher performing data extraction

Date of data extraction

Identification features of the study:
- Record number (to uniquely identify study)
- Author
- Article title
- Citation
- Type of publication (e.g. journal article, conference abstract)
- Country of origin
- Source of funding

Study characteristics

Aim/objectives of the study

Study design

Study inclusion and exclusion criteria

Recruitment procedures used (e.g. details of randomisation, blinding)

Unit of allocation (e.g. participant, GP practice, etc.)

Participant characteristics

Characteristics of participants at the beginning of the study e.g.
- Age
- Gender
- Ethnicity
- Socio-economic status
- Disease characteristics
- Co-morbidities

Number of participants in each characteristic category for intervention and control group(s) or mean/median characteristic values (record whether it is the number eligible, enrolled, or randomised that is reported in the study)

(Continued)

Intervention and setting
Setting in which the intervention is delivered

Description of the intervention(s) and control(s) (e.g. dose, route of administration, number of cycles, duration of cycle, care provider, how the intervention was developed, theoretical basis (where relevant))

Description of co-interventions

Outcome data/results
Unit of assessment/analysis

Statistical techniques used

For each pre-specified outcome:
- Whether reported
- Definition used in study
- Measurement tool or method used
- Unit of measurement (if appropriate)
- Length of follow-up, number and/or times of follow-up measurements

For all intervention group(s) and control group(s):
- Number of participants enrolled
- Number of participants included in analysis
- Number of withdrawals, exclusions, lost to follow-up
- Summary outcome data e.g.
  - Dichotomous: number of events, number of participants
  - Continuous: mean and standard deviation

Type of analysis used in study (e.g. intention to treat, per protocol)

Results of study analysis e.g.
- Dichotomous: odds ratio, risk ratio and confidence intervals, p-value
- Continuous: mean difference, confidence intervals

If subgroup analysis is planned the above information on outcome data or results will need to be extracted for each patient subgroup

Additional outcomes

Record details of any additional relevant outcomes reported

Costs

Resource use

Adverse events

NB: Notes fields can be useful for occasional pieces of additional information or important comments that do not easily fit into the format of other fields.

Reviews that include only published studies may be at risk of overestimating the treatment effect. Including data from unpublished studies (or unpublished outcomes) is therefore important in minimizing bias. However, this can be time-consuming and the original data may no longer be available. Although those performing IPD meta-analyses,[72] have generally been successful in obtaining data from the authors of unpublished studies, the same may not be true of other types of review. The practical difficulties of locating and obtaining information from unpublished studies may, for example, make the ideal of including relevant unpublished studies unachievable in the timescales available for many commissioned reviews. When information from unpublished studies is obtained, the published and unpublished material should be subjected to the same methodological evaluation.

Summary: Data extraction

- Standardised data extraction forms provide consistency in a systematic review, thereby potentially reducing bias, improving validity and reliability.
- Data extraction forms should be designed and developed with both the review question and subsequent analysis in mind. Sufficient time should be allocated early in the project for developing and piloting the data extraction forms.
- The data extraction forms should contain only information required for descriptive purposes or for analyses later in the systematic review. Information on study characteristics should be sufficiently detailed to allow readers to assess the applicability of the findings to their area of interest.
- Data extraction needs to be unbiased and reliable, however it is prone to human error and often subjective decisions are required. Clear instructions and decision rules about coding data should be used.
- As a minimum, one researcher should extract the data with a second researcher independently checking the data extraction forms for accuracy and detail. If disagreements occur between assessors, they should be resolved according to a predefined strategy using consensus and arbitration as appropriate.

1.3.4 Quality assessment

1.3.4.1 Introduction

Research can vary considerably in methodological rigour. Flaws in the design or conduct of a study can result in bias, and in some cases this can have as much influence on observed effects as that of treatment. Important intervention effects, or lack of effect, can therefore be obscured by bias.

Recording the strengths and weaknesses of included studies provides an indication of whether the results have been unduly influenced by aspects of study design or conduct (essentially the extent to which the study results can be 'believed'). Assessment of study quality gives an indication of the strength of evidence provided by the review and can also inform the standards required for future research. Ultimately, quality assessment helps answer the question of whether the studies are robust enough to guide treatment, prevention, diagnostic or policy decisions.

Many useful books discuss the sources of bias in different study designs in detail, or provide an in-depth guide to critical appraisal.[73-75] No single approach to assessing methodological quality is appropriate to all systematic reviews. The best approach will be determined by contextual, pragmatic and methodological considerations. However, the following sections describe the underlying principles of quality assessment and the key issues to consider.

1.3.4.2 Defining quality

Quality is a complex concept and the term is used in different ways. For example, a project using the Delphi consensus method with experts in the field of quality assessment of RCTs was unable to generate a definition of quality acceptable to all participants.[76]

Taking a broad view, the aim of assessing study quality is to establish how near the 'truth' its findings are likely to be and whether the findings are of relevance in the particular setting or patient group of interest. Quality assessment of any study is likely to consider the following:

- Appropriateness of study design to the research objective
- Risk of bias
- Other issues related to study quality
  - Choice of outcome measure
  - Statistical issues
  - Quality of reporting
  - Quality of the intervention
  - Generalisability

The importance of each of these aspects of quality will depend on the focus and nature of the review. For example, issues around statistical analysis are less important if the study data are to be re-analysed in a meta-analysis, and the quality of reporting is irrelevant where data (either individual patient or aggregate) and information are obtained directly from those responsible for the study.

Appropriateness of study design

As discussed previously, types of study used to assess the effects of interventions can be arranged into a hierarchy, based broadly on their susceptibility to bias (*Box 1.3*). Although the RCT is considered the best study design to evaluate the effect of an intervention, in cases where it is unworkable or unethical to randomise participants (e.g. when evaluating the effects of smoking on health), researchers may instead have to use a quasi-experimental or an observational design. Simply grading studies using this hierarchy does not provide an adequate assessment of study quality, because it does not take into account variations in quality among studies of the same design. Even RCTs can be implemented in such a way that findings are likely to be seriously biased and therefore of little value in decision-making.

It should be noted that the terminology used to describe study designs (e.g. cohort, prospective, retrospective, historical controls, etc.) can be ambiguous and used in different ways by different researchers. Therefore it is important to consider the individual aspects of the study design that may introduce bias rather than focussing on the descriptive label used. This is particularly important for the description of non-randomised studies.

Risk of bias

Bias refers to systematic deviations from the true underlying effect brought about by poor study design or conduct in the collection, analysis, interpretation, publication or review of data. Bias can easily obscure intervention effects, and differences in the risk of bias between studies can help explain differences in findings.

Internal validity is the extent to which an observed effect can be truly attributed to the intervention being evaluated, rather than to flaws in the design or conduct of the study. Any such flaws can increase the risk of bias.

The types of bias, and the ways in which they can be minimized by each type of study design, are described below.

Randomised controlled trials

The RCT is generally considered to be the most appropriate study design for evaluating the effects of an intervention. This is because, when properly conducted, it limits the risk of bias. The simplest form of RCT is known as the parallel group trial which randomises eligible participants to two or more groups, treats according to assignment, and compares the groups with respect to outcomes of interest.

Participants are allocated to groups using both randomisation (allocation involves the play of chance) and concealment (ensures that the intervention that will be allocated cannot be known in advance of assignment). When appropriately implemented, these aspects of design should ensure that the groups being compared are similar in all respects other than the intervention. The groups should be balanced for both known and unknown factors that might influence outcome, such that any observed differences should be attributable to the effect of the intervention rather than to intrinsic differences between the groups.

Allocation in this way avoids the influence of confounding, where an additional factor is associated both with receiving the intervention and with the outcome of interest. For example, babies who are breast fed are less likely to have gastrointestinal illnesses than those who are bottle fed. Though this might suggest evidence for the protective effect of breastfeeding, mothers who breast feed also tend to be of higher socio-economic status, which in itself is associated with a range of health benefits to the baby. Therefore, when evaluating any possible protective effects of breastfeeding socio-economic status should be considered as a potential confounding factor. In some cases, the possible confounding factor(s) may not be known or measurable. In an RCT, so long as a sufficient number of participants are assigned then the groups should be balanced with respect to both known and unknown potential confounding factors.

Selection bias or allocation bias occurs where there are systematic differences between comparison groups in terms of prognosis or responsiveness to treatment. Concealed assignment prevents investigators being able to predict which intervention will be allocated next and using that information to select which participant receives which treatment. For example, clinicians may want to 'try out' the new intervention in patients with a poorer prognosis. If they succeed in doing this by knowing or correctly 'guessing' the order of allocation, the intervention group will eventually contain more seriously ill participants than the comparison group, such that the intervention will probably appear less effective than if the two groups had been properly balanced.

The most robust method for concealing the sequence of treatment allocation is a central telephone randomisation service, in which the care provider calls an independent trial service, registers the participant's details and then discovers which intervention they are to be given. Similarly, an on-site computer-based randomisation system that is not readable until the time of allocation might be used. Envelope methods of randomisation, where allocation details are stored in pre-prepared envelopes, are less robust and more easily subverted than centralised methods. Where this method is adopted, sealed opaque sequentially numbered envelopes that are only opened in front of the participant being randomised should be used. Unfortunately, the methods which are used to ensure that the randomisation sequence remains concealed during implementation (frequently referred to as concealment of allocation) are often poorly reported making it difficult to discern whether the methods were susceptible to bias.

Some studies, which may describe themselves as randomised, may allocate participants to groups on an alternating basis, or based on whether their date of birth is an odd or even number. Allocation in these studies is neither random nor concealed.

Performance bias refers to systematic differences (apart from the intervention of interest) in the treatment or care given to comparison groups during the study and detection bias refers to systematic differences between groups in the way that outcomes are ascertained. The risk of these biases can be minimized by ensuring that people involved in the study are unaware of which groups participants have been assigned to (i.e. they are blinded or masked). Ideally, the participants, those administering the intervention, those assessing outcomes and those analysing the data should all be blinded. If not, the knowledge of which comparison group is which may consciously or unconsciously influence the behaviour of any of these people. The feasibility and/ or success of blinding will partly depend on the intervention in question. There are situations where blinding is not possible owing to the nature of the intervention, for

example where a particular intervention has an obvious physiological effect whereas the comparator does not, and others where it may be unethical (e.g. sham surgery carries risks with no intended benefit). Methods of blinding for studies of drugs involve the use of pills and containers of identical size, shape and number (placebos). Sham devices can be used for many device interventions and for some procedural interventions sham procedures can be used (e.g. sham acupuncture). Blinding of outcome assessors is particularly important for more subjective outcome measures such as pain, but less important for objective measures such as mortality. Implementation of a blinding process does not however guarantee successful blinding in practice. In study reports, terms such as double-blind, triple-blind or single-blind can be used inconsistently[77] and explicit reporting of blinding is often missing.[78] It is important to clarify the exact details of the blinding process.

A well-conducted RCT should have processes in place to achieve complete and good quality data,[79] in order to avoid attrition bias. Attrition bias refers to systematic differences between the comparison groups in terms of participants withdrawing or being excluded from the study. Participants may withdraw or drop-out from a study because the treatment has intolerable adverse effects, or on the other hand, they may recover and leave for that reason. They may simply be lost to follow-up, or they may be withdrawn due to a lack of data on outcome measures. Other reasons that participants may be excluded include mistaken randomisation of participants who, on review, did not meet the study inclusion criteria, and participants receiving the wrong intervention due to protocol violation. The likely impact of such withdrawals and exclusions needs to be considered carefully; if the exclusion is related to the intervention and outcome then it can bias the results (for example, not accounting for high numbers of withdrawals due to adverse effects in one intervention arm will unduly favour that intervention). Serious bias can arise as a result of participants being withdrawn for apparently *ad hoc* reasons that are related to the success or failure of an intervention. There is evidence from the field of cancer research that exclusion of patients from the analysis may bias results,[80] though how this may apply to other fields is unclear. An intention to treat (ITT) analysis is generally recommended in order to reduce the risk of bias.

An ITT analysis includes outcome data on all trial participants and analyses them according to the intervention to which they were randomised, regardless of the intervention(s) they actually received. Complete outcome data are often unavailable for participants who drop-out before the end of the trial, so in order to include all participants, assumptions need to be made about their missing outcome data (for example by imputation of missing values). ITT analysis generally provides a more conservative, and potentially less biased, estimate in trials of effectiveness (see *Section 1.3.5.2 Quantitative synthesis of comparative studies*). However, ITT analyses are often poorly described and applied[81] and if assessing methodological quality associated with statistical analysis, care needs to be taken in judging whether the use of ITT analysis has minimized the risk of attrition bias and whether it was appropriately applied. If an ITT analysis is not used, then the study should at least report the proportion of participants excluded from the analysis to allow a researcher to judge whether this is likely to have led to bias.

The minimum criteria for assessment of risk of bias in RCTs are set out in *Box 1.5.* While all these criteria are relevant to assessing risk of bias, their relative importance can be context specific. For example, the importance of blinded outcome assessment will vary depending on whether the outcomes involve subjective judgement (this may vary between different outcomes measured within the same trial). Therefore, when planning which criteria to use it is important to think carefully about what characteristics would realistically be considered ideal. The Cochrane handbook provides a detailed assessment tool for use when assessing risk of bias in an RCT.[82]

Box 1.5: Criteria for assessment of risk of bias in RCTs

- Was the method used to generate random allocations adequate?
- Was the allocation adequately concealed?
- Were the groups similar at the outset of the study in terms of prognostic factors, e.g. severity of disease?
- Were the care providers, participants and outcome assessors blind to treatment allocation? If any of these people were not blinded, what might be the likely impact on the risk of bias (for each outcome)?
- Were there any unexpected imbalances in drop-outs between groups? If so, were they explained or adjusted for?
- Is there any evidence to suggest that the authors measured more outcomes than they reported?
- Did the analysis include an intention to treat analysis? If so, was this appropriate and were appropriate methods used to account for missing data?

Other randomised study designs

In addition to parallel group RCTs, there are other randomised designs where further quality criteria may need to be considered. These are described below.

Randomised cross-over trials

In randomised cross-over trials all participants receive all the interventions. For example in a two arm cross-over trial, one group receives intervention A before intervention B, and the other group receives intervention B before intervention A. It is the sequence of interventions that is randomised. The advantage of cross-over trials is that they are potentially more efficient than parallel trials of a similar size, in which each participant receives only one of the interventions. The criteria for assessing risk of bias in RCTs also apply to cross-over trials, but there are some additional factors that need to be taken into consideration.

The cross-over design is inappropriate for conditions where the intervention may provide a cure or remission, where there is a risk of spontaneous improvement or resolution of the condition, where there is a risk of deterioration over the period of the

trial (e.g. degenerative conditions) or where there is a risk that patients may die.[83] This is because these outcomes lead either to the participant being unable to enter the second period or, on entering the second period, their condition is systematically different from that in the first period.

The possibility of a 'carryover' of the effect of the intervention provided in the first period into the second intervention period is an important concern in this study design.[83] This risk is dealt with by building in a treatment-free or placebo 'washout period' between the intervention periods.[83] The adequacy of the washout period length will need to be considered as part of the assessment of risk of bias.

The statistical analysis appropriate to cross-over trials are discussed in the synthesis section and statistical advice is likely to be required (see *Section 1.3.5 Data synthesis*).

Cluster randomised trials

A cluster randomised trial is a trial where clusters of people rather than single individuals are randomised to different interventions.[84] For example, whole clinics or geographical locations may be randomised to receive particular interventions, rather than individuals.

The distinctive feature of cluster trials is that the outcome for each participant within a cluster may not be independent, since individuals within the cluster are likely to respond in a similar way to the intervention. Underlying reasons for this intra-cluster correlation include individuals in a cluster being affected in a similar manner due to shared exposure to a common environment such as specific hospital policies on discharge times; or personal interactions between cluster members and sharing of attitudes, behaviours and norms that may lead to similar responses.[84] This has implications for estimating the sample size required (i.e. the sample needs to be larger than for an individually randomised trial) and the statistical analysis.

When assessing the risk of selection bias in cluster randomised trials there are two factors that need to be considered: the randomisation of the clusters and how participants within clusters are selected into the study.[85] The first can be dealt with by using an appropriate randomisation method with concealed allocation (clusters are often allocated at the outset). However, where the trial design then requires selection of participants from within a cluster, the risk of selection bias should also be assessed. There is a clear risk of selection bias when the person recruiting participants knows in advance the clinical characteristics of a participant and which intervention they will receive. Also, potential participants may know in advance which intervention their cluster will receive, leading to different participation rates in the comparison groups.[85] Two key methods for reducing bias in the selection of individuals within clusters have been identified: recruitment of individuals prior to the random allocation of clusters and, where this is not possible, use of an impartial individual to recruit participants following randomisation of the clusters.[86]

The statistical analyses appropriate to cluster randomised trials are discussed in *Section 1.3.5 Data synthesis* and statistical advice is likely to be required.

Wider reading is recommended prior to conducting a quality assessment of cluster randomised trials. Several texts discuss the design, analysis and reporting of this trial design.[75, 84, 87, 88]

Quasi-experimental studies

The main distinction between randomised and quasi-experimental studies is the way in which participants are allocated to the intervention and control groups; quasi-experimental studies do not use random assignment to create the comparison groups.

In non-randomised controlled studies, individuals are allocated to concurrent comparison groups, using methods other than randomisation. The lack of concealed randomised allocation increases the risk of selection bias.

Before-and-after studies evaluate participants before and after the introduction of an intervention. The comparison is usually made in the same group of participants, thus avoiding selection bias, although a different group can be used. In this type of design however, it can be difficult to account for confounding factors, secular trends, regression to the mean, and differences in the care of the participants apart from the intervention of interest.

An alternative to this is a 'time series' design. Interrupted time series studies are multiple observations over time that are 'interrupted', usually by an intervention or treatment and thus permit separating real intervention effects from other long-term trends. It is a study design used where others, such as RCTs, are not feasible, for example in the evaluation of a screening service or a mass media campaign. It is also frequently used in policy evaluation, for example to measure the effect of a smoking ban.

The circumstances in which, and extent to which, studies without randomisation are at risk of bias are not fully understood.[89] A key influencing factor may be the extent to which prognosis influences selection for a particular intervention as well as eventual outcome.[89] Because of the risk of bias, careful consideration should be given to the inclusion of quasi-experimental studies in a review to assess the effectiveness of an intervention. If included, researchers should think carefully about the strength of this evidence and how it should be interpreted.

A review of quality assessment tools designed for or used to assess studies without randomisation identified key aspects of quality as being particularly pertinent:[89]

- How the treatment groups were created (how allocation occurred; and whether the study was designed to generate groups that are comparable on key prognostic factors e.g. by 'matching' participants in each group).
- The comparability of intervention and comparison groups at the analysis stage. For example, whether prognostic factors were identified; and whether case-mix adjustment was used to account for any between group differences.

Other quality issues identified were similar to those for assessing performance, detection and attrition bias in RCTs: blinding of participants and investigators; the level of confidence that the participants received the intervention to which they were assigned and experienced the reported outcome as a result of that intervention; the adequacy of the follow-up; and appropriateness of the analysis.

Observational studies

In observational studies the intervention(s) that individuals receive are determined by usual practice or 'real-world' choices, as opposed to being actively allocated as part of the study protocol.

Observational studies are usually more susceptible to bias than experimental studies, and the conclusions that can be drawn from them are necessarily more tentative and are often hypothesis generating, highlighting areas for further research.

Observational designs such as cohort studies, case-control studies and case series are often considered to form a hierarchy of increasing risk of bias. However, such a hierarchy is not always helpful because, as noted before, the same label can be used to describe studies with different design features and there is not always agreement on the definitions of such studies. Attention should focus on specific features of the studies (e.g. participant allocation, outcome assessment) and the extent to which they are susceptible to bias.

In a cohort study design, a defined group of participants is followed over time and comparison is made between those who did and did not receive an intervention (e.g. a study may follow a cohort of women who choose to use oral contraceptives and compare them over time with women who choose other forms of contraception). Prospective cohort studies are planned in advance and define their participants before the intervention of interest and follow them into the future. These are less likely to be susceptible to bias than retrospective cohort studies, which identify participants from past records and follow them from the time of that record.

Case-control studies compare groups from the same population with (cases) and without (controls) a specific outcome of interest, to evaluate the association between exposure to an intervention and the outcome. The risk of selection bias in such studies will be dependent on how the control group was selected. Groups may be matched to make them comparable for potential confounding factors. However, since analysis cannot be performed on matched variables, the matching criteria must be selected carefully, as this can give rise to 'over-matching' when the factors are related to allocation to the intervention.

Case series are observations made on a number of individuals (with no control group) and are not comparative. They can, however, provide useful information, for example about the unintentional effects of an intervention (see *Chapter 4*) and in such situations it is important to assess their quality.

Other issues related to study quality

Choice of outcome measure

As well as using blinding to minimize bias when assessing outcomes, it is usually necessary to consider the reliability or validity of the actual outcome measure being used (e.g. several different scales can be used to measure quality of life or psychological outcomes). It is important that the scales are fully understood to enable comparison, (e.g. a high score implies a favourable outcome in some scales and an unfavourable one in others).

The outcome should also be relevant and meaningful to both the intervention and the evaluation (i.e. a treatment intended to reduce mortality should measure mortality, not merely a range of biochemical indicators).

Statistical issues

Although issues around statistical analysis are less important if the study data are to be combined in a meta-analysis, when studies are not being quantitatively pooled it is also important to assess statistical issues around design and analysis. For example, assessing whether a study is adequately powered to detect an effect of the intervention.[90] The assessment of statistical power may involve relying on the sample size calculation in the primary study, where reported. However, defining population parameters for sample size calculations is a subjective judgement which may vary between investigators;[91] for some review topics it may be appropriate to define *a priori* an adequate sample size for the purposes of the review.

Quality of reporting

Inadequate reporting of important aspects of methodological quality such as allocation concealment, blinding and statistical analysis is common,[92] as is failure to report detail about the intervention and its implementation. Quality of reporting does not necessarily reflect the quality of the underlying methods or data, but when planning quality assessment it is important to decide how to deal with poor reporting. One approach is to assume that if an item is not reported then the criterion has not been met. While this may often be justifiable,[93, 94] there is evidence to suggest that failure to report a method does not necessarily mean it has not been used.[95-97] Therefore it is important to be accurate and distinguish between failure to report a criterion and failure to meet a criterion. For example, a criterion can be described as being met, not met, or unclear due to inadequate reporting.

There have been a number of initiatives aimed at improving the quality of reporting of primary research. The CONSORT statement contains a set of recommendations for the reporting of RCTs,[98] the TREND statement provides guidelines for the reporting of non-randomised evaluations of behavioural and public health interventions,[99] and the STROBE statement is an initiative to improve reporting of observational studies.[100] The EQUATOR network promotes the transparent and accurate reporting of health research in a number of ways, including the use of these consensus reporting guidelines.[101] It is anticipated that implementation of these guidelines will help improve the standard of reporting, which should make quality assessment more straightforward.

Quality of the intervention

In addition to study design, it is often helpful to assess the quality of the intervention and its implementation. At its most simplistic, the quality of an intervention refers to whether it has been used appropriately. This is a fairly straightforward assessment where, for example drug titration studies have been conducted. It is more problematic where there is no preliminary research suggesting that an intervention should be administered in a particular way,[102] or where the intervention requires a technical skill such as surgery or physiotherapy.[103] It is important to establish to what extent these are standardised, as this will affect how the results should be interpreted.

The quality of the intervention is particularly relevant to complex interventions made up from a number of components, which act independently and inter-dependently.[104, 203, 204] These include clinical interventions such as physiotherapy as well as public health interventions such as community-based programmes. The quality of an intervention can be conceptualised as having two main aspects: (i) whether the intervention has been appropriately defined and (ii) whether it has been delivered as planned (the integrity or fidelity of the intervention).

If the quality of the intervention is relevant, the review should assess whether the intervention was implemented as planned in the individual studies (i.e. how many participants received the intervention as planned, whether consistency of implementation was measured, and whether it is likely that participants received an unintended intervention/contamination of the intervention that may influence the results). In some topic areas, for example when a sham device or procedure is being used, it may also be relevant to assess the quality of the comparator. When an intervention relies on the skill of the care provider it may be useful to assess whether the performance of those providing the intervention was measured. For more detailed information on complex interventions see *Chapter 3*.

Generalisability

Generalisability, also known as applicability or external validity, is not considered in detail in this section. In addition to assessing the risk of bias (internal validity), researchers may also consider how closely a study reflects routine practice or the usual setting where the intervention would be implemented. However, this is not an inherent characteristic of a study as the extent to which a study is 'generalisable' depends also on the situation to which the findings are being applied.[105] Therefore the issue of generalisability is also raised in *Section 1.2 The review protocol* in the context of defining inclusion criteria for the review, *Section 1.3.3 Data extraction* and in *Section 1.3.6 Report writing*.

1.3.4.3 The impact of study quality on the estimate of effect

Several empirical studies have explored how quality can influence the results of clinical trials (and therefore the results of reviews of trials). Trials with double-blinding and adequate concealment of allocation have been found to indicate less beneficial treatment effects than trials without these features.[106] Similarly, exclusion of lower quality studies has led to less beneficial effects in meta-analyses.[106] In meta-analyses of subjectively assessed outcomes (e.g. patient reported outcomes), inadequate allocation concealment and lack of blinding have been associated with substantially more beneficial treatment effects, whereas for objective outcomes (e.g. mortality) there was a modest effect of inadequate allocation concealment and no effect of lack of blinding.[107] There is some evidence about the relationship between study quality and the estimate of effect that is contradictory to the above,[108, 109] though this may be due to the data sets used and how specific quality criteria were defined.

1.3.4.4 The process of quality assessment in systematic reviews

There are two main approaches towards assessing quality. One involves the use of checklists of quality items and the other of scales which provide an overall numerical quality score for each study.[110]

Tools for assessing quality

Checklists can be a reliable means of ensuring that all the studies assessed are critically appraised in a standardised way. There are many different checklists and scales readily available, [75, 111-116] which can be modified to meet the requirements of the review, or a new detailed checklist, specific to the review, may be developed.

Because some items included may require a degree of subjective judgement, it is important to pilot the use of the checklist and to ensure that the quality assessment is undertaken independently by two researchers.

The use of scales with summary scores to distinguish high and low quality studies is questionable and not recommended.[117, 118] Very few scales have been developed using standard techniques to establish their validity and reliability.[113] The weighting assigned to methodological items varies considerably between scales,[117] and does not usually take into account the direction of bias.[119] An investigation comparing low-molecular-weight heparin (LMWH) with standard heparin for thromboprophylaxis in general surgery found that trials identified as 'high quality' by some of the 25 scales investigated indicated that LMWH was not superior to standard heparin, whereas trials identified as 'high quality' by other scales led to the opposite conclusion, that LMWH was beneficial.[117] It is therefore preferable that aspects of quality such as blinding and treatment allocation (and their potential impact on study results) should be considered individually.[117]

Checklists by type of study design

In general checklists tend to be specific to particular study designs, and where reviews include more than one type of study design, separate lists can be used or a combined list selected or developed. Checklists have also been developed for use with both randomised and non-randomised studies such as that by Downs and Black.[111]

There are multiple systems available for the evaluation of RCTs,[112, 113] in addition to the Cochrane handbook assessment tool for assessing risk of bias.[82] In a review of checklists for the assessment of non-randomised studies, nearly 200 tools were identified. From these, six were recommended as being suitable for use in systematic reviews including non-randomised studies.[89] The Cochrane Effective Practice and Organisation of Care Group (EPOC) have developed guidelines to assist researchers in making decisions about when to include studies that use interrupted time series designs and how to assess their methodological quality.[115, 116] A useful checklist for observational studies was published as part of the US Agency for Healthcare Research and Quality's (AHRQ) 'Systems to Rate the Strength of Scientific Evidence'.[112] The most recent version of the Cochrane Handbook also contains guidance on dealing with non-randomised studies in systematic reviews of interventions, from the protocol to synthesis stages.[75]

How will the quality assessment information be used?

Simply reporting which quality criteria were met by studies included in a systematic review is not sufficient. The implications of the quality assessment for interpreting results need to be explicitly considered.

Study quality can be incorporated into the synthesis either quantitatively through subgroup or sensitivity analyses (see *Section 1.3.5.2: Quantitative synthesis*), or in a narrative synthesis. In the latter, the quality assessment can be used to help interpret and explain differences in results across studies (e.g. unblinded studies with subjective outcomes may have consistently larger effects than blinded studies) and inform a qualitative interpretation of the risk of bias (see *Section 1.3.5.1 Narrative synthesis*).

Summary: Quality assessment

- An important part of the systematic review process is to assess the risk of bias in included studies caused by inadequacies in study design, conduct or analysis that may have led to the treatment effect being over or underestimated.
- Various tools are available but there is no single tool that is suitable for use in all reviews. Choice should be guided by:
    - Study design
    - The level of detail required in the assessment
    - The ability to distinguish between internal validity (risk of bias) and external validity (generalisability)
- Using quality scores is problematic; it is preferable to consider individual aspects of methodological quality in the quality assessment and synthesis.
- Where appropriate, the potential impact that methodological quality had on the findings of the included studies should be considered.
- Detailed quality assessment can be time consuming if a review includes a large number of studies and may require considerable expertise in critical appraisal. If resources are limited, priority should be given to assessment of the key sources of bias.

1.3.5 Data synthesis

Synthesis involves the collation, combination and summary of the findings of individual studies included in the systematic review. Synthesis can be done quantitatively using formal statistical techniques such as meta-analysis, or if formal pooling of results is inappropriate, through a narrative approach. As well as drawing results together, synthesis should consider the strength of evidence, explore whether any observed effects are consistent across studies, and investigate possible reasons for any inconsistencies. This enables reliable conclusions to be drawn from the assembled body of evidence.

Deciding what type of synthesis is appropriate

Many systematic reviews evaluating the effects of health interventions focus on evidence from RCTs, the results of which, generally, can be combined quantitatively. However, not all health care questions can be addressed by RCTs, and systematic reviews do not automatically involve statistical pooling. Meta-analysis is not always possible or sensible. For example, pooling results obtained from diverse non-randomised study types is not recommended.[120] Similarly, meta-analysis of poor quality studies could be seriously misleading as errors or biases in individual studies would be compounded and the very act of synthesis may give credence to poor quality studies. However, when used appropriately, meta-analysis has the advantage of being explicit in the way that data from individual studies are combined, and is a powerful tool for combining study findings, helping avoid misinterpretation and allowing meaningful conclusions to be drawn across studies.

The planned approach should be decided at the outset of the review, depending on the type of question posed and the type of studies that are likely to be available. There may be topics where it can be decided *a priori* that a narrative approach is appropriate. For example, in a systematic review of interventions for people bereaved by suicide, it was anticipated there would be such diversity in the included studies, in terms of settings, interventions and outcome measures, that a narrative synthesis alone was proposed in the protocol.[121]

Narrative and quantitative approaches are not mutually exclusive. Components of narrative synthesis can be usefully incorporated into a review that is primarily quantitative in focus and those that take a primarily narrative approach can incorporate some statistical analyses such as calculating a common outcome statistic for each study.

Initial descriptive synthesis

Both quantitative and narrative synthesis should begin by constructing a clear descriptive summary of the included studies. This is usually done by tabulating details about study type, interventions, numbers of participants, a summary of participant characteristics, outcomes and outcome measures. An indication of study quality or risk of bias may also be given in this or a separate table (see *Section 1.3.2 Study selection* and *Section 1.3.4 Quality assessment*). An example is given in *Table 1.1*. If the review will not involve re-calculating summary statistics, but will rather rely on the reported results of the author's analyses, these may also be included in the table. The descriptive process should be both explicit and rigorous and decisions about how to group and tabulate data should be based on the review question and what has been planned in the protocol. This initial phase will also be helpful in confirming that studies are similar and reliable enough to synthesise, and that it is appropriate to pool results.

Table 1.1: Example table describing studies included in a systematic review of the effectiveness of drug treatments for attention deficit hyperactivity disorder in children and adolescents. [122]

Study	Design	Intervention – N	Age (years)	Duration (weeks)	Core outcomes
Administered once daily<br>Rapport, 1989	C (5x)	MPH (5 mg/day, o.d.) – 45<br>MPH (10 mg/day, o.d.) – 45<br>MPH (15 mg/day, o.d.) – 45	5–12	5	Core: no hyp; Abbreviated CTRS: total score<br>QoL: not reported<br>AE: not reported
DuPaul, 1993	C (5x)	MPH (5 mg/day, o.d.) – 31<br>MPH (10 mg/day, o.d.) – 31<br>MPH (15 mg/day, o.d.) – 31	6–11	6	Core: No hyp; Abbreviated CTRS: total score<br>QoL: not reported<br>AE: not reported
Werry, 1980	C (3x)	MPH (0.40 mg/kg, o.d.) – 30	5.5–12.5	4	Core: Conners' Teacher Questionnaire: hyperactivity; Conners' Parent Questionnaire: hyperactivity<br>QoL: CGI (physician)<br>AE: weight
Administered two or more times daily<br>Brown, 1988	C (4x)	MPH (8.76 mg/day, b.d.) – 11	13–15	8	Core: CPRS: Hyperactivity Index; Conners' Teacher Hyperactivity Index; ACTeRS: hyperactivity<br>QoL: not reported<br>AE: SERS (parents); weight
Fischer, 1991	C (3x)	MPH (0.40 mg/kg/day, b.d.) – 161	2.4–17.2	3	Core: CPRS-R: Hyperactivity Index; CTRS-R: hyperactivity index; CTRS-R: hyperactivity<br>QoL: not reported<br>AE: CPRS-R: psychosomatic; SERS (parents, teachers): number of side-effects, mean severity rating
Fitzpatrick, 1992	C (4x)	MPH (10–15 mg/day, b.d.) – 19	6.9–11.5	8	Core: Conners' Hyperactivity Index (parents and teacher); TOTS: hyperactivity (parents and teachers)<br>QoL: no CGI; comments ratings (parent/teacher)<br>AE: STESS (parents); weight

Study	Design	Intervention – N	Age (years)	Duration (weeks)	Core outcomes
Fine, 1993	C (3x)	MPH [0.30 mg/kg/day (unclear), b.d.] – 12	6–10	3	Core: not reported<br>QoL: not reported<br>AE: side-effects questionnaire
Hoeppner, 1997	C (3x)	MPH (0.30 mg/kg/day, b.d.) – 50	6.1–18.2	4	Core: CPRS: Hyperactivity Index; CTRS: Hyperactivity Index<br>QoL: not reported<br>AE: not reported
Handen, 1999	C (3x)	MPH (12–15 mg/day, max. 3x) – 11	4–5.1	3	Core: CTRS: Hyperactivity Index; CTRS: hyperactivity<br>QoL: not reported<br>AE: Side Effects Checklist (teachers, parents); mean severity rating 0–6
Manos, 1999	C (4x)	MPH (10 mg/day, b.d.) – 42	5–17	4	Core: no hyp; ASQ (parents and teachers); ARS (parent)<br>QoL: no CGI; composite ratings (clinician)<br>AE: Side Effects Behaviour Monitoring Scale (parents)
Barkley, 2000	C (5x)	MPH (10 mg/day, b.d.) – 38	12–17	5	Core: no hyp; ADHD Total Parent/Teacher rating<br>QoL: not reported<br>AE: number and severity of side-effects (teachers, parents, self)
Tervo, 2002	C (3x)	MPH (0.10 mg/kg/day, b.d.) – 41	M=9.9 (2.9)	3	Core: no hyp; CBCL (parent)<br>QoL: not reported<br>AE: not reported

ACTeRS, ADD-H Comprehensive Teachers' Rating Scale; AE, adverse effects; ARS, ADHD Rating Scale; ASQ, Abbreviated Symptoms Questionnaire; b.d., twice daily; C, cross-over trial (number of cross-overs); CBCL, Child Behaviour Checklist; CGI, Clinical Global Impression; CPRS, Conners' Parent Rating Scale; CTRS, Conners' Teacher Rating Scale; MPH, methylphenidate hydrochloride; N, number of participants; o.d., once daily; P, parallel trial; hyp, hyperactivity; PACS, Parental Account of Childhood Symptoms; SERS, Side Effects Rating Scale.

1.3.5.1 Narrative synthesis

All systematic reviews should contain text and tables to provide an initial descriptive summary and explanation of the characteristics and findings of the included studies. However simply describing the studies is not sufficient for a synthesis. The defining characteristic of narrative synthesis is the adoption of a textual approach that provides an analysis of the relationships within and between studies and an overall assessment of the robustness of the evidence.

A narrative synthesis of studies may be undertaken where studies are too diverse (either clinically or methodologically) to combine in a meta-analysis, but even where a meta-analysis is possible, aspects of narrative synthesis will usually be required in order to fully interpret the collected evidence.

Narrative synthesis is inherently a more subjective process than meta-analysis; therefore, the approach used should be rigorous and transparent to reduce the potential for bias. The idea of narrative synthesis within a systematic review should not be confused with broader terms like ‘narrative review’, which are sometimes used to describe reviews that are not systematic.

A general framework for narrative synthesis

How narrative syntheses are carried out varies widely, and historically there has been a lack of consensus as to the constituent elements of the approach or the conditions for establishing credibility. A project for the Economic and Social Research Council (ESRC) Methods Programme has developed guidance on the conduct of narrative synthesis in systematic reviews.[123-126] The guidance offers both a general framework and specific tools and techniques that help to increase the transparency and trustworthiness of narrative synthesis.

The general framework consists of four elements:

- Developing a theory of how the intervention works, why and for whom
- Developing a preliminary synthesis of findings of included studies
- Exploring relationships within and between studies
- Assessing the robustness of the synthesis

Though the framework is divided into these four elements, the elements themselves do not have to be undertaken in a strictly sequential manner, nor are they totally independent of one another. A researcher is likely to move iteratively among the activities that make up these four elements.

For each element of the framework, this guidance presents a range of practical tools and techniques. It is not mandatory (or indeed appropriate) to employ each one of these for every narrative synthesis, but the appropriate tools/techniques should be selected depending upon the nature of the evidence being synthesised. The reason for the choice of tool or technique should be specified in the methods section of the review.

A fuller description of these tools and techniques and narrative synthesis in general can be found in the ESRC guidance report.[125, 126] It should be noted that the list given here is not comprehensive and other tools and techniques may be appropriate in certain circumstances.

The four elements of the narrative synthesis framework (and some of their related tools and techniques) are described below (*Figure 1.2*).[126]

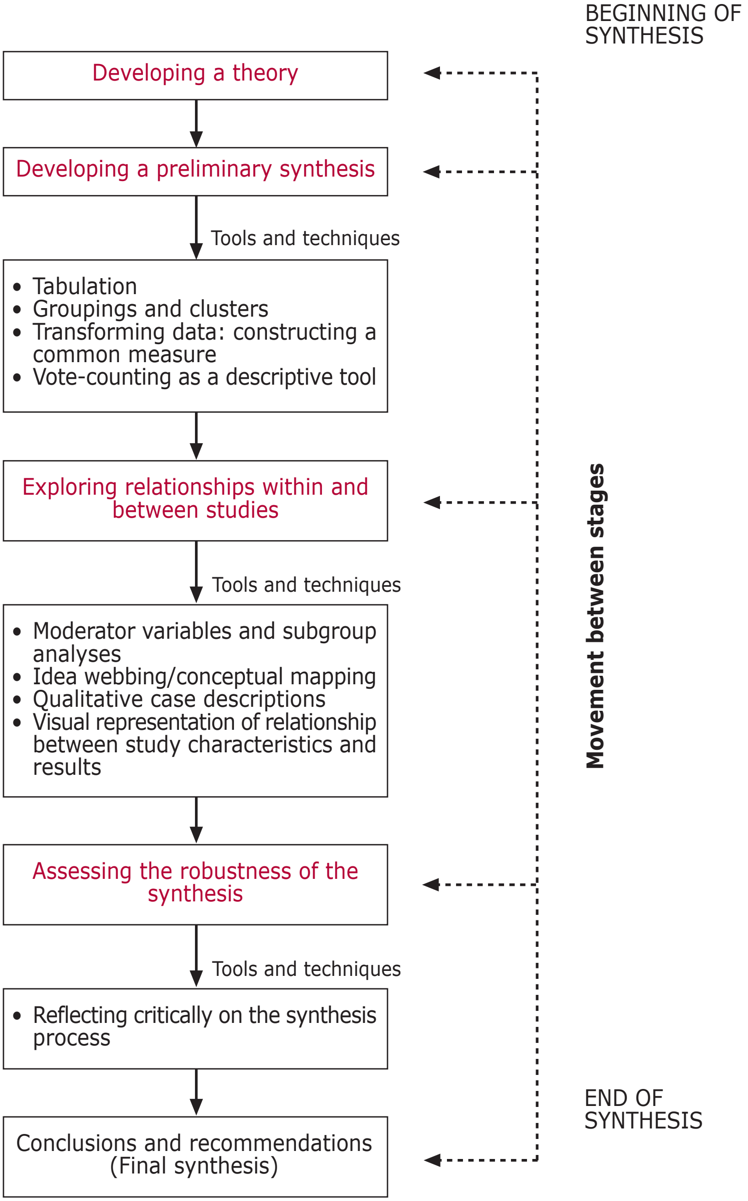

Figure 1.2: Example of applying the narrative synthesis framework

Developing a theory of how the intervention works, why and for whom

The extent to which theory will play a role will partly depend upon the type of intervention(s) being evaluated. For example, theory may only play a minor role in a systematic review looking at the effects of a single therapeutic drug on patient outcomes because many aspects of the 'mechanism of action' will have been established in early studies investigating pharmacodynamics, dose-finding etc. Alternatively, in a systematic review evaluating the effects of a psychosocial or educational programme, theories about the causal chain linking the intervention to the outcomes of interest will be of crucial importance and might be presented descriptively or in diagrammatic form, as displayed in *Figure 1.3*.

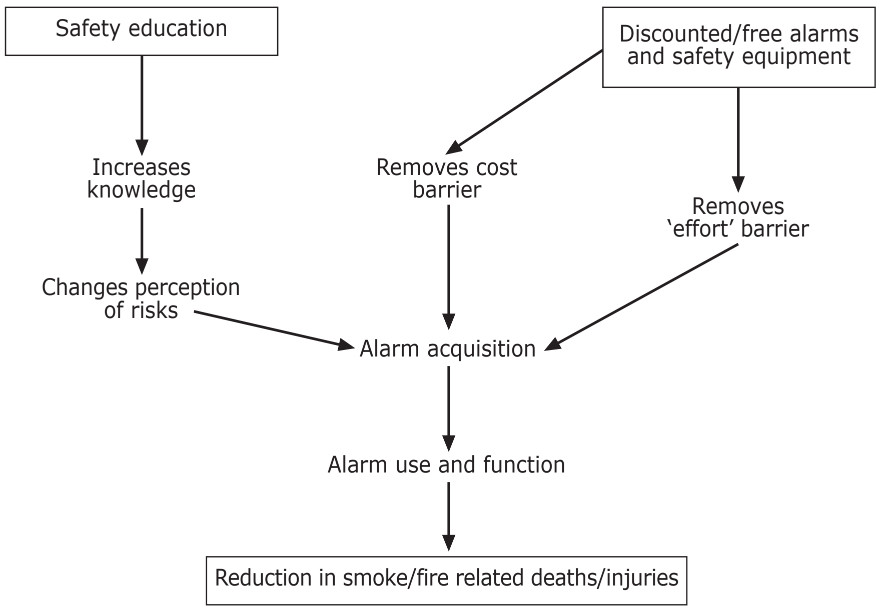

Figure 1.3: Interventions to increase use and function of smoke alarms: implicit theory of change model

Developing a preliminary synthesis of findings of included studies

Once the relevant studies have been data extracted, the first step is to bring together, organise and describe their findings. The direction and size of the reported effects may be the starting point. Or, for example, a collection of studies evaluating one kind of intervention might be divided into subgroups of studies with distinct populations, such as children and adults. It is important to remember that this is only the first step of the synthesis. The remaining elements of the framework need to be taken into account before it can be considered adequate as a narrative synthesis.

Table 1.2 describes a range of tools and techniques that might be employed at this stage of the synthesis.

Table 1.2: Developing a preliminary synthesis of findings of included studies

Textual descriptions of studies	A descriptive paragraph on each included study. These descriptions should be produced in a systematic way, including the same type of information for all studies if possible and in the same order. It may be useful for recording purposes to do this for all excluded studies as well.
Groupings and clusters	The included studies might be grouped at an early stage of the review, though it may be necessary to refine these initial groups as the synthesis develops. This can also be a useful way of aiding the process of description and analysis and looking for patterns within and across groups. It is important to use the review question(s) to inform decisions about how to group the included studies.
Tabulation	A common approach, used to represent data visually. The way in which data are tabulated may affect readers' impressions of the relationships between studies, emphasising the importance of a narrative interpretation to supplement the tabulated data.
Transforming data into a common measure	In both narrative and quantitative synthesis it is important to ensure that data are presented in a common measure to allow an accurate description of the range of effects.
Vote-counting as a descriptive tool	Simple vote-counting might involve the tabulation of findings according to direction of effect. More complex approaches can be developed both in terms of the categories used and by assigning different weights or scores to different categories. However, vote-counting can disregard sample size and be misleading. So, the interpretation of the results must be approached with caution and subjected to further scrutiny.
Translating data: thematic analysis	A technique used in the analysis of qualitative data in primary research can be used to systematically identify the main, recurrent and/or most important (based on the review question) themes and/or concepts across multiple studies.[127]
Translating data: content analysis	A technique for compressing many words of text into fewer content categories based on explicit rules of coding.[128] Unlike thematic analysis, it is essentially a quantitative method, since all the data are eventually converted into frequencies.

Exploring relationships within and between studies

Patterns emerging from the data during the preliminary synthesis need to be rigorously scrutinised in order to identify factors that might explain variations in the size/direction of effects. At this stage there is a clear attempt to explore relationships between: (a) characteristics of individual studies and their reported findings; and (b) the findings of different studies.

However, when exploring heterogeneity in this way, it is necessary to be wary of uncovering associations between characteristics and results that are based on comparisons of many subgroups – some of these may simply have occurred by chance.

Subgroup comparisons which are specified in advance (i.e. as part of the review protocol) are more likely to be plausible than those which are not.[129, 130]

The extent to which these factors can be explored in the review depends on how clearly they are reported in the primary research studies. The amount of detail may depend on the type of publication and the nature of the intervention being reviewed (e.g. highly standardised interventions may not be described as fully as more unusual ones).

Tools and techniques that might be employed at this stage of the synthesis are described in *Table 1.3.*

Table 1.3: Exploring relationships within and between studies

Graphs, frequency distributions, funnel plots, forest plots and L'Abbe plots	There are several visual or graphical tools that can help reviewers explore relationships within and between studies. These include: presenting results in graphical form; plotting findings (e.g. effect size) against study quality; plotting confidence intervals; and/or plotting outcome measures.
Moderator variables and subgroup analyses	This refers to the analysis of variables which can be expected to moderate the main effects being examined in the review. This can be done at the study level, by examining characteristics that vary between studies (such as study quality, study design or study setting) or by analysing characteristics of the sample (such as subgroups of participants).
Idea webbing and conceptual mapping	Involves using visual methods to help to construct groupings and relationships. The basic idea underpinning these approaches is (i) to group findings that are empirically and/or conceptually similar and (ii) to identify (again on the basis of empirical evidence and/or conceptual/theoretical arguments) relationships between these groupings.
Qualitative case descriptions	Any process in which descriptive data from studies included in the systematic review are used to try to explain differences in statistical findings. For example why one intervention outperforms another apparently similar intervention or why some studies are statistical outliers.
Investigator/methodological/ conceptual triangulation	Triangulation makes use of a combination of different perspectives and/or assessment methods to study a particular phenomenon. This could apply to the methodological and theoretical approaches adopted by the researchers undertaking primary studies included in a systematic review, e.g. investigator triangulation explores the extent to which heterogeneity in study results may be attributable to the diverse approaches taken by different researchers. Triangulation involves analysing the data in relation to the context in which they were produced, notably the disciplinary perspectives and expertise of the researchers producing the data.

Assessing the robustness of the synthesis

Towards the end of the synthesis process, the analysis of relationships as described above should lead into an overall assessment of the strength of the evidence. This is essential when drawing conclusions based on the narrative synthesis.

Robustness can relate to the methodological quality of the included studies (such as risk of bias), and/or the credibility of the product of the synthesis process. Obviously, these are related. The credibility of a synthesis will depend on both the quality and the quantity of the evidence base it is built on, and the method of synthesis and the clarity/transparency of its description. If primary studies of poor methodological quality are included in the review in an uncritical manner then this will affect the integrity of the synthesis. Attempts to minimize the introduction of bias might include 'weighting' the findings of studies according to technical quality (i.e. giving greater credence to the findings of more methodologically sound studies) and providing a clear justification for this. Similarly, a clear description of the potential sources of bias within the synthesis process itself helps establish credibility with the reader.

Table 1.4 describes the tools and techniques that might be employed at this stage of the synthesis.

Table 1.4: Assessing the robustness of the synthesis

Use of validity assessment	Use of specific rules to define weak, moderate or good evidence. An example is the approach used by the US Centers for Disease Control and Prevention[131] although there are many other evidence grading systems available. Decisions about the strength of evidence are explicit although the criteria used are often debated.
Reflecting critically on the synthesis process	Use of a critical discussion to address methodology of the synthesis used[132] (especially focusing on its limitations and their potential influence on the results); evidence used (quality, validity, generalisability) – with emphasis on the possible sources of bias and their potential influence on results of the synthesis; assumptions made; discrepancies and uncertainties identified; expected changes in technology or evidence (e.g. identified ongoing studies); aspects that may have an influence on implementation and effectiveness in real settings. Such a discussion would provide information on both the robustness and generalisability of the synthesis.
Checking the synthesis with authors of primary studies	It is possible to consult with the authors of included primary studies in order to test the validity of the interpretations developed during the synthesis and the extent to which they are supported by the primary data.[133] The authors of the primary studies may have useful insights into the possible accuracy and generalisability of the synthesis; this is most likely to be useful when the number of primary studies is small. This is a technique that has been used with qualitative evidence.

1.3.5.2 Quantitative synthesis of comparative studies

As with narrative synthesis, quantitative synthesis should be embedded in a review framework that is based on a clear hypothesis, should consider the direction and size of any observed intervention effects in relation to the strength of evidence, and should explore relationships within and between studies. The requirements for a careful and thoughtful approach, the need to assess the robustness of syntheses, and to reflect critically on the synthesis process, apply equally but are not repeated here.

This section aims to outline the rationale for quantitative synthesis of comparative studies and to focus on describing commonly used methods of combining study results and exploring heterogeneity. A more detailed overview of quantitative synthesis for systematic review is given in the Cochrane Handbook.[75] Comprehensive accounts are also given by Whitehead[134] and Cooper and Hedges,[135] and a discussion of recent developments and more experimental approaches is given in a paper by Sutton and Higgins.[136]

Decisions about which comparisons to make, and which outcomes and summary effect measures to use, should have been addressed as part of the protocol development. However, as synthesis depends partly on what results are actually reported, some planned analyses may not be possible, and others may have to be adapted or developed. Any departures from the analyses planned in the protocol should be clearly justified and reported.

Decisions about what studies should and should not be combined are inevitably subjective and require careful discussion and judgement. As far as possible *a priori* consideration at the time of writing the protocol is desirable. There will always be differences between studies that address a common question. Reserving meta-analyses for only those studies that evaluate exactly the same interventions in near identical participant populations would be severely limiting and seldom achievable in practice. For example, whilst it may not be sensible to average the results of studies using different classes of experimental drugs or comparators, it may be reasonable to combine results of studies that use analogues or drugs with similar mechanisms of action. Likewise, it will often be reasonable to combine results of studies that have used similar but not identical comparators (e.g. placebo and no treatment). Where there are substantial differences between studies addressing a broadly similar question, although combining their results to give an estimate of an average effect may be meaningless, a test of whether an overall effect is present might be informative. It can be useful to calculate summary statistics for each individual study to show the variability in results across studies. It may also be helpful to use meta-analysis methods to quantify this heterogeneity, even when combined estimates of effect are not produced.

Reasons for meta-analysis

Combining the results of individual studies in a meta-analysis increases power and precision in estimating intervention effects. In most areas of health care, 'breakthroughs' are rare and we may reasonably expect that new interventions will lead to only modest improvements in outcome; such improvements can of course be extremely important to individuals and of significant benefit in terms of population health. Large numbers of events are required to detect modest effects, which are easily obscured by the play

of chance, and studies are often too small to do so reliably. Thus, in any group of small trials addressing similar questions, although a few may have demonstrated statistically significant results by chance alone, most are likely to be inconclusive. However, combining the results of studies in a meta-analysis provides increased numbers of participants, reduces random error, narrows confidence intervals, and provides a greater chance of detecting a real effect as statistically significant (i.e. increases statistical power). Meta-analysis also allows observation and statistical exploration of the pattern of results across studies and quantification and exploration of any differences.

Combining comparative study results in a meta-analysis

Most meta-analyses take a two-step approach in that they first analyse the outcome of interest and calculate summary statistics for each individual study. In the second stage, these individual study statistics are combined to give an overall summary estimate. This is usually calculated as a weighted average of the individual study estimates. The greater the weight awarded to a study, the more it influences the overall estimate. Studies are usually, at least in part, weighted in inverse proportion to their variance (or standard error squared), a method which essentially gives more weight to larger studies and less weight to smaller studies. It is also possible to weight studies according to other factors such as trial quality, but such methods are very seldom implemented and not recommended.

Two main statistical models are used. Fixed-effect models weight the contribution of each study proportional to the amount of information observed in the study. This considers only variability in results within studies and no allowance is made for variation between studies. Random-effects models allow for between-study variability in results by weighting studies using a combination of their own variance and the between-study variance. Where there is little between-study variability, the within-study variance will dominate and the random-effects weighting will tend towards that of the fixed-effect weighting. If there is substantial between-study variability, this dominates the weighting factor and within-study variability contributes little to the analysis. In this way, all trials will tend towards contributing equally towards the overall estimate and it can be argued that small studies will unduly influence the estimate. Those in favour of random-effects argue that it formally allows for between-study variability and that the fixed-effect approach unrealistically assumes a single effect across trials and gives over-precise estimates. In practice, with well-defined questions, the results of both approaches are often very similar and it is common to run both to test robustness of the choice of statistical model.

Generic inverse variance method of combining study results

The generic inverse variance method is a widely used and easy to implement method of combining study results that underlies many of the approaches that are described later. It is very flexible and can be used to combine any type of effect measure provided that an effect estimate and its standard error is available from each study. Effect estimates may include adjusted estimates, estimates corrected for clustering and repeat measurements, or other summaries derived from more complex statistical methods.

A fixed-effect meta-analysis using the generic inverse variance method calculates a weighted average of study effect estimates (EE_{IV}) by summing individual effect

estimates (EE_i), for example, the log odds ratio or the mean difference, and weighting these by the reciprocal of their squared standard errors (SE_i) as follows:[137]

$$EE_{IV} = \frac{\sum EE_i \cdot \frac{1}{SE_i^2}}{\sum \frac{1}{SE_i^2}}$$

A random-effects approach involves adjusting the study specific standard errors to incorporate between-study variation, which can be estimated from the effects and standard errors associated with the included studies.[138]

Types of data

Other ways to combine studies of effectiveness are available, some of which are specific to the nature of the data that have been collected, analysed and presented in the included studies.

Box 1.6: Illustration of how to calculate risk ratio, relative and absolute risk reduction, and odds ratios and their standard errors

	Individuals with event		Individuals without event			
	Notation	Example	Notation	Example	Total	
Experimental group	*a*	2	*b*	18	n_e	20
Control group	*c*	4	*d*	16	n_c	20
Total		6		34	*N*	40

Risk ratio

$$RR = \frac{\text{risk of event in experimental group}}{\text{risk of event in control group}} = \frac{\left(\frac{a}{n_e}\right)}{\left(\frac{c}{n_c}\right)}$$

$$RR = \frac{\left(\frac{2}{20}\right)}{\left(\frac{4}{20}\right)} = 0.5$$

$$\ln(RR) = \ln\left[\frac{\left(\frac{a}{n_e}\right)}{\left(\frac{c}{n_c}\right)}\right]$$

$$\ln(0.5) = -0.69$$

$$SE_{\ln(RR)} = \sqrt{\frac{1}{a} + \frac{1}{c} - \frac{1}{n_e} - \frac{1}{n_c}}$$

$$SE_{\ln(0.5)} = \sqrt{\frac{1}{2} + \frac{1}{4} - \frac{1}{20} - \frac{1}{20}} = 0.81$$

(Continued)

Relative risk reduction

$$RRR = (1 - RR) \times 100\%$$

$$RRR = (1 - 0.5) \times 100\% = 50\%$$

Odds ratio

$$OR = \frac{\text{odds of event in experimental group}}{\text{odds of event in control group}} = \frac{\left(\frac{a}{b}\right)}{\left(\frac{c}{d}\right)} = \frac{a \times d}{b \times c}$$

$$OR = \frac{\left(\frac{2}{18}\right)}{\left(\frac{4}{16}\right)} = \frac{2 \times 16}{18 \times 4} = 0.44$$

$$\ln(OR) = \ln\left[\frac{a \times d}{b \times c}\right]$$

$$\ln(0.44) = -0.82$$

$$SE_{\ln(OR)} = \sqrt{\frac{1}{a} + \frac{1}{b} + \frac{1}{c} + \frac{1}{d}}$$

$$SE_{\ln(OR)} = \sqrt{\frac{1}{2} + \frac{1}{18} + \frac{1}{4} + \frac{1}{16}} = \sqrt{0.87} = 0.93$$

Peto odds ratio

$$OR_{Peto} = \exp\left(\frac{\text{observed} - \text{expected events}}{\text{variance}}\right) = \exp\left(\frac{O - E}{v}\right)$$

$$OR_{Peto} = \exp\left(\frac{2 - 3}{1.31}\right) = 0.47$$

where $E = \left(\frac{n_e}{N}\right) \times (a + c)$

$$E = \left(\frac{20}{40}\right) \times (2 + 4) = 3$$

and $v = \frac{n_e\, n_c\, (a + c)(b + d)}{N^2 (N - 1)}$

$$v = \frac{20 \times 20 \times 6 \times 34}{40^2 (40 - 1)} = 1.31$$

$$\ln(OR_{Peto}) = \ln\left[OR_{Peto}\right]$$

$$\ln(OR_{Peto}) = -0.76$$

$$SE_{\ln(OR_{Peto})} = \sqrt{\frac{1}{v}}$$

$$SE_{\ln(OR_{Peto})} = \sqrt{\frac{1}{1.31}} = 0.87$$

Dichotomous/binary outcomes

Dichotomous outcomes are those that either happen or do not happen and an individual can be in one of only two states, for example having an acute myocardial infarction or not having an infarction. Dichotomous outcomes are most commonly expressed in terms of risks or odds. Although, in everyday use, the terms risk and odds are often used to mean the same thing, in the context of statistical evaluation they have quite specific meanings.

Risk describes the probability with which a health outcome will occur and is often expressed as a decimal number between 0.0 and 1.0, where 0.0 indicates that there is no risk of the event occurring, and 1.0 indicating certainty that the event will take place. A risk of 0.4 indicates that about four in ten people will experience the event. Odds describe the ratio of the probability that an event will happen to the probability that it

will not happen and can take any value between zero and infinity. Odds are sometimes expressed as the ratio of two integers such that 0.001 can be written 1:1000 indicating that for every one individual who will experience the event, one thousand will not.

Risk ratios (RR), also known as relative risks, indicate the change in risk brought about by an intervention and are calculated as the probability of an event in the intervention group divided by the probability of an event in the control group (where the probability of an event is estimated by the total number of events observed in the group divided by the total number of individuals in that group). A risk ratio of 2.0 indicates that the intervention leads to the risk becoming twice that of the comparator. A risk ratio of 0.75 indicates that the risk has been reduced to three quarters of that of the comparator. This can also be expressed in terms of a reduction in risk whereby the relative risk reduction (RRR) is given as one minus the risk ratio multiplied by 100. For example, a risk ratio of 2.0 corresponds to a relative risk reduction of −100% (a 100% increase), while a risk ratio of 0.75 corresponds to a relative risk reduction of 25%. *Box 1.6* illustrates the calculation of these measures and further details of the formulae can be found elsewhere.[137]

Risk ratios can be combined using the generic inverse variance method applied to the log risk ratio and its standard error (either in a fixed effect or a random-effects model). Odds ratios (OR) describe the ratio of the odds of events occurring on treatment to the odds of events occurring on control, and therefore describes the multiplication of the odds of the outcome that occur with use of the intervention. *Box 1.6* illustrates how to calculate the odds ratio for a single study. Odds ratios can be combined using the generic inverse variance method applied to the log odds ratio and its standard error as described above.

The Mantel-Haenszel method for combining risk ratios or odds ratios, which uses a different weighting scheme, is more robust when data are sparse, but assumes a fixed effect model.[137]

The Peto odds ratio[139] (OR_{Peto}) is an alternative estimate of a combined odds ratio in a fixed effect model, and is based on the difference between the observed number of events and the number of events that would be expected (O − E) if there was no difference between experimental and control interventions (see *Box 1.6*). Combining studies using the Peto method is straightforward, and it may be particularly useful for meta-analysis of dichotomous data when event rates are very low, and where other methods fail.

$$OR_{PetoCombined} = \exp\left(\frac{\text{sum of (O} - \text{E) across studies}}{\text{sum of (v) across studies}}\right)$$

This approach works well when the effect is small (that is when the odds ratio is close to 1.0), events are relatively uncommon, and there are similar numbers in the experimental and control groups. The approach is commonly used to combine data from cancer trials which generally conform to these expectations. Correction for zero cells is not necessary (see below) and the method appears to perform better than alternative approaches when events are very rare. It can also be used to combine time-to-event data by pooling log rank observed minus expected (O − E) events and associated variance. However, the Peto method does give biased answers in some circumstances,

especially when treatment effects are very large, or where there is a lack of balance in treatment allocation within the individual studies.[140] Such conditions will not usually apply to RCTs but may be particularly important when combining the results of observational studies which are often unbalanced.

Although both risk ratios and odds ratios are perfectly valid ways of describing a treatment effect, it is important to note that they are not the same measure, cannot be used interchangeably and should not be confused. When events are relatively rare, say less than 10%,[141] differences between the two will be small, but where the event rate is high, differences will be large. For treatments that increase the chance of events, the odds ratio will be larger than the risk ratio and for interventions that reduce the chance of events, the odds ratio will be smaller than the risk ratio. Thus if an odds ratio is misinterpreted as a risk ratio it will lead to an overestimation of the effect of intervention. Unfortunately, this error in interpretation is quite common in published reports of individual studies and systematic reviews. Although some statisticians prefer odds ratios owing to their mathematical properties (they do not have inherent range limitations associated with high baseline rates and naturally arise as the antilog of coefficients in mathematical modelling, making them more suitable for statistical manipulation), they have been criticised for not being well understood by clinicians and patients.[142, 143] It may therefore be preferable, even when calculations have been based on odds ratios, to transform the findings to describe results as changes in the more intuitively understandable concept of risk.

Neither the risk ratio nor the odds ratio can be calculated for a trial if there are no events in the control group (as calculation would involve division by zero), and so in this situation it is customary to add 0.5 to each cell of the 2x2 table.[137] If there are no events (or all participants experience the event) in both groups, then the trial provides no information about relative probability and so it is omitted from the meta-analysis. These situations are likely to occur when the event of interest is rare, and in such situations the choice of effect measure requires careful thought. A simulation study has shown that when events are rare, most meta-analysis methods give biased estimates of effect,[144] and that the Peto odds ratio (which does not require a 0.5 correction) may be the least biased.

Continuous outcomes

Continuous outcomes are those that take any value in a specified range and can theoretically be measured to many decimal places of accuracy, for example, blood pressure or weight. Many other quantitative outcomes are typically treated as continuous data in meta-analysis, including measurement scales. Continuous data are usually summarized as means and presented with an indication of the variation around the mean using the standard deviation (SD) or standard error (SE). The effect of an intervention on a continuous outcome is measured by the absolute difference between the mean outcome observed for the experimental intervention and control, termed the mean difference (MD). This estimates the amount by which the treatment changes the outcome on average and is expressed:

$$MD = Mean_{experimental} - Mean_{control}$$

$$SE_{MD} = \sqrt{\left(\frac{SD^2_{experimental}}{n_{experimental}}\right) + \left(\frac{SD^2_{control}}{n_{control}}\right)}$$

Study mean differences and their associated standard errors can be combined using the generic inverse variance method.

Where studies assess the same outcome but measure it using different scales (for example, different quality of life scales), the individual study results must be standardised before they can be combined. This is done using the standardised mean difference (SMD), which considers the effect size in each study relative to the variability in the study and is calculated as the mean difference divided by the standard deviation among all participants. Where scales differ in direction of effect (i.e. some increase with increasing severity of outcome whilst others decrease with increasing severity), this needs to be accounted for by assigning negative values to the mean of one set of studies thereby giving all scales the same direction of measurement. There are three commonly used methods of recording the effect size in the standardised mean difference method, Cohen's d,[145] Hedges adjusted g,[145] and Glass' delta.[146] The first two differ in whether the standard deviation is adjusted for small sample bias. The third differs from the other two by standardizing by the control group standard deviation rather than an average standard deviation across both groups. The standardised mean difference assumes that differences in the standard deviation between studies reflect differences in the measurement scale and not differences between the study populations. The summary intervention effect can be difficult to interpret as it is presented in abstract units of standard deviation rather than any particular scale.

Note that in social science meta-analyses, the term 'effect size' usually refers to versions of the standardised mean difference.

Time-to-event outcomes

Time-to-event analysis takes account not only of whether an event happens but when it happens. This is especially important in chronic diseases where even although we may not be able to ultimately stop an event from happening, slowing its occurrence can be beneficial. For example, in cancer studies in adult patients we rarely anticipate cure, but hope that we can significantly prolong survival. Time-to-event data are often referred to as 'survival' data since death is often the event of interest, but can be used for many different types of event such as time free of seizures, time to healing or time to conception. Each study participant has data capturing the event status and the time of that status. An individual may be recorded with a particular elapsed time-to-event, or they may be recorded as not having experienced the event by a particular elapsed time or period of follow-up. When the event has not (yet) been observed, the individual is described as censored, and their event-free time contributes information to the analysis up until the point of censoring.

The most appropriate way to analyse time-to-event data is usually to use Kaplan Meier analysis and express results as a hazard ratio (HR). The HR summarises the entire

survival experience and describes the overall likelihood of a participant experiencing an event on the experimental intervention compared to control. Meta-analyses that collect individual participant data are able to carry out such analysis for each included study and then pool these using a variant of the Peto method described above. Alternatively a modelling approach can be used.

Meta-analyses of aggregate data often treat time-to-event data as dichotomous and carry out analyses using the numbers of individuals who did or did not experience an event by a particular point in time. However, using such dichotomous measures in a meta-analysis of time-to-event outcomes is discarding information and can pose additional problems. If the total number of events reported for each study is used to calculate an odds ratio or risk ratio, this can involve combining studies reported at different stages of maturity, with variable follow-up, resulting in an estimate that is both unreliable and difficult to interpret. This approach is not recommended. Alternatively, ORs or RRs can be calculated at specific points in time. Although this makes estimates comparable, interpretation can still be difficult, particularly if individual studies contribute data at different time points. In this case it is unclear whether any observed difference in effect between time points is attributable to the timing or to the analyses being based on different sets of contributing studies. Furthermore, bias could arise if the time points are subjectively chosen by the researcher or selectively reported by the study author at times of maximal or minimal difference between intervention groups.

A preferable approach is to estimate HRs by using and manipulating published or other summary statistical data or survival curves.[147, 148] This approach has also been described in non-technical step-by-step terms.[149] Currently, such methods are under-used in meta-analyses,[149] which may reflect unfamiliarity with the methods and that study reports do not always include the necessary statistical information[150, 151] to allow the methods to be used.

Ordinal outcomes

Outcomes may be presented as ordinal scales, such as pain scales (where individuals' rate their pain as none, mild moderate or severe). These are sometimes analysed as continuous data, with each category being assigned a numerical value (for example, 0 for none, 1 for mild, 2 for moderate and 3 for severe). This is usual when there are many categories, as is the case for many psychometric scales such as the Hamilton depression scale or the Mini-Mental State Examination for measuring cognition. However, a mean value may not be meaningful. Thus, an alternative way to analyse ordinal data is to dichotomise them (e.g. none or mild versus moderate or severe) to produce a standard 2×2 table. Methods are available for analysing ordinal data directly, but these typically require expert input.

Counts and rates

When outcomes can be experienced repeatedly they are usually expressed as event counts, for example, the number of asthma attacks. When these represent common events, they are often treated and analysed as continuous data (for example, number of days in hospital) and where they represent uncommon events they are often dichotomised (for example, whether or not each individual had *at least one* stroke).

When events are rare, analyses usually focus on rates expressed at the group level, such as the number of asthma attacks per person, per month. Although these can be combined as rate ratios using the generic inverse variance method, this is not always appropriate as it assumes a constant risk over time and over individuals, and is not often done in practice. It is important not to treat rate data as dichotomous data because more than one event may have arisen from the same individual.

Presentation of quantitative results

Results should be expressed in formats that are easily understood, and in both relative and absolute terms.

Where possible, results should be shown graphically. The most commonly used graphic is the forest plot (see *Box 1.7*), which illustrates the effect estimates from individual studies and the overall summary estimate. It also gives a good visual summary of the review findings, allowing researchers and readers to get a sense of the data. Forest plots provide a simple representation of the precision of individual and overall results and of the variation between-study results. They give an 'at a glance' identification of any studies with outlying or unusual results and can also indicate whether particular studies are driving the overall results. Forest plots can be used to illustrate results for dichotomous, continuous and time-to-event outcomes.[152]

Individual study results are shown as boxes centred on their estimate of effect, with extending horizontal lines indicating their confidence intervals. The confidence interval expresses the uncertainty around the point estimate, describing a range of values within which it is reasonably certain that the true effect lies; wider confidence intervals reflect greater uncertainty. Although intervals can be reported for any level of confidence, in most systematic reviews of health interventions, the 95% confidence interval is used. Thus, on the forest plot, studies with wide horizontal lines represent studies with more uncertain results. Different sized boxes may be plotted for each of the individual studies, the area of the box representing the weight that the study takes in the analysis providing a visual representation of the relative contribution that each study makes to the overall effect.

The plot shows a vertical line of equivalence indicating the value where there is no difference between groups. For odds ratios, risk ratios or hazard ratios this line will be drawn at an odds ratio/risk ratio/hazard ratio value of 1.0, while for risk difference and mean difference it will be drawn through zero. Studies reach conventional levels of statistical significance where their confidence intervals do not cross the vertical line. Summary (meta-analytic) results are usually presented as diamonds whose extremities show the confidence interval for the summary estimate. A summary estimate reaches conventional levels of statistical significance if these extremities do not cross the line of no effect. If individual studies are too dissimilar to calculate an overall summary estimate of effect, a forest plot that omits the summary value and diamond can be produced.

Odds ratios, risk ratios and hazard ratios can be plotted on a log-scale to introduce symmetry to the plot. The plot should also incorporate the extracted numerical data for the groups for each study, e.g. the number of events and number of individuals for odds ratios, the mean and standard deviation for continuous outcomes. Other forms of graphical displays have also been proposed.[153]

Box 1.7: Effects of four trials included in a systematic review

a) Presented without meta-analysis

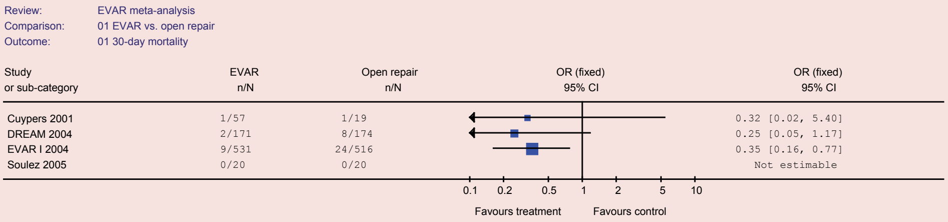

b) Presented with meta-analysis (fixed effect model)

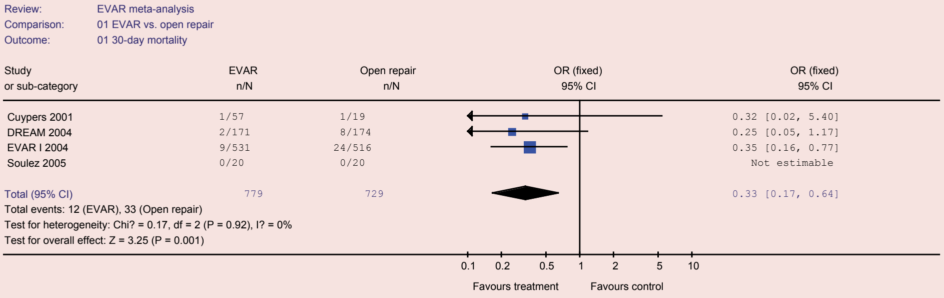

c) Presented with meta-analysis (random-effects model)

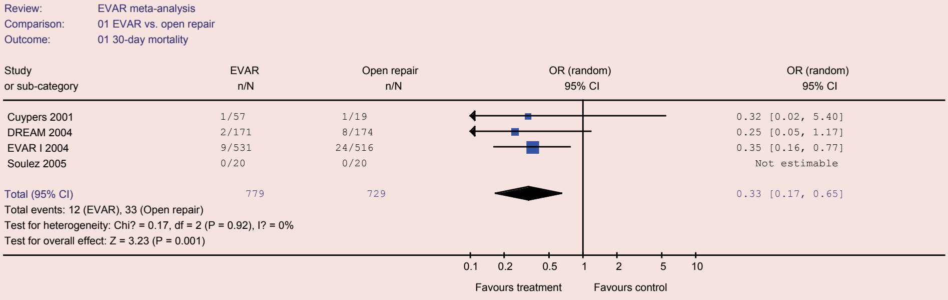

Example forest plots taken from a systematic review of endovascular stents for abdominal aortic aneurism (EVAR).[154]

Relative and absolute effects

Risk ratios, odds ratios and hazard ratios describe relative effects of one intervention versus another, providing a measure of the overall chance of the event occurring on the experimental intervention compared to control. These relative effects do not provide information on what this comparison means in absolute terms. Although there may be a large relative effect of an intervention, if the absolute risk is small, it may not be clinically significant because the change in absolute terms is minimal (a big percentage of a small amount may still be a small amount). For example, a risk ratio of 0.8 may represent a 20% relative reduction in events from 50% to 40% or it could represent a 20% relative reduction from 5% to 4% corresponding to absolute differences of 10% and 1% respectively. There may be situations where the former is judged to be clinically significant whilst the latter is not. Meta-analysis should use ratio measures; for example, dichotomous data should be combined as risk ratios or odds ratios and pooling risk differences should be avoided. However, when reporting results it is generally useful to convert relative effects to absolute effects. This can be expressed as either an absolute difference or as a number needed to treat (NNT). Absolute change is usually expressed as an absolute risk reduction which can be calculated from the underlying risk of experiencing an event if no intervention were given and the observed relative effect as shown in *Box 1.8*.

Box 1.8: Calculation of absolute risk reduction and number needed to treat from relative risks, odds ratios and hazard ratios

Absolute risk reduction from relative risk

$$\text{ARR} = (\text{RR} - 1)\,\text{Risk}_{\text{baseline}}$$

Absolute risk reduction from odds ratio[155]

$$\text{ARR} = \frac{(1 - \text{Risk}_{\text{baseline}})\,(1 - \text{OR})}{\text{Risk}_{\text{baseline}} + \text{OR}\,(1 - \text{Risk}_{\text{baseline}})} \times \text{Risk}_{\text{baseline}}$$

Absolute risk reduction from hazard ratio[156]

$$\text{ARR} = S_{\text{control}}{}^{\text{HR}} - S_{\text{control}} \text{ at chosen time point}$$

Number needed to treat

$$\text{NNT} = \frac{1}{\text{ARR}}$$

Where:

RR = relative risk

ARR = absolute risk reduction

S_{control} = proportion event free on control treatment

HR = hazard ratio

Consideration of absolute effects is particularly important when considering how results apply to different types of individuals who may have different underlying prognoses and associated risks. Even if there is no evidence that the relative effects of an intervention vary across different types of individual (see *Subgroup analyses* and *Meta-regression* below), if the underlying risks for different categories of individual differ, then the effect of intervention in absolute terms will be different. It is therefore important when reporting results to consider how the absolute effect of an intervention varies for different types of individual and a table expressing results in this way, as shown in *Table 1.5*, can be useful. The underlying risk for different types of individual can be estimated from the studies included in the meta-analysis, or generally accepted standard estimates can be used. Confidence intervals should be calculated around absolute effects.

Table 1.5: Example table expressing relative effects as absolute effects for individuals with differing underlying prognoses.

			2 year survival rate	
**HR = 0.84<br>95% CI (0·78–0·92)**		**Baseline**	**Absolute increase (95% CI)**	**Change**
Age	<=40	50%	5% (3% – 8%)	From 50 to 55%
	41-59	14%	5% (2% – 8%)	From 14 to 19%
	>=60	4%	2% (1% – 4%)	From 4 to 6%
Histology	AA	31%	6% (3% – 9%)	From 31 to 37%
	GBM	9%	4% (2% – 6%)	From 9 to 13%
	Other	52%	5% (3% – 8%)	From 52 to 57%
Performance Status	Good	22%	6% (3% – 9%)	From 22 to 28%
	Poor	9%	4% (2% – 6%)	From 9 to 13%

Baseline survival and equivalent absolute increases in survival calculated from a meta-analysis of chemotherapy in high-grade glioma.[157]
AA = anaplastic astrocytoma, GBM = glioblastoma multiforme.

The NNT, which is derived from the absolute risk reduction as shown in *Box 1.8*, also depends on both relative effect and the underlying risk. The NNT represents the number of individuals who need to be treated to prevent one event that would be experienced on the control intervention. The lower the number needed to treat, the fewer the patients that need to be treated to prevent one event, and the greater the efficacy of the treatment. For example a meta-analysis of antiplatelet agents for the prevention of pre-eclampsia found an RR of 0.90 (0.84 – 0.97) for pre-eclampsia.[158] Plausible underlying risks of 2%, 6% and 18% had associated NNTs of 500 (313-1667), 167 (104-556) and 56 (35-185) respectively.

Sensitivity analyses

Sensitivity analyses explore the robustness of the main meta-analysis results by repeating the analyses having made some changes to the data or methods.[159] Analyses run with and without the inclusion of certain trials will assess the degree to which

particular studies (perhaps those with poorer methodology) affect the results. For example, analyses might be carried out on all eligible trials and a sensitivity analysis restricted to only those that used a placebo in the control group. If results differ substantially, the final results will require careful interpretation. However care must be taken in attributing reasons for differences, especially when a single or small numbers of trials are included/excluded in the sensitivity analysis, as a study may differ in additional ways to the issue being explored in the sensitivity analysis. Some sensitivity analyses should be proposed in the protocol, but as many issues suitable for exploration in sensitivity analyses only come to light whilst the review is being done, and in response to decisions made or difficulties encountered, these may have to change and/or be supplemented.

Exploring heterogeneity

There will inevitably be variation in the observed estimates of effect from the studies included in a meta-analysis. Some of this variation arises by chance alone, reflecting the fact that no study is so large that random error can be removed entirely. Statistical heterogeneity refers to variation other than that which arises by chance. It reflects methodological or clinical differences between studies. Exploring statistical heterogeneity in a meta-analysis aims to tease out the factors contributing to differences, such that sources of heterogeneity can be accounted for and taken into consideration when interpreting results and drawing conclusions.

There is inevitably a degree of clinical diversity between the studies included in a review,[160] for example because of differing patient characteristics and differences in interventions. If these factors influence the estimated intervention effect then there will be some statistical heterogeneity between studies. Methodological differences that influence the observed intervention effect will also lead to statistical heterogeneity. For example, combining results from blinded and unblinded studies may lead to statistical heterogeneity, indicating that they might best be analysed separately rather than in combination. Although it manifests itself in the same way, heterogeneity arising from clinical differences is likely to be because of differences in the true intervention effect, whereas heterogeneity arising from differences in methodology is more likely to be because of bias.

An idea of heterogeneity can be obtained straightforwardly by visually examining forest plots for variations in effects. If there is poor overlap between the study confidence intervals, then this generally indicates statistical heterogeneity.

More formally a χ^2 (chi-squared) test (see *Box 1.9*), often also referred to as *Q*-statistic, can assess whether differences between results are compatible with chance alone. However, care must be taken in interpreting the chi-squared test as it has low power, consequently a larger P value ($P<0.1$) is sometimes used to designate statistical significance. Although a statistically significant test result may point to a problem with heterogeneity, a nonsignificant test result does not preclude important between-study differences, and cannot be taken as evidence of no heterogeneity. Conversely, if there are many studies in a meta-analysis, the test has high power to detect a small amount of heterogeneity that, although statistically significant, may not be clinically important.

Accepting that diversity is likely to be inherent in any review, methods have also been developed to quantify the degree of inconsistency across studies, shifting the focus from significance testing to quantifying heterogeneity. The I^2 statistic[160, 161] describes the percentage of variability in the effect estimates that can be attributed to heterogeneity rather than chance (see *Box 1.9*).

Box 1.9: Chi-squared test (or *Q*-statistic) and test for interaction

Chi-squared test:

$$Q = \sum \frac{1}{SE_i^{\,2}} \left(EE_i - EE_{pooled} \right)^2$$

$$I^2 = \left[\frac{Q - df}{Q} \right] \times 100\%$$

Where Q is the chi-squared statistic, and df its degrees of freedom.

To examine differences across subgroups, either Q or I^2 can be applied to meta-analytic results from each subgroup rather than to individual studies (i.e. the sum in Q is across subgroups rather than across studies).

Although the I^2 statistic often has wide confidence intervals and it is difficult to provide hard and fast rules on what level of inconsistency is reasonable in a meta-analysis, as a rough guide it has been suggested that I^2 values of up to 40% might be unimportant, 30% to 60% might be moderate, 50 to 90% may be substantial and 75% to 100% considerable.[75]

If statistical heterogeneity is observed, then the possible reasons for differences should be explored[162] and a decision made about if and how it is appropriate to combine studies. A systematic review does not always need to include a meta-analysis and, if there are substantial differences between study estimates of effect, particularly if they are in opposing directions, combining results in a meta-analysis can be misleading. One way of addressing this is to split studies into less heterogeneous groups according to particular study level characteristics (e.g. by type of drug), and perform separate analyses for each group. Forest plots can be produced to show subsets of studies on the same plot. Each subset of studies can have its own summary estimate, and if appropriate an overall estimate combined across all studies can also be shown. Showing these groupings alongside each other in this way provides a good visual summary of how they compare. This approach allows the consistency and inconsistency between subsets of studies to be examined. Differences can be summarised narratively, but where possible they should also be evaluated formally. A χ^2 test for differences across subgroups can be carried out (see *Box 1.9*).

The influence of patient-level characteristics (e.g. age, gender) or issues related to equity (e.g. ethnicity, socioeconomic group) can also be explored through subgroup analyses, meta-regression or other modelling approaches. However, there is generally insufficient information in published study reports to allow full exploration of heterogeneity in this way and this can usually only be addressed satisfactorily when IPD are available. Such exploration of heterogeneity may enable additional questions to be addressed, such as which particular treatments perform best or which types of patient will benefit most, but is unlikely to be helpful when there are few studies. Wherever possible, potential sources of heterogeneity should be considered when writing the review protocol and possible subgroup analyses pre-specified rather than trying to explain statistical heterogeneity after the fact.

Subgroup analyses

Subgroup analyses divide studies (for study level characteristics) or participant data (for participant level characteristics) into subgroups and make indirect comparisons between them. These analyses may be carried out to explore heterogeneity (see above) as well as to try to answer particular questions about patient or study factors. For example a subgroup analysis for study level characteristics might examine whether the results of trials carried out in primary health care settings are the same as trials carried out in a hospital setting. A participant level subgroup analysis might examine whether the effect of the intervention is the same in men as in women.

In individual studies it is unusual to have sufficient numbers and statistical power to permit reliable subgroup analyses of patient characteristics. However, provided that such data have been collected uniformly across studies, a meta-analysis may achieve sufficient power in each subgroup to permit a more reliable exploration of whether the effect of an intervention is larger (or smaller) for any particular type of individual. Although, owing to the multiplicity of testing, these analyses are still potentially misleading, subgroup analysis within the context of a large meta-analysis may be the only reasonable way of performing such exploratory investigations. Not only do the greater numbers give increased statistical power, but consistency across trials can be investigated. Indeed, the possibility of undertaking such analyses is a major attraction of IPD meta-analyses as dividing participant data into groups for subgroup analysis is seldom possible in standard reviews of aggregate data.[163] Subgroup analyses in most (non IPD) systematic reviews focus on grouping according to trial attributes.

The interpretation of the results of subgroup analyses must be treated with some caution. Even where the original data have come from RCTs, the investigation of between-study differences is indirect and equivalent to an observational study.[164, 165] There may be explanations for the observed differences between groups, other than the attributes chosen to categorise groupings. Comparisons which are planned in advance on the basis of a plausible hypothesis and written into the protocol are more credible than findings that are found through *post hoc* exploratory analyses. Furthermore, the likelihood of finding false negative and false positive significance tests rises rapidly as more subgroup analyses are done. Subgroups should therefore be restricted to a few potentially important characteristics where it is reasonable to suspect that the characteristic will interact with or modify the effect of the intervention. Note that there is often confusion between prognostic factors and potential effect modifiers; just because a characteristic is

prognostic does not mean that it will modify the effect of an intervention. For example, whilst gender is prognostic for survival (women live longer than men) it does not necessarily mean that women will benefit more than men will from a drug to treat lung cancer.

Meta-regression

Meta-regression can be used to investigate the effects of differences in study characteristics on the estimates of the treatment effect,[140] and can explore continuous as well as categorical characteristics. In principle it can allow for the simultaneous exploration of several characteristics and their interactions, though in practice this is seldom possible because of small numbers of studies.[166] As in any simple regression analysis, meta-regression aims to predict outcome according to explanatory variables or covariates of interest. The covariates may be constant for the entire trial, for example, the protocol dose of a drug, or a summary measure of attributes describing the patient population, for example, mean age or percentage of males. The regression is weighted by precision of study estimates such that larger studies have more influence than smaller studies. The regression coefficient is tested to establish whether there is an association between the intervention effect and the covariate of interest. Provided that enough data are available (at least 10 studies),[82] the technique may be a useful exploratory tool. However, there are limitations. Not all publications will report on all the covariates of interest (and there could be potential bias associated with selective presentation of data that have shown a positive association within a primary study). If a study is missing a covariate it drops out of the regression, limiting the power and usefulness of the analysis, which is already likely to be based on relatively few data points.

Meta-regression is not a good way to explore differences in treatment effects between different types of individual as summary data may misrepresent individual participants.[167] What is true of a study with a median participant age of 60 may not necessarily be true for a 60-year-old patient. Potentially all the benefit could have been shown in the 50-year-olds and none in the 60 and 70-year-olds. Comparison of treatment effects between different types of individual, for example between men and women, should be done using subgroup analyses and not by using meta-regression incorporating the proportion of women in each trial. It should always be borne in mind that finding a significant association in a meta-regression does not prove causality and should rather be regarded as hypothesis generating.

Assessing the possibility of publication bias

Although thorough searches should ensure that a systematic review captures as many relevant studies as possible, they cannot eliminate the risk of publication bias. As publication and associated biases can potentially influence profoundly the findings of a review, the risk of such bias should be considered in the review's conclusions and inferences.[24] The book by Rothstein et al provides a comprehensive discussion of publication bias and associated issues.[168]

The obvious way to test for publication bias is to compare formally the results of published and unpublished studies. However, more often than not unpublished studies are hidden from the reviewer, and more ad hoc methods are required. A common technique to help assess potential publication bias is the funnel plot.

This is a scatter plot based on the fact that precision in estimating effect increases with increasing sample size. Effect size is plotted against some measure of study precision – of which standard error is likely to be the best choice.[169] A wide scatter in results of small studies, with the spread narrowing as the trial size increases, is expected. If there is no difference between the results of small and large studies, the shape of the plot should resemble an inverted funnel (see *Box 1.10*). If there are differences, the plot will be skewed and a gap where the small unfavourable studies ought to be is often cited as evidence of publication bias. However, the shape of a funnel plot can also depend on the measures selected for estimating effect and precision[169, 170] and could be attributable to differences between small and large studies other than publication bias. These differences could be a result of other types of methodological bias, or genuine clinical differences. For example, small studies may have a more selected participant population where a larger treatment effect might be expected. Funnel plots are therefore more accurately described as a tool for investigating small study effects.

Box 1.10: Example funnel plots from a systematic review of dressings and topical agents used in the healing of chronic wounds[183]

Symmetrical

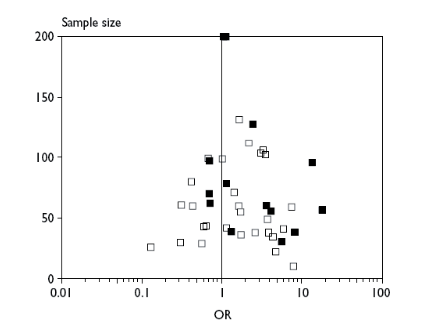

Asymmetrical

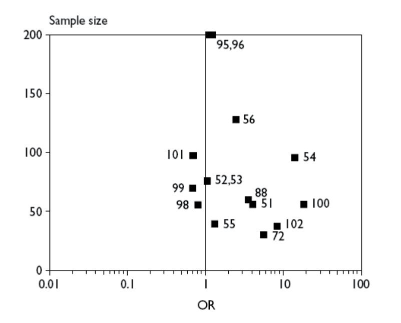

☐ Traditional vs. dressing/topical agent other than hydrocolloid

■ Traditional vs. hydrocolloid dressing only

This funnel plot, of all the studies that compared traditional treatments with modern dressing or topical agents for the treatment of leg ulcers and pressure sores, showed little evidence of asymmetry.

This funnel plot, of trials that compared traditional treatments with hydrocolloid dressings for the treatment of leg ulcers and pressure sores, showed clear asymmetry. This was considered likely to be the result of publication bias.

Although visual inspection of funnel plots has been shown to be unreliable,[170, 171] this might be improved if contour zones illustrating conventional levels of significance are overlaid on the plot to illustrate whether 'missing' studies are from zones of statistical significance or not. If the 'missing' studies are from nonsignificant zones, this may support a publication bias. On the other hand if 'missing' studies are from statistically significant zones, the asymmetry may be more likely to be attributable to other causes.[172] Over time a range of statistical and modelling methods have been developed to test for asymmetry, the most frequently used of which are those based on rank correlation[173] or linear regression[174, 175] and complex modelling[176] methods. Some methods (for example, the trim and fill method[177, 178]) attempt to adjust for any publication bias detected.[176] However, all methods are by nature indirect and the appropriateness of many methods is based on some strict assumptions that can be difficult to justify in practice.

Although frequently used to help assess possible publication bias, funnel plots and associated statistical tests are often used and interpreted inappropriately,[179, 180] potentially giving false assurance where a symmetrical plot overlooks important bias or undermining important valid evidence because of an asymmetric plot.[179] The methods are inappropriate where there is statistical heterogeneity; have low power and are of little use where there are few studies; and are meaningless where studies are of similar size. Consequently, situations where they are helpful are few and their use is not generally a good way of dealing with publication bias.[181] Therefore use of these methods to identify or adjust for publication bias in a meta-analysis should be considered carefully and generally be restricted to sensitivity analyses. Results should be interpreted with caution. Statistical tests will not resolve bias and avoidance of publication bias is preferable. In time this may become easier with more widespread registration of clinical trials and other studies at inception.[182]

Dealing with special study designs and analysis issues

Intention to treat analyses

ITT is usually the preferred type of analysis as it is less likely to introduce bias than alternative approaches. True intention to treat analysis captures two criteria: (i) participants should be analysed irrespective of whether or not they received their allocated intervention and irrespective of what occurred subsequently, for example, participants with protocol violations or those subsequently judged ineligible should be included in the analysis; (ii) all participants should be included irrespective of whether outcomes were collected. Although the first criterion is generally accepted, there is no clear consensus on the second[81] as it involves including participants in the analyses whose outcomes are unknown, and therefore requires imputation of data. Many authors describe their analyses as ITT when only the first criterion has been met. Alternative analysis of all participants for whom outcome data are available is termed available case analysis. Some studies present analysis of all participants who completed their allocated treatment, this is per protocol or treatment received analysis which may be seriously biased.

Imputing missing data

Although statistical techniques are available to impute missing data, this cannot reliably compensate for missing data[184] and in most situations imputation of data is not recommended. It is reasonable for most systematic reviews to aim for an available case analysis and include data from only those participants whose outcome is known. Achieving this may require making contact with the study author if individuals for whom outcome data were recorded have been excluded from the published analyses. The extent and implications of missing data should always be reported and discussed in the review. If the number of participants missing from the final analysis is large it will be helpful to detail the reasons for their exclusion.

In some circumstances, it might be informative to impute data in sensitivity analyses to explore the impact of missing data.[185] For missing dichotomous data the analysis can assume that either all participants with missing data experienced the event, or that they all did not experience the event. This generates the theoretical extremes of possible effect. Data could also be imputed using the rate of events observed in the control group, however this does not add information, gives inflated precision and is not recommended. Where missing data are few, imputation will have little impact on the results. Where missing data are substantial, analysis of worst/best case scenarios will give a wide range of possible effect sizes and may not be particularly helpful. Approaches to imputing missing continuous data have received little attention. In some cases it may be possible to use last observation carried forward, or to assume that no change took place. However, this cannot be done from aggregate data and the value of such analysis is unclear. Any researcher contemplating imputing missing data should consult with an experienced statistician.

Cluster randomised trials

In cluster randomised trials, groups rather than individuals are randomised, for example clinical practices or geographical areas. Reasons for allocating interventions in this way include evaluating policy interventions or group effects such as in immunisation programmes, and avoiding cross-contamination, for example, health promotion information may be easily shared by members of the same clinic or community. In many instances clustering will be obvious, for example where primary care practices are allocated to receive a particular intervention. In other situations the clustering may be less obvious, for example where multiple body parts on the same individual are allocated treatments or where a pregnant woman has more than one fetus. It is important that any cluster randomised trials are identified as such in the review.

As participants within any one cluster are likely to respond in a manner more similar to each other than to other individuals (owing to shared environmental exposure or personal interactions), their data cannot be assumed to be independent. It is therefore inappropriate to ignore the clustering and analyse as though allocation had been at the individual level. This unit of analysis error would result in overly narrow confidence intervals and straightforward inclusion of trials analysed in this way would give undue weight to that study in a meta-analysis. Unfortunately, many primary studies have ignored clustering and analysed results as though from an individual randomised trial.[186, 187] One way to avoid the problem of inappropriately analysed cluster trials is to carry out meta-

analyses using a summary measure for each cluster as a single observation. The sample size becomes the number of clusters (not the number of individuals) and the analysis then proceeds as normal. However, depending on the size and number of clusters, this will reduce the statistical power of the analysis considerably and unnecessarily. Indeed the information required to do this is unlikely to be available in many study publications.

A better approach is to adjust the results of inappropriately analysed primary studies to take account of the clustering, by increasing the standard error of the estimate of effect.[75] This may be achieved by multiplying the original standard error by the square root of the 'design effect'. The design effect can be calculated from the intracluster correlation coefficient, which, although seldom reported, can use external values from similar studies such as those available from the University of Aberdeen Health Services Research Unit (www.abdn.ac.uk/hsru/epp/iccs-web.xls). A common design effect is usually adopted across the intervention groups.

$$DE = 1 + \left[\left(M - 1\right) \times ICC\right]$$

$$SE_{adjusted} = SE\sqrt{DE}$$

where:
DE = design effect
M = mean cluster size
ICC = intracluster correlation coefficient
SE = standard error of the effect estimate

These values can then be used in a generic inverse variance meta-analysis alongside unadjusted values from appropriately analysed trials.

Cross-over trials

Cross-over trials allocate each individual to a sequence of interventions, for example one group may be allocated to receive treatment A followed by treatment B, and the other group allocated to receive B followed by A. This type of trial has the advantage that each participant acts as their own control, eliminating between participant variability such that fewer participants are required to obtain the same statistical power. They are suitable for evaluating interventions that have temporary effects in treating stable conditions. They are not appropriate where an intervention can have a lasting effect that compromises treatment in subsequent periods of the trial, or where a condition has rapid evolution, or the primary outcome is irreversible. The first task of the researcher is to decide whether the cross-over design is appropriate in assessing the review question.

Appropriate analysis of cross-over trials involves paired analysis, for example using a paired t-test to analyse a study with two interventions and two periods (using experimental measurement – control measurement) for each participant, with standard errors calculated for these paired measurements. These values can then be combined in a generic inverse variance meta-analysis. Unfortunately, cross-over trials are frequently inappropriately analysed and reported.

A common naive analysis of cross-over data is to treat all measurements on experimental and control interventions as if they were from a standard parallel group trial. This results in confidence intervals that are too wide and the trial receives too little weight in the meta-analysis. However, as this is a conservative approach, it might not be unreasonable in some circumstances. Where the effect of the first intervention is thought to have influenced the outcome in subsequent periods (carry-over), a common approach is to use only the data from the first period for each individual. However, this will be biased if the decision to analyse in this way is based on a test of carry-over and studies analysed in this way may differ from those using paired analyses. One approach to combining studies with differing types of reported analyses is to carry out an analysis grouped by type of study i.e. grouped by cross-over trial paired analysis, cross-over trial with first period analysis, parallel group trial, and explore whether their results differ (see *Subgroup analyses* above).

Alternatively, the researcher can carry out their own paired analysis for each trial if (i) the mean and standard deviation or standard error of participant differences are available; (ii) the mean difference plus a t-statistic, p-value or confidence interval from a paired analysis is available; (iii) a graph from which individual matched measurements can be extracted; or (iv) if individual participant data are available.[188] Another approach is to attempt to approximate a paired analysis by imputing missing standard errors by 'borrowing' from other studies that have used the same measurement scale or by a correlation coefficient obtained from other studies or external sources.[75] Researchers will need to decide whether excluding trials is preferable to inferring data. If imputation is thought to be reasonable, advice should be sought from an experienced statistician. Authors should state explicitly where studies have used a cross-over design and how this has been handled in the meta-analysis.

Mixed treatment comparisons

Mixed treatment comparisons (MTC), or network meta-analyses, are used to analyse studies with multiple intervention groups and to synthesise evidence across a series of studies in which different interventions were compared. These are used to rank or identify the optimal intervention. They build a network of evidence that includes both direct evidence from head to head studies and indirect comparisons whereby interventions that have not been compared directly are linked through common comparators. A framework has been described that outlines some of the circumstances in which such syntheses might be considered.[189] Methods for conducting indirect comparisons[190, 191] and more complex mixed treatment methods[192, 193] require expert advice. Researchers wishing to undertake such analyses should consult with an appropriately experienced statistician.

Bayesian methods

Unlike standard analysis techniques, Bayesian analyses allow for the combination of existing information with new evidence using established rules of probability.[194] A simple Bayesian analysis model includes three key elements:

1. Existing knowledge on the effect of an intervention can be retrieved from a variety of sources and summarised as a prior distribution
2. The data from the studies are used to form the likelihood function

3. The prior distribution and the likelihood function are formally combined to provide a posterior distribution which represents the updated knowledge about the effect of the intervention

Bayesian approaches to meta-analysis may be useful when evidence comes from a diverse range of sources particularly when few data from RCTs exist.[195, 196] They can also be used to account for the uncertainty introduced by estimating the between-study variance in the random-effects model, which can lead to reliable estimates and predictions of treatment effects.[197] While there are several good texts available,[198-200] if a Bayesian approach is to be used, the advice of a statistical expert is strongly recommended.

Describing results

When describing review findings, the results of all analyses should be considered as a whole, and overall coherence discussed. Consistency across studies should be considered and results interpreted in relation to biological and clinical plausibility. Where there have been many analyses and tests, care should be taken in interpreting unexpected or implausible findings as among a large number of tests the play of chance alone is likely to generate spurious statistically significant results.

Quantitative results of meta-analyses should be expressed as point estimates together with associated confidence intervals and exact p-values. They should not be presented or discussed only in terms of statistical significance. This is particularly important where results are not statistically significant as nonsignificance can arise both when estimates are close to no effect with narrow confidence intervals, or when estimates of effect are large with wide confidence intervals. Whilst in the former, we can be confident that there is little difference between the interventions compared, in the latter there is insufficient evidence to draw conclusions. Researchers should be aware that describing a result as 'there is no statistical (or statistically significant) difference between the two interventions' can be (mis)read as there being no difference between interventions.

It is important that inconclusive results are not interpreted as indicating that an intervention is ineffective and estimates with wide confidence intervals that span no effect should be described as showing no clear evidence of a benefit or harm rather than as there being no difference between interventions. Demonstrating lack of sufficient evidence to reach a clear conclusion is an important finding in its own right.

Similarly, care should be taken not to overplay results that are statistically significant, as with large enough numbers, even very modest differences between interventions can be statistically significant. The size of the estimated effect, and its confidence intervals, should be considered in view of how this relates to current or future practice (see *Section 1.3.6 Report writing*).

It is usually helpful to present findings in both relative and absolute terms and in particular to consider how relative effects may translate into different absolute effects for people with differing underlying prognoses (see *Relative and absolute effects* section above). Where a number of outcomes or subgroup analyses are included in a review it can be helpful to tabulate the main findings in terms of effect, confidence intervals and inconsistency or heterogeneity statistics.

Summary: Data synthesis

- Synthesis involves bringing the results of individual studies together and summarising their findings.
- This may be done quantitatively or, if formal pooling of results is inappropriate, through a narrative approach.
- Synthesis should also explore whether observed intervention effects are consistent across studies, and investigate possible reasons for any inconsistencies.

Initial descriptive synthesis

All syntheses should begin by constructing a clear descriptive summary of the included studies.

Narrative synthesis is frequently an essential part of a systematic review, and as with every other stage of the process, bias must be minimized.

Narrative synthesis has typically not followed a strict set of rules. However, a general framework can be applied in order to help maintain transparency and add credibility to the process. The four elements of this framework are:

- Developing a theory of how the intervention works, why and for whom
- Developing a preliminary synthesis of findings of included studies
- Exploring relationships within and between studies
- Assessing the robustness of the synthesis

Each element contains a range of tools and techniques that can be applied. A researcher is likely to move iteratively among the four elements, choosing those tools and techniques that are appropriate to the data being synthesised and providing justifications for these choices.

Quantitative synthesis

- Meta-analysis increases power and precision in estimating intervention effects.
- Results of individual studies are combined statistically to give a pooled estimate of the 'average' intervention effect.
- Most meta-analysis methods are based on calculating a weighted average of the effect estimates from each study.
- The methods used to combine results will depend on the type of outcome assessed.
- Quantitative results should be expressed as point estimates together with associated confidence intervals and exact p-values.
- Variation in results across studies should be investigated.
- Sensitivity analyses give an indication of the robustness of results to the type of study included and the methods used.

1.3.6 Report writing

Report writing is an integral part of the systematic review process. This section deals with the primary scientific report of the review which often takes the form of a comprehensive report to the commissioning body. Many commissioners have their own guidance for production and submission of the report. Alternatively the primary report may take the form of a journal article, where space limitations may mean that important details of the review methods have to be omitted. These can be made available through the journal's or the review team's website. Whatever the format, it is important to take as much care over report preparation as over the review itself. The report should describe the review methods clearly and in sufficient detail that others could, if they wished, repeat them. There is evidence that the quality of reporting in reports of primary studies may affect the readers' interpretation of the results, and the same is likely to be true of systematic reviews.[201] It has also been argued that trials and reviews often provide incomplete or omit the crucial 'how to' details about interventions, limiting a clinicians' ability to implement findings in practice.[202-204]

The QUOROM statement[9] has set standards for how reviews incorporating meta-analysis should be reported, and many journals require articles submitted to adhere to these standards. The QUOROM checklist and flow chart are useful resources for all authors of systematic review reports. However, recognising that the quality of reporting of many systematic reviews is disappointing,[205] the QUOROM group have broadened their remit, been renamed PRISMA (Preferred Reporting Items for Systematic Reviews and Meta-Analyses),[206] and developed a flow chart and checklist for the reporting of systematic reviews with or without a meta-analysis.[66, 67]

1.3.6.1 General considerations

Resources for writers

There are many resources for writers available in both printed and electronic form. These include guides to technical writing and publishing,[207-209] style manuals[210, 211] and guides to use of English.[212] The EQUATOR Network is an initiative that seeks to improve the quality of scientific publications by promoting transparent and accurate reporting of health research.[101] It provides an introduction to reporting guidelines, and information for authors of research reports, editors and peer reviewers as well as those developing reporting guidelines.

Style and structure

Commissioning bodies and journals usually have specific requirements regarding presentation and layout that should be followed when preparing a report or article. Some organisations offer detailed guidance while others are less specific. In the absence of guidance, a layered approach such as a one page summary of the research 'actionable messages', three-page executive summary and a 25-page report is advocated as the optimal way to present research evidence to health service managers and policy-makers.[213] *Box 1.11* presents a suggested outline structure for a typical report of a systematic review.

Many journals publish papers electronically ahead of print publication and electronic publishing often allows additional material, such as large tables, or search strategies to be made available through the journal's website. There is no specific word limit for reports published in electronic format only, for example in the Cochrane Library, although Cochrane reviews 'should be as succinct as possible'.[75]

Box 1.11: Suggested structure of a systematic review report

Title
Contents list
Abbreviations/glossary

Executive summary or structured abstract
Background
Objectives
Methods (data sources, study selection, data extraction, quality assessment, data synthesis)
Results
Conclusions

Main text
Background/introduction
Review question(s)
Review methods
- *Identification of studies*
- *Study selection (inclusion and exclusion criteria; methods)*
- *Data extraction*
- *Quality assessment*
- *Data synthesis*

Results of the review
- *Details of included and excluded studies*
- *Findings of the review*
- *Secondary analyses (sensitivity analyses etc.)*

Discussion (interpretation of the results)
Conclusions
- Recommendations/implications for practice/policy
- Recommendations/implications for further research

Acknowledgements or list of contributors and contributions
Funding
Conflicts of interest
References
Appendices

Researchers should familiarise themselves with the conventions favoured by their commissioning body or 'target' journal. Many journals now prefer a clear and active style that is understandable to a general audience. Weaknesses in the use of grammar and spelling constitute obstacles to clear communication and should be eliminated as far as possible. The field of scientific and technical communication predominantly uses English as its common language, so those who are unsure of their ability in written English may find it helpful to have their report checked by an accomplished speaker/writer who is familiar with the subject matter before submission.

Contents lists and headings are essential for guiding the reader through longer documents. Inclusion of an index may also be helpful. It is particularly important to adopt a consistent style (e.g. font, point size, font style) for different levels of main headings and sub-headings.

Planning

Time spent preparing a brief outline covering the main points to be included in the report can save time overall. The outline should focus on who the intended audience is and what they need to know. The review team will need to agree the outline and, if the report is to be written by multiple authors, allocate writers for each section. Dividing the work amongst a number of people reduces the burden on each individual but there is a risk of loss of consistency in style and terminology. In addition, completion of the report relies on all the team members working to the agreed schedule. It is essential for the lead author (corresponding author for journal articles) to monitor progress and take responsibility for accuracy and consistency.

Authorship and contributorship

The report of a systematic review will usually have a number of authors. According to the International Committee of Medical Journal Editors (ICMJE),[214] authorship credit should be based on:

1. Substantial contributions to conception and design, or acquisition of data, or analysis and interpretation of data
2. Drafting the article or revising it critically for important intellectual content; and
3. Final approval of the version to be published

All authors should meet all of these conditions. The review team should agree amongst themselves who will be authors and the order of authorship. Order of authorship is often taken to reflect an individual's contribution to the report and methods are available for scoring contributions to determine authorship.[215] Alternatively authors can simply be listed alphabetically. Contributions that do not meet the criteria for authorship (for example, data extraction or membership of an advisory group) should be included in the acknowledgements.

Some journals, for example the BMJ, favour a system of contributorship.[216] In addition to the standard list of authors, there is a list of all those who contributed to the paper with details of their contributions. One contributor (occasionally more than one) is listed as guarantor and accepts overall responsibility for the work. This system gives some credit to those who do not meet the ICMJE criteria for authorship and provides accountability for each stage of the review.

Peer review and feedback

Most systematic reviews have an expert advisory group assembled at the beginning of the project and members of this group should be asked to review the draft report and comment on its scientific quality and completeness. The commissioning body may also organise its own independent peer review of the draft report before publication.

Medical journals almost invariably seek external peer review of manuscripts submitted for publication. Draft manuscripts may also be posted on institutional websites or electronic preprint servers, allowing an opportunity for feedback from a wide range of interested parties, although for reports intended for journals it is important to ensure that such posting will not be considered as prior publication.

In addition to scientific peer review, end users may also be asked to assess the relevance and potential usefulness of the review. They may recommend changes that would help in identifying the main messages for dissemination and important target audiences as well as possible formats and approaches.

When feedback from external reviewers has been received, a final report can be prepared. A record of the comments and the way in which they were dealt with should be kept with the archive of the review.

Conflict of interests

The ICMJE state that a conflict of interests exists if 'an author (or the author's institution), reviewer, or editor has financial or personal relationships that inappropriately influence (bias) his or her actions'.[214] Relationships that might constitute a conflict of interests are common and there is nothing wrong with having such relationships. However, it is important that they are declared so that readers are aware of the possibility that authors' judgements may have been influenced by other factors. Review authors need to be explicit about any potential conflict of interests because such transparency is important in maintaining the readers' confidence.

1.3.6.2 Executive summary or abstract

The executive summary (for full-length reports) or abstract (for journal articles) is the most important part of the report because potentially it is the only section that many readers will actually read (perhaps in conjunction with the discussion and conclusions). It should present the findings of the review clearly and concisely and allow readers to quickly judge the quality of the review and the generalisability of its findings. Providing a good balance between detail of the intervention and how the review was conducted, and the results and conclusions is always a challenge, and may require several iterations across the whole review team. The summary is usually the last section to be written so that full consideration can be given to all relevant aspects of the project. However the process of summary writing may help in the further development of the recommendations by forcing review teams to identify the one or two most important findings and the conclusions which flow from them. It should be remembered that revisions to the report or article following peer review may also need to be reflected in the summary. Assistance from outside parties and medical writers may be helpful in developing a good summary.

1.3.6.3 Formulating the discussion

The purpose of the discussion section of a report is to help readers to interpret the results of the review. This should be done by presenting an analysis of the findings and outlining the strengths and weaknesses of the review. The discussion should also place the findings in the context of the existing evidence base, particularly in relation to any existing relevant reviews. It has been suggested that more could and should be done in discussion sections to contextualise both the nature of the research and the findings to the existing evidence base.[217] There should be a balance between objectively describing the results, and subjectively speculating on their meaning.[218] It is important to present a clear and logical train of thought and reasoning, supported by the findings of the review and other existing knowledge. For example although statistically significant results and clear evidence of effectiveness may have been demonstrated, without an exploration of the impact on clinical practice it may not be clear whether they are clinically significant. Information on the interpretation of the analysis is given throughout *Section 1.3.5 Data synthesis.*

Some commissioners and most journals have a set format or structure for the report. This may require the discussion section to incorporate the conclusions and any implications or recommendations, or may require these as separate sections. In the absence of a structured format for the discussion section, the framework given in *Box 1.12* may be helpful.

Box 1.12: Framework for the discussion section of a review

Statement of principal findings

Strengths and weaknesses of the review

Appraisal of methodological quality of the review
Relation to other reviews, in particular considering any differences

Meaning of the review's findings

Strengths and weaknesses of the evidence included in the review
Direction and magnitude of effects observed in the included studies
Applicability of the findings of the review

Implications

Practical implications for clinicians and policy-makers
Unanswered questions and implications for further research

Based on Docherty and Smith (1999)[219]

1.3.6.4 Conclusions, implications, recommendations

Faced with the need to make decisions and limited time to read the whole report, many readers may go directly to the conclusions. Therefore, whether incorporated in the discussion section or presented separately, it is essential that the conclusions be clearly

worded and based solely on the evidence reviewed. The conclusions should summarise the evidence and draw out the implications for health care, and preferably be worded to show how they have been derived from the evidence.

Conclusions are generally a standard requirement, however, many commissioners and journals have their own conventions about implications and recommendations. For example, the NIHR HTA programme require the conclusions section of reports to include the implications for health care and specify recommendations for future research, in order of priority. They specifically exclude making recommendations for policy or clinical practice.[220] Authors' conclusions from Cochrane reviews are presented as the implications for practice and research; recommendations are not made.[130]

In the absence of guidance from the commissioner, it is generally advisable to avoid making recommendations about policy or practice, unless this is the focus of the review. The nature of the review question should therefore guide whether it is appropriate to include recommendations or focus on the implications for policy, practice and/or further research, and how these are best presented. Whether recommendations are made or implications drawn, it is important to ensure that these are supported by the evidence and to avoid making any statements that are outside the defined scope of the review. The way in which a recommendation or implication is phrased can considerably influence the way in which it is interpreted and implemented (or ignored). Hence, it is important to make all statements as precise as possible.[221-223]

Recommendations for practice are usually only made in guidelines, and are formulated from a variety of sources of information in addition to review findings. There are a number of schemes available for grading practice recommendations according to the strength of the evidence that supports them.[224-230] Systematic review reports should aim to provide the information required to implement any of these systems if used. It should be noted that not all the schemes take into account the generalisability of the findings of the review to routine clinical practice. This should always be a consideration when drawing up the implications or if making recommendations.

A clear statement of the implications or recommendations for future research should be made; vague statements along the lines of 'more research is needed' are not helpful and should be avoided. Specific gaps in the evidence should be highlighted to identify the research questions that need answering. Where methodological issues have been identified in existing studies, suggestions for future approaches may be made. Where possible, research recommendations should be listed in order of priority, and an indication of how rapidly the knowledge base in the area is developing should be included. This can assist in planning an update of the review and help guide commissioners when allocating funding.

The DUETs initiative (Database of Uncertainties about the Effects of Treatments; (www.duets.nhs.uk), recommends the presentation of research recommendations in a structured format represented by the acronym EPICOT (Evidence, Population(s), Intervention(s), Comparison(s), Outcome(s), Time stamp). Timeliness (duration of intervention/follow-up), disease burden and suggested study design are considered as optional additional elements of a structured research recommendation. Further details and an example of how to formulate research recommendations using the EPICOT format can be found in an article published by the DUETS Working Group.[231] It is worth

noting that there is some debate about the applicability of the EPICOT format for some reviews, particularly those of complex interventions.[232]

Summary: Report writing

- Report writing is an integral part of the systematic review process.
- Reviews may be published as a report for the commissioner, as a journal article or both. Researchers should be aware of the requirements of commissioning bodies and journals and adhere to them.
- Readability is a key aspect of reporting; a review's findings will not be acted on if they are not clearly presented and understood.
- The executive summary (for full-length reports) or abstract (for journal articles) is the most important part of the report, because it is potentially the only section that many readers will actually read (perhaps in conjunction with the discussion and conclusions).
- A structured framework can be helpful for preparing the discussion section of the report.
- Implications for practice or policy and recommendations for further research should be based solely on the evidence contained in the review.
- The findings from systematic reviews are frequently used to inform guideline development. Guideline recommendations are often formulated using a grading scheme. Systematic review reports should therefore aim to provide the information required for such grading schemes.
- A structured format for the presentation of research recommendations has been developed as a result of the DUETS initiative.

1.3.7 Archiving the review

There are published guidelines relating to the retention of primary research data.[233] While these do not currently relate to systematic reviews, they do represent appropriate good practice. Where policies on retention, storage and protection are not specified by a commissioner, researchers might consider including this information in research proposals so that it is clear from the outset what will be kept and for how long.

Decisions need to be made about which documents are vital to keep and which can be safely disposed of. Extracted data and quality assessment information should be preserved. In addition, records of decisions made during protocol development, inclusion screening and data extraction, are unique and should be kept. Minutes of meetings, correspondence as well as peer review comments and responses might also be held for a specific period of time as further records of the decision-making process. It is always advisable to permanently store a copy of the final report, particularly if the only other copy in existence is the one submitted to the commissioners.

Some information used in the review such as conference abstracts, additional information from authors, and unpublished material may be particularly difficult to obtain at a later stage so hard copies should be archived. This also applies to material retrieved from the Internet, which should be printed for the archive, as links to web pages are not permanent.

Whilst it may be easy and space saving to archive material electronically, paper records are often preferable as the equipment used to access documents stored in electronic formats can become obsolete after a relatively short period of time.

1.3.8 Disseminating the findings of systematic reviews

In recent years, there has been substantial investment in the commissioning of systematic reviews assessing the effects of a range of different health care interventions. To improve the quality of health care, and ultimately health outcomes, the review findings need to be effectively communicated to practitioners and policy-makers. The transfer of knowledge obtained through research into practice has long been acknowledged as a complex process[234-238] that is highly dependent on context and the interaction of a multitude of interconnected factors operating at the level of the individual, group, organisation and wider health system.

A number of conceptual frameworks have attempted to represent the complexity of knowledge translation processes.[234, 236, 238-244] One recent framework,[244] whilst recognising the importance of non-linear diffusion, highlights a pivotal role for the direct or planned dissemination of contextualised, actionable messages derived from systematic reviews to inform practice and policy decision-making processes.

CRD's experience of direct dissemination has led to the development of a framework, which is supported by both theoretical and empirical research into the ways by which different audiences become aware of, receive, access, read and use research findings (*Figure 1.4*). This involves targeting the right people with a clear and relevant message, communicating via appropriate and often multiple channels (any medium used to convey a message to an audience or audiences), whilst taking account of the environment in which the message will be received.

Detailed information about this framework is provided here; case studies showing the framework in use can be found on the CRD website (www.york.ac.uk/inst/crd). The framework provides a basic structure that enables researchers to consider carefully the appropriateness of their plans for dissemination, simple or complex, and could be used by anyone seeking to promote the findings of a review.

1.3.8.1 What is dissemination?

As interest in enhancing the impact of health research has increased, so too has the terminology used to describe the approaches employed.[241, 245] Terms like dissemination, diffusion, implementation, knowledge transfer, knowledge mobilisation, linkage and exchange and research into practice are all being used to describe overlapping and interrelated concepts and practices. Given this, it is helpful to explain how the term dissemination is used here.

Dissemination is a planned and active process that seeks to ensure that those who need to know about a piece of research get to know about it and can make sense of the findings. As such it involves more than making research accessible through the traditional mediums of academic journals and conference presentations. It requires forethought about the groups who need to know the answer to the question a review is addressing, the best way of getting the message directly to that audience, and doing so by design rather than chance. Hence an active rather than passive process.

The term dissemination is often used interchangeably with implementation but it is more appropriate to see the terms as complementary. Dissemination and implementation are

part of a continuum.[239, 246, 247] At one end are activities that focus on making research accessible, raising awareness of new findings and encouraging consideration of practice alternatives and policy options. At the other end of the continuum are activities that seek to increase the adoption of research findings into practice and policy and that facilitate, reinforce and maintain changes in practice.

CRD's primary focus is very much at the awareness raising end of the continuum, though there is no clear cut off point, and there is evidence for the positive effects of planned dissemination on the implementation of research evidence in practice.[237] For example, there is some evidence that the centre's *Effective Health Care* and *Effectiveness Matters* series of bulletins had a positive impact on the quality of health care delivered. Empirical studies have suggested that the dissemination of these bulletins contributed to reductions in the prophylactic extraction of wisdom teeth,[248, 249] in the use of surgical interventions for glue ear,[250, 251] and impacted on the prescribing of selective serotonin reuptake inhibitors for depression.[252, 253]

Dissemination should not be viewed as an adjunct to the review process or as something to be considered at the end when thoughts turn to publication. Nor should it be seen as separate from the wider social context in which the review findings are expected to be used. It is an integral part of the review process and should be considered from an early stage to allow adequate time for planning and development, for the allocation of responsibilities and to ensure that the proposed activities are properly resourced. The CRD framework (*Figure 1.4*) offers a sequential approach to considering, developing and implementing appropriate dissemination strategies for individual systematic reviews. The framework has been utilised for a wide range of topics and audiences for over a decade.

1.3.8.2 CRD's approach to dissemination

Traditionally, research on dissemination and implementation has tended to focus on the use of research knowledge, rather than on the effects of dissemination activities. However, a number of conceptual frameworks have been put forward which consistently suggest that the effectiveness of dissemination activities is determined by careful consideration of a number of key attributes.[234, 237, 254-258] These are:

- The characteristics of the research message
- The setting in which the message is received
- The characteristics of the target audience(s)
- The source of the research message
- The presentation of the research message
- The communication channel(s) used

Assuming that all research has an audience (but not that all research should be widely disseminated), whether the message provides an unequivocal answer or simply highlights the need for further research, our approach is structured around six key attributes which are interlinked and difficult to consider in isolation (see *Figure 1.4*). The key messages from the review are the starting point for determining the audience to be targeted.

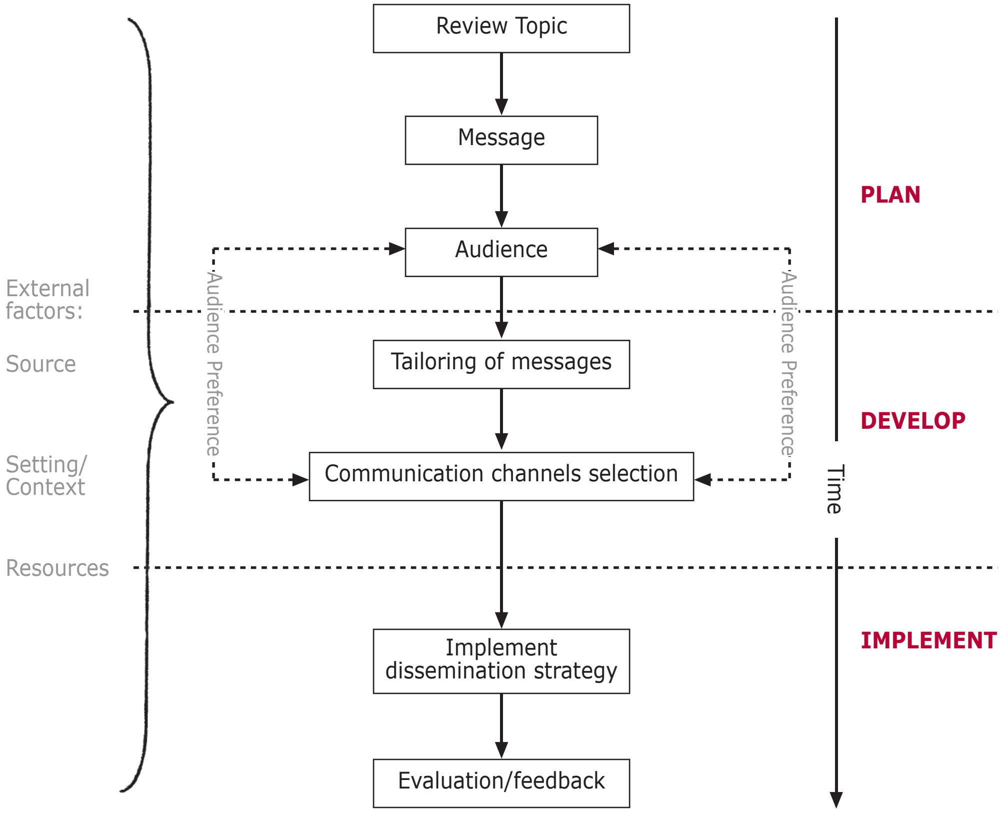

Figure 1.4: CRD Dissemination framework

Characteristics of the research message and the setting in which it will be received

The literature on communication[259] and diffusion[239] (i.e. how, why, and at what rate ideas/innovations spread through social systems) highlights three types of messages that can impact on the knowledge and attitudes of target audiences: awareness, instruction ('how to') and persuasion (information that reduces uncertainty about expected consequences). Message characteristics to consider include the nature of the intervention, the strength of the evidence, its transferability, the degree of uncertainty and whether the findings confirm or reject existing predispositions or practices. Messages also have to be perceived as relevant and meaningful by the audiences being targeted. Knowledge about both the wider setting (economic, social, organisational and political environments) within which a target audience resides and the context (hostile or receptive) in which a message is to be received, should be used to inform the development of appropriate dissemination strategies.

Characteristics of the target audience(s)

Deciding who to target usually involves an element of prioritisation (segmentation) as resource constraints can make it difficult to reach all possible audiences. In prioritising, relevance (who needs to know about this research) and receptivity (who is most likely to be influenced and to influence others) need to be considered. The question of how best to reach target audiences can in part be answered by drawing upon the theoretical literature on research utilisation (the ways by which different audiences become aware of, access, read and make use of research findings).[260, 261] This literature helps to inform the selection of the most appropriate or feasible communication channels for the audiences being targeted. Channels frequently used to promote review findings include paper and electronic publishing, email alerting services, direct and relationship marketing, mass media campaigns as well as engaging directly with target audiences.

Presentation of the research message and communication channel(s) used

The literature on diffusion[239] makes a distinction between mass media channels and interpersonal (face to face) channels. The former are generally regarded as being more important for dissemination purposes whereas interpersonal channels are more important for activity at the implementation end of the continuum. CRDs experience is that a combination of communication channels is helpful in increasing the likelihood that target audiences will encounter the review messages being promoted.

The selection of communication channels may also inform the presentation (tailoring) of the research message itself. When tailoring messages, consideration is given to the target audience, language used, the format, structure and style of presentation, the types of appeal and the amount of repetition. It is generally appropriate to try to write for an educated but non-research specialist health professional or decision-maker. Lay terms are used rather than technical language and statistics presented in as simple a form as possible. The aim is to make information accessible to a broad range of readers and anyone who would like more details can access the full report. It has been advocated that a layered structure such as the '1:3:25' format (i.e. one page of the research 'bottom lines' or 'actionable messages', three-page executive summary and a 25-page report) is the optimal way to present research evidence to health service managers and policy-makers.[213] This type of structuring involving a front page of key messages has become common place and reflects documented audience preferences for the 'bottom line' up front. There is some evidence that this order of presentation can increase overall understanding of the research findings but may also in some instances alienate those who are less receptive to or in disagreement with the conclusions presented.[262, 263]

Source of the research message

How the source (i.e. the research team or organisation) is perceived by a target audience in terms of its credibility (trustworthiness), attractiveness (likemindedness) or power, is an important consideration. For example, where the evidence base is contested (clinically or politically), and/or where audiences are less familiar with systematic review methods generally, promoting source credibility can be crucial from the outset. An approach CRD has used when encountering these issues, has been to

create dedicated, publicly accessible websites that provide information about all aspects of the review. These websites enable external scrutiny of the review process, and include feedback facilities for interested parties to comment, ask questions or submit evidence for consideration. Our experience suggests it is important to make it clear that contributions of existing research evidence, including published/grey literature, are welcome, but that personal experience and anecdote, whilst important, does not usually form part of a systematic review. An example of a review dedicated website can be found at www.york.ac.uk/inst/crd/fluorid.htm. Considerable effort is required to set up, monitor and maintain a dedicated website and our experience of the benefit is varied. It is important therefore to consider the likely benefit to the review and the target audience before setting up a site.

Dissemination strategies

It has been proposed that there are four dissemination models that can be employed to link 'research to action'.[262, 263] These are:

- Push strategies which are largely associated with supply (researcher) led distribution of new research findings
- Pull strategies which facilitate demand (audience) led access to research
- Linkage and exchange[264] efforts which involve two way communications and partnerships between producers and users of research
- Integrated approaches that incorporate aspects of all three

In reality, push, pull and exchange strategies are not mutually exclusive; facilitating user pull often requires the application of a promotional push strategy (e.g. utilising email alerting services or RSS feeds) to inform and remind target audiences about review findings that are forthcoming or have been made available online for example. CRD favours the integrated approach that incorporates elements of all three strategies, but where the emphasis shifts according to the topic and the audiences to be targeted.

Evaluation of impact

There is an increasing requirement, particularly from funders, for the impact of research to be predicted in advance of the work and then assessed after completion.[265, 266] There are a number of specialised research impact assessment approaches, but these usually require specialist skills and additional resources.[267, 268] Taking the issue of whether academic quality or practical use and impact of research is most important, a pragmatic framework has been proposed which addresses both points.[269] The framework is based on the assessment criteria used in UK universities. It provides a structure for a narrative description of the impact of the findings from why the research question was first posed and funded, to where the results were sent, discussed, and put into policy and/or practice.

Summary: Dissemination

- Simply making research available does not ensure that those that need to know about it get to know about it or can make sense of the findings.
- Dissemination is a planned and active process that can aid the transfer of research into practice.
- Dissemination should not be viewed as an adjunct but rather as an integral part of the review process and should be considered from the outset.
- CRD employs a topic-driven approach that involves targeting the right people with understandable and relevant messages, communicating via appropriate (often multiple) channels, whilst taking account of the environment in which the message will be received.
- The CRD framework provides a basic structure for developing appropriate dissemination strategies and could be used by anyone seeking to promote the findings of a review.

REFERENCES

1. Centre for Reviews and Dissemination. *DARE (Database of Abstracts of Reviews of Effects) [internet]*. York: Centre for Reviews and Dissemination, University of York; [cited 2008 9 Sep]. Available from: www.crd.york.ac.uk/crdweb/

2. *The Cochrane Library, Issue 2, 2008 [internet]*. Chichester: Wiley; [cited 2008 Apr 17]. Available from: www3.interscience.wiley.com/cgi-bin/mrwhome/106568753/HOME

3. *The Campbell Collaboration [internet]*. The Campbell Collaboration; c2008. [cited 2008 Apr 17]. Available from: www.campbellcollaboration.org/frontend.aspx

4. *EPPI-Centre [internet]*. London: The Evidence for Policy and Practice Information, Social Science Research Unit, Institute of Education, University of London; c2008. [cited 2008 Apr 17]. Available from: http://eppi.ioe.ac.uk/cms/

5. *National Guidelines Clearinghouse [internet]*. Agency for Healthcare Research and Quality (AHRQ), U.S. Department of Health and Human Services; c2008. [cited 2008 Apr 17]. Available from: http://guidelines.gov/

6. *Scottish Intercollegiate Guidelines Network (SIGN) [internet]*. Edinburgh: Scottish Intercollegiate Guidelines Network; c2008. [cited 2008 Apr 17]. Available from: www.sign.ac.uk/index.html

7. Greenhalgh T. How to read a paper: papers that summarise other papers (systematic reviews and meta-analyses). *BMJ* 1997;315:672-5.

8. Oxman AD, Guyatt GH. Guidelines for reading literature reviews. *CMAJ* 1988;138:697-703.

9. Moher D, Cook DJ, Eastwood S, Olkin I, Rennie D, Stroup DF. Improving the quality of reports of meta-analyses of randomised controlled trials: the QUOROM statement. *Lancet* 1999;354:1896-900.

10. Oxman AD, Cook DJ, Guyatt GH. User's guide to the medical literature. VI. How to use an overview. *JAMA* 1994;272:1367-71.

11. Moher D, Tsertsvadze A, Tricco A, Eccles M, Grimshaw J, Sampson M, et al. When and how to update systematic reviews. *Cochrane Database of Systematic Reviews* 2008, Issue 1. Art. No.: MR000023. DOI: 10.1002/14651858.MR000023.pub3.

12. Lumley J, Oliver SS, Chamberlain C, Oakley L. Interventions for promoting smoking cessation during pregnancy. *Cochrane Database of Systematic Reviews* 2004, Issue 4. Art. No.: CD001055. DOI: 10.1002/14651858.CD001055.pub2.

13. NHS Centre for Reviews and Dissemination. *A systematic review of public water fluoridation.* CRD Report 18. York: University of York; 2000.

14. McDaid C, Hartley S, Bagnall AM, Ritchie G, Light K, Riemsma R. Systematic review of effectiveness of different treatments for childhood retinoblastoma. *Health Technol Assess* 2005;9:1-145.

15. Gøtzsche PC, Liberati A, Torri V, Rossetti L. Beware of surrogate outcome measures. *Int J Technol Assess Health Care* 1996;12:238-46.

16. Sackett DL, Wennberg JE. Choosing the best research design for each question. *BMJ* 1997;315:1636.

17. Horwitz RI. Large-scale randomized evidence: large, simple trials and overviews of trials: discussion. A clinician's perspective on meta-analyses. *J Clin Epidemiol* 1995;48:41-4.

18. Eysenck HJ. Meta-analysis and its problems. *BMJ* 1994;309:789-92.

19. Egger M, Zellweger-Zähner T, Schneider M, Junker C, Lengeler C, Antes G. Language bias in randomised controlled trials published in English and German. *Lancet* 1997;350:326-9.

20. Vickers A, Goyal N, Harland R, Rees R. Do certain countries produce only positive results? A systematic review of controlled trials. *Control Clin Trials* 1998;19:159-66.

21. Grégoire G, Derderian F, Le Lorier J. Selecting the language of the publications included in a meta-analysis: is there a Tower of Babel bias? *J Clin Epidemiol* 1995;48:159-63.

22. Moher D, Pham B, Klassen TP, Schulz KF, Berlin JA, Jadad AR, et al. What contributions do languages other than English make on the results of meta-analyses? *J Clin Epidemiol* 2000;53:964-72.

23. Moher D, Pham B, Lawson ML, Klassen TP. The inclusion of reports of randomised trials published in languages other than English in systematic reviews. *Health Technol Assess* 2003;7:1-90.

24. Song F, Eastwood AJ, Gilbody S, Duley L, Sutton AJ. Publication and related biases. *Health Technol Assess* 2000;4:1-115.

25. Dundar Y, Dodd S, Dickson R, Walley T, Haycox A, Williamson PR. Comparison of conference abstracts and presentations with full-text articles in the health technology assessments of rapidly evolving technologies. *Health Technol Assess* 2006;10:1-145.

26. Chokkalingam A, Scherer R, Dickersin K. Concordance of data between conference abstracts and full reports [abstract]. In: *6th Cochrane Colloquium*; 1998 Oct 22-26; Baltimore, MD, USA.

27. Tooher R, Middleton P, Griffin T, Pham C, Hopewell S. How different are conference abstracts of surgical RCTs from the subsequent full publication? [abstract]. In: *12th Cochrane Colloquium*; 2004 Oct 2-6; Ottawa, Ontario, Canada.

28. Dundar Y, Dodd S, Williamson P, Dickson R, Walley T. Case study of the comparison of data from conference abstracts and full-text articles in health technology assessment of rapidly evolving technologies: does it make a difference? *Int J Technol Assess Health Care* 2006;22:288-94.

29. Song FJ, Fry-Smith A, Davenport C, Bayliss S, Adi Y, Wilson JS, et al. Identification and assessment of ongoing trials in health technology assessment reviews. *Health Technol Assess* 2004;8:1-87.

30. Silagy CA, Middleton P, Hopewell S. Publishing protocols of systematic reviews: comparing what was done to what was planned. *JAMA* 2002;287:2831-4.

31. Thomson Scientific. *BIOSIS [database]*. Thomson Scientific; 2006. [cited 2006 31 Aug]. Available from: www.biosis.org/

32. Thomson Scientific. *Index to Scientific & Technical Proceedings [database]*. Thomson Scientific; 2006. [cited 2006 31 Aug]. Available from: http://scientific.thomson.com/products/istp/

33. British Library. *ZETOC [internet]*. Mimas, University of Manchester; 2006. [cited 2006 31 Aug]. Available from: http://zetoc.mimas.ac.uk/

34. CSA. *Conference Papers Index [database]*. Bethesda, MD: CSA; 2006. [cited 2006 31 Aug]. Available from: www.csa.com/factsheets/cpi-set-c.php

35. Chokkalingam A, Scherer R, Dickersin K. Agreement of data in abstracts compared to full publications. *Control Clin Trials* 1998;19:61S-62S.

36. Hopewell S, Eisinga A, Clarke M. Better reporting of randomized trials in biomedical journal and conference abstracts. *Journal of Information Science* 2008;34:162-73.

37. Loria A, Arroyo P. Language and country preponderance trends in MEDLINE and its causes. *J Med Libr Assoc* 2005;93:381-5.

38. *Cochrane Central Register of Controlled Trials (CENTRAL) [internet]*. Chichester: John Wiley & Sons Ltd; [cited 2008 05 Aug]. Available from: www.mrw.interscience.wiley.com/cochrane/cochrane_clcentral_articles_fs.html

39. Hopewell S, Clarke M, Lefebvre C, Scherer R. Handsearching versus electronic searching to identify reports of randomized trials. *Cochrane Database of Systematic Reviews* 2007, Issue 2. Art. No.: MR000001. DOI: 10.1002/14651858.MR000001.pub2.

40. Ogilvie D, Hamilton V, Egan M, Petticrew M. Systematic reviews of health effects of social interventions: 1. Finding the evidence: how far should you go? *J Epidemiol Community Health* 2005;59:804-8.

41. Bambra C, Whitehead M, Hamilton V. Does 'welfare-to-work' work? A systematic review of the effectiveness of the UK's welfare-to-work programmes for people with a disability or chronic illness. *Soc Sci Med* 2005;60:1905-18.

42. Lefebvre C, Manheimer E, Glanville J. Chapter 6: Searching for studies. In: Higgins JPT, Green S, editors. *Cochrane handbook for systematic reviews of interventions. Version 5.0.0 (updated February 2008)*: The Cochrane Collaboration; 2008. Available from: www.cochrane-handbook.org

43. National Centre for Text Mining (NaCTeM). *ASSERT project webpage [internet]*. 2008. [cited 21 Aug 2008]. Available from: www.nactem.ac.uk/assert/

44. Ananiadou S, Procter R, Rea B, Sasaki Y, Thomas J. Supporting systematic reviews using text mining. In: *3rd International Conference on e-Social Science*; Oct 7-9; Ann Arbor, MI. 2007.

45. National Centre for Text Mining. *Text mining briefing paper*. Bristol: Joint Information Systems Committee (JISC); 2006.

46. Ananiadou S. The National Centre for Text Mining: a vision for the future. *Ariadne* 2007;53.

47. Okazaki N, Ananiadou S. Building an abbreviation dictionary using a term recognition approach. *Bioinformatics* 2006;22:3089-95.

48. *KLEIO [internet]*. National Centre for Text Mining (NaCTeM), School of Computer Science, The University of Manchester; [cited 2008 10 Sep]. Available from: www.nactem.ac.uk/software/kleio/index.jsp

49. *FACTA [internet]*. National Centre for Text Mining (NaCTeM), School of Computer Science, The University of Manchester; [cited 2008 8 Sep]. Available from: http://text0.mib.man.ac.uk/software/facta/

50. Thomas J, Brunton J. *EPPI-Reviewer 3.0: analysis and management of data for research synthesis. EPPI-Centre Software*. London: EPPI-Centre, Social Science Research Unit, Institute of Education; 2006.

51. Mead TL, Richards DT. Librarian participation in meta-analysis projects. *Bull Med Libr Assoc* 1995;83:461-64.

52. Slavin RE. Best evidence synthesis: an intelligent alternative to meta-analysis. *J Clin Epidemiol* 1995;48:9-18.

53. Oxman AD, Stachenko SJ. Meta-analysis in primary care: theory and practice. In: Tudiver F, Bass MJ, Dunn EV, Norton PG, editors. *Assessing interventions: traditional and innovative research methods for primary care*. Newbury Park, CA: Sage Publications; 1992. p. 191-207.

54. Goodman C. Step 2: specify inclusion criteria for studies. In: *Literature searching and evidence interpretation for accessing health care practices*. Stockholm: Swedish Council on Technology Assessment in Health Care; 1993.

55. Oxman AD, Guyatt GH. The science of reviewing research. *Ann N Y Acad Sci* 1993;703:125-33.

56. Cooper H, Ribble RG. Influences on the outcome of literature searches for integrative research reviews. *Knowledge* 1989;10:179-201.

57. Edwards P, Clarke M, DiGuiseppi C, Pratap S, Roberts I, Wentz R. Identification of randomized controlled trials in systematic reviews: accuracy and reliability of screening records. *Stat Med* 2002;21:1635-40.

58. Altman DG. Measuring agreement. In: Altman DG, editor. *Practical statistics for medical research*. London: Chapman and Hall; 1991.

59. McDonagh MS, Whiting PF, Wilson PM, Sutton AJ, Chestnutt I, Cooper J, et al. Systematic review of water fluoridation. *BMJ* 2000;321:855-59.

60. Chalmers TC, Levin H, Sacks HS, Reitman D, Berrier J, Nagalingam R. Meta-analysis of clinical trials as a scientific discipline. I: control of bias and comparison with large co-operative trials. *Stat Med* 1987;6:315-25.

61. Berlin JA, University of Pennsylvania Meta-analysis Blinding Study Group. Does blinding of readers affect the results of meta-analyses? *Lancet* 1997;350:185-86.

62. Haines T, Kennedy C, Gross A, Goldsmith C, Hondras M, Roving I, et al. Influence of 'blinding' on selection of articles for relevance [abstract]. In: *7th Cochrane Colloquium:* 1999 Oct 5-9; Rome, Italy.

63. von Elm E, Poglia G, Walder B, Tramer MR. Different patterns of duplicate publication: an analysis of articles used in systematic reviews. *JAMA* 2004;291:974-80.

64. von Elm E, Tramer MR, Jüni P, Egger M. Does duplicate publication of trials introduce bias in systematic reviews? A systematic review [abstract]. In: *11th Cochrane Colloquium:* 2003 Oct 26-31; Barcelona, Spain.

65. Tramer MR, Reynolds DJ, Moore RA, McQuay HJ. Impact of covert duplicate publication on meta-analysis: a case study. *BMJ* 1997;315:635-40.

66. Moher D, Liberati A, Tetzlaff J, Altman DG, and the PRISMA Group. Preferred Reporting Items for Systematic reviews and Meta-Analyses: the PRISMA Statement [forthcoming].

67. Liberati A, Altman DG, Tetzlaff J, Mulrow C, Gøtzsche P, Ioannidis JPA, et al. The PRISMA Statement for reporting systematic reviews and meta-analyses of studies that evaluate health care interventions: explanation and elaboration [forthcoming].

68. Higgins JPT, Deeks JJ, (editors). Chapter 7: Selecting studies and collecting data. In: Higgins JPT, Green S, editors. *Cochrane handbook for systematic reviews of interventions. Version 5.0.0 (updated February 2008)*: The Cochrane Collaboration; 2008. Available from: www.cochrane-handbook.org

69. Buscemi N, Hartling L, Vandermeer B, Tjosvold L, Klassen TP. Single data extraction generated more errors than double data extraction in systematic reviews. *J Clin Epidemiol* 2006;59:697-703.

70. Jadad AR, Moore RA, Carroll D, Jenkinson C, Reynolds DJM, Gavaghan DJ, et al. Assessing the quality of reports of randomized clinical trials: is blinding necessary? *Control Clin Trials* 1996;17:1-12.

71. Sacks HS, Berrier J, Reitman D, Acona-Berk VA, Chalmers TC. Meta-analysis of randomized controlled trials. *N Engl J Med* 1987;316:450-5.

72. Stewart LA, Tierney JF, Clarke M. Chapter 19: Reviews of individual patient data. In: Higgins JPT, Green S, editors. *Cochrane handbook for systematic reviews of interventions. Version 5.0.0 (updated February 2008)*: The Cochrane Collaboration; 2008. Available from: www.cochrane-handbook.org

73. Egger M, Davey Smith G, Altman DG. *Systematic reviews in health care: meta analysis in context*. 2nd ed. London: BMJ Books; 2001.

74. Petticrew M, Roberts H. *Systematic reviews in the social sciences: a practical guide*. Malden, MA: Blackwell Publishing; 2006.

75. Higgins JPT, Green S, (editors). *Cochrane handbook for systematic reviews of interventions. Version 5.0.0 [updated February 2008]*: The Cochrane Collaboration; 2008. Available from: www.cochrane-handbook.org

76. Verhagen AP, de Vet HC, de Bie RA, Kessels AG, Boers M, Bouter LM, et al. The Delphi list: a criteria list for quality assessment of randomized clinical trials for conducting systematic reviews developed by Delphi consensus. *J Clin Epidemiol* 1998;51:1235-41.

77. Devereaux PJ, Manns BJ, Ghali WA, Quan H, Lacchetti C, Montori VM, et al. Physician interpretations and textbook definitions of blinding terminology in randomized controlled trials. *JAMA* 2001;285:2000-3.

78. Montori VM, Bhandari M, Devereaux PJ, Manns BJ, Ghali WA, Guyatt GH. In the dark: the reporting of blinding status in randomized controlled trials. *J Clin Epidemiol* 2002;55:787-90.

79. Wright CC, Sim J. Intention-to-treat approach to data from randomized controlled trials: a sensitivity analysis. *J Clin Epidemiol* 2003;56:833-42.

80. Tierney JF, Stewart LA. Investigating patient exclusion bias in meta-analysis. *Int J Epidemiol* 2005;34:79-87.

81. Hollis S, Campbell F. What is meant by intention to treat analysis? Survey of published randomised controlled trials. *BMJ* 1999;319:670-4.

82. Higgins JPT, Altman DG, on behalf of the Cochrane Statistical Methods Group and the Cochrane Bias Methods Group. Chapter 8: Assessing risk of bias in included studies. In: Higgins JPT, Green S, editors. *Cochrane handbook for systematic reviews of interventions. Version 5.0.0 (updated February 2008)*: The Cochrane Collaboration; 2008. Available from: www.cochrane-handbook.org

83. Senn S. *Cross-over trials in clinical research*. 2nd ed. Chichester: John Wiley & Sons; 2002.

84. Donner A, Klar N. *Design and analysis of cluster randomization*. London: Arnold; 2000.

85. Puffer S, Torgerson DJ, Watson J. Evidence for risk of bias in cluster randomised trials: review of recent trials published in three general medical journals. *BMJ* 2003;327:785-9.

86. Hahn S, Puffer S, Torgerson DJ, Watson J. Methodological bias in cluster randomised trials. *BMC Med Res Methodol* 2005;5:10.

87. Campbell MK, Elbourne DR, Altman DG, Consort group. CONSORT statement: extension to cluster randomised trials. *BMJ* 2004;328:702-8.

88. Klar N, Donner A. Current and future challenges in the design and analysis of cluster randomization trials. *Stat Med* 2001;20:3729-40.

89. Deeks JJ, Dinnes J, D'Amico R, Sowden AJ, Sakarovitch C, Song F, et al. Evaluating non-randomised intervention studies. *Health Technol Assess* 2003;7:1-173.

90. Altman DG, Bland JM. Absence of evidence is not evidence of absence. *BMJ* 1995;311:485.

91. Schulz KF, Grimes DA. Sample size calculations in randomised trials: mandatory and mystical. *Lancet* 2005;365:1348-53.

92. Devereaux PJ, Manns BJ, Ghali WA, Quan H, Guyatt GH. The reporting of methodological factors in randomized controlled trials and the association with a journal policy to promote adherence to the Consolidated Standards of Reporting Trials (CONSORT) checklist. *Control Clin Trials* 2002;23:380-8.

93. Jüni P, Altman DG, Egger M. Assessing the quality of randomised controlled trials. In: Egger M, Davey Smith G, Altman DG, editors. *Systematic reviews in health care: meta-analysis in context*. 2nd ed. London: BMJ Books; 2001. p. 87-108.

94. Pildal J, Chan AW, Hrobjartsson A, Forfang E, Altman DG, Gøtzsche PC. Comparison of descriptions of allocation concealment in trial protocols and the published reports: cohort study. *BMJ* 2005;330:1049.

95. Hill CL, LaValley MP, Felson DT. Discrepancy between published report and actual conduct of randomized clinical trials. *J Clin Epidemiol* 2002;55:783-6.

96. Devereaux PJ, Choi PT, El-Dika S, Bhandari M, Montori VM, Schunemann HJ, et al. An observational study found that authors of randomized controlled trials frequently use concealment of randomization and blinding, despite the failure to report these methods. *J Clin Epidemiol* 2004;57:1232-6.

97. Soares HP, Daniels S, Kumar A, Clarke M, Scott C, Swann S, et al. Bad reporting does not mean bad methods for randomised trials: observational study of randomised controlled trials performed by the Radiation Therapy Oncology Group. *BMJ* 2004;328:22-4.

98. Altman DG, Schulz KF, Moher D, Egger M, Davidoff F, Elbourne D, et al. The revised CONSORT statement for reporting randomized trials: explanation and elaboration. *Ann Intern Med* 2001;134:663-94.

99. Des Jarlais DC, Lyles C, Crepaz N, TREND Group. Improving the reporting quality of nonrandomized evaluations of behavioral and public health interventions: the TREND statement. *Am J Public Health* 2004;94:361-6.

100. von Elm E, Altman DG, Egger M, Pocock SJ, Gøtzsche PC, Vandenbroucke JP, et al. The Strengthening the Reporting of Observational Studies in Epidemiology (STROBE) statement: guidelines for reporting observational studies. *Lancet* 2007;370:1453-7.

101. *EQUATOR Network [internet] Enhancing the Quality and Transparency of Health Research*. Centre for Statistics in Medicine, Oxford; [cited 2008 14 Aug]. Available from: www.equator-network.org/

102. Herbert RD, Bø K. Analysis of quality of interventions in systematic reviews. *BMJ* 2005;331:507-9.

103. Boutron I, Tubach F, Giraudeau B, Ravaud P. Methodological differences in clinical trials evaluating nonpharmacological and pharmacological treatments of hip and knee osteoarthritis. *JAMA* 2003;290:1062-70.

104. Medical Research Council. *A framework for development and evaluation of RCTs for complex interventions to improve health*. London: Medical Research Council; 2000.

105. Jüni P, Altman DG, Egger M. Systematic reviews in health care: assessing the quality of controlled clinical trials. *BMJ* 2001;323:42-6.

106. Egger M, Jüni P, Bartlett C, Holenstein F, Sterne J. How important are comprehensive literature searches and the assessment of trial quality in systematic reviews? Empirical study. *Health Technol Assess* 2003;7:1-76.

107. Wood L, Egger M, Gluud LL, Schulz KF, Jüni P, Altman DG, et al. Empirical evidence of bias in treatment effect estimates in controlled trials with different interventions and outcomes: meta-epidemiological study. *BMJ* 2008;336:601-5.

108. Balk EM, Bonis PA, Moskowitz H, Schmid CH, Ioannidis JP, Wang C, et al. Correlation of quality measures with estimates of treatment effect in meta-analyses of randomized controlled trials. *JAMA* 2002;287:2973-82.

109. Verhagen AP, de Bie RA, Lenssen AF, de Vet HC, Kessels AG, Boers M, et al. Impact of quality items on study outcome. Treatments in acute lateral ankle sprains. *Int J Technol Assess Health Care* 2000;16:1136-46.

110. Moher D, Jadad AR, Tugwell P. Assessing the quality of randomized controlled trials: current issues and future directions. *Int J Technol Assess Health Care* 1996;12:195-208.

111. Downs SH, Black N. The feasibility of creating a checklist for the assessment of the methodological quality both of randomised and non-randomised studies of health care interventions. *J Epidemiol Community Health* 1998;52:377-84.

112. Agency for Healthcare Research and Quality. *Systems to rate the strength of scientific evidence. Evidence Report/Technology Assessment: number 47*. Rockville, MD: Agency for Healthcare Research and Quality (AHRQ); 2002.

113. Moher D, Jadad AR, Nichol G, Penman M, Tugwell P, Walsh S. Assessing the quality of randomised controlled trials: an annotated bibliography of scales and checklists. *Control Clin Trials* 1995;16:62-73.

114. Chalmers TC, Smith H, Blackburn BA, Silverman B, Schroeder B, Reitman D, et al. A method for assessing the quality of a randomized control trial. *Control Clin Trials* 1981;2:31-49.

115. Ramsay CR, Matowe L, Grilli R, Grimshaw JM, Thomas RE. Interrupted time series designs in health technology assessment: lessons from two systematic reviews of behavior change strategies. *Int J Technol Assess Health Care* 2003;19:613-23.

116. Cochrane Effective Practice and Organisation of Care Group. *Including interrupted time series (ITS) designs in a EPOC review. Draft. Revised April 21, 1998. EPOC methods paper*: Cochrane Effective Practice and Organisation of Care Group; 1998. Available from: www.epoc.cochrane.org/Files/Website/Reviewer%20Resources/inttime.pdf

117. Jüni P, Witschi A, Bloch R, Egger M. The hazards of scoring the quality of clinical trials for meta-analysis. *JAMA* 1999;282:1054-60.

118. Colle F, Rannou F, Revel M, Fermanian J, Poiraudeau S. Impact of quality scales on levels of evidence inferred from a systematic review of exercise therapy and low back pain. *Arch Phys Med Rehabil* 2002;83:1745-52.

119. Greenland S. Quality scores are useless and potentially misleading. Reply to "re: a critical look at some popular analytic methods". *Am J Epidemiol* 1994;140:300-1.

120. Sterne JA, Egger M, Moher D. Chapter 10: Addressing reporting biases. In: Higgins JPT, Green S, editors. *Cochrane handbook for systematic reviews of interventions. Version 5.0.0 (updated February 2008)*: The Cochrane Collaboration; 2008. Available from: www.cochrane-handbook.org

121. Centre for Reviews and Dissemination. *Interventions for people bereaved through suicide: a systematic review.* CRD Report 38. York: University of York; 2008.

122. King S, Griffin S, Hodges Z, Weatherly H, Asseburg C, Richardson G, et al. A systematic review and economic model of the effectiveness and cost-effectiveness of methylphenidate, dexamfetamine and atomoxetine for the treatment of attention deficit hyperactivity disorder in children and adolescents. *Health Technol Assess* 2006;10:1-146.

123. Arai L, Britten N, Popay J, Roberts H, Petticrew M, Rodgers M, et al. Testing methodological developments in the conduct of narrative synthesis: a demonstration review of research on the implementation of smoke alarm interventions. *Evidence & Policy* 2007;3:361-83.

124. Popay J, Roberts H, Sowden A, Petticrew M, Britten N, Arai L, et al. Developing guidance on the conduct of narrative synthesis in systematic reviews. *J Epidemiol Community Health* 2005;59 Suppl 1:A7.

125. Popay J, Roberts H, Sowden A, Petticrew M, Arai L, Rodgers M, et al. *Guidance on the conduct of narrative synthesis in systematic reviews*. ESRC Research Methods Programme; 2006.

126. Rodgers M, Sowden A, Petticrew M, Arai L, Roberts H, Britten N, et al. Testing methodological guidance on the conduct of narrative synthesis in systematic reviews: effectiveness of interventions to promote smoke alarm ownership and function. *Evaluation* 2009;15:47-71.

127. Mays N, Pope C, Popay J. Systematically reviewing qualitative and quantitative evidence to inform management and policy-making in the health field. *J Health Serv Res Policy* 2005;10 Suppl 1:6-20.

128. Ragin CC. *The comparative method: moving beyond qualitative and quantitative strategies*. Berkeley, CA: University of California Press; 1987.

129. Schünemann HJ, Oxman AD, Higgins JPT, Vist GE, Glasziou P, Guyatt GH. Chapter 11: Presenting results and 'Summary of findings tables'. In: Higgins JPT, Green S, editors. *Cochrane handbook for systematic reviews of interventions. Version 5.0.0 (updated February 2008)*: The Cochrane Collaboration; 2008. Available from: www.cochrane-handbook.org

130. Schünemann HJ, Oxman AD, Vist GE, Higgins JPT, Deeks JJ, Glasziou P, et al. Chapter 12: Interpreting results and drawing conclusions. In: Higgins JPT, Green S, editors. *Cochrane handbook for systematic reviews of interventions. Version 5.0.0 (updated February 2008)*: The Cochrane Collaboration; 2008. Available from: www.cochrane-handbook.org

131. Centers for Disease Control and Prevention. *The guide to community preventative services: what works to promote health? Task Force on Community Preventive Services [monograph online]* Atlanta, GA: Centers for Disease Control and Prevention; 2005. Available from: www.thecommunityguide.org

132. Busse R, Orvain J, Velasco M, Perleth M, Drummond M, Gurtner F, et al. Best practice in undertaking and reporting health technology assessments. Working group 4 report. *Int J Technol Assess Health Care* 2002;18:361-422.

133. Britten N, Campbell R, Pope C, Donovan J, Morgan M, Pill R. Using meta ethnography to synthesise qualitative research: a worked example. *J Health Serv Res Policy* 2002;7:209-15.

134. Whitehead A. *Meta-analysis of controlled clinical trials*. Chichester: John Wiley & Sons; 2002.

135. Cooper H, Hedges LV, editors. *The handbook of research synthesis*. New York: Russell Sage Foundation, 1994.

136. Sutton AJ, Higgins JPT. Recent developments in meta-analysis. *Stat Med* 2008;27:625-50.

137. Deeks JJ, Altman DG, Bradburn MJ. Statistical methods for examining heterogeneity and combining results from several studies in meta-analysis. In: Egger M, Davey Smith G, Altman DG, editors. *Systematic reviews in health care: meta-analysis in context*. 2nd ed. London: BMJ Books; 2001. p. 285-312.

138. DerSimonian R, Laird N. Meta-analysis in clinical trials. *Control Clin Trials* 1986;7:177-88.

139. Yusuf S, Peto R, Lewis J, Collins R, Sleight P. Beta blockade during and after myocardial infarction: an overview of the randomized trials. *Prog Cardiovasc Dis* 1985;27:335-71.

140. Greenland S, Salvan A. Bias in the one-step method for pooling study results. *Stat Med* 1990;9:247-52.

141. Zhang J, Yu KF. What's the relative risk? A method of correcting the odds ratio in cohort studies of common outcomes. *JAMA* 1998;280:1690-1.

142. Sinclair JC, Bracken MB. Clinically useful measures of effect in binary analysis of randomized trials. *J Clin Epidemiol* 1994;47:881-9.

143. Sackett DL, Deeks JJ, Altman DG. Down with odds ratios! *Evid Based Med* 1996;1:164-6.

144. Bradburn MJ, Deeks JJ, Berlin JA, Russell Localio A. Much ado about nothing: a comparison of the performance of meta-analytical methods with rare events. *Stat Med* 2007;26:53-77.

145. Rosenthal R. Parametric measures of effect size. In: Cooper H, Hedges LV, editors. *The handbook of research synthesis*. New York: Russell Sage Foundation; 1994. p. 231-44.

146. Glass GV. Primary, secondary and meta-analysis of research. *Education Res* 1976;5:3-8.

147. Parmar MK, Torri V, Stewart L. Extracting summary statistics to perform meta-analyses of the published literature for survival endpoints. *Stat Med* 1998;17:2815-34.

148. Williamson PR, Tudur Smith C, Hutton JL, Marson AG. Aggregate data meta-analysis with time-to-event outcomes. *Stat Med* 2002;21:3337-51.

149. Tierney JF, Stewart LA, Ghersi G, Burdett S, Sydes MR. Practical methods for incorporating summary time-to-event data into meta-analysis. *Trials* 2007;8:16.

150. Altman DG, De Stavola BL, Love SB, Stepniewska KA. Review of survival analyses published in cancer journals. *Br J Cancer* 1995;72:511-18.

151. Pocock SJ, Clayton TC, Altman DG. Survival plots of time-to-event outcomes in clinical trials: good practice and pitfalls. *Lancet* 2002;359:1686-9.

152. Lewis S, Clarke M. Forest plots: trying to see the wood and the trees. *BMJ* 2001;322:1479-80.

153. Galbraith RF. A note on graphical presentation of estimated odds ratios from several clinical trials. *Stat Med* 1988;7:889-94.

154. Chambers D, Epstein D, Walker S, Fayter D, Paton F, Wright K, et al. Endovascular stents for abdominal aortic aneurysms (EVAR): a systematic review. *Health Technol Assess [forthcoming]*.

155. Stewart LA, Parmar MKB. Meta-analysis of the literature or of individual patient data: is there a difference? *Lancet* 1993;341:418-22.

156. Barrat Al, Wyer PC, Guyatt G, Simpson JM. NNT for studies with long term follow up. *CMAJ* 2005;172:613-5.

157. Glioma Meta-analysis Trialists (GMT) Group. Chemotherapy in adult high-grade glioma: a systematic review and meta-analysis of individual patient data from 12 randomised trials. *Lancet* 2002;359:1011-8.

158. Askie L, Duley L, Henderson-Smart D, Stewart L. Antiplatelet agents for prevention of pre-eclampsia: a meta-analysis of individual patient data. *Lancet* 2007;369:1791-8.

159. Greenhouse JB, Iyengar S. Sensitivity analysis and diagnostics. In: Cooper H, Hedges LV, editors. *The handbook of research synthesis*. New York, NY: Russell Sage Foundation; 1994. p. 383-98.

160. Higgins JP, Thompson SG, Deeks JJ, Altman DG. Measuring inconsistency in meta-analyses. *BMJ* 2003;327:557-60.

161. Higgins JP, Thompson SG. Quantifying heterogeneity in a meta-analysis. *Stat Med* 2002;21:1539-58.

162. Thompson SG. Systematic review: why sources of heterogeneity in meta-analysis should be investigated. *BMJ* 1994;309:1351-5.

163. Stewart LA, Clarke MJ. Practical methodology of meta-analyses (overviews) using updated individual patient data. Cochrane Working Group. *Stat Med* 1995;14:2057-79.

164. Yusuf S, Wittes J, Probstfield J, Tryoler HA. Analysis and interpretation of treatment effects in subgroups of patients in randomized clinical trials. *JAMA* 1991;266:93-8.

165. Oxman AD, Guyatt GH. A consumer's guide to subgroup analyses. *Ann Intern Med* 1992;116:78-84.

166. Thompson SG, Higgins JP. How should meta-regression analyses be undertaken and interpreted? *Stat Med* 2002;21:1559-73.

167. Lau J, Ioannidis JP, Schmid CH. Summing up evidence: one answer is not always enough. *Lancet* 1998;351:123-7.

168. Rothstein HR, Sutton AJ, Borenstein M. *Publication bias in meta-analysis: prevention, assessment and adjustments*. Chichester: Wiley; 2005.

169. Sterne JA, Egger M. Funnel plots for detecting bias in meta-analysis: guidelines on choice of axis. *J Clin Epidemiol* 2001;54:1046-55.

170. Tang JL, Liu JL. Misleading funnel plot for detection of bias in meta-analysis. *J Clin Epidemiol* 2000;53:477-84.

171. Liu GJ, Wu TX. Clinical implementation of forest plots in meta-analysis. *Chinese Journal of Evidence-Based Medicine* 2004;4:198-201.

172. Peters JL, Sutton AJ, Jones DR, Abrams KR, Rushton L. Contour-enhanced meta-analysis funnel plots help distinguish publication bias from other causes of asymmetry. *J Clin Epidemiol* 2008;61:991-6.

173. Begg CB, Mazumdar M. Operating characteristics of a rank correlation test for publication bias. *Biometrics* 1994;50:1088-101.

174. Egger M, Davey Smith G, Schneider M, Minder C. Bias in meta-analysis detected by a simple, graphical test. *BMJ* 1997;315:629-34.

175. Harbord R, Egger M, Sterne J. A modified test for small-study effects in meta-analyses of controlled trials with binary endpoints. *Stat Med* 2006;25:3443-57.

176. Sutton AJ, Abrams KR, Jones DR. An illustrated guide to the methods of meta-analysis. *J Eval Clin Pract* 2001;7:135-48.

177. Taylor S, Tweedie R. *A non-parametric 'trim and fill' method of assessing publication bias in meta-analysis. Technical Report*. Fort Collins, CO: Department of Statistics, Colorado State University; 1998.

178. Sutton AJ, Duval SJ, Tweedie RL, Abrams KR, Jones DR. Empirical assessment of effect of publication bias on meta-analyses. *BMJ* 2000;320:1574-7.

179. Lau J, Ioannidis J, Terrin N, Schmid C, Olkin I. The case of the misleading funnel plot. *BMJ* 2006;333:597-600.

180. Ioannidis J, Trikalinos T. The appropriateness of asymmetry tests for publication bias in meta-analyses: a large survey. *CMAJ* 2007;176:1091-6.

181. Dickersin K. The existence of publication bias and risk factors for its occurrence. *JAMA* 1990;163:1385-9.

182. Dickersin K, Rennie D. Registering clinical trials. *JAMA* 2003;290:516-23.

183. Bradley M, Cullum N, Nelson EA, Petticrew M, Sheldon T, Torgerson D. Systematic reviews of wound care management (2). Dressings and topical agents used in the healing of chronic wounds. *Health Technol Assess* 1999;3:1-35.

184. Unnebrink K, Windeler J. Intention-to-treat: methods for dealing with missing values in clinical trials of progressively deteriorating diseases. *Stat Med* 2001;20:3931-46.

185. Higgins JPT, White IR, Wood AM. Imputation methods for missing outcome data in meta-analysis of clinical trials. *Clin Trials* 2008;5:225-39.

186. Eldridge SM, Ashby D, Feder GS, Rudnicka AR, Ukoumunne OC. Lessons for cluster randomized trials in the twenty-first century: a systematic review of trials in primary care. *Clin Trials* 2004;1:80-90.

187. Eldridge S, Ashby D, Bennett C, Wakelin M, Feder G. Internal and external validity of cluster randomised trials: systematic review of recent trials. *BMJ* 2008;336:876-80.

188. Elbourne DR, Altman DG, Higgins JP, Curtin F, Worthington HV, Vail A. Meta-analyses involving cross-over trials: methodological issues. *Int J Epidemiol* 2002;31:140-9.

189. Sutton AJ, Cooper NJ, Jones DR. Formalising the use of evidence synthesis to designing future research coherently and efficiently: a framework proposal [abstract]. In: *16th Cochrane Colloquium.* 2008 Oct 3-7; Freiburg, Germany.

190. Song F, Altman DG, Glenny AM, Deeks JJ. Validity of indirect comparison for estimating efficacy of competing interventions: empirical evidence from published meta-analyses. *BMJ* 2003;326:472.

191. Glenny AM, Altman DG, Song F, Sakarovitch C, Deeks JJ, D'Amico R, et al. Indirect comparisons of competing interventions. *Health Technol Assess* 2005;9:1-134.

192. Caldwell DM, Ades AE, Higgins JP. Simultaneous comparison of multiple treatments: combining direct and indirect evidence. *BMJ* 2005;331:879-900.

193. Salanti G, Higgins J, Ades AE, Ionnidis JP. Evaluations of networks of randomised trials. *Stat Methods Med Res* 2007;17:279-301.

194. Babapulle MN, Joseph L, Belisle P, Brophy JM, Eisenberg MJ. A hierarchical Bayesian meta-analysis of randomised clinical trials of drug-eluting stents. *Lancet* 2004;364:583-91.

195. Price D, Jefferson T, Demicheli V. Methodological issues arising from systematic reviews of the evidence of safety of vaccines. *Vaccine* 2004;22:2080-4.

196. Lilford RJ, Thornton JG, Braunholtz D. Clinical trials and rare diseases: a way out of a conundrum. *BMJ* 1995;311:1621-5.

197. Dumouchel W. Meta-analysis for dose-response models. *Stat Med* 1995;14:679-85.

198. Sutton AJ, Abrams KR. Bayesian methods in meta-analysis and evidence synthesis. *Stat Methods Med Res* 2001;10:277-303.

199. Sutton AJ, Abrams KR, Jones DR, Sheldon TR. Methods for meta-analysis in medical research. *Chichester, UK: John Wiley & Sons Ltd, 2000*.

200. Spiegelhalter D, Abrams K, Myles J. *Bayesian approaches to clinical trials and health-care evaluation* Chichester (UK): John Wiley & Sons; 2004.

201. Hartling L, Klassen T, Moher D, Tubman M, Chiu A, Wiebe N. Quality of reporting of systematic reviews and its affect on estimates of intervention effectiveness [abstract]. In: *12th Cochrane Colloquium*; 2004 Oct 2-6; Ottawa, Ontario, Canada.

202. Glasziou P, Meats E, Heneghan C, Shepperd S. What is missing from descriptions of treatments in trials and reviews? *BMJ* 2008;336:1472-4.

203. Craig P, Dieppe P, Macintyre S, Michie S, Nazareth I, Petticrew M, et al. Developing and evaluating complex interventions: the new Medical Research Council guidance. *BMJ* 2008;337:a1655.

204. Craig P, Dieppe P, Macintyre S, Michie S, Nazareth I, Petticrew M, et al. *Developing and evaluating complex interventions: new guidance*. London: Medical Research Council; 2008. Available from: www.mrc.ac.uk/Utilities/Documentrecord/index.htm?d=MRC004871

205. Moher D, Tetzlaff J, Tricco AC, Sampson M, Altman DG. Epidemiology and reporting characteristics of systematic reviews. *PLoS Med* 2007;4:e78.

206. PLoS Medicine Editors. Many reviews are systematic but some are more transparent and completely reported than others. *PLoS Med* 2007;4:e147.

207. Taylor RB. *The clinician's guide to medical writing*. New York, NY: Springer; 2005.

208. Hall GM, editor. *How to write a paper*. 3rd ed. London: BMJ Books, 2003.

209. Fraser J. *How to publish in biomedicine: 500 tips for success*. Abingdon: Radcliffe Medical Press; 1997.

210. Iverson C, Flanagin A, Fontanarosa PB, Glass RM, Glitman P, Lantz JC, et al. *American Medical Association manual of style: a guide for authors and editors*. 9th ed. Baltimore, MD: Williams & Wilkins; 1998.

211. American Psychological Association. *Publication manual of the American Psychological Association*. 5th ed. Washington, DC: American Psychological Association; 2001.

212. Burchfield RW. *The new Fowler's modern English usage*. Oxford: Clarendon Press; 1996.

213. Lavis J, Davies H, Oxman A, Denis JL, Golden-Biddle K, Ferlie E. Towards systematic reviews that inform health care management and policy-making. *J Health Serv Res Policy* 2005;10 Suppl 1:35-48.

214. International Committee of Medical Journal Editors (ICMJE). *Uniform requirements for manuscripts submitted to biomedical journals: writing and editing for biomedical publication. Updated October 2007 [internet]*. American College of Physicians; 2007. [cited 2008 12 Aug]. Available from: www.icmje.org/

215. Ahmed SM, Maurana CA, Engle JA, Uddin DE, Glaus KD. A method for assigning authorship in multiauthored publications. *Fam Med* 1997;29:42-4.

216. Smith R. Authorship is dying: long live contributorship. *BMJ* 1997;315:696.

217. Wilson P, Petticrew M. Why promote the findings of single research studies? *BMJ* 2008;336:722.

218. Skelton JR, Edwards SJ. The function of the discussion section in academic medical writing. *BMJ* 2000;320:1269-70.

219. Docherty M, Smith R. The case for structuring the discussion of scientific papers. *BMJ* 1999;318:1224-5.

220. NHS R&D HTA Programme. *Instructions to authors: preparation of reports for the HTA programme*. Southampton: The National Coordinating Centre for Health Technology Assessment; 2007.

221. Michie S, Johnston M. Changing clinical behaviour by making guidelines specific. *BMJ* 2004;328:343-5.

222. Grol R, Dalhuijsen J, Thomas S, Veld C, Rutten G, Mokkink H. Attributes of clinical guidelines that influence use of guidelines in general practice: observational study. *BMJ* 1998;317:858-61.

223. Michie S, Lester K. Words matter: increasing the implementation of clinical guidelines. *Qual Saf Health Care* 2005;14:367-70

224. Sackett D, Straus S, Richardson W, Rosenberg W, Haynes R, editors. *Evidence-based medicine: how to practice and teach EBM*. Edinburgh: Churchill Livingstone; 2000.

225. Atkins D, Best D, Briss PA, Eccles M, Falck-Ytter Y, Flottorp S, et al. Grading quality of evidence and strength of recommendations. *BMJ* 2004;328:1490-7.

226. Harris RP, Helfand M, Woolf SH, Lohr KN, Mulrow CD, Teutsch SM, et al. Current methods of the US Preventive Services Task Force: a review of the process. *Am J Prev Med* 2001;20:21-35.

227. Treadwell JR, Tregear SJ, Reston JT, Turkelson CM. A system for rating the stability and strength of medical evidence. *BMC Med Res Methodol* 2006;6:52.

228. Guyatt GH, Oxman AD, Kunz R, Falck-Ytter Y, Vist GE, Liberati A, et al. Going from evidence to recommendations. *BMJ* 2008;336:1049-51.

229. Guyatt GH, Oxman AD, Kunz R, Vist GE, Falck-Ytter Y, Schünemann HJ, et al. What is 'quality of evidence' and why is it important to clinicians? *BMJ* 2008;336:995-8.

230. Guyatt GH, Oxman AD, Vist GE, Kunz R, Falck-Ytter Y, Alonso-Coello P, et al. GRADE: an emerging consensus on rating quality of evidence and strength of recommendations. *BMJ* 2008;336:924-6.

231. Brown P, Brunnhuber K, Chalkidou K, Chalmers I, Clarke C, Fenton M, et al. How to formulate research recommendations. *BMJ* 2006;333:804-6.

232. Greenhalgh T. How to formulate research recommendations: the pie or the slice? *BMJ* 2006;333:917.

233. Medical Research Council. *Good research practice*. London: Medical Research Council; 2000.

234. Lomas J. Retailing research: increasing the role of evidence in clinical services for childbirth. *Milbank Q* 1993;71:439-75.

235. NHS Centre for Reviews and Dissemination. Getting evidence into practice. *Effective Health Care* 1999;5:1-12.

236. Ferlie E, Gabbay J, FitzGerald L, Locock L, Dopson S. Evidence based medicine and organisational change: an overview of some recent qualitative research. In: Ashburner L, editor. *Organisational behaviour and organisational studies in health care: reflections on the future*. Basingstoke: Palgrave; 2001. p. 18-42.

237. Grimshaw JM, Thomas RE, MacLennan G, Fraser C, Ramsay CR, Vale L, et al. Effectiveness and efficiency of guideline dissemination and implementation strategies. *Health Technol Assess* 2004;8:1-84.

238. Greenhalgh T, Robert G, Bate P, Kyriakidou O, MacFarlane F, Peacock R, et al. *How to spread good ideas: a systematic review of the literature on diffusion, dissemination and sustainability of innovations in health service delivery and organisation. Report for the National Co-ordinating Centre for NHS Service Delivery and Organisation R & D (NCCSDO)*. London: NCCSDO; 2004.

239. Rogers EM. *Diffusion of innovations*. 5th ed. New York, NY; London: Free Press; 2003.

240. Tugwell P, Robinson V, Grimshaw J, Santesso N. Systematic reviews and knowledge translation. *Bull World Health Organ* 2006;84:643-51.

241. Graham ID, Logan J, Harrison MB, Straus SE, Tetroe J, Caswell W, et al. Lost in knowledge translation: time for a map? *J Contin Educ Health Prof* 2006;26:13-24.

242. Canadian Institutes of Health Research. *About knowledge translation [internet]*. Canadian Institutes of Health Research; 2008. [cited May 14]. Available from: www.cihr-irsc.gc.ca/e/29418.html

243. Bowen S, Zwi A. Pathways to "evidence-informed" policy and practice: a framework for action. *PLoS Med* 2005;2:e166.

244. Ogilvie D, Craig P, Griffin S, Macintyre S, Petticrew M, Wareham N. *Towards a translational framework for public health research [unpublished manuscript]*: MRC Population Health Sciences Research Network; 2008.

245. World Health Organization. *Bridging the 'know–do' gap: meeting on knowledge translation in global health. 10–12 October 2005*. Geneva, Switzerland: World Health Organization.

246. Knott J, Wildavsky A. If dissemination is the solution, what is the problem? *Knowledge: Creation, Diffusion, Utilization* 1980;1:537-78.

247. McGuire WJ. Input and output variables currently promising for constructing persuasive communications. In: Rice R, Atkin C, editors. *Public communication campaigns*. 3rd ed. Thousand Oaks, CA: Sage; 2001. p. 22-48.

248. Ryan J, Piercy J, James P. Assessment of NICE guidance on two surgical procedures. *Lancet* 2004;363:1525-6.

249. Sheldon TA, Cullum N, Dawson D, Lankshear A, Lowson K, Watt I, et al. What's the evidence that NICE guidance has been implemented? Results from a national evaluation using time series analysis, audit of patients' notes, and interviews. *BMJ* 2004;329:999-1004.

250. Mason J, Freemantle N, Browning G. Impact of effective health care bulletin on treatment of persistent glue ear in children: time series analysis. *BMJ* 2001;323:1096-7.

251. Black N, Hutchings A. Reduction in the use of surgery for glue ear: did national guidelines have an impact? *Qual Saf Health Care* 2002;11:121-4.

252. Freemantle N, Mason JM, Watt I. Evidence into practice. Prescribing selective serotonin reuptake inhibitors. *Int J Technol Assess Health Care* 1998;14:387-91.

253. Mason J, Freemantle N, Young P. The effect of the distribution of Effective Health Care Bulletins on prescribing selective serotonin reuptake inhibitors in primary care. *Health Trends* 1998;30:120-2.

254. Aristotle. *On rhetoric: a theory of civic discourse*. New York, NY: Oxford University Press; 1991.

255. McGuire WJ. The nature of attitudes and attitude change. In: Lindzey G, Aronsen E, editors. *Handbook of social psychology*. Reading, MA: Addison-Wesley Publishing; 1969. p. 136-314.

256. Winkler JD, Lohr KN, Brook RH. Persuasive communication and medical technology assessment. *Arch Intern Med* 1985;145:314-7.

257. Lavis JN, Robertson D, Woodside JM, McLeod CB, Abelson J, Knowledge Transfer Study Group. How can research organizations more effectively transfer research knowledge to decision makers? *Milbank Q* 2003;81:221-48.

258. Hughes M, McNeish D, Newman T, Roberts H, Sachdev D. *What works? Making connections: linking research and practice. A review by Barnardo's Research and Development Team*. Ilford: Barnardo's; 2000.

259. Atkin C. Theory and principles of media health campaigns. In: Rice R, Atkin C, editors. *Public communication campaigns*. 3rd ed. Thousand Oaks, CA: Sage; 2001. p. 49-68.

260. Weiss CH. The many meanings of research utilization. *Public Adm Rev* 1979;39:426-31.

261. Innvaer S, Vist G, Trommald M, Oxman A. Health policy-makers' perceptions of their use of evidence: a systematic review. *J Health Serv Res Policy* 2002;7:239-44.

262. Lavis JN. Linking research to action. In: *World report on knowledge for better health: strengthening health systems*. Geneva, Switzerland: World Health Organization; 2004. p. 97-130.

263. Lavis JN, Lomas J, Hamid M, Sewankambo NK. Assessing country-level efforts to link research to action. *Bull World Health Organ* 2006;84:620-8.

264. Lomas J. Using 'linkage and exchange' to move research into policy at a Canadian foundation. *Health Aff (Millwood)* 2000;19:236-40.

265. Lavis JN, Ross SE, Hurley JE, Hohenadel JM, Stoddart GL, Woodward CA, et al. Examining the role of health services research in public policymaking. *Milbank Q* 2002;80:125-54.

266. Dash P. *Increasing the impact of health services research on service improvement and delivery: a report for The Health Foundation and the Nuffield Trust*. London: Health Foundation, Nuffield Trust; 2003.

267. Lavis J, Ross S, McLeod C, Gildiner A. Measuring the impact of health research. *J Health Serv Res Policy* 2003;8:165-70.

268. Buxton M, Hanney S. How can payback from health research be assessed? *J Health Serv Res Policy* 1996;1:35-43.

269. Kuruvilla S, Mays N, Walt G. Describing the impact of health services and policy research. *J Health Serv Res Policy* 2007;12 Suppl 1:23-31.

CHAPTER 2

SYSTEMATIC REVIEWS OF CLINICAL TESTS

2.1 INTRODUCTION **111**

2.2 DIAGNOSTIC TESTS **113**

2.2.1 The review question **113**

2.2.1.1 Population 113

2.2.1.2 Intervention (index test) 113

2.2.1.3 Reference standard/comparator 114

2.2.1.4 Outcome measures 115

2.2.1.5 Study design 118

2.2.2 Identifying research evidence **119**

2.2.2.1 Sources 119

2.2.2.2 Database searching 120

2.2.3 Publication bias **120**

2.2.4 Data extraction **121**

2.2.5 Quality assessment **124**

2.2.6 Data synthesis **129**

2.2.6.1 Assessment of statistical heterogeneity 129

2.2.6.2 Meta-analysis 130

2.2.6.3 Software 133

2.2.7 Presentation of results **133**

2.3 PROGNOSTIC TESTS **135**

2.3.1 Defining the review question: setting inclusion criteria **135**

2.3.1.1 Population/study design 135

2.3.1.2 Intervention 136

2.3.2 Defining the review question: other considerations **136**

2.3.2.1 Publication bias and sample size 136

2.3.2.2 Cutpoints 136

2.3.2.3 IPD vs summary data 137

2.3.3 Identifying research evidence **137**

2.3.4 Data extraction **137**

2.3.5 Quality assessment **138**

2.3.5.1 Generic criteria 139

2.3.5.2 Context-specific criteria 139

2.3.5.3 Implementing quality assessment 139

2.3.5.4 Quality of reporting 140

2.3.6 Data synthesis **142**

2.3.6.1 Outcome measures 142

2.3.6.2 Adjustment for other variables 142

2.3.6.3 Sensitivity analyses 143

2.3.7 Case study **144**

2.3.8 Systematic review as a driver for improved study quality **146**

REFERENCES **148**

2.1 INTRODUCTION

Clinical tests are routinely used for diagnosis, confirming or excluding the presence of a disease or condition (such as pregnancy). They are also used to monitor disease progression, assess prognosis, and screen asymptomatic populations for disease. Any process that yields information used to inform patient management can be regarded as a clinical test.[1] This includes a wide range of processes from history taking and physical examination to complex imaging techniques. The test itself is an intervention and forms part of the continuum of patient care. New tests are adopted into clinical practice for a number of reasons, including replacement of an existing test (where the new test is expected to reduce the negative impact on the patient, provide better information, or equivalent information for less cost), triage (to decide whether a more expensive or invasive test is necessary), or as an addition to the existing testing protocol.

The ultimate aim of any research on clinical tests should be to determine impact upon patient management and outcome. An RCT comparing the effect of different diagnostic strategies on one or more clinical outcomes could be considered ideal, as it provides direct information on the benefit to patients and can be modified to address various types of diagnostic question.[2] However, RCTs may not be appropriate for addressing all diagnostic questions[3, 4] and to date much of the research on diagnostic tests is in the form of test accuracy studies. The basic aim of test accuracy studies is to assess how well a test can distinguish between people with and without the disease/condition of interest. The outcome measures used describe the probabilistic relationships between positive and negative test results, and the presence or absence of disease, as compared with the best currently available method (i.e. the clinical reference standard). As such, test accuracy studies do not directly measure the relative benefits and harms to patients of testing. Evidence on the accuracy of a test, combined with evidence of a prognostic link between the target condition and preventable morbidity/mortality, may be considered indicative of the likely effectiveness of the test.[5] Where a new test is being evaluated, evidence for a prognostic link between the target disease/condition and long-term morbidity or mortality should be available as should an effective intervention. However, this is not always the case as tests can be established in clinical practice with limited supporting evidence.

When considering a systematic review of test accuracy studies, it is important to assess whether review findings will be able to provide the information necessary to inform clinical practice. Any review of test accuracy is likely to be of limited value where evidence is lacking that the disease/condition is associated with long-term morbidity or mortality, or where no effective intervention is available. This is illustrated by the following examples:

- Magnetic Resonance Angiography (MRA) versus intra-arterial Digital Subtraction Angiography (DSA) for the detection of carotid artery stenosis.[6] There is evidence from RCTs that carotid endarterectomy is an effective treatment for symptomatic carotid artery stenosis at thresholds defined by DSA. MRA is a less invasive test option. A review of test accuracy is therefore likely to be informative.
- Ultrasound versus Micturating Cystourethrography (MCUG) for the detection of vesicoureteric reflux (VUR) in children with urinary tract infection (UTI).[7] There is conflicting evidence of a link between VUR and long-term renal damage and

the effectiveness of treatment options, such as prophylactic antibiotics, is also uncertain. A review of test accuracy alone is therefore unlikely to be informative.

Although some study designs, such as those based upon multivariable prediction modelling, may better reflect the true nature of the diagnostic workup and are potentially more informative than test accuracy studies,[8, 9] they are rare. Consequently, systematic review methods for assessing clinical tests have largely focused upon test accuracy studies and this chapter discusses methods developed specifically to deal with such studies. *Section 2.2* focuses on diagnostic accuracy studies, but the methods described also apply to test accuracy studies used to assess the performance of new screening tests, within established screening programmes. The clinical effectiveness of screening programmes is best evaluated using RCTs and systematic reviews of such studies should follow the principles outlined in *Chapter 1. Section 2.3* describes methods for reviewing prognostic studies.

In light of the limitations described in relation to test accuracy studies, careful consideration should always be given to the likely informative value and any additional data requirements before undertaking a systematic review of test accuracy.

2.2 DIAGNOSTIC TESTS

2.2.1 The review question

As with all systematic reviews, the development of a clear, well-defined question is essential to maintaining transparency of the review process and to the quality and relevance of the findings. Some aspects of the question require particular consideration when planning a review of test accuracy.

2.2.1.1 Population

Diagnostic tests perform differently in different populations,[10, 11] for example it would generally be inappropriate to evaluate the performance of a test in a secondary care population when the test is mainly used in primary care. Both frequency and severity of the target condition would be expected to be greater in secondary care. It is therefore important to clearly define the population of interest. The ideal study sample for a test accuracy study is a consecutive or randomly selected series of patients in whom the target condition is suspected, or for screening studies, the target population. Because participant sampling methods are often poorly reported in test accuracy studies,[12] using the sampling method as an inclusion/exclusion criterion is likely to result in a substantial reduction in available data. It is likely to be more useful to consider the sampling method and/or its reporting as an aspect of study quality (see *Section 2.2.5 Quality assessment*) and to base the inclusion criteria relating to the population upon participant characteristics. For example in a review comparing the accuracy of different imaging techniques, the inclusion criteria might state that only patients with a specified level of symptoms, representative of those in whom the test would be used for intervention planning, are eligible.

2.2.1.2 Intervention (index test)

In reviews of test accuracy the 'index test' (the test whose performance is being evaluated) can be viewed as the intervention. As with any review, the scope of the question can be broad such as 'what is the optimum testing pathway for the diagnosis and follow-up investigation of childhood urinary tract infection (UTI)?'[13] or it can be narrow; for example 'what is the diagnostic accuracy of magnetic resonance angiography (MRA) when compared with intra-arterial x-ray angiography, for the detection of carotid artery stenosis?'[6] The former is likely to include a number of different technologies, addressing multiple target conditions, whereas the latter compares the performance of an alternative (replacement), less invasive or less costly diagnostic technology with that of the reference standard for the detection of a specified target condition. The rate of technological development may be an important consideration; in this latter example inclusion of MRA techniques that are already obsolete in clinical practice, is unlikely to be useful.

Careful consideration should always be given to the equivalence of different analytical techniques when setting inclusion criteria. For example, a systematic review of faecal occult blood tests to screen for colorectal cancer[14, 15] evaluated both immunochemical

and colourimetric methods for detecting blood in the faeces; though both methods target blood, they cannot be considered equivalent tests.

The traditional concept of test accuracy often implies the dichotomisation of data into test results which are classified as positive (target condition present) or negative (target condition absent). Any systematic review of test accuracy will therefore need to consider diagnostic thresholds (points at which results are classified as positive or negative) for each included index test.

2.2.1.3 Reference standard/comparator

The reference standard is usually the best test currently available, and is the standard against which the index test is compared. It need not be the test used routinely in practice (although it can be), and may include information which is not known for some time after the tests have been done (e.g. follow-up of test negatives in cancer).

The test accuracy study is based upon a one-sided comparison between the results of the index test and those of the reference standard. Any discrepancy is assumed to arise from error in the index test. Selection of the reference standard is therefore critical to the validity of a test accuracy study and the definition of the diagnostic threshold forms part of that reference standard.

It is important to note that the assumption of 100% accuracy for the reference standard rarely holds true in practice. This represents a fundamental flaw in the test accuracy study design, since the index test can never be deemed to perform better than the reference standard, and its value may therefore be underestimated.[16]

Where several tests are available to diagnose the target condition, there is often no consensus about which test constitutes the reference standard. In such cases a composite reference standard, which combines the results of several available tests to produce a better indicator of true disease status may be used.[17] A number of statistical methods have been proposed to estimate the performance of tests in the absence of a single accepted reference standard.[18, 19]

There may be instances when it is deemed unethical to use an invasive procedure as a reference standard in a study.[20] In such cases, clinical follow-up and final diagnosis may sometimes be used as a surrogate reference standard. There will also be occasions when clinical follow-up and final diagnosis provides the most appropriate reference standard. The length of follow-up should ideally be defined in advance. Studies using follow-up and clinical outcome in this way may be viewed as prognostic studies in that they are measuring the accuracy with which the test is able to predict a future event, rather than the accuracy with which it is able to determine current status. Where such studies are included in a systematic review, it is important to define, in advance, what constitutes appropriate follow-up and hence an adequate reference standard.

The comparator is an alternative test, usually that which is used in current practice, against which the index test must be evaluated in order to assess its potential role. Ideally, this should be done by comparing index test and comparator to the reference standard in the same population.

2.2.1.4 Outcome measures

The primary outcome of interest for any systematic review of test accuracy is the data required to populate 2 x 2 contingency tables. These describe the relationship between the results of the index test and the reference standard at a given diagnostic threshold (point at which results are classified as positive or negative). The table includes the number of true positives (TP: those that have the disease and test positive), false positives (FP: those that do not have the disease and test positive), false negatives (FN: those that do have the disease and test negative) and true negatives (TN: those that do not have the disease and test negative). See *Figure 2.1.*

		Reference standard	
		**Positive**	**Negative**
**Index test**	**Positive**	TP	FP
	**Negative**	FN	TN

Figure 2.1: The 2x2 contingency table

From the 2 x 2 contingency table, the following commonly used measures of test performance can be calculated:

Sensitivity =

$$\frac{TP}{TP + FN}$$

The proportion of people with the target condition who have a positive test result.

Specificity =

$$\frac{TN}{TN + FP}$$

The proportion of people without the target condition who have a negative test result.

Overall accuracy =

$$\frac{TP + TN}{TP + FN + FP + TN}$$

The proportion of people correctly classified by the test.

Positive predictive value =

$$\frac{TP}{TP + FP}$$

The probability of disease among persons with a positive test result.

Negative predictive value =

$$\frac{TN}{TN + FN}$$

The probability of non-disease among persons with a negative test result.

Positive likelihood ratio =

$$\frac{\left(\frac{TP}{TP + FN}\right)}{\left(\frac{FP}{FP + TN}\right)} \quad \text{or} \quad \frac{\text{Sensitivity}}{1 - \text{Specificity}}$$

Negative likelihood ratio =

$$\frac{\left(\frac{FN}{TP + FN}\right)}{\left(\frac{TN}{TN + FN}\right)} \quad \text{or} \quad \frac{1 - \text{Sensitivity}}{\text{Specificity}}$$

Likelihood ratios (LR) describe how many times more likely it is that a person with the target condition will receive a particular test result than a person without. Positive likelihood ratios greater than 10 or negative likelihood ratios less than 0.1 are sometimes judged to provide convincing diagnostic evidence.[21]

Diagnostic odds ratio =

$$\frac{TP \times TN}{FP \times FN}$$

Used as an overall indicator of diagnostic performance and calculated as the odds of a positive test result among those with the target condition, divided by the odds of a positive test result among those without the condition.

In primary studies, a receiver operating characteristic (ROC) curve describes the relationship between 'true positive fraction' (sensitivity) and 'false positive fraction' (1– specificity) for different positivity thresholds. It is used to display the trade-offs between sensitivity and specificity as a result of varying the diagnostic threshold.

Below is an example ROC analysis for serum thyroid stimulating hormone (TSH) as a diagnostic test for primary hypothyroidism:

Test results (Serum TSH) vs. reference standard (thyroid status)

Serum TSH (mIU/L)	Number with primary hypothyroidism	Number without primary hypothyroidism
<6	17	325
6-12	42	158
13-15	46	48
16-20	66	33
>20	284	5

2 x 2 contingency data for serum TSH diagnostic threshold (derived by summing the numbers of participants, with and without primary hypothyroidism, on either side of the diagnostic threshold)

Diagnostic threshold for a positive test result (mIU/L)	TP	FP	FN	TN
≥6	438	244	17	325
>12	396	86	59	483
>15	350	38	105	531
>20	284	5	171	564

Sensitivity and specificity values for each diagnostic threshold (derived from the 2 x 2 contingency data and expressed as percentages)

Diagnostic threshold for a positive test result (mIU/L)	Sensitivity	Specificity
≥6	96.2%	57.1%
>12	87.0%	84.9%
>15	76.9%	93.3%
>20	62.4%	99.1%

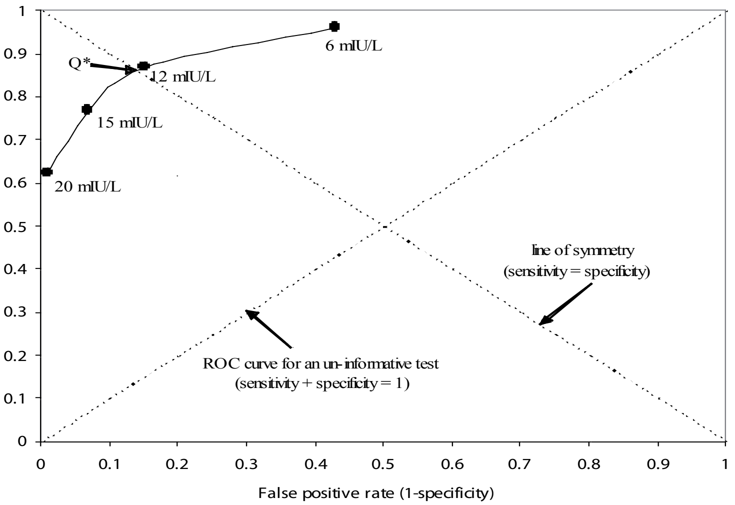

Figure 2.2: Example ROC curve for thyroid stimulating hormone (TSH)

'Q*', or maximal joint sensitivity and specificity, is the point on the ROC curve that intersects with the line of symmetry. It is sometimes used as an indicator of overall test performance where there is no clinical preference for maximising either sensitivity (minimizing false negatives) or specificity (minimizing false positives). However Q* is not useful if the thresholds at which tests have been evaluated do not lie close to the line of symmetry and can then give misleading results if used to compare performance between tests.

In some scenarios (e.g. tests used in population screening) a threshold which skews diagnostic performance may be preferable (e.g. minimizing the number of false negatives at the expense of some increase in the number of false positive results, in conditions/diseases where missing the presence of disease will lead to serious consequences). Overall diagnostic accuracy is summarised by the area under the curve (AUC); the closer the curve is to the upper left hand corner the better the diagnostic performance.[22] The AUC ranges from 0 to 1, with 0.5 indicating a poor test where the accuracy is equivalent to chance.

As with other types of intervention, when assessing the clinical effectiveness of a diagnostic test, it is important to consider all outcome measures which may be relevant to the use of the test in practice. These might include adverse events (see *Chapter 4*) and the preferences of patients, although inclusion of such information is rare.

2.2.1.5 Study design

There are two basic types of test accuracy study: 'single-gate' which are similar to consecutive series (and previously sometimes called diagnostic cohort studies) and 'two-gate' which are similar to case-control studies. The term 'two-gate' being used

where two sets of inclusion criteria or 'gates' are applied, one for participants who have the target condition and one for those who do not. These designs differ in structure from other cohort and case-control studies in that both are generally cross-sectional in nature.[23]

- The single-gate design includes participants in whom the disease status is unknown, and compares the results of the index test with those of the reference standard used to confirm diagnosis, i.e. it is broadly representative of the scenario in which the test would be used in practice.
- The two-gate design compares the results of the index test in patients with an established diagnosis of the target condition with its results in healthy controls or controls with another diagnosis (known status, with respect to the target condition, is therefore treated as the reference standard); i.e. it is unrepresentative of practice and is unlikely to contain the full spectrum of health and disease over which the test would be used.

There are inherent problems with the two-gate design that may lead to bias. The selective inclusion of cases with more advanced disease is likely to lead to over estimations of sensitivity and inclusion of healthy controls is likely to lead to over estimations of specificity. The recruitment of healthy controls from the general population has been associated with two- to three-fold increases in measures of test performance time-to-events derived from a diagnostic cohort design.[11, 24, 25] This over estimation can be increased further when cases of severe disease are used alongside healthy controls.[26] By contrast, where cases are derived from individuals with mild disease, underestimations of sensitivity can result.[27] Where the control group is derived from patients with alternative diagnoses, specificity may be under or overestimated, depending upon the alternative diagnosis.[23] In theory, the two-gate study design could produce a valid estimate of test performance if the cases were sampled to match the reference standard positive patients in a single-gate study (in terms of the spectrum of disease severity) and controls were matched to the reference standard negative patients (in terms of the spectrum of alternative conditions). In practice however, this is difficult to achieve.[23] Whilst two-gate studies are therefore of limited use in assessing how a test is likely to perform in clinical practice, they can be useful in the earlier phases of test development.[28]

Where systematic reviews include both single and two-gate study designs, careful consideration should be given to the methods of analysis and the impact of study design should be assessed in any meta-analyses.[29]

2.2.2 Identifying research evidence

2.2.2.1 Sources

The importance of searching a wide range of databases to avoid missing relevant diagnostic test accuracy studies has been demonstrated, with MEDLINE, EMBASE, BIOSIS, LILACS, Pascal and Science Citation Index all providing unique records.[30] The reference lists of included studies can also be a useful resource.

The Cochrane Diagnostic Test Accuracy Working Group[31] is creating a database of test accuracy studies,[32] similar to the non-topic specific Cochrane Central Register of Controlled Trials (CENTRAL) which includes details of published articles taken from bibliographic databases and other published and unpublished sources.[33]

2.2.2.2 Database searching

Many electronic databases do not have appropriate indexing terms to label test accuracy studies, and those that do tend not to apply them consistently.[30, 34-36] They also vary in their design which adds to the difficulty in identification.[34] The problem is compounded by the fact that the original authors are often poor at identifying their studies as being test accuracy.[30]

It has been reported that the use of filters to identify reports of diagnostic test accuracy studies in electronic databases may miss a considerable number of relevant articles and is therefore not generally considered appropriate.[34, 36, 37] Database searching should concentrate on terms for index tests and target conditions. If further restriction is required, it can be achieved by means of topic specific terms, rather than using a filter.[36, 38] It is hoped, however, that in time, as the issues of reporting and indexing diagnostic, screening and prognostic studies are more widely realised, the situation will improve allowing the development of more accurate filters.

2.2.3 Publication bias

As the data used in studies of test accuracy are often collected as part of routine clinical practice (and in the past have tended not to require formal registration) it has been argued that test accuracy studies are more easily conducted and abandoned than RCTs. They may therefore be particularly susceptible to publication bias.[39] Simulation studies have, however, indicated that the effect of publication bias on meta-analytic estimates of the Diagnostic Odds Ratio (DOR) is not likely to be large.[40]

It has been demonstrated that the unique features of the test accuracy study make the application of the Begg, Egger, and Macaskill tests of funnel plot asymmetry potentially misleading.[40] An alternative approach uses funnel plots of (natural logarithm (ln) DOR) vs. (1/√effective sample size) and tests for asymmetry using related regression or rank correlation tests.[40] It should be noted that the power of all statistical tests for funnel plot asymmetry decreases with increasing heterogeneity of DOR. It should also be noted that factors other than publication bias, for example aspects of study quality and population characteristics, may be associated with sample size.

Given the limitations of current knowledge, to ignore the possibility of publication bias would seem unwise, however, its assessment in reviews of test accuracy is complex.

2.2.4 Data extraction

The same precautions against reviewer bias and error should be employed whilst extracting data from test accuracy studies as would be applied in any other type of review. Independent checking of 2x2 data is particularly important, as test accuracy studies are often poorly reported,[12, 41] and the production of a 2x2 table from these studies can be far from straightforward.

Some studies may provide the actual results for each test for individual patients. In this case the researcher may need to classify each patient according to the diagnostic thresholds defined in the review protocol.

Studies may provide categorical data, which may represent multiple categories or stages of disease (as shown in example 1). In this case data will need to be extracted for the numbers of index test positive and negative participants (using the threshold(s) defined in the review protocol, which may include all thresholds reported) with and without the target condition (as defined by the reference standard, using the threshold(s) defined in the review protocol).

Data extraction, example 1

		Reference standard			
		0-19%	20-49%	50-79%	80-100%
Index test	0–19%	5	8	4	1
	20–49%	6	4	5	1
	50–79%	1	9	15	4
	80–100%	2	5	6	10

If the threshold for a positive test in example 1 was 20% (for both index test and reference standard), then the 2x2 data would be extracted as in example 1a.

Data extraction, example 1a

		Reference standard	
		Positive	Negative
Index test	Positive	TP	FP
	Negative	FN	TN

The value for TP is derived by summing the number of participants, from the shaded cells, who have both reference standard and index test results at or above the diagnostic threshold (20%), i.e. 4+5+1+9+15+4+5+6+10=59.

The value for FP is derived by summing the number of participants who have a reference standard result below 20%, but an index test result above this threshold, i.e. 6+1+2=9.

The value for FN is derived by summing the number of participants who have a reference standard result at or above 20%, but an index test result below this threshold, i.e. 8+4+1=13.

The value for TN corresponds to the single cell representing participants who have both a reference standard and index test result below 20%, i.e. 5.

		Reference standard	
		**Positive**	**Negative**
**Index test**	**Positive**	59	9
	**Negative**	13	5

If the index test threshold was increased to 50%, with the reference standard remaining at 20% and the same procedure of summing relevant cells applied, then the 2x2 data would be extracted as in example 1b.

Data extraction, example 1b

		Reference standard	
		**Positive**	**Negative**
**Index test**	**Positive**	49	3
	**Negative**	23	11

There may be instances when the raw data are not reported, but 2x2 data can be calculated from reported accuracy measures and total numbers of diseased or non-diseased patients. In example 2, 100 patients underwent an index test and reference standard test. The study reports that the index test correctly identified 20 diseased patients and had a sensitivity of 80% and a specificity of 95%.

Data extraction, example 2

Sensitivity = TP/(TP+FN) = 0.8

Therefore 20/(20+FN)=0.8

20=0.8(20+FN)

20=16 + 0.8FN

0.8FN=20-16=4

FN=4/0.8=5

This gives:

		Reference standard	
		**Positive**	**Negative**
**Index test**	**Positive**	20	
	**Negative**	5	

Therefore, if there were 100 patients in total and 25 were reference standard positive, then 75 were reference standard negative.

Specificity = TN/(TN+FP) = 0.95

TN/75=0.95

TN=75x0.95

TN=71

As there are 100 patients, FP= 100-(20+5+71) = 4, so the final 2x2 table is:

		Reference standard	
		**Positive**	**Negative**
**Index test**	**Positive**	20	4
	**Negative**	5	71

Somewhat more problematic are cases when the data do not 'fit' the 2x2 contingency table model. 'Forcing' data into a 2x2 contingency table, for example by classifying uncertain index test results as FP or FN, may be inappropriate. The contingency table can be extended to form a six cell table, which accommodates uncertain or indeterminate index test results,[42] as shown in *Figure 2.3*.

		Reference standard	
		**Positive**	**Negative**
**Index test**	**Positive**	A	B
	**Indeterminate**	E	F
	**Negative**	C	D

Figure 2.3: The 3x2 contingency table

The informative value of an indeterminate test result can be assessed using an indeterminate likelihood ratio (or LR+/-), defined as the probability of an indeterminate test result in the presence of disease divided by the probability of an indeterminate test result in the absence of disease.[42]

When index test and reference standard give clear results (ie considered determinate), but there is incomplete concordance, the 2x2 table may be expanded to accommodate a more complete clinical picture. In example 3, taken from an analysis of imaging techniques for the localisation of epileptic foci, category E represents those patients for whom the index test and reference standard both detected disease, but differing numbers of foci. Category F represents those patients for whom the index test and reference standard both detected disease, but identified foci in different areas of the brain.[43]

Data extraction, example 3

		Reference standard	
		**Positive**	**Negative**
**Index test**	**Positive**	A	B
	**Negative**	C	D
	**Partially correct**	E	
	**Incorrect**	F	

2.2.5 Quality assessment

Structured appraisal of methodological quality is key to assessing the reliability of test accuracy studies included in a systematic review.[44] Quality assessment should consider the association of individual elements of methodological quality with test accuracy; generating overall 'quality scores' is not recommended.[45]

There are many differences in the design and conduct of diagnostic accuracy studies that can affect the interpretation of their results. Some differences lead to systematic bias such that estimates of diagnostic performance will differ from their true values,

others give rise to variation in results between studies, which can limit applicability. The distinction between bias and variation is not always clear, and quality assessment checklists have tended to include items that are pertinent to both.[46, 47] Sources of variation and bias that are potentially relevant when considering studies of test accuracy are described in *Table 2.1*. Whilst it is clear that variation (e.g. in the demographic characteristics or severity of disease in the study population) can affect the applicability of the results of both individual studies and systematic reviews, there is limited evidence on the effects of design-related biases in primary studies on the results of systematic reviews.[11, 24, 26, 48] Research on the impact of design-related biases is largely a work in progress, being dependent upon the availability of adequate data sets and consistent methods of quality assessment.

Guidelines for assessing the methodological quality of test accuracy studies were first developed in the 1980s.[16, 46] A large number of quality assessment tools and checklists have since been published, often as part of individual systematic reviews. Methodological work has identified 67 tools designed to assess the quality of test accuracy studies and 24 guides to the interpretation, conduct or reporting of test accuracy studies.[49] Only six of the quality assessment tools specified which aspects of quality they aimed to cover.[50-55] One quality assessment tool[46] and one guide to the reporting of diagnostic accuracy studies[56] provided detailed information of how items had been selected for inclusion in the tool, and none reported systematic evaluation of the tool.

QUADAS was the first attempt to develop an evidence-based, validated, quality assessment tool specifically for use in systematic reviews of test accuracy studies.[47] The items included in QUADAS were derived by combining empirical evidence from three systematic reviews, reported in two publications[11, 49] with expert opinion, using a formal consensus method.[47] The QUADAS criteria and the sources of bias and variation to which they relate are given in *Table 2.2*. Each item is scored as 'Yes', 'No' or 'Unclear' and generic guidance on scoring has been published.[47, 57] It is, however, impossible to provide a universally applicable description of how some QUADAS items should be scored, e.g. the definition of 'an appropriate patient spectrum', or 'a reference standard likely to correctly classify the target condition.' It is therefore important that guidance on scoring be refined for individual reviews, with the definition of what should be scored as 'Yes', 'No' and 'Unclear' being specified for each QUADAS item and agreed by the whole review team at the start of the review; this should be done in close consultation with clinical experts.[57] Piloting of the quality assessment process on a small sample of included studies should be done in an attempt to eliminate any discrepancies in understanding between reviewers.

Table 2.1: Sources of bias and variation in test accuracy studies[11]

Source	Bias and/ or variation	Description
**Population**		
Demographic characteristics	Variation	Test may perform differently in different populations.
Disease severity	Variation	Differences in disease severity may lead to different estimates of diagnostic performance.
Disease prevalence	Variation<br>Bias	The prevalence of the target condition varies with the setting and may affect estimates of diagnostic performance. In settings of higher prevalence, interpreters are more prone to classify test results as abnormal (context bias).
Participant selection	Variation	A selection process that may not include a spectrum of patients similar to that in which the test will be used in practice may limit the applicability of study findings.
**Test methods**		
Test execution	Variation	Differences in the execution of the index test and/or reference standard can result in different estimates of diagnostic performance; clear reporting of the methods used is therefore important.
Technological	Variation<br>development	Diagnostic performance of tests can change over time due to technological improvements.
Treatment paradox	Bias	Occurs when treatment is started, based upon the results of one test prior to undertaking the other; thus disease state is potentially altered between tests.
Disease progression	Bias	Occurs when there is sufficient time delay between the application of the index test and the reference standard to allow change in the disease state.
**Application of the reference standard**		
Use of an inappropriate reference standard	Bias	The error in diagnoses derived from an imperfect reference standard can result in underestimation of the performance of the index test.
Differential verification	Bias	Occurs when the diagnosis is verified using different reference standards, depending upon the result of the index test.
Partial verification	Bias	Occurs where only a selected sample of participants undergoing the index test also receive the reference standard.

(Continued)

Source	Bias and/ or variation	Description
**Interpretation (reading process)**		
Test or diagnostic review	Bias	Where interpretation of either the index test or reference standard may be influenced by knowledge of the results of the other test. Diagnostic review bias may be present when the results of the index test are known to those interpreting the reference standard. Test review bias may be present when the results of the reference standard are known to those interpreting the index test.
Clinical review	Bias	The availability of other relevant clinical information (e.g. symptoms, co-morbidities) may also affect estimates of test performance.
Incorporation	Bias	Occurs when the result of the index test is used in establishing the final diagnosis (i.e. it forms part of the reference standard).
Observer	Variation	The interpretation placed upon a test result may vary between observers and this can affect estimates of test accuracy. The reproducibility of a test within (intra) and between (inter) observers affects its applicability in practice.
**Analysis**		
Handling of un-interpretable results	Bias	Diagnostic tests fail or produce un-interpretable results with varying frequency. Study participants for whom a test result could not be obtained are often removed from reported analyses. This may lead to a biased assessment of test performance.
Arbitrary choice of threshold value (the diagnostic threshold is derived from the same data set in which test performance is evaluated)	Variation	The choice of a threshold value based upon that which maximises sensitivity and specificity for the study data may result in exaggerated estimates of test performance. The test may perform less well at the chosen threshold when evaluated in a new independent patient set.

QUADAS is a generic tool, which may be adapted to optimise its usefulness for specific topic areas. Researchers should, therefore, also consider in advance whether all QUADAS items are relevant to their topic area, and whether there are any additional items that are not included in QUADAS.[57] For example, disease progression bias may not be a relevant issue where the clinical course of the target condition is slow; when comparing the performance of imaging tests, or other tests which require subjective interpretation by the operator, the impact of observer variation may need to be considered as variation in test performance with individual operators of the same test (e.g. different individuals conducting and/or interpreting an ultrasound examination) can exceed, and therefore mask, a difference in performance between two different tests (e.g. ultrasound and magnetic resonance imaging).[58, 59]

Table 2.2: The QUADAS items

QUADAS criterion	Bias/variation assessed
Was the spectrum of patients representative of the patients who will receive the test in practice?	Population characteristics (demographic, severity and prevalence of disease)
Were the selection criteria clearly described?	Participant selection
Is the reference standard likely to correctly classify the target condition?	Use of an inappropriate reference standard
Is the time period between reference standard and index test short enough to be reasonably sure that the target condition did not change between the two tests?	Disease progression
Did the whole sample or random selection of the sample receive verification using a reference standard of diagnosis?	Partial verification
Did the patients receive that same reference standard regardless of the index test results?	Differential verification
Was the reference standard independent of the index test?	Incorporation
Was the execution of the index test described in sufficient detail to permit replication?	Test execution
Was the execution of the reference standard described in sufficient detail to permit replication?	
Were the index test results interpreted without knowledge of the results of the reference standard?	Test review
Were the reference standard results interpreted without knowledge of the results of the index test?	Diagnostic review
Were the same clinical data available when the test results were interpreted as would be available when the test is used in practice?	Clinical review
Were un-interpretable/intermediate test results reported?	Handling of un-interpretable or missing results
Were withdrawals from the study explained?	

It is worth noting that the information that can be derived from the quality assessment of test accuracy studies is often limited by poor reporting. Where QUADAS items are scored 'unclear' the researcher cannot be certain whether this indicates poor methods with the attendant consequences for bias/variation, or simply poor reporting of a methodologically sound study. The STARD initiative[60] has proposed standards for the reporting of diagnostic accuracy studies. If these standards are widely adopted and lead to a general improvement in the reporting of test accuracy studies, reviewers will increasingly be able to assess methodological quality rather than the quality of reporting.

2.2.6 Data synthesis

A thorough investigation of heterogeneity should be undertaken before deciding if studies are suitable for combining in a meta-analysis and if so what method to use. Clinical and methodological differences such as patient populations, tests, study design and study conduct, should be considered in addition to statistical variation in the accuracy measures reported by studies. Where a meta-analysis is not considered clinically or statistically meaningful, a structured narrative synthesis can be carried out which can include the presentation of results in one or more graphical formats.[61] For example the results of individual studies can be plotted in ROC space, as in *Figure 2.4*, whether or not a summary curve is included. As well as stratification by index test characteristics, reviews which focus on determining the optimal diagnostic pathway for a condition, rather than the diagnostic performance of a single test, should consider structuring narrative reports to represent the order in which tests would be applied in clinical practice. Reviews which consider differential diagnosis from a common presenting symptom, such as a review of the performance tests to determine the cause of haematuria, should consider stratifying the narrative by target condition with the most common diagnosis addressed first. These approaches aim to increase readability for practitioners and can equally be applied to the structure of reports which include meta-analyses.

2.2.6.1 Assessment of statistical heterogeneity

Threshold effect

A source of heterogeneity unique to test accuracy studies, which requires careful assessment, arises from the choice of the threshold used to define a positive result.[62] Even when different thresholds are not explicitly defined, variation in interpretation by observers may result in implicit variation in threshold. This can be assessed visually using a ROC space plot and statistically by measuring the correlation between sensitivity and specificity. However, statistical tests may be unreliable where studies in a systematic review have small sample sizes; threshold effect may be present but undetected by statistical tests. A ROC space plot is a plot of the 'true positive rate' (sensitivity) from each study against the 'false positive rate' (1 - specificity). If a threshold effect exists then the plot will show a curve (as the threshold decreases the sensitivity will increase and the specificity will decrease). This curve follows the operating characteristics of the test at varying thresholds.

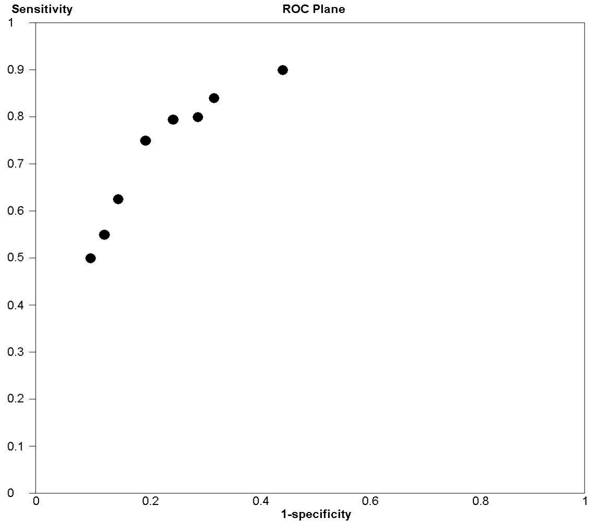

Figure 2.4: ROC space plot

Figure 2.4 clearly shows a curve in the top left hand corner of the plot, indicating the presence of a threshold effect. The presence of a threshold effect can also be investigated using a regression[62] or a hierarchical summary ROC (HSROC) model[63] which are described in more detail in the meta-analysis section below.

Heterogeneity of individual diagnostic accuracy measures

Variability amongst each of the individual measurements (sensitivity, specificity, positive and negative likelihood ratio, and DOR) can be assessed using the same methods as for other study types. Forest plots can be used to visually assess differences between studies, although these will not show any threshold effects. Paired forest plots should be used when illustrating paired outcome measures such as sensitivity and specificity. Use of statistical tests of heterogeneity does not reliably indicate absence of heterogeneity and it is generally advisable to assume the presence of heterogeneity and to fit models which aim to describe and account for it.

2.2.6.2 Meta-analysis

The meta-analysis of diagnostic accuracy studies requires the use of some specific statistical methods which differ from standard methods. Meta-analysis has two main aims: to obtain a pooled measure of diagnostic accuracy and in the case of summary ROC (SROC) models, to explore the heterogeneity amongst studies. Diagnostic accuracy is usually represented by a pair of related measurements, for example: sensitivity and specificity; positive and negative likelihood ratio; and this relationship needs to be incorporated into the analysis methods.

Pooling individual diagnostic accuracy measures

A robust approach to combining data and estimating the underlying relationship between sensitivity and specificity is the construction of an SROC curve. Methods that involve pooling sensitivities and specificities from individual studies, or combining positive and negative likelihood ratios fail to account for the paired nature of the parameters, and should generally be avoided. However, where only one parameter (e.g. sensitivity, but not specificity) is presented, simple pooling of proportions is the only option. Assessment of single parameters is usually inappropriate, but is sometimes used when there is a specific clinical reason why only one parameter should be the focus of interest.

Diagnostic odds ratios can be pooled using standard fixed or random-effects methods for pooling odds ratios. However, these methods do not help estimate average sensitivity and specificity and may produce erroneous results where there is a relationship between DOR and threshold.[64]

Predictive values should not be pooled in meta-analyses as they are affected by the prevalence of disease in the populations of the studies. Overall predictive values are sometimes calculated using estimates of prevalence from the included studies and pooled estimates of likelihood ratios. However, the potentially misleading nature of such estimates should be considered carefully.

Simple methods of estimating summary ROC curves

The Moses-Littenburg regression based method,[62] has been used as a simple method of pooling study results in the presence of a suspected threshold effect. It can be used in preliminary exploratory analyses and is helpful in understanding the data.[65] However, it has limitations and should not be used to obtain summary estimates of sensitivity and specificity. The usual regression model assumptions are not met.[66, 67] It also assumes that there is only one result per study and so cannot deal adequately with studies which have multiple data sets per test (e.g. data for a number of different thresholds).

It is possible to pool ROC curves, or the AUC from individual studies although this is not recommended and would not be practical in the case where some studies reported data for a single threshold and others presented data (or a ROC curve) for a number of thresholds.[21]

Optimal methods of modelling SROC curves

Statistical models, including hierarchical and bivariate models, have been developed for the estimation of SROC curves in the meta-analysis of test accuracy results. The HSROC model[63] accounts for both within- and between-study variation in true positive and false positive rates. The model estimates parameters for the threshold, log DOR and the shape of the underlying ROC curve. It has been shown that it is possible to fit this model using statistical package SAS, and that this method provides results that agree with the more complex Bayesian methods.[68] The HSROC model can be extended to deal with studies that provide results for more than one threshold, but programming is challenging. The bivariate model[67] analyses sensitivity and specificity jointly, therefore retaining the paired nature of the original data (a STATA command function has recently been produced for the bivariate model). The HSROC and bivariate models have been

shown to produce equivalent results in the absence of other study-level covariates.[69] It is recommended that meta-analyses using these models should be undertaken with the assistance of a statistician.

Exploring heterogeneity

Sources of methodological and/or clinical heterogeneity can be explored using subgroup analyses. Ideally subgroups should be planned at the protocol stage. However, where this is dependent upon what data are available, and an adaptive process is needed, this should be stated clearly in the protocol. Results from different groups, for example different tests, or study designs, can be visually assessed by using a ROC space plot with different symbols. *Figure 2.5* illustrates the divergent accuracy results between different study designs from a systematic review of faecal occult blood tests used in screening for colorectal cancer,[15] which indicates that two-gate studies (white circles) overestimate test performance compared with single-gate studies (black circles).

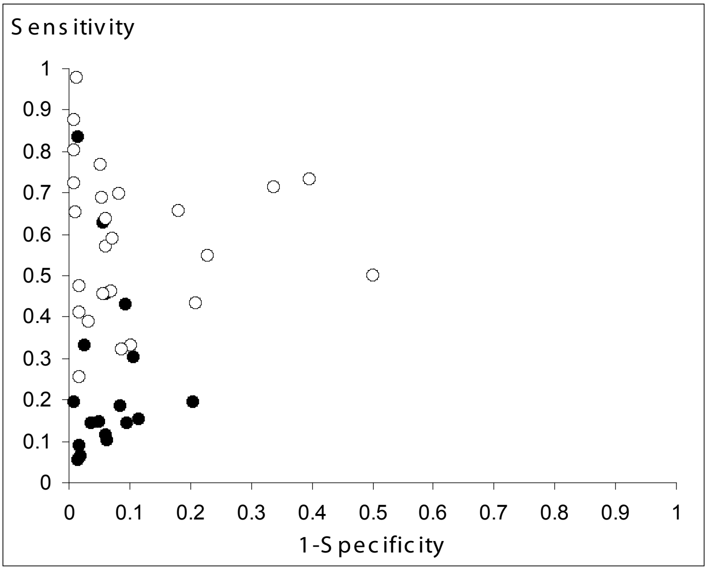

Figure 2.5: ROC space plot using different symbols

HSROC and bivariate models can be used to assess heterogeneity by including covariates. These models allow investigation of the effect of covariates on sensitivity and specificity separately, rather than just the DOR (although this can still be obtained). Further research is needed to determine which SROC models are the most appropriate for the exploration of heterogeneity as the choice of model may depend on which accuracy measure (DOR, sensitivity, specificity) is most affected.[69] An overview of the different methods used to explore heterogeneity in systematic reviews of diagnostic test accuracy is available.[70] It should be noted that, as for meta-regression analyses of other study designs, these analyses are exploratory, can only include covariates reported by

the studies and should not be conducted if there are only a small number of studies (a minimum of 10 studies per covariate is needed). Regardless of the approach used, study-level factors to be examined should be defined in the protocol and aspects of methodological quality, (e.g. QUADAS items) should be considered individually, rather than as overall quality scores.[45, 48]

2.2.6.3 Software

Methods for calculating outcome measures, assessing heterogeneity, producing plots (both with and without summary estimates) and undertaking exploratory analyses using the Moses model are available in a user-friendly form in the Meta-DiSc software (www.hrc.es/investigacion/metadisc_en.html).[71] Systematic reviews of diagnostic accuracy studies have been incorporated in version 5.0 of the Cochrane Review Manager software. More specialist statistical software packages, such as STATA, SAS or WINBUGS, are needed to fit HSROC/bivariate models and the support of a statistician with knowledge of the field is generally recommended.

2.2.7 Presentation of results

When presenting the results of a systematic review of clinical tests it is important to consider how these results will be understood by clinicians and applied in practice. The understanding of and preferences for measures of test performance by clinicians has been the subject of much research and comment.[72-74] The 'best' method remains elusive but some general points, which may improve clarity and aid interpretation, are given below.

The presentation of diagnostic measures should be similar for both narrative and meta-analytic approaches, with graphical representation and/or tabulation of individual study results and additional results presented if meta-analysis was performed. Sufficient detail of the tests, participants, study design and conduct should be presented in tables.[75] The 2 x 2 table results of TP, FP, FN and TN together with sensitivity and specificity, as a minimum should be presented for each study. The choice of accuracy measures presented depends on the aims and anticipated users of the review. Sensitivity and specificity and likelihood ratios are measures of test performance; likelihood ratios may be more useful in a clinical setting as they can be used to calculate the probability of disease given a particular test result, whereas DORs are difficult to interpret clinically.[22] Forest plots or ROC space plots provide useful visual summaries and can be easier to interpret than large tables of numbers. The ranges should be presented when summarising results which have not been subject to meta-analytic pooling. For paired results it may be useful to also present the corresponding measure for the studies at each end of the range, e.g. 'sensitivity ranged from 48% (at a specificity of 80%) to 92% (at a specificity of 70%)'.

If a meta-analysis was undertaken then the presentation of results depends on the methods used. If sensitivity or specificity have been pooled as individual measures then the summary estimate together with the 95% confidence intervals should be presented. If an SROC model has been used then the relevant SROC curve(s) should be presented. Where the performance of a number of index tests is being compared it may

be useful to present multiple SROC curves (or un-pooled data sets) on the same plot. Summary measures of overall diagnostic accuracy, such as AUC or the Q* point (the point on the curve where sensitivity and specificity are equal) may also be presented. However, the relevance of the Q* point is debatable, as its use may lead to summary estimates of sensitivity and specificity outside the values in the original studies.[67] Pairs of sensitivity and specificity values can also be read from the SROC curve and presented as a number of summary points in order to provide an overall description of the curve. The estimated SROC curves should also be presented if HSROC or bivariate models have been used. These models enable the calculation of summary estimates of sensitivity and specificity, which should be reported along with their 95% confidence intervals. Although the use of HSROC or bivariate models to generate summary likelihood ratios is not recommended,[76] where likelihood ratios are considered helpful to interpretation, summary likelihood ratios can be calculated from the pooled estimates of sensitivity and specificity generated by these models. For results from a HSROC or bivariate model, as these retain the paired nature of sensitivity and specificity, a region can be plotted around the summary operating point which represents the 95% confidence intervals of both measures.[67] Confidence interval regions can also be plotted for the results of individual studies, but care is required to ensure that these are not mistakenly interpreted as representations of study weighting. Both models can also be used to plot a prediction region; this is the region which has a particular probability of including the true sensitivity and specificity of a future study.[69]

Summary: Diagnostic studies

- Researchers planning systematic reviews of test accuracy should give careful consideration to context (e.g. is there evidence of a prognostic link between the target condition and preventable morbidity/mortality).
- Diagnostic tests should be evaluated in patients who are representative of those in whom the test will be used in practice; ideally a consecutive or randomly selected series whose diagnosis is unknown at the time of testing.
- Careful consideration should be given to what is the appropriate reference standard to establish diagnosis.
- Difficulties in searching bibliographic databases for test accuracy studies and the lack of suitable methodological search filters mean that more specific searches carry a risk of missing studies. Searches based upon index test and target condition, which are designed to maximise sensitivity, are therefore recommended.
- Test accuracy studies are often poorly reported, hampering data extraction, quality assessment and synthesis.
- Though often unable to provide a definitive estimate of test accuracy, systematic reviews can highlight important gaps in the evidence base and aid in the design of future studies.

2.3 PROGNOSTIC TESTS

Prognostic markers (biomarkers) are characteristics that help to identify or categorise people with different risks of specific future outcomes. They may be simple clinical measures such as body mass index, but are more often pathological, biochemical, molecular or genetic measures or attributes. Identifying those who are or who are not at risk can facilitate intervention choice, and aid patient counselling.

Prognostic research has to date received much less attention than research into therapeutic or diagnostic areas, and an evidence-based approach to the design, conduct and reporting of primary studies of prognostic markers is needed.[77] Reviews have shown that primary prognostic studies are often of poor quality.[78]

Synthesis of prognostic studies is a relatively new and evolving area in which the methods are less well developed than for reviews of therapeutic interventions or of diagnostic accuracy, and available reviews have often been of poor quality.[79-82]

Although numbers of completed prognostic reviews are relatively few,[83] they are becoming more common. Of 294 reviews of prognostic studies published since 1966, almost all have appeared since 1996, occurring most commonly in cancer (15%), musculoskeletal disorders and rheumatology (13%), cardiology (10%), neurology (10%), and obstetrics (10%).[79] Available reviews often include large numbers of studies and patients. For example, some reviews in cancer and cardiovascular disease have reported data on over 10,000 patients for a single marker.[84-87]

This section focuses mainly on reviews of studies of potential prognostic markers and builds on previous work.[88] Given that this is a developing area where methods and approaches will undoubtedly change rapidly, this section presents a discussion rather than firm guidance. Systematic reviews of studies which develop a prognostic model (risk score) are not considered here.

2.3.1 Defining the review question: setting inclusion criteria

Defining the review question and setting inclusion criteria should be approached in the same way as set out in *Chapter 1, Section 1.2 The review protocol*. However, some aspects of methodology require particular attention when planning a systematic review of prognostic studies, and should be considered at an early stage.

2.3.1.1 Population/study design

Patients included in a prognostic study are usually selected as an 'inception' cohort of patients identified very early in the course of their disease, perhaps at diagnosis. Even if the cohort is identified retrospectively, it should be followed forwards in time from a particular point, such as diagnosis or (if relevant) randomisation. The case-control design is liable to bias.[89] Careful thought as to what study designs will be included in the review is needed.

2.3.1.2 Intervention

Although often ignored in prognostic studies, if the intervention that patients receive varies on account of perceived prognosis, this precludes an unbiased assessment of the prognostic ability of a marker (unless alternative interventions are equally effective). Although the intervention effect may be small compared to the effect of important prognostic variables and consequently will have little impact on findings, ideally, prognostic variables should be evaluated in a cohort of patients treated the same way, or that have been included in an RCT.[90, 91] The intervention received is rarely reported in primary studies.

2.3.2 Defining the review question: other considerations

2.3.2.1 Publication bias and sample size

Evidence of publication and associated reporting biases is accumulating for prognostic studies.[92, 93] For example, in a systematic review of studies of a marker Bcl2 in non-small cell lung cancer, almost all the smaller studies showed a statistically significant relationship between Bcl2 and risk of dying, with large hazard ratios, whereas the three large studies were all nonsignificant and showed a much smaller effect.[94] A recent review of the prognostic importance of TP53 status in head and neck cancer showed clearly that published studies had larger effects than unpublished studies.[80] This is in keeping with the belief that epidemiological studies are more prone to publication bias than randomised trials.[80, 95] Publication bias may indeed be worse as many studies are based on retrospective analysis of existing clinical databases, and so in essence they do not really exist until published.

Adequate sample size is equally as important for prognostic studies as for clinical trials, but has received little attention. For example, three quarters of 47 papers reporting prognostic studies in osteosarcoma had fewer than 100 cases.[96] The likely presence of publication bias means that small studies are unreliable and for prognostic reviews there is a good argument for omitting small studies from meta-analysis, for example those with fewer than 100 patients or even 100 events.

Selective reporting of outcomes is also a concern in prognostic studies. For example, in cancer studies the two principal outcomes are time to death (overall survival) and time to recurrence of disease ('disease-free survival'). Many studies, such as in the case-study in *Section 2.3.7*, report only one of these outcomes, which may have been chosen in relation to the findings.

2.3.2.2 Cutpoints

Most markers are continuous measurements. However, it is very common in cancer, and occasionally in other fields, for continuous marker values to be converted to binary variables whereby each patient is characterised as having a high or low value. Dichotomisation is statistically inefficient,[97, 98] but in some fields, notably cancer, it is ubiquitous. Dichotomising does not introduce bias if the split is made at the median or some other pre-specified percentile. However, if the cutpoint is chosen based on analysis

of the data, in particular by splitting at the value which produced the largest difference in outcome between categories, then severe bias will be introduced.[99] Significant findings associated with a data-derived cutpoint will be overoptimistic, perhaps by a large amount. Such studies may best be excluded from any meta-analysis.

Many reports do not state how cutpoints were chosen. When the numbers above and below the cutpoint differ or are not stated, and when the chosen cutpoint is unique to that study, it may be unwise to assume that the choice was made in a valid way.

2.3.2.3 IPD vs summary data

Several authors have noted the considerable advantages of obtaining individual patient data (IPD),[100, 101] and it is clear that IPD could be especially valuable for systematic reviews of prognostic markers. In addition to the usual advantages of IPD over published summary statistics[100] (see *Appendix 1*), there are some specific advantages. Firstly, it may allow inclusion of more studies as not all studies provide the necessary outcome data. Secondly, it allows all data sets to be analysed in a consistent way, which in this case means adjusting for the same variables and using the same analysis method. Thirdly, the marker values can be kept continuous, increasing statistical power and informativeness. Finally, it is possible to conduct analyses restricted to clinical subgroups, for example by stage of disease.

The natural extension of standard systematic reviews would be to try to collect IPD from all identified studies, whether published or not. Although this has been attempted for prognostic studies it has been found to be very time consuming.[102, 103] Concerns about publication bias and the overhead attached to identifying, obtaining and processing each data set have led to the suggestion that for a prognostic meta-analysis of IPD, restriction to only the larger studies or perhaps those carried out in one region[104] would be preferable to one based on summary published data that included every published study.[77]

2.3.3 Identifying research evidence

Identifying prognostic studies is hampered by an absence of standard descriptors and indexing terms. In recent years search strategies have been developed to identify prognostic studies in MEDLINE[105] (see *Box 2.1*) and EMBASE.[106] An improved search strategy for MEDLINE, CINAHL and HealthStar has recently been presented[107] but is as yet unpublished.

2.3.4 Data extraction

Aspects of particular relevance in prognostic studies include recording how the measurements were made (e.g. equipment or assay used), length of follow-up, distribution of the marker, any cutpoints used (with rationale), amount of missing data, methods of statistical analysis, including variables adjusted for, and the number of participants included in the final model.

Box 2.1: Effective MEDLINE searching strategies for studies of prognosis

Best single term
exp epidemiologic studies

The complex search strategy with the highest sensitivity
incidence.sh.
OR exp mortality
OR follow-up studies.sh.
OR prognos:.tw.
OR predict:.tw.
OR course:.tw.

Key
exp denotes exploding the succeeding indexing term
: is a truncation symbol in Ovid
sh denotes searching in the subject headings
tw denotes searching for a textword

A prognostic study with a dichotomous endpoint, such as 30 day mortality after surgery, is statistically no different from a diagnostic accuracy study and poses no additional difficulties for extraction of results. Random-effects endpoints are desirable but there are often difficulties in extracting the log hazard ratio and its standard error from published reports. Guidance on how to estimate these quantities when they are not given explicitly is available.[108]

2.3.5 Quality assessment

The assessment of the appropriateness of the methodology used in the primary studies is a key element of any systematic review, but has been performed in a minority of cases in prognostic systematic reviews.[79, 109] This may reflect the absence of widely agreed criteria for assessing the quality of prognostic studies. Although it is not good practice to use quality as an inclusion criterion, an evaluation of reviews[79] found that this was done in 55/163 (34%) reviews.

Reviews of prognostic studies have demonstrated that generally the methodological quality of included studies is poor. For example, one review which assessed 104 prognostic studies in kidney disease against eight criteria, found that three-quarters of the studies satisfied four or fewer of the eight criteria.[78]

As with other study designs, quality scores are problematic.[48, 110, 111] For example, a quality score was developed which evaluated aspects of study methodology grouped into four main categories: the scientific design; laboratory methodology; the generalisability of the results; and the analysis of the study data.[112] No details were provided of the development of this scoring system, and as it includes elements of both methodology

and reporting it is hard to interpret. Further, for many of the items (e.g. 'source of samples') there is no explanation of the coding scheme. It is preferable to consider specific aspects of methodology related to the risk of bias.

Despite the lack of empirical evidence to support the importance of particular study features affecting the reliability of study findings, especially the risk of bias, theoretical considerations and common sense point to several methodological aspects that are likely to be important.

2.3.5.1 Generic criteria

Table 2.3 lists methodological features that are likely to be important for the internal validity of prognostic studies.[88] The items are not phrased as questions but rather as domains of likely importance. Most authors have presented their checklists as questions. For example, 'Was there a representative and well-defined sample of patients at a similar point in the course of the disease?', taken from a checklist produced by the Evidence-Based Medicine Working Group,[113] is a question that includes three elements from *Table 2.3*. This checklist is widely quoted, for example in a guide for clinicians,[114] but it omits several of the items in *Table 2.3*.

It is generally agreed that to be reliable (and clinically interpretable) a prognostic study requires a well-defined ('inception') cohort of patients at the same stage of their disease, preferably at diagnosis.[115] This also illustrates the more general requirement that the cohort can be clearly described, which is necessary for the study to have external validity.

2.3.5.2 Context-specific criteria

There may also be context-related quality aspects that should be considered in individual reviews. For example, some studies may have used inferior laboratory methods to measure the marker. However, it is important to distinguish aspects of a study that might be a cause of bias, and hence be genuinely a matter of quality, and those that just reflect variation in study conduct but where no bias is likely. Examples of the latter are patient inclusion criteria, length of follow-up, and choice of measuring device or assay kit. Such factors may well be a cause of heterogeneity and it may be prudent to perform separate (subgroup) analyses to investigate whether they are in fact of importance. There are several published checklists for assessing prognostic studies in cancer.[116-118]

2.3.5.3 Implementing quality assessment

Quality assessment in prognostic systematic reviews is often incomplete and there is wide variation in current practice. A review of reviews identified 14 methodological domains grouped within six dimensions relating to the risk of bias of prognostic studies[79] as shown in *Table 2.4*.

Table 2.3: A framework for assessing the internal validity of articles describing prognostic factor studies[88]

Study feature	Qualities sought
Sample of patients	Inclusion criteria defined<br>Sample selection explained<br>Adequate description of diagnostic criteria<br>Clinical and demographic characteristics fully described<br>Representative<br>Assembled at a common (usually early) point in the course of their disease<br>Complete
Follow-up of patients	Sufficiently long
Outcome	Objective<br>Unbiased (e.g. assessment blinded to prognostic information)<br>Fully defined<br>Appropriate<br>Known for all or a high proportion of patients
Prognostic variable	Fully defined, including details of method of measurement if relevant<br>Precisely measured<br>Available for all or a high proportion of patients<br>If relevant, cutpoint(s) defined and justified
Analysis	Continuous predictor variable analysed appropriately<br>Statistical adjustment for all important prognostic factors
Intervention subsequent to inclusion in cohort	Fully described<br>Intervention standardised or randomised

2.3.5.4 Quality of reporting

Assessment of study quality is often seriously hampered by poor reporting of methodological details,[119, 120] as is well known for other types of research. The REporting recommendations for tumour MARKer prognostic studies (REMARK) initiative has proposed guidelines for reporting prognostic studies in cancer, most of which apply to any medical context.[121] Adoption of the REMARK guidelines should lead to improved reporting of prognostic studies.

Table 2.4: System for assessing quality of prognostic factor studies, with proportion of 153 prognostic systematic reviews meeting each item[79]

Potential bias	% reviews adequately assessing bias	Domains addressed	% reviews assessing domain
**1. Study participation**<br>The study sample represents the population of interest on key characteristics, sufficient to limit potential bias to the results	55	1. Source population clearly defined<br>2. Study population described<br>3. Study population represents source population or population of interest	50<br>21<br>50
**2. Study attrition**<br>Loss to follow-up (from sample to study population) is not associated with key characteristics, sufficient to limit potential bias (i.e., the study data adequately represent the sample)	42	4. Completeness of follow-up described<br>5. Completeness of follow-up adequate	19<br>42
**3. Prognostic factor measurement**<br>The prognostic factor of interest is adequately measured	59	6. Prognostic factors defined in study participants to sufficiently limit potential bias<br>7. Prognostic factors measured appropriately	31<br>59
**4. Outcome measurement**<br>The outcomes of interest are adequately measured in study participants to sufficiently limit potential bias	51	8. Outcome defined<br>9. Outcome measured appropriately	42<br>51
**5. Confounding measurement and account**<br>Important potential confounders are appropriately accounted for, limiting potential bias with respect to the prognostic factor of interest	13	10. Confounders defined and measured<br>11. Confounding accounted for	21<br>53
**6. Analysis**<br>The statistical analysis is appropriate for the design of the study, limiting potential for presentation of invalid results	33	12. Analysis described<br>13. Analysis appropriate<br>14. Analysis provides sufficient presentation of data	8<br>33<br>32

2.3.6 Data synthesis

2.3.6.1 Outcome measures

In prognostic studies the focus of interest is what may happen in the future. It is natural, therefore, that most prognostic studies have outcomes that are the time to a specific event, such as death. However, some prognostic studies with dichotomous outcomes may inappropriately ignore the time element. For example, a study looking at death within three years may classify all patients as dead or alive, but those patients who are lost to follow-up before three years (i.e. have censored survival times) cannot be so classified and may be excluded. One exception is studies of prognosis in pregnancy where outcomes often relate to the birth of the baby (e.g. predicting caesarean section or pre-term birth). Such outcomes are genuinely dichotomous and can be analysed in the same way as a study of diagnostic accuracy.

Meta-analysis of time-to event outcomes of aggregate data derived from publications is usually done using the generic inverse-variance approach and may use a fixed effect or random-effects model (see *Chapter 1, Section 1.3.5 Data synthesis*). This type of analysis and extensions have been discussed, as has investigation of heterogeneity in such studies.[122, 123] Although the preferred statistical summary is the hazard ratio (HR) (see *Chapter 1, Section 1.3.5 Data synthesis*) many publications do not report the HR or the information needed to calculate it. Consequently, some of the identified studies cannot be included in the synthesis. Furthermore, non-reporting of appropriate statistical summary measures may be more likely if the marker was found not to be statistically significantly related to outcome, leading to bias. Statistical methods for analysing IPD time-to-event data have been compared,[124] and methods have been published for combining IPD with published summary data.[125]

When all studies have reported data as dichotomous or continuous, meta-analysis may be relatively straightforward. However, if there is a mixture of binary, multi-category, and continuous representation of the same marker, meta-analysis will be problematic and expert input will be advisable. Similar problems have been reported in meta-analysis of epidemiological studies.[126]

In principle researchers may need to combine estimates of a marker that is kept continuous in some studies and dichotomised in others. It is important to note that the hazard ratios for those two cases are not comparable so they should not be combined. There is a related literature on combining data on dose-response relationships in epidemiology.[127-129]

2.3.6.2 Adjustment for other variables

In RCTs the groups being compared are expected to be very similar with regard to prognostic factors (baseline characteristics) through the use of a random sequence of intervention assignment. In non-randomised studies there is no such safeguard and we should expect the groups being compared to differ in various ways. In prognostic studies we are comparing individuals with different levels of a marker, whether binary or continuous. That comparison could easily be biased by other variables that are associated with both the marker and patient prognosis – in other words the comparison may be ‘confounded’.

Furthermore, while it may be of interest to know if a marker considered alone is prognostic, in most cases the real aim of a prognostic marker study should be to ascertain if the marker adds useful clinical information to what is already known. In many clinical contexts much is already known about prognosis, and it is important to know whether the new marker offers additional prognostic value over and above that achieved with previously identified prognostic variables. As an example, a study examined the 'incremental usefulness' of 10 biomarkers for predicting the risk of cardiovascular events, adjusted for age, sex, and conventional risk factors.[130] That approach implies the addition of the marker to a statistical model that includes other known prognostic variables. As well as addressing the most sensible clinical question, adjustment should greatly reduce the risk of confounding.

Dealing with adjustment presents a problem for synthesis, as individual studies are likely to have used different statistical approaches for adjustment and adjusted for different selections of variables. Some syntheses avoid this methodological variation by using unadjusted estimates.[131] While this approach is standard in systematic reviews of RCTs, in prognostic studies it replaces one problem with a worse one; unadjusted analyses are likely to be biased. Although the unadjusted estimate provides the maximum opportunity for comparison of consistent estimates across studies,[131] it is important to adjust for other prognostic variables to get a valid picture of the relative prognosis for different values of the marker. Prognostic studies thus generally require analysis using multiple regression analysis, although stratification may be useful in simpler situations. For outcomes which are dichotomous or time to a specific event, logistic or Cox proportional hazards regression models respectively are appropriate for examining the influence of several prognostic factors simultaneously. For this purpose, known prognostic factors should preferably not be subjected to a variable selection process. Even though such variables may not reach specified levels of significance in a particular study, they should be included in the models generated in order to compare results to other reported studies. Comparison of models with and without the marker of interest provides an estimate of its independent effect and a test of statistical significance of whether the new marker contains additional prognostic information.

In practice, researchers will often find a mixture of adjusted and unadjusted results. Only 47/129 (36%) of prognostic marker studies in cancer used multivariate modelling in which the marker was added to standard clinical variables.[132] A recent review presented separate meta-analyses of adjusted and unadjusted results of BCL-2 as a protective prognostic marker in breast cancer.[133] It demonstrated, as expected, that the adjusted hazard ratio was lower than the unadjusted value but these differences were small (disease free survival (DFS) HR 1.58 vs HR 1.66). This approach reduces the need for speculation about the value of adjustment, which seems a good strategy even if all studies are then combined.

2.3.6.3 Sensitivity analyses

General considerations of investigating the sensitivity of the review findings to various choices apply equally to reviews of prognostic studies. In the specific context of prognosis, given the evidence about publication bias, it may be advisable to conduct a sensitivity analysis in which smaller studies are excluded.

2.3.7 Case study

An example of a systematic review addressing a prognostic question is given in Box 2.2.[87]

Box 2.2: Case study

Objective
This systematic review of aggregate data obtained from study publications aimed to obtain better quantification of the prognostic importance of Ki-67/MIB-1 expression as a marker of cell proliferation in early breast cancer. Ki-67 is present in all proliferating cells and there is great interest in its role as a marker of proliferation. MIB-1 is a monoclonal antibody against recombinant parts of the Ki-67 antigen.

Inclusion criteria
The review included studies evaluating the relationship between Ki-67/MIB-1 status and prognosis in early breast cancer published by May 2006. Studies had to have been published as a full paper in English. No minimal sample size or minimal median duration of follow-up was defined.

Searching
PubMed was searched using the following keywords: 'breast cancer', 'Ki-67', 'MIB-1', 'proliferative index', 'proliferative marker', 'survival' and 'prognostic'. The authors also screened references from the relevant literature, including all the identified studies and reviews. When the same patient population was reported in more than one publication, only the most recent or complete study was included.

Data extraction
The methods of Parmar et al[134] were used to extract log HR and SE(log HR). Three people independently extracted information from survival curves.

Data availability
Sixty-eight eligible studies were identified of which 46 studies (including 12,155 patients) could be included in meta-analyses; 38 studies for disease free survival and 35 studies for overall survival.

Study characteristics
Table 2.5 shows that there was considerable variation in study characteristics, for example in patient characteristics, cutpoint used to define high Ki-67, and prevalence of raised levels of the marker. All studies dichotomised Ki-67 values. Even studies with the same threshold had prevalence of high values ranging from 11% to 88%. The studies also varied considerably in the interventions patients had received and in the antibody used in laboratory evaluations of Ki-67.

(Continued)

Table 2.5: Systematic review of Ki-67 as a prognostic marker in early breast cancer: excerpt from table of study characteristics and results for disease-free survival (hazard ratios and 95% confidence intervals) [87]

Study	N	Follow-up (median months)	Threshold	Preval-ence	How chosen	HR	95% CI
Bevilacqua, 1996	107	74	10%	88%	arbitrary	2.75	1.02 – 7.39
Bos, 2003	150	106 (mean)	10%	42%	arbitrary	2.47	1.08 – 5.65
Brown, 1996	674	72	5%	25%	optimal cutoff	1.19	0.79 – 1.80
Caly, 2004	244	72 (min)	32%	50%	unclear	1.95	0.92 – 4.14
Domagala (N0), 1996	111	88	10%	60%	median	3.04	1.03 – 8.99
Domagala (N+), 1996	75	88	10%	53%	median	1.38	0.66 – 2.86
Erdem, 2005	47	73	10%	28%	median	17.23	2.42 – 122.4
Fresno, 1997	146	75	10%	58%	arbitrary	1.81	0.71 – 4.59
Gasparini, 1994	165	60	7.5%	50%	mean	2.58	1.21 – 5.49
Gonzalez, 2003	221	103	30%	NR	arbitrary	3.18	1.52 – 6.65
Goodson, 2000	112	61	24%	50%	mean	2.90	1.18 – 7.15
Heatley, 2002	59	60	10%	44%	mean	0.81	0.36 – 1.81
Hlupic (N+), 2004	192	180	10%	61%	arbitrary	1.30	0.80 – 2.11
Jacquemier, 1998	152	60	3.5%	49%	median	3.29	1.49 – 7.22
Jansen, 1998	321	128	7%	48%	median	1.35	1.01 – 1.80
Jensen, 1995	118	104	17%	46%	median	3.41	1.44 – 8.06
Liu, 2001	773	196	17.8%	50%	median	1.76	1.41 – 2.20
Locker, 1992	67	27	9%	34%	tertile	4.19	1.19 – 14.7
Mottolese, 2000	157	60	10%	55%	arbitrary	1.82	0.90 – 3.67
Pellikainen, 2003	414	57	20%	44%	arbitrary	2.56	1.46 – 4.50
Pierga, 1996	136	70	8%	49%	median	1.37	0.64 – 2.91
Pietilainen, 1996	188	103 (mean)	20%	53%	arbitrary	1.88	1.16 – 3.05
Pinder, 1995	177	NR	34%	42%	tertile	1.66	1.09 – 2.52
Pinto, 2001	295	39.6	10%	46%	arbitrary	1.46	0.74 – 2.87
Querzoli, 1996	170	66.5	13%	25%	tertile	2.05	1.11 – 3.77
Railo, 1993	326	32.4 (mean)	10%	11%	unclear	2.39	0.77 – 7.38
Etc							

N: Number of participants; HR: Hazard ratio; CI: Confidence interval

(Continued)

Meta-analysis
Study results were combined using the Peto-Yusuf method. No studies were excluded because of methodological quality but some studies were excluded because suitable data were not available – those included studies which did not provide unadjusted results. Random-effects meta-analyses were used because there was considerable heterogeneity. Separate meta-analyses were performed for overall (OS) and DFS. Both showed a significant association between raised Ki-67 and worse survival: HR 1.93 (95% CI: 1.74 – 2.14) and 1.95 (1.70 – 2.24) respectively. Table 2.5 shows the reported characteristics and the results (HR) for DFS for a subset of the studies.

The 17 omitted studies were included in a sensitivity analysis with no appreciable change to the findings. The authors did not consider possible publication bias.

Conclusions
The authors concluded that 'Despite some limitations, this meta-analysis supports the prognostic role of Ki-67 in early breast cancer, by showing a significant association between its expression and the risk of recurrence and death in all populations considered and for both outcomes, DFS and OS.' They also noted that the reporting of the individual studies was suboptimal and that they had assessed only the univariate prognostic value of Ki-67. They suggested that a prospective study to examine whether Ki-67 was of prognostic importance over and above known factors. Thus, in common with many reviewers of such studies, these authors did not feel that the existing literature was strong enough on which to base clinical decisions.

2.3.8 Systematic review as a driver for improved study quality

Systematic reviews can play a valuable role not just in summarising the findings of published studies but also in drawing attention to the poor and inconsistent methods used. Good systematic reviews are needed to highlight the weaknesses of the evidence base behind prognostic markers and to provide guidance on how better quality studies can be carried out in the future. This is true of prognostic studies and it has been commented that 'one has to question why it is acceptable for tumour marker studies to be performed with less scientific rigor than studies of new pharmaceutical agents.'[135]

As an example, a review of 26 published systematic reviews of prognostic markers in cancer found common deficiencies in both conduct and reporting.[109] Less than 75% of the systematic reviews stated clearly their aims and objectives, the literature search strategy, and the study eligibility criteria. Only 20% reported the final number of primary studies used. Less than 50% of the systematic reviews reported elements of primary study description and analysis, such as sampling methods, cancer stage, cutpoint, and numeric results including CIs and P-values. The exception was the sample size, which was reported in 73% of the systematic reviews. About half of the systematic reviews had carried out a meta-analysis. Of those, some did not include a forest plot or numerical summary with confidence intervals. Most had explored heterogeneity,

but only 66% investigated possible publication/small study bias. Surprisingly, only one group of systematic review investigators assessed the quality of the primary studies.

Summary: Prognostic studies

- Difficulties in searching the literature for prognostic studies mean that there is a higher risk of missing studies than is the case for RCTs.
- Prognostic variables should be evaluated in a representative sample of patients assembled at a common point in the course of their disease. Ideally they should all have received the same medical treatment or have been included in an RCT.
- There is no standard approach for assessing methodological quality. Prognostic studies are frequently found to be methodologically poor.
- Meta-analysis based on published data is often hampered by poor reporting of methods and results and by variation in study and patient characteristics.
- Publication bias is a common problem in studies of prognosis.
- Meta-analysis of prognostic studies using individual patient data can overcome some of the difficulties.
- Systematic reviews can inform the conduct of better primary studies in the future.

REFERENCES

1. Feinstein AR. Misguided efforts and future challenges for research on “diagnostic tests”. *J Epidemiol Community Health* 2002;56:330-2.

2. Limer J, Bossuyt P. Diagnostic testing and prognosis: the randomised controlled trial in diagnostic research. In: Knottnerus J, editor. *The evidence base of clinical diagnosis*. London: BMJ Books; 2002. p. 61-80.

3. Biesheuvel CJ, Grobbee DE, Moons KG. Distraction from randomization in diagnostic research. *Ann Epidemiol* 2006;16:540-4.

4. Bossuyt PM, Lijmer JG, Mol BW. Randomised comparisons of medical tests: sometimes invalid, not always efficient. *Lancet* 2000;356:1844-7.

5. Lord SJ, Irwig L, Simes RJ. When is measuring sensitivity and specificity sufficient to evaluate a diagnostic test, and when do we need randomized trials? *Ann Intern Med.* 2006;144:850-5.

6. Westwood ME, Kelly S, Berry E, Bamford JM, Gough MJ, Airey CM, et al. Use of magnetic resonance angiography to select candidates with recently symptomatic carotid stenosis for surgery: systematic review. *BMJ* 2002;324:198.

7. Westwood ME, Whiting PF, Cooper J, Watt IS, Kleijnen J. Further investigation of confirmed urinary tract infection (UTI) in children under five years: a systematic review. *BMC Pediatr* 2005;5:2.

8. Moons KG, Biesheuvel CJ, Grobbee DE. Test research versus diagnostic research. *Clin Chem* 2004;50:473-6.

9. Moons KG, Grobbee DE. Diagnostic studies as multivariable, prediction research. *J Epidemiol Community Health* 2002;56:337-8.

10. Ransohoff DF, Feinstein AR. Problems of spectrum and bias in evaluating the efficacy of diagnostic tests. *N Engl J Med* 1978;299:926-30.

11. Whiting P, Rutjes AW, Reitsma JB, Glas AS, Bossuyt PM, Kleijnen J. Sources of variation and bias in studies of diagnostic accuracy: a systematic review. *Ann Intern Med* 2004;140:189-202.

12. Smidt N, Rutjes AW, van der Windt DA, Ostelo RW, Reitsma JB, Bossuyt PM, et al. Quality of reporting of diagnostic accuracy studies. *Radiology* 2005;235:347-53.

13. Whiting P, Westwood M, Bojke L, Palmer S, Richardson G, Cooper J, et al. Clinical effectiveness and cost-effectiveness of tests for the diagnosis and investigation of urinary tract infection in children: a systematic review and economic model. *Health Technol Assess* 2006;10:1-172.

14. Burch JA, Soares-Weiser K, St John DJ, Duffy S, Smith S, Kleijnen J, et al. Diagnostic accuracy of faecal occult blood tests used in screening for colorectal cancer: a systematic review. *J Med Screen* 2007;14:132-7.

15. Centre for Reviews and Dissemination. *Diagnostic accuracy and cost-effectiveness of faecal occult blood tests used in screening for colorectal cancer: a systematic review.* CRD Report 36. York: University of York; 2007.

16. Riegelman RK. *Studying a study and testing a test: how to read the medical literature*. 5th ed. Philadelphia, PA: Lippincott, Williams and Wilkins; 2005.

17. Alonzo TA, Pepe MS. Using a combination of reference tests to assess the accuracy of a new diagnostic test. *Stat Med* 1999;18:2987-3003.

18. Hui SL, Zhou XH. Evaluation of diagnostic tests without gold standards. *Stat Methods Med Res* 1998;7:354-70.

19. Rutjes A, Reitsma J, Coomarasamy A, Khan K, Bossuyt P. Evaluation of diagnostic tests when there is no gold standard. A review of methods. *Health Technol Assess* 2007;11:1-51.

20. Knottnerus JA, van Weel C, Muris JW. Evaluation of diagnostic procedures. *BMJ* 2002;324:477-80.

21. Deeks JJ. Systematic reviews of evaluations of diagnostic and screening tests. In: Egger M, Davey Smith G, Altman DG, editors. *Systematic reviews in health care: meta-analysis in context*. 2nd ed. London: BMJ Books; 2001. p. 248-82.

22. Deeks JJ. Systematic reviews in health care: systematic reviews of evaluations of diagnostic and screening tests. *BMJ* 2001;323:157-62.

23. Rutjes AW, Reitsma JB, Vandenbroucke JP, Glas AS, Bossuyt PMM. Case-control and two-gate designs in diagnostic accuracy studies. *Clin Chem* 2005;51:1335-41.

24. Lijmer JG, Mol BW, Heisterkamp S, Bonsel GJ, Prins MH, van der Meulen JH, et al. Empirical evidence of design-related bias in studies of diagnostic tests. *JAMA* 1999;282:1061-6.

25. Pai M, Flores LL, Pai N, Hubbard A, Riley LW, Colford JM. Diagnostic accuracy of nucleic acid amplification tests for tuberculous meningitis: a systematic review and meta-analysis. *Lancet Infect Dis* 2003;3:633-43.

26. Rutjes AW, Reitsma JB, Di Nisio M, Smidt N, van Rijn JC, Bossuyt PM. Evidence of bias and variation in diagnostic accuracy studies. *CMAJ* 2006;174:469-76.

27. Glas AS, Roos D, Deutekom M, Zwinderman AH, Bossuyt PM, Kurth KH. Tumor markers in the diagnosis of primary bladder cancer: a systematic review. *J Urol* 2003;169:1975-82.

28. Sackett DL, Haynes RB. The architecture of diagnostic research. In: Knottnerus JA, editor. *The evidence base of clinical diagnosis*. London: BMJ Books; 2002. p. 19-38.

29. Burch J, Westwood M, Soares-Weiser K. Should data from case-control studies be included in systematic reviews alongside diagnostic cohort studies? [abstract]. In: *14th Cochrane Colloquium*; 2006 Oct 23-26; Dublin, Ireland.

30. Whiting P, Westwood M, Burke M, Sterne J, Glanville J. Systematic reviews of test accuracy should search a range of databases to identify primary studies. *J Clin Epidemiol* 2008;61:357-64.

31. Cochrane Diagnostic Test Accuracy Working Group. *Cochrane handbook for diagnostic test accuracy reviews [in press]*: The Cochrane Collaboration; 2008. Available from: http://srdta.cochrane.org/en/authors.html

32. Cochrane Diagnostic Test Accuracy Working Group. *Cochrane Diagnostic Test Accuracy Working Group [internet]*. Cochrane Collaboration; [cited 2008 18 Jul]. Available from: http://srdta.cochrane.org/en/index.html

33. *Cochrane Central Register of Controlled Trials (CENTRAL) [internet]*. Chichester: John Wiley & Sons Ltd; [cited 2008 05 Aug]. Available from: www.mrw.interscience.wiley.com/cochrane/cochrane_clcentral_articles_fs.html

34. Leeflang MMG, Scholten RJPM, Rutjes AWS, Reitsma JB, Bossuyt PMM. Use of methodological search filters to identify diagnostic accuracy studies can lead to the omission of relevant studies. *J Clin Epidemiol* 2006;59:234-40.

35. Higgins JPT, Green S, (editors). *Cochrane Handbook for Systematic Reviews of Interventions. Version 5.0.0 [updated February 2008]*: The Cochrane Collaboration; 2008. Available from: www.cochrane-handbook.org

36. Ritchie G, Glanville J, Lefebvre C. Do published search filters to identify diagnostic test accuracy studies perform adequately? *Health Info Libr J* 2007;24:188-92.

37. Mitchell R, Rinaldi F, Craig J. *Performance of published search strategies for studies of diagnostic test accuracy (SDTAs) in Medline and Embase*. Oxford: The Cochrane Collaboration; 2005. [cited 2006 14 Sept]. Available from: www.cochrane.org/colloquia/abstracts/melbourne/O-01.htm

38. Smidt N, Deeks J, Moore T, editors. Chapter 4: Guide to the contents of a Cochrane review and protocol for diagnostic test accuracy. In: Cochrane Diagnostic Test Accuracy Working Group, editor. *Cochrane handbook for diagnostic test accuracy reviews [in press]*: The Cochrane Collaboration; 2008. Available from: http://srdta.cochrane.org/en/authors.html

39. Song F, Khan KS, Dinnes J, Sutton AJ. Asymmetric funnel plots and publication bias in meta analyses of diagnostic accuracy. *Int J Epidemiol* 2002;31:88-95.

40. Deeks JJ, Macaskill P, Irwig L. The performance of tests of publication bias and other sample size effects in systematic reviews of diagnostic test accuracy was assessed. *J Clin Epidemiol* 2005;58:882-93.

41. Bossuyt PM. The quality of reporting in diagnostic test research: getting better, still not optimal. *Clin Chem* 2004;50:465-6.

42. Simel DL, Feussner JR, DeLong ER, Matchar DB. Intermediate, indeterminate, and uninterpretable diagnostic test results. *Med Decis Making* 1987;7:107-14.

43. Whiting P, Gupta R, Burch J, Kleijnen J, Marson A, Forbes C. What to do with non 2 x 2 data from a diagnostic systematic review? An example from a review on identifying the seizure focus in patients with epilepsy [abstract]. In: *12th Cochrane Colloquium*; 2004 Oct 2-6; Ottawa, Ontario, Canada.

44. Oosterhuis WP, Niessen RW, Bossuyt PM. The science of systematic reviewing studies of diagnostic tests. *Clin Chem Lab Med* 2000;38:577-88.

45. Whiting P, Harbord R, Kleijnen J. No role for quality scores in systematic reviews of diagnostic accuracy studies. *BMC Med Res Methodol* 2005;5:19.

46. Mulrow CD, Linn WD, Gaul MK, Pugh JA. Assessing quality of a diagnostic test evaluation. *J Gen Intern Med* 1989;4:288-95.

47. Whiting P, Rutjes AW, Reitsma JB, Bossuyt PM, Kleijnen J. The development of QUADAS: a tool for the quality assessment of studies of diagnostic accuracy included in systematic reviews. *BMC Med Res Methodol* 2003;3:25.

48. Westwood ME, Whiting PF, Kleijnen J. How does study quality affect the results of a diagnostic meta-analysis? *BMC Med Res Methodol* 2005;5:20.

49. Whiting P, Rutjes AW, Dinnes J, Reitsma JB, Bossuyt PM, Kleijnen J. A systematic review finds that diagnostic reviews fail to incorporate quality despite available tools. *J Clin Epidemiol* 2005;58:1-12.

50. Lacasse Y, Wong E, Guyatt GH, Cook DJ. Transthoracic needle aspiration biopsy for the diagnosis of localised pulmonary lesions: a meta-analysis. *Thorax* 1999;54:884-93.

51. Berry E, Kelly S, Westwood ME, Davies LM, Gough MJ, Bamford JM, et al. The cost-effectiveness of magnetic resonance angiography for carotid artery stenosis and peripheral vascular disease: a systematic review. *Health Technol Assess* 2002;6:1-155.

52. Chien PF, Khan KS, Ogston S, Owen P. The diagnostic accuracy of cervico-vaginal fetal fibronectin in predicting preterm delivery: an overview. *Br J Obstet Gynaecol* 1997;104:436-44.

53. Metlay JP, Kapoor WN, Fine MJ. Does this patient have community-acquired pneumonia? Diagnosing pneumonia by history and physical examination. *JAMA* 1997;278:1440-5.

54. Khan KS, Dinnes J, Kleijnen J. Systematic reviews to evaluate diagnostic tests. *Eur J Obstet Gynecol Reprod Biol* 2001;95:6-11.

55. Liddle J, Williamson M, Irwig L. *Method for evaluating research and guideline evidence*. Sydney, Australia: NSW Health Department; 1996.

56. Bruns DE, Huth EJ, Magid E, Young DS. Toward a checklist for reporting of studies of diagnostic accuracy of medical tests. *Clin Chem* 2000;46:893-95.

57. Whiting PF, Westwood ME, Rutjes AWS, Reitsma JB, Bossuyt PMM, Kleijnen J. Evaluation of QUADAS, a tool for the quality assessment of diagnostic accuracy studies. *BMC Med Res Methodol* 2006;6:9.

58. Robinson PJ. Radiology's Achilles' heel: error and variation in the interpretation of the Rontgen image. *Br J Radiol* 1997;70:1085-98.

59. Brealey S, Westwood M. Are you reading what we are reading? The effect of who interprets medical images on estimates of diagnostic test accuracy in systematic reviews. *Br J Radiol* 2007;80:674-7.

60. Bossuyt PM, Reitsma JB, Bruns DE, Gatsonis CA, Glasziou PP, Irwig LM, et al. Towards complete and accurate reporting of studies of diagnostic accuracy: the STARD Initiative. *Radiology* 2003;226:24-28.

61. Whiting PF, Sterne JA, Westwood ME, Bachmann LM, Harbord R, Egger M, et al. Graphical presentation of diagnostic information. *BMC Med Res Methodol* 2008;8:20.

62. Moses LE, Shapiro D, Littenberg B. Combining independent studies of a diagnostic test into a summary ROC curve: data-analytic approaches and some additional considerations. *Stat Med* 1993;12:1293-316.

63. Rutter CA, Gatsonis CA. A hierarchical regression approach to meta-analysis of diagnostic test accuracy evaluations. *Stat Med* 2001;20:2865-84.

64. Irwig L, Macaskill P, Glasziou P, Fahey M. Meta-analytic methods for diagnostic test accuracy. *J Clin Epidemiol* 1995;48:119-30.

65. Williams GJ, Macaskill P, Chan SF, Karplus TE, Yung W, Hodson EM, et al. Comparative accuracy of renal duplex sonographic parameters in the diagnosis of renal artery stenosis: paired and unpaired analysis. *AJR Am J Roentgenol* 2007;188:798-811.

66. Schulz KF, Altman DG, Moher D. Allocation concealment in clinical trials. *JAMA* 2002;288:2406-7.

67. Reitsma JB, Glas AS, Rutjes AW, Scholten RJ, Bossuyt PM, Zwinderman AH. Bivariate analysis of sensitivity and specificity produces informative summary measures in diagnostic reviews. *J Clin Epidemiol* 2005;58:982-90.

68. Macaskill P. Empirical Bayes estimates generated in a hierarchical summary ROC analysis agreed closely with those of a full Bayesian analysis. *J Clin Epidemiol* 2004;57:925-32.

69. Harbord RM, Deeks JJ, Egger M, Whiting P, Sterne JA. A unification of models for meta-analysis of diagnostic accuracy studies. *Biostatistics* 2006;1:1-21.

70. Dinnes J, Deeks J, Kirby J, Roderick P. A methodological review of how heterogeneity has been examined in systematic reviews of diagnostic test accuracy. *Health Technol Assess* 2005;9:1-128.

71. Zamora J, Abraira V, Muriel A, Khan K, Coomarasamy A. Meta-DiSc: a software for meta-analysis of test accuracy data. *BMC Med Res Methodol* 2006;6:31.

72. Bachmann LM, Steurer J, ter Riet G. Simple presentation of test accuracy may lead to inflated disease probabilities [letter]. *BMJ* 2003;326:393.

73. Puhan MA, Steurer J, Bachmann LM, ter Riet G. A randomized trial of ways to describe test accuracy: the effect on physicians' post-test probability estimates. *Ann Intern Med* 2005;143:184-9.

74. Steurer J, Fischer JE, Bachmann LM, Koller M, ter Riet G. Communicating accuracy of tests to general practitioners: a controlled study. *BMJ* 2002;324:824-6.

75. Mallett S, Deeks JJ, Halligan S, Hopewell S, Cornelius V, Altman DG. Systematic reviews of diagnostic tests in cancer: review of methods and reporting. *BMJ* 2006;333:413.

76. Zwinderman AH, Bossuyt PM. We should not pool diagnostic likelihood ratios in systematic reviews. *Stat Med* 2008;27:687-97.

77. Altman DG, Riley RD. Primer: an evidence-based approach to prognostic markers. *Nat Clin Pract Oncol* 2005;2:466-72.

78. Keough-Ryan T, Hutchinson T, MacGibbon B, Senecal M. Studies of prognostic factors in end-stage renal disease: an epidemiological and statistical critique. *Am J Kidney Dis* 2002;39:1196-205.

79. Hayden JA, Cote P, Bombardier C. Evaluation of the quality of prognosis studies in systematic reviews. *Ann Intern Med* 2006;144:427-37.

80. Kyzas PA, Loizou KT, Ioannidis JP. Selective reporting biases in cancer prognostic factor studies. *J Natl Cancer Inst* 2005;97:1043-55.

81. Clarke M. Systematic review of reviews of risk factors for intracranial aneurysms. *Neuroradiology* 2008;50:653-64.

82. Smith V, Devane D, Begley CM, Clarke M, Higgins S. A systematic review and quality assessment of systematic reviews of fetal fibronectin and transvaginal cervical length for predicting preterm birth. *Eur J Obstet Gynecol Reprod Biol* 2007;133:134-42.

83. Moher D, Tetzlaff J, Tricco AC, Sampson M, Altman DG. Epidemiology and reporting characteristics of systematic reviews. *PLoS Med* 2007;4:e78.

84. Nienhuis MB, Ottervanger JP, Bilo HJ, Dikkeschei BD, Zijlstra F. Prognostic value of troponin after elective percutaneous coronary intervention: a meta-analysis. *Catheter Cardiovasc Interv* 2008;71:318-24.

85. Stuart-Harris R, Caldas C, Pinder SE, Pharoah P. Proliferation markers and survival in early breast cancer: a systematic review and meta-analysis of 85 studies in 32,825 patients. *Breast* 2008;17:323-34.

86. Malats N, Bustos A, Nascimento CM, Fernandez F, Rivas M, Puente D, et al. P53 as a prognostic marker for bladder cancer: a meta-analysis and review. *Lancet Oncol* 2005;6:678-86.

87. de Azambuja E, Cardoso F, de Castro G, Jr., Colozza M, Mano MS, Durbecq V, et al. Ki-67 as prognostic marker in early breast cancer: a meta-analysis of published studies involving 12,155 patients. *Br J Cancer* 2007;96:1504-13.

88. Altman DG. Systematic reviews in health care: systematic reviews of evaluations of prognostic variables. *BMJ* 2001;323:224-8.

89. Altman DG, Bland JM. Time to event (survival) data. *BMJ* 1998;317:468-9.

90. Hudak PL, Cole DC, Haines AT. Understanding prognosis to improve rehabilitation: the example of lateral elbow pain. *Arch Phys Med Rehabil* 1996;77:586-93.

91. Simon R, Altman DG. Statistical aspects of prognostic factor studies in oncology. *Br J Cancer* 1994;69:979-85.

92. Shaheen NJ, Crosby MA, Bozymski EM, Sandler RS. Is there publication bias in the reporting of cancer risk in Barrett's Esophagus? *Gastroenterology* 2000;119:333-8.

93. Popat S, Matakidou A, Houlston RS. Thymidylate synthase expression and prognosis in colorectal cancer: a systematic review and meta-analysis. *J Clin Oncol* 2004;22:529-36.

94. Martin B, Paesmans M, Berghmans T, Branle F, Ghisdal L, Mascaux C, et al. Role of Bcl-2 as a prognostic factor for survival in lung cancer: a systematic review of the literature with meta-analysis. *Br J Cancer* 2003;89:55-64.

95. Kyzas PA, Denaxa-Kyza D, Ioannidis JP. Almost all articles on cancer prognostic markers report statistically significant results. *Eur J Cancer* 2007;43:2559-79.

96. Bentzen SM. Prognostic factor studies in oncology: osteosarcoma as a clinical example. *Int J Radiat Oncol Biol Phys* 2001;49:513-8.

97. Altman DG, Royston P. The cost of dichotomising continuous variables. *BMJ* 2006;332:1080.

98. Royston P, Altman DG, Sauerbrei W. Dichotomizing continuous predictors in multiple regression: a bad idea. *Stat Med* 2006;25:127-41.

99. Altman DG, Lausen B, Sauerbrei W, Schumacher M. Dangers of using "optimal" cutpoints in the evaluation of prognostic factors. *J Natl Cancer Inst* 1994;86:829-35.

100. Stewart LA, Clarke MJ. Practical methodology of meta-analyses (overviews) using updated individual patient data. Cochrane Working Group. *Stat Med* 1995;14:2057-79.

101. Piedbois P, Buyse M. Meta-analyses based on abstracted data: a step in the right direction, but only a first step. *J Clin Oncol* 2004;22:3839-41.

102. Altman DG, Trivella M, Pezzella F, Harris AL, Pastorino U. Systematic review of multiple studies of prognosis: the feasibility of obtaining individual patient data. In: Balakrishnan N, Auget J-L, Mesbah M, Molenberghs G, editors. *Advances in statistical methods for the health sciences: applications to cancer and AIDS studies, genome sequence analysis, and survival analysis*. Boston, MA: Birkhäuser; 2007. p. 3-18.

103. Trivella M, Pezzella F, Pastorino U, Harris AL, Altman DG. Microvessel density as a prognostic factor in non-small-cell lung carcinoma: a meta-analysis of individual patient data. *Lancet Oncol* 2007;8:488-99.

104. Fagard RH, Celis H, Thijs L, Staessen JA, Clement DL, De Buyzere ML, et al. Daytime and nighttime blood pressure as predictors of death and cause-specific cardiovascular events in hypertension. *Hypertension* 2008;51:55-61.

105. Wilczynski NL, Haynes RB. Developing optimal search strategies for detecting clinically sound prognostic studies in MEDLINE: an analytic survey. *BMC Med* 2004;2:23.

106. Wilczynski NL, Haynes RB. Optimal search strategies for detecting clinically sound prognostic studies in EMBASE: an analytic survey. *J Am Med Inform Assoc* 2005;12:481-5.

107. Irvin E, Hayden JA. Developing and testing an optimal search strategy for identifying studies of prognosis [abstract]. In: *14th Cochrane Colloquium*; 2006 Oct 23-26; Dublin, Ireland.

108. Tierney JF, Stewart LA, Ghersi G, Burdett S, Sydes MR. Practical methods for incorporating summary time-to-event data into meta-analysis. *Trials* 2007;8:16.

109. Trivella MH. *Systematic reviews of prognostic factor studies* [DPhil]. University of Oxford; 2005.

110. Greenland S. Quality scores are useless and potentially misleading. Reply to "re: a critical look at some popular analytic methods". *Am J Epidemiol* 1994;140:300-1.

111. Jüni P, Altman DG, Egger M. Systematic reviews in health care: assessing the quality of controlled clinical trials. *BMJ* 2001;323:42-6.

112. Steels E, Paesmans M, Berghmans T, Branle F, Lemaitre F, Mascaux C, et al. Role of p53 as a prognostic factor for survival in lung cancer: a systematic review of the literature with a meta-analysis. *Eur Respir J* 2001;18:705-19.

113. Laupacis A, Wells G, Richardson WS, Tugwell P. Users' guides to the medical literature. V. How to use an article about prognosis. Evidence-Based Medicine Working Group. *JAMA* 1994;272:234-7.

114. Straus SE, McAlister FA. A clinician's guide to journal articles about prognosis. *ACP J Club* 1999;130:A13-A15.

115. Kernan WN, Feinstein AR, Brass LM. A methodological appraisal of research on prognosis after transient ischemic attacks. *Stroke* 1991;22:1108-16.

116. Levine MN, Browman GP, Gent M, Roberts R, Goodyear M. When is a prognostic factor useful? A guide for the perplexed. *J Clin Oncol* 1991;9:348-56.

117. Marras LC, Geerts WH, Perry JR. The risk of venous thromboembolism is increased throughout the course of malignant glioma: an evidence-based review. *Cancer* 2000;89:640-6.

118. Melnikow J, Nuovo J, Willan AR, Chan BK, Howell LP. Natural history of cervical squamous intraepithelial lesions: a meta-analysis. *Obstet Gynecol* 1998;92:727-35.

119. Carson CA, Fine MJ, Smith MA, Weissfeld LA, Huber JT, Kapoor WN. Quality of published reports of the prognosis of community-acquired pneumonia. *J Gen Intern Med* 1994;9:13-9.

120. Sauerbrei W. Prognostic factors. Confusion caused by bad quality design, analysis and reporting of many studies. *Adv Otorhinolaryngol* 2005;62:184-200.

121. McShane LM, Altman DG, Sauerbrei W, Taube SE, Gion M, Clark GM, et al. REporting recommendations for tumour MARKer prognostic studies (REMARK). *Br J Cancer* 2005;93:387-91.

122. Tudur Smith C, Williamson PR, Marson AG. An overview of methods and empirical comparison of aggregate data and individual patient data results for investigating heterogeneity in meta-analysis of time-to-event outcomes. *J Eval Clin Pract* 2005;11:468-78.

123. Williamson PR, Tudur Smith C, Hutton JL, Marson AG. Aggregate data meta-analysis with time-to-event outcomes. *Stat Med* 2002;21:3337-51.

124. Tudur Smith C, Williamson PR. A comparison of methods for fixed effects meta-analysis of individual patient data with time to event outcomes. *Clin Trials* 2007;4:621-30.

125. Riley RD, Lambert PC, Staessen JA, Wang J, Gueyffier F, Thijs L, et al. Meta-analysis of continuous outcomes combining individual patient data and aggregate data. *Stat Med* 2008;27:1870-93.

126. Chêne G, Thompson SG. Methods for summarizing the risk associations of quantitative variables in epidemiologic studies in a consistent form. *Am J Epidemiol* 1996;144:610-21.

127. Hartemink N, Boshuizen HC, Nagelkerke NJ, Jacobs MA, Van Houwelingen HC. Combining risk estimates from observational studies with different exposure cutpoints: a meta-analysis on body mass index and diabetes type 2. *Am J Epidemiol* 2006;163:1042-52.

128. Shi JQ, Copas JB. Meta-analysis for trend estimation. *Stat Med* 2004;23:3-19.

129. Key J, Hodgson S, Omar RZ, Jensen TK, Thompson SG, Boobis AR, et al. Meta-analysis of studies of alcohol and breast cancer with consideration of the methodological issues. *Cancer Causes Control* 2006;17:759-70.

130. Wang TJ, Gona P, Larson MG, Tofler GH, Levy D, Newton-Cheh C, et al. Multiple biomarkers for the prediction of first major cardiovascular events and death. *N Engl J Med* 2006;355:2631-9.

131. Wu YW, Colford JM, Jr. Chorioamnionitis as a risk factor for cerebral palsy: a meta-analysis. *JAMA* 2000;281:1417-24.

132. Vickers AJ, Jang K, Sargent D, Lilja H, Kattan MW. Systematic review of statistical methods used in molecular marker studies in cancer. *Cancer* 2008;112:1862-8.

133. Callagy GM, Webber MJ, Pharoah PD, Caldas C. Meta-analysis confirms BCL2 is an independent prognostic marker in breast cancer. *BMC Cancer* 2008;8:153.

134. Parmar MK, Torri V, Stewart L. Extracting summary statistics to perform meta-analyses of the published literature for survival endpoints. *Stat Med* 1998;17:2815-34.

135. Henry NL, Hayes DF. Uses and abuses of tumor markers in the diagnosis, monitoring, and treatment of primary and metastatic breast cancer. *Oncologist* 2006;11:541-52.

CHAPTER 3

SYSTEMATIC REVIEWS OF PUBLIC HEALTH INTERVENTIONS

3.1 INTRODUCTION **159**

3.2 THE REVIEW QUESTION, SCOPE AND PLANNING **159**

3.2.1 Formulating different types of question **160**

3.2.2 Considerations when applying PICOS **160**

3.2.2.1 Population 160

3.2.2.2 Complex packages of interventions (and comparators) 161

3.2.2.3 Outcomes, surrogates for health and sustainability 162

3.2.2.4 Context 163

3.2.2.5 Study designs to assess effects, processes and implementation 163

3.3 IDENTIFYING RESEARCH EVIDENCE **164**

3.3.1 Database searching **164**

3.3.1.1 Exploring beyond health-related databases 164

3.3.1.2 Dealing with poor terminology and indexing of studies 165

3.3.2 Acknowledging wider data sources and searching techniques **166**

3.3.3 Managing document acquisition **166**

3.4 DATA EXTRACTION **166**

3.4.1 Incorporating diversity in the data extraction form **166**

3.4.2 Dealing with inadequate information **167**

3.5 QUALITY ASSESSMENT **167**

3.5.1 Addressing the quality of the intervention and implementation **167**

3.5.1.1 Has the intervention been appropriately defined? 167

3.5.1.2 Was the intervention delivered as planned? 168

3.6 DATA SYNTHESIS **169**

3.6.1 Developing an appropriate strategy **169**

3.6.2 Handling heterogeneity **169**

3.6.3 Applicability **169**

3.7 REPORTING **170**

REFERENCES **171**

3.1 INTRODUCTION

This chapter provides an overview of issues relevant to systematic reviews of public health interventions. Public health is a broadly defined set of activities that aim to protect, promote, and restore the health of all people. Their success is invariably dependent on health, social, and economic contexts that have a wide reaching and sustainable impact on peoples' lives. Public health interventions are diverse, including those that seek to address change at the individual level (for example, targeting specific attitude or behaviour change), and those that operate with structural (or policy-changing) intent to promote a wider population or community effect often focusing on the social, physical, economic, or legislative context.

Evaluation of public health interventions is usually complex, as multiple interventions, outcomes, participants, settings and stakeholders are often necessary components. Because of the complexity, no single evaluation method is likely to be appropriate and a range of different study designs are used. A framework for the design and evaluation of complex interventions has been proposed which offers guidance on the various phases, including establishing the theoretical basis (mechanisms of action) for the intervention.[1, 2] A sound theoretical base is considered vital to the design of complex interventions and in explaining likely mechanisms for success.[3, 4] However, in practice many interventions and evaluations lack explicit theoretical underpinning.

The complexity of public health research may dictate a process that is far more iterative than in most other types of systematic review, although the use of a conceptual framework for guiding pathways within the systematic review process may offer a way forward (see *Section 3.2, The review question, scope and planning*). Because of the complexity, traditional criteria for producing systematic reviews have been criticised for being too tightly defined, and only partially fulfilling requirements for reviews addressing public health questions.[5-9] As a result, existing guidelines are being updated and expanded.[10]

The aim of this chapter is to identify key issues and challenges specifically related to reviews of public health interventions which researchers need to consider in addition to the core principles presented in *Chapter 1*. A number of these key issues recur throughout the systematic review process, but to avoid repetition are only discussed in *Section 3.2 The review question, scope and planning*. As with all types of systematic review, the need to ensure a systematic approach remains paramount, with focus on critical thought, transparency, and explicitness about methods.

3.2 THE REVIEW QUESTION, SCOPE AND PLANNING

Determining the scope of the review and the subsequent question(s) to be addressed is a critical stage in the review process as decisions made in the early stages will determine both the protocol (see *Chapter 1*) and the subsequent review. A carefully selected advisory group with a range of experiences will help to ensure the questions addressed are those of importance to decision-makers. For reviews of public health interventions, authors might want to consider including practitioners, policy-makers (local or national), funders, and potential recipients or users of services.

The development and use of a conceptual framework to guide the review process may offer a useful starting point. Although there are relatively few examples reported in the literature, one approach that offers particular promise is that developed by the Task Force on Community Preventive Services based in the USA.[11] For each systematic review undertaken, the Task Force develop a logic framework, which is essentially a diagram mapping the hypothesised causal relationships between determinants (environmental, social, biological) and outcomes. The framework is then used to identify links between determinants, outcomes, possible interventions and strategic points to intervene. Once mapped in this way decisions can be made about which interventions to include. The approach has been used to review interventions ranging from early childhood development programmes such as Head Start[12] to affordable family housing.[13]

Other approaches which researchers may find useful are Realist synthesis[14] and The Theory of Change.[15] Realist synthesis, originally designed to work with complex social interventions, outlines the importance of using theory to drive the synthesis of evidence. The Theory of Change is currently used to guide health and social reform programmes in the UK, and is an approach to designing, implementing and evaluating complex interventions, involving multiple stakeholders who work together to map out the pathway for change.

3.2.1 Formulating different types of question

Public health questions are usually broad and multi-faceted, often seeking to address wide policy-based enquiries, where a range of specific interventions exist; examples being the promotion of walking[16] and the reorganisation of shift-work.[17] Specific examples of broad questions include 'what is the evidence that national programmes of urban regeneration improve health?'[18] and 'which health promotion interventions reduce the risk of coronary heart disease in the general population?'[19]

Broad questions can be split to provide a more narrowly focussed enquiry, where immediate decisions of policy relevance are often required.[5] Examples of narrowly focused questions include 'what are the effects of water fluoridation on the incidence of caries?'[20] and 'does over-the-counter nicotine replacement therapy increase abstinence rates?'[21]

3.2.2 Considerations when applying PICOS

The application of PICOS (Population, Interventions, Comparisons, Outcomes, Study Design, *Chapter 1, Section 1.2 The review protocol*) to the review question is relevant to reviews in public health, but adaptation may be required. For example, the importance of context within which the intervention is delivered has prompted the proposed inclusion of an additional C in the acronym (PICOCS).[22]

3.2.2.1 Population

The population of interest is often represented by groups of people, or entire communities, such as young people in schools, users of Healthy Living Centres or

particular geographical regions. This is in contrast to reviews of clinical topics where individuals are usually the focus, for example patients undergoing coronary artery bypass graft surgery or adults diagnosed with moderate to severe psoriasis.

Detail about participants is often lacking.[22] Information about initial and final study populations is not always easy to distinguish, and studies often fail to report levels of engagement, along with the characteristics of those that do and do not participate in the interventions of interest.

Researchers may be especially interested in the effects of interventions on disadvantaged groups in order to investigate the potential for reducing inequalities (see *Section 3.2.2.4 Context*).

3.2.2.2 Complex packages of interventions (and comparators)

Public health interventions are often characterised as a package of components, for example, the inclusion of diet, exercise, and counselling in weight loss programmes or education, community events, and access to nicotine replacement therapy to promote smoking cessation. This type of intervention is often referred to as 'complex', due to the fact that the constituent parts may act both independently and inter-dependently,[1, 2] and defining the 'active ingredient' can be less straightforward than in other topic areas. Where research questions apply specifically to interventions that are part of a multi-component package, it is important to decide on the usefulness of separating the component parts for evaluation. To do so may mean that the essence of the intervention is lost.[23]

The use of theory in guiding the development of complex interventions is considered good practice, due to its potential to predict success and also to explain failure. Theories can explain behaviour and behaviour change at the individual level such as the Theory of Planned Behaviour[24-26] and the Health Belief Model.[27] Theory can also explain change at the organisational or community level. Researchers might decide to only include interventions based on one particular theory, as in a review that focused on Stages of Change,[28] or to include interventions based on different theories and record their theoretical underpinning as part of the description of the intervention, as in a review of HIV prevention.[29] Theory can also be used to group interventions and explore potential differences in effect.

As with populations, interventions (and comparators) are often poorly described. A variety of possible interpretations and terminology is commonplace in the literature (see later in *Section 3.3 Identifying research evidence*). For example, in a systematic review of workplace exercise interventions, the words exercise and physical activity were interpreted differently by researchers and practitioners in terms of the activity being promoted, and there was no standardisation of use in the literature.[30] With the help of the advisory group (see *Chapter 1*), researchers will need to spend time agreeing definitions for the interventions of interest. How the intervention is defined (e.g. in terms of intensity, frequency, duration), delivered, and whether it is sustainable are important indicators in determining effectiveness and these aspects should be considered at the protocol development stage and again later during quality assessment.

It is also important to specify comparators, which might be no intervention, standard practice (or care) or another intervention. Choice of comparator is important, as it will have implications for the interpretation of results.

When framing the review question it may be appropriate to consider broad comparisons, for example the effectiveness of any community intervention for preventing the uptake of smoking versus no intervention, or the effectiveness of any community intervention versus any single component intervention,[31] or all programmes aimed at preventing and treating childhood obesity.[32] This represents a departure from the often well-defined interventions specified in questions about the effects of clinical interventions such as efalizumab for the treatment of psoriasis.

3.2.2.3 Outcomes, surrogates for health and sustainability

Public health interventions often have a range of impacts on those receiving them, some of which will be considered more important than others. Questions about which outcomes to prioritise can be usefully discussed with the advisory group and often it will be necessary to assess a number of different outcomes (or groups of outcomes). In a review focusing on the prevention of smoking in young people,[31] outcomes included the number of cigarettes purchased, membership of anti-smoking clubs, media reach, and level of implementation or exposure to each component of the intervention. Examining why and how the intervention works will require the development of questions addressing process or implementation.[22, 33, 34] (See *Section 3.5 Quality assessment,* and *Chapter 6*). These questions might refer to critical success factors including communication, infrastructure, staff behaviour, and organisational flexibility. As in other types of review, unintended outcomes should also be considered. For example, smoking cessation interventions might reduce the number of cigarettes smoked, but contribute to weight gain.

Although public health interventions have the potential to improve population health overall, improvements (in terms of the total number who benefit from the intervention) may mask differences between groups. For example a review of healthy eating interventions in schoolchildren found differences between males and females in knowledge and consumption of healthy foods.[35] It is possible that a strategy that improves population health, might actually widen inequalities between social groups if benefits are concentrated among the better off.[36] Researchers might want to consider investigating differential outcomes according to varying levels of disadvantage, as in a recent review of school feeding programmes,[37] or programmes that focus solely on disadvantaged populations (for example, the current UK Sure Start initiative). Information on differential outcomes is vital to the development of policy guidance, but is often ignored.

One set of criteria for measuring disadvantage is PROGRESS, which stands for place of residence, race/ethnicity, occupation, gender, religion, education, socioeconomic status and social capital.[38] These criteria have been used in a small number of systematic reviews, including one on population tobacco control interventions,[39] and a modified and extended version (including items on disability, sexual orientation, and age) in a review of school-based cognitive behavioural therapy programmes for preventing/reducing depression.[40, 41] The feasibility, usability and usefulness of the PROGRESS criteria

are being further investigated.[42] The Cochrane Health Equity Field and The Campbell Equity Methods Group have identified other examples of equity relevant reviews, which may be of interest to researchers considering investigating the differential impact of interventions.[42]

Given that many public health interventions involve long-term investment and have long-term outcomes, follow-up assessment is important. Indeed, the degree to which the intervention and its outcomes are sustainable may ultimately tip the balance for decision-makers. Despite this, long-term follow-up is often lacking or simply not feasible. As a consequence, there may be no other option than to work with interim or surrogate outcomes, for example, maximum oxygen uptake (VO^2 Max) as an indicator of cardiovascular health; or measures of attitude and intention as markers for behaviour change). However, researchers should pay particular attention to the validity and reliability of such measures, and to the extent to which they can actually predict the primary outcome(s) of interest.[43]

3.2.2.4 Context

Consideration of the context (e.g. social and political, environmental, and seasonal)[6, 44] in which the intervention is introduced is important. If an intervention is found to be effective it is useful to be able to assess whether context was a contributor. However, boundaries between a particular intervention and the context in which it is delivered are not always easy to identify.

3.2.2.5 Study designs to assess effects, processes and implementation

The choice of study design should be guided by the review question and the needs of the end users. The traditional hierarchy of evidence discussed in *Chapter 1, Sections 1.2 The review protocol* and *1.3.4. Quality Assessment* is relevant to reviews of public health.

Given the greater diversity in conditions and settings, planning to evaluate the effectiveness of public health interventions requires consideration of the influences that might impact upon the success of the intervention. Public health questions often require consolidation of efficacy (does the intervention work under ideal conditions?) and effectiveness (does the intervention work in everyday life?) criteria in the form of 'does the intervention work', in conjunction with 'when, why, and how does it work' (taking account of differences in context, setting or delivery formats) and for 'whom does it work' (with exploration of differential effects amongst groups).

The appropriateness of different study designs for answering a wide range of questions has been summarised.[22] Although the RCT is considered to be the gold standard in establishing whether the intervention works, there are likely to be fewer conducted in public health, especially where policy questions are being addressed and the limitations of sole reliance on data from RCTs has been discussed.[45] Restricting reviews to RCTs and controlled trials may also skew the findings towards particular types of intervention. For example a review of interventions promoting a population shift from using cars to walking or cycling found that RCTs had been used to evaluate only two types of

intervention. Evidence about transport service developments, financial incentives and population wide health promotion activities would have been missed, if other types of study design had been excluded.[46]

The cluster randomised trial design[47] offers promise, however, methodological difficulties with this design (such as the need for a sufficient number of randomised units to ensure even distribution of confounders) suggest that further developments may be required. Others have commented on the difficulties of low power, secular trends and small effect sizes in community-based designs.[48] However, a small effect at the community level may have more practical significance than an effect of comparable size at the individual level.[47] Therefore, small measures of change should not routinely be ignored.

Other designs such as before-and-after, interrupted time series and uncontrolled are often used and should be considered. Regression-discontinuity and matched controlled designs have also been proposed.[49]

The inclusion of other types of study, such as qualitative research and surveys should also be considered as they can help to shape questions of importance to end users, help to understand the mechanisms behind effectiveness or ineffectiveness, contribute to understanding heterogeneous results, identify factors that impact on the implementation of an intervention, describe the experience of people receiving the interventions, and provide participants' subjective evaluations of outcomes. Examples of systematic reviews in public health which have considered a range of evidence in the context of evaluating effectiveness include those focussing on healthy eating,[35, 50] HIV health promotion,[51] and breastfeeding.[52] The inclusion of qualitative evidence is discussed in *Chapter 6*.

The concept of using the best available evidence,[22, 53] similar to the idea of best evidence synthesis,[54] where the desire to include the 'best' evidence does not stand in the way of using the best available evidence, may be the preferred strategy. However, it is important to note that the choice of study designs will impact on the level of complexity in subsequent stages of the review, including searching, quality assessment, and especially synthesis.

3.3 IDENTIFYING RESEARCH EVIDENCE

3.3.1 Database searching

3.3.1.1 Exploring beyond health-related databases

Reviews of public health interventions tend to cut across a number of topic and disciplinary areas, meaning that relevant studies are scattered widely and are unlikely to be identified if only health databases are searched. Databases should be selected according to the question(s) being addressed. In a review of water fluoridation[20] a wide range of databases were searched, including EI Compendex (Engineering Index), PAIS (Public Affairs Information Service), Water Resources Abstracts, and Agricola (Agriculture Online access). Other examples of specialist (non-health) databases are those relating to housing or architecture, such as ICONDA, Waternet, and Enviroline; to modes of transport such as the TRANSPORT database; to education, such as ERIC;

and to geographical data, such as GEOBASE. In an analysis of the sources of studies for a systematic review of interventions to promote walking and cycling, only four of 69 relevant studies were identified from a health database with a significant proportion identified by searching the TRANSPORT database.[55]

Many of the core databases used in medicine are readily available via the Internet, at university and college libraries, or as a result of national agreements between the NHS and database suppliers. Details of how and where to identify relevant databases are given in *Chapter 1, Section 1.3.1 Identifying research evidence for systematic reviews*. Access to some specialist databases such as SportDiscus may be restricted to subscribers or organisational members. Researchers need to consider carefully which databases are appropriate for identifying studies relevant to their specified question, along with any cost implications.

3.3.1.2 Dealing with poor terminology and indexing of studies

In the databases that cover medicine, namely MEDLINE and EMBASE, there are structured and well-recognised thesauri (MeSH and EMTREE) that help to create a focused search strategy. The use of thesaurus terms to index an international literature can compensate for the use of different terms across countries and for variations in spelling. Although the settings of public health interventions are important, for example 'community-based' or 'neighbourhood level' there are no MeSH terms for these specific concepts so the more general heading 'residence characteristics' has to be used.[56] To address this problem the UK Health Development Agency (now part of NICE) and England's Public Health Observatories (PHO's) have developed a unified Public Health Language (PHL) to facilitate interoperability. The thesaurus (available from the Public Health Language website (www.nphl.nhs.uk/uk/default.aspx)) provides a detailed set of terms for indexing documents and records related to public health.

Searching for studies will be greatly enhanced when the use of this language becomes more widespread and especially when it is mapped to MeSH. While the PHL is still being developed, differences in terminology need to be compensated for by using free text terms and synonyms when constructing strategies.

Many of the available databases are poorly indexed or abstracted, and some, such as Midwifery & Infant Care, and PAIS International, do not have a thesaurus. This means there is a greater reliance on using free text terms, which impacts on the sensitivity and specificity of the search.

The greater the number of databases searched the more likely it becomes that different search interfaces will need to be used, with strategies being translated to take account of differing search operators (e.g. the symbols used to indicate truncation of search terms or the adjacency of search terms).

Smaller databases or web-based databases do not routinely allow sophisticated searches to be undertaken so broad strategies need to be used to ensure completeness. Again, this results in a lower precision rate for the search.

3.3.2 Acknowledging wider data sources and searching techniques

Extensive database searching is only part of the solution to identifying relevant research. Studies may appear as books, book chapters, working papers, policy documents or departmental reports, and traditional database searching may not be sufficient to identify these sources. To identify studies published in these formats it is important to supplement database searches with Internet searching, scanning relevant organisational websites (for example, in the UK The Centre for Public Health Excellence, NICE; the EPPI-Centre, University of London; CRD, University of York), contacting experts in the field, and reference checking to minimize publication bias.[57] Searching grey literature sources for unpublished material is a vital part of any search strategy. Handsearching selected journals is helpful in keeping abreast of literature not yet loaded on electronic databases. A technique known as snowballing (citation tracking using the citation databases Science Citation Index, Social Sciences Citation Index, and Arts and Humanities Citation Index) might also be considered.

3.3.3 Managing document acquisition

Retrieval of a large number of articles is likely and this has consequences in terms of resources. For example, a review of tobacco control interventions[58] identified over 17,000 citations of potential relevance. It may be difficult to acquire the identified studies, especially if published as reports or working papers. Individuals and organisations often need to be contacted, so the whole process may take longer than when accessing journal publications.

3.4 DATA EXTRACTION

3.4.1 Incorporating diversity in the data extraction form

As indicated in *Chapter 1, Section 1.3.3 Data Extraction,* the data extraction form should always be designed in line with the research protocol and with the desired output in mind. In general, the form should follow the broad format according to PICOCS, taking account of any necessary adaptations. Given the potential variety and extent of information to be extracted, the design, content and completion of data extraction forms may be more time consuming than in most other types of review. For example, more information may be required on intervention characteristics including theoretical underpinning. It is also important to identify where theoretical information is absent from the primary studies. It is likely that all data on differential effects will be relevant, so adequate detail relating to the population is important.

Examples of potentially relevant data extraction forms are presented in *Box 3.1.* Whilst these can provide a foundation for data extraction, forms should always be developed according to the requirements of the individual review.

Box 3.1: Data extraction forms

- A tool for data collection relating to studies of community prevention services[59]
- The Cochrane Effective Practice and Organisation of Care Review Group (EPOC) data collection checklist (including quality assessment for multiple study designs)[60]
- The Cochrane Non Randomised Studies Methods Group (NRSMG) checklist (to include data extraction and quality assessment of nonrandomised studies www.cochrane.dk/nrsmg/guidelines.html)
- EPPI-Centre data extraction and quality assessment guidelines for health promotion outcome and process evaluations[61]
- The RE-AIM framework to document data on intervention reach, efficacy, adoption, implementation and maintenance[62]

3.4.2 Dealing with inadequate information

Given the potential reporting problems in many primary studies, researchers may need to allow extra time (at the study selection and/or data extraction stage) to make contact with study authors. It is important to record the potential impact of missing data for later discussion on review findings and a designated area of the form is useful for this purpose.

3.5 QUALITY ASSESSMENT

3.5.1 Addressing the quality of the intervention and implementation

The importance of intervention and implementation quality in relation to systematic reviews of complex interventions is raised in *Chapter 1*, *Section 1.3.4 Quality Assessment,* and the discussion is expanded here. A distinction should also be made between the quality of the intervention and quality of the evaluation, the latter being extensively covered in *Chapter 1* and therefore not discussed further here.

The quality of an intervention can be conceptualised as having two main aspects (i) whether the intervention has been appropriately defined, and (ii) whether the intervention was delivered as planned (integrity, or fidelity of the intervention).

3.5.1.1 Has the intervention been appropriately defined?

Theoretical underpinning, use of qualitative research and exploratory studies are important in developing a fully defined intervention. This includes outlining the most likely mechanism of action, and the most appropriate duration and timing of the intervention.[2, 63] Developing the intervention from a needs assessment or piloting

exercise is also likely to be integral to effectiveness.[64] However, in reality, complex interventions are not always developed in this way.

While this aspect of intervention quality is relevant to the appraisal of a primary study, and the subsequent synthesis of studies, it is often not formally assessed. A checklist is available to aid researchers with this task, although further developments have been recommended.[65] Where there is evidence that aspects of an intervention should be administered in a particular way, it is important that this is assessed systematically. As a minimum, this should include the extraction of appropriate information describing the intervention and this information should be considered in the synthesis and interpretation as a possible source of heterogeneity.

3.5.1.2 Was the intervention delivered as planned?

The integrity or fidelity of an intervention refers to the extent to which the intervention has been delivered (or implemented) as planned.[66] If an intervention is not delivered as planned a positive effect is less likely to be found. It is therefore important to distinguish between a failure of implementation and an ineffective intervention.[5, 6] It has been argued that five dimensions – adherence, exposure, quality of delivery, participant responsiveness and programme differentiation – need to be measured to provide a comprehensive picture of integrity/fidelity.[66] In practice however, these aspects are often not assessed and/or reported in primary studies,[6, 33, 66, 67] and this has led to a recommendation for their inclusion in reporting statements such as CONSORT and TREND.[68] A new conceptual framework has also recently been developed to aid understanding of the concept and for measuring the process.[69]

There are few assessment tools available for use in systematic reviews that include items on intervention integrity. However, one such tool recommended by The Cochrane Public Health Review Group[10] is the Effective Public Health Practice Project Quality Assessment Tool for Quantitative Studies (www.city.hamilton.on.ca/PHCS/EPHPP). The Oxford Implementation Index is a new tool to help researchers extract, appraise and use implementation data in systematic reviews. Its applicability to Cochrane reviews of HIV prevention and psychosocial interventions has been demonstrated.[70]

Consideration of whether an intervention was implemented as planned overlaps with the concept of process evaluation, though the latter covers a wider range of activities. Process evaluation (within trials) has been described as an exploration of the implementation, receipt, and setting of an intervention, and helps to interpret the outcome results.[33] It is of particular relevance to public health interventions though it is often not conducted in a formalised way.[6] The methodology for process evaluations embedded within RCTs requires further development,[33] but as knowledge develops it is likely to impact on how such studies are assessed in systematic reviews.

3.6 DATA SYNTHESIS

3.6.1 Developing an appropriate strategy

The choice of synthesis method will ultimately depend on the question(s) addressed and the type of data included. Researchers should refer to *Chapter 1, Section 1.3.5 Data synthesis,* for general guidance on data synthesis. Where the review question relates to whether interventions work in different groups, and centres around dimensions of inequality such as race or ethnic origin, occupation, education, gender and socioeconomic status, synthesis poses particular challenges. Researchers might want to consider a method[71] devised for use in a review of population level tobacco control interventions on social inequalities in smoking.[39] The method combines aspects of the graphical directness of a forest plot (see *Chapter 1, Section 1.3.5.2 Quantitative synthesis of comparative studies*) with a narrative account of what can be learned from a group of very diverse studies. The results from each category of intervention are weighted according to certain methodological criteria and plotted on a matrix (harvest plot). The harvest plot allows best use of all available evidence and provides a visual display, which aids the process of synthesis and the assimilation of findings.[71]

In addition a comprehensive review of approaches to synthesis is available which outlines a range of options, including methods for qualitative evidence, for quantitative data and for both types of evidence. Readers interested in comparing the options available are advised to consult this text.[72]

3.6.2 Handling heterogeneity

Careful judgement is needed when integrating different types of evidence.[73] Indeed, differences in study design, participants, context, and in processes/methods of implementation, theoretical underpinnings, outcomes and outcome measures are major contributions to the complexity, and thus heterogeneity at the synthesis stage.[5] Exploring heterogeneity may be more complex in public health reviews due to mechanisms and interactions being less well developed and not always possible to determine *a priori*.[5] Subgroup analysis can aid the evaluation of differential impacts across groups and in assessing inequalities. Importantly, subgroup analysis can also be used to explore interactions between effects and the quality of the intervention.[74] Available techniques are described in *Chapter 1, Section 1.3.5.2 Quantitative synthesis of comparative studies*.

3.6.3 Applicability

The extent to which public health interventions are expected to work in other contexts can be less predictable than for some clinical interventions. Therefore, it is usually necessary to examine the details of process and context (for example, the mechanics of the intervention and implementation process in relation to the study population, location, and wider environmental influences) before extrapolating the findings from individual studies and any subsequent synthesis.[6] Summarising the results of several studies carried out in different settings and with different populations is in itself a

test of the applicability of findings.[75] If study findings are similar across a range of circumstances then confidence that the findings are transferable is increased. Where effects vary according to setting, population or intervention characteristics, this information is useful for understanding in which circumstances the evidence is likely to be applicable. Although not frequently reported in systematic reviews, applicability (often referred to as external validity or generalisability) is included in some checklists.[76] A tool for assessing applicability and transferability (another term with similar meaning) has been proposed.[77]

3.7 REPORTING

The guidance for report writing discussed in *Chapter 1, Section 1.3.6 Report writing*, is relevant to reviews of public health. However, given the diversity of public health research, and its concern with what works, for whom, why, when, and at what cost, there are likely to be additional reporting requirements, in particular factors impacting on applicability. These include context, development and rationale, implementation process, and sustainability.

Summary: Systematic reviews of public health interventions

- Public health interventions represent a set of activities aiming to protect, promote, and restore the health of all people.
- The evaluation of public health interventions is usually complex.
- Traditional criteria for producing systematic reviews only partially fulfil the requirements for public health interventions.
- Unique challenges to reviewing in this topic area require consideration. These include:
    - Applying an extended version of PICOS, with attention to context and to equity and inequalities
    - Embracing a wider range of data sources and searching techniques
    - Attention to quality assessment beyond traditional measures of methodological adequacy
    - Dealing with diverse data and choosing appropriate methods of synthesis
    - Reporting with additional focus on applicability

REFERENCES

1. Craig P, Dieppe P, Macintyre S, Michie S, Nazareth I, Petticrew M, et al. Developing and evaluating complex interventions: the new Medical Research Council guidance. *BMJ* 2008;337:a1655.

2. Craig P, Dieppe P, Macintyre S, Michie S, Nazareth I, Petticrew M, et al. *Developing and evaluating complex interventions: new guidance*. London: Medical Research Council; 2008. Available from: www.mrc.ac.uk/Utilities/Documentrecord/index.htm?d=MRC004871

3. Tones K, Green J. *Health promotion: planning and strategies*. London: SAGE Publications Ltd; 2004.

4. Crosby RA, Kegler MC, DiClemente RJ. Understanding and applying theory in health promotion practice and research. In: DiClemente RJ, Crosby RA, Kegler MC, editors. *Emerging theories in health promotion practice and research: strategies for public health*. San Francisco, CA: Jossey-Bass; 2002. p. 1-15.

5. Jackson N, Waters E, for the Guidelines for Systematic Reviews of Health Promotion and Public Health Interventions Taskforce. *Guidelines for systematic reviews of health promotion and public health interventions. Version 1.2*. Australia: Deakin University; 2005.

6. Rychetnik L, Frommer M, Hawe P, Shiell A. Criteria for evaluating evidence on public health interventions. *J Epidemiol Community Health* 2002;56:119-27.

7. Boaz A, Ashby D, Denyer D, Egan M, Harden A, Jones DR, et al. A multitude of syntheses: a comparison of five approaches from diverse policy fields. *Evidence & Policy* 2006;2:479-502.

8. Jackson N, Waters E. Criteria for the systematic review of health promotion and public health interventions. *Health Promot Int* 2005;20:367-74.

9. Waters E, Doyle J, Jackson N, Howes F, Brunton G, Oakley A. Evaluating the effectiveness of public health interventions: the role and activities of the Cochrane Collaboration. *J Epidemiol Community Health* 2006;60:285-9.

10. Armstrong R, Waters E, Doyle J, editors. Chapter 21: Reviews in health promotion and public health In: Higgins JPT, Green S, editors. *Cochrane Handbook for Systematic Reviews of Interventions Version 5.0.0* (updated February 2008): The Cochrane Collaboration; 2008. Available from: www.cochrane-handbook.org

11. Briss PA, Zaza S, Pappaioanou M, Fielding J, Wright-De Agüero L, Truman BI, et al. Developing an evidence-based guide to community preventive services--methods. The Task Force on Community Preventive Services. *Am J Prev Med* 2000;18:35-43.

12. Anderson LM, Shinn C, Fullilove MT, Scrimsha SC, Fielding JE, Normand J, et al. The effectiveness of early childhood development programs. A systematic review. *Am J Prev Med* 2003;24:32-46.

13. Anderson LM, Charles JS, Fullilove MT, Scrimshaw SC, Fielding JE, Normand J, et al. Providing affordable family housing and reducing residential segregation by income. A systematic review. *Am J Prev Med* 2003;24:47-67.

14. Pawson R, Greenhalgh T, Harvey G, Walshe K. Realist review: a new method of systematic review designed for complex policy interventions. *J Health Serv Res Policy* 2005;10 Suppl 1:21-34.

15. Coote A, Allen J, Woodhead D. *Finding out what works: understanding complex, community-based initiatives*. London: King's Fund; 2004.

16. Ogilvie D, Foster CE, Rothnie H, Cavill N, Hamilton V, Fitzsimons CF, et al. Interventions to promote walking: systematic review. *BMJ* 2007;334:1204-13.

17. Bambra C, Petticrew M, Whitehead M, Akers J, Sowden A. Shifting schedules: the health effects of reorganising shift work. *Am J Prev Med* 2008;34:427-34.

18. Thomson H, Atkinson R, Petticrew M, Kearns A. Do urban regeneration programmes improve public health and reduce health inequalities? A synthesis of the evidence from UK policy and practice (1980–2004). *J Epidemiol Community Health* 2006;60:108-15.

19. Peersman G, Oliver S, Oakley A. Systematic reviews of effectiveness. In: Oliver S, Peersman G, editors. *Using research for effective health promotion*. Buckingham: Open University Press; 2001. p. 96-108.

20. NHS Centre for Reviews and Dissemination. *A systematic review of public water fluoridation.* CRD Report 18. York: University of York; 2000.

21. Hughes JR, Shiffman S, Callas P, Zhang J. A meta-analysis of the efficacy of over-the-counter nicotine replacement. *Tob Control* 2003;12:21-7.

22. Petticrew M, Roberts H. *Systematic reviews in the social sciences: a practical guide*. Malden, MA: Blackwell Publishing; 2006.

23. Hawe P, Shiell A, Riley T. Complex interventions: how "out of control" can a randomised controlled trial be? *BMJ* 2004;328:1561-3.

24. Ajzen I. From intentions to action: a theory of planned behavior. In: Kuhl J, Baeckman J, editors. *Action control: from cognition to behaviours*. New York, NY: Springer; 1985.

25. Ajzen I. *Attitudes, personality and behavior*. Milton Keynes: Open University Press; 1988.

26. Ajzen I. The theory of planned behavior. *Organ Behav Hum Decis Process* 1991;50:179-211.

27. Becker MH. The health belief model and personal health behavior. *Health Educ Monogr* 1974;2:324–508.

28. Riemsma RP, Pattenden J, Bridle C, Sowden AJ, Mather L, Watt IS, et al. Systematic review of the effectiveness of stage based interventions to promote smoking cessation. *BMJ* 2003;326:1175-7.

29. Underhill K, Operario D, Montgomery P. Abstinence-only programs for HIV infection prevention in high-income countries. *Cochrane Database of Systematic Reviews* 2007, Issue 4. Art. No.: CD005421. DOI: 10.1002/14651858.CD005421.pub2.

30. Dalton JE. *Exploring the potential effectiveness of workplace exercise and physical activity interventions* [PhD thesis]. York: University of York; 2003.

31. Sowden A, Stead L. Community interventions for preventing smoking in young people. *Cochrane Database of Systematic Reviews* 2003, Issue 1. Art. No.: CD001291. DOI: 10.1002/14651858.CD001291.

32. Flynn MA, McNeil DA, Maloff B, Mutasingwa D, Wu M, Ford C, et al. Reducing obesity and related chronic disease risk in children and youth: a synthesis of evidence with 'best practice' recommendations. *Obes Rev* 2006;7 Suppl 7-66.

33. Oakley A, Strange V, Bonnell C, Allen E, Stephenson J, RIPPLE Study Team. Process evaluation in randomised controlled trials of complex interventions. *BMJ* 2006;332:413-6.

34. Roen K, Arai L, Roberts H, Popay J. Extending systematic reviews to include evidence on implementation: methodological work on a review of community-based initiatives to prevent injuries. *Soc Sci Med* 2006;63:1060-71.

35. Shepherd J, Harden A, Rees R, Brunton G, Garcia J, Oliver S, et al. *Young people and healthy eating: a systematic review of research on barriers and facilitators* London: EPPI-Centre, Social Science Research Unit, Institute of Education, University of London; 2001.

36. Macintyre S, Chalmers I, Horton R, Smith R. Using evidence to inform health policy: case study. *BMJ* 2001;322:222-5.

37. Kristjansson EA, Robinson V, Petticrew M, MacDonald B, Krasevec J, Janzen L, et al. School feeding for improving the physical and psychosocial health of disadvantaged students. *Cochrane Database of Systematic Reviews* 2007, Issue 1. Art. No.: CD004676. DOI: 10.1002/14651858.CD004676.pub2.

38. Evans T, Brown H. Road traffic crashes: operationalizing equity in the context of health sector reform. *Inj Control Saf Promot* 2003;10:11-2.

39. Thomas S, Fayter D, Misso K, Ogilvie D, Petticrew M, Sowden A, et al. Population tobacco control interventions and their effects on social inequalities in smoking: systematic review. *Tob Control* 2008;17:230-7.

40. Oliver S, Kavanagh J, Caird J, Lorenc T, Oliver K, Harden A, et al. *Health promotion, inequalities and young people's health: a systematic review of research*. London: EPPI-Centre, Social Science Research Unit, Institute of Education, University of London; 2008.

41. Kavanagh J, Oliver S, Caird J, Tucker H, Greaves A, Harden A, et al. *Inequalities and the mental health of young people: a systematic review of secondary school-based cognitive behavioural interventions*. London: EPPI-Centre, Social Science Research Unit, Institute of Education, University of London; 2008.

42. Cochrane Health Equity Field. *Cochrane Health Equity Field [internet]*. Cochrane Collaboration; [cited 2008 12 May]. Available from: www.equity.cochrane.org/en/index.html

43. Gøtzsche PC, Liberati A, Torri V, Rossetti L. Beware of surrogate outcome measures. *Int J Technol Assess Health Care* 1996;12:238-46.

44. Tilford S. Evidence-based health promotion. *Health Educ Res* 2000;15:659-63.

45. Victora CG, Habicht JP, Bryce J. Evidence-based public health: moving beyond randomized trials. *Am J Public Health* 2004;94:400-5.

46. Ogilvie D, Egan M, Hamilton V, Petticrew M. Systematic reviews of health effects of social interventions: 2. Best available evidence: how low should you go? *J Epidemiol Community Health* 2005;59:886-92.

47. Donner A, Klar N. Pitfalls of and controversies in cluster randomization trials. *Am J Public Health* 2004;94:416-22.

48. Merzel C, D'Afflitti J. Reconsidering community-based health promotion: promise, performance, and potential. *Am J Public Health* 2003;93:557-74.

49. Society for Prevention Research. *Standards of evidence: criteria for efficacy, effectiveness and dissemination*. Falls Church, VA, USA: Society for Prevention Research; 2004.

50. Thomas J, Sutcliffe K, Harden A, Oakley A, Oliver S, Rees R, et al. *Children and healthy eating: a systematic review of barriers and facilitators*. London: EPPI-Centre, Social Science Research Unit, Institute of Education, University of London; 2003.

51. Rees R, Kavanagh J, Burchett H, Shepherd J, Brunton G, Harden A, et al. *HIV health promotion and men who have sex with men (MSM): a systematic review of research relevant to the development and implementation of effective and appropriate interventions*. London EPPI-Centre, Social Science Research Unit, Institute of Education, University of London; 2004.

52. Miller T, Bonas S, Dixon Woods M. Qualitative research on breastfeeding in the UK: a narrative review and methodological reflection. *Evidence & Policy* 2007;3:197-230.

53. Glasziou P, Vandenbroucke J, Chalmers I. Assessing the quality of research. *BMJ* 2004;328:39-41.

54. Slavin RE. Best evidence synthesis: an intelligent alternative to meta-analysis. *J Clin Epidemiol* 1995;48:9-18.

55. Ogilvie D, Hamilton V, Egan M, Petticrew M. Systematic reviews of health effects of social interventions: 1. Finding the evidence: how far should you go? *J Epidemiol Community Health* 2005;59:804-8.

56. Alpi KM. Expert searching in public health. *J Med Libr Assoc* 2005;93:97-103.

57. Beahler CC, Sundheim JJ, Trapp NI. Information retrieval in systematic reviews: challenges in the public health arena. *Am J Prev Med* 2000;18:6-10.

58. Centre for Reviews and Dissemination. *Systematic overview of population tobacco control interventions and their effects on social inequalities in health.* CRD Report 39. York: University of York; 2008.

59. Zaza S, Wright-De Aguero LK, Briss PA, Truman BI, Hopkins DP, Hennessy MH, et al. Data collection instrument and procedure for systematic reviews in the Guide to Community Preventive Services. *Am J Prev Med* 2000;18:44-74.

60. Cochrane Effective Practice and Organisation of Care Review Group. *Data collection checklist*. Ottawa, Canada: Cochrane Effective Practice and Organisation of Care Review Group; 2002.

61. Peersman G, Oliver S, Oakley A. *EPPI-Centre review guidelines: data collections for the EPIC database*. London: EPPI-Centre, Social Science Research Unit, Institute of Education, University of London; 1997.

62. Glasgow RE, Vogt TM, Boles SM. Evaluating the public health impact of health promotion interventions: the RE-AIM framework. *Am J Public Health* 1999;89:1322-7.

63. Medical Research Council. *A framework for development and evaluation of RCTs for complex interventions to improve health*. London: Medical Research Council; 2000.

64. Harden A, Peersman G, Oliver S, Mauthner M, Oakley A. A systematic review of the effectiveness of health promotion interventions in the workplace. *Occup Med (Lond)* 1999;49:540-8.

65. van Driel WG, Keijsers JF. An instrument for reviewing the effectiveness of health education and health promotion. *Patient Educ Couns* 1997;30:7-17.

66. Dane AV, Schneider BH. Program integrity in primary and early secondary prevention: are implementation effects out of control? *Clin Psychol Rev* 1998;18:23-45.

67. Bradley F, Wiles R, Kinmonth A-L, Mant D, Gantley M, for the SHIP Collaborative Group. Development and evaluation of complex interventions in health services research: case study of the Southampton heart integrated care project (SHIP). *BMJ* 1999;318:711-5.

68. Armstrong R, Waters E, Moore L, Riggs E, Cuervo LG, Lumbiganon P, et al. Improving the reporting of public health intervention research: advancing TREND and CONSORT. *J Public Health* 2008;30:103-9.

69. Carroll C, Patterson M, Wood S, Booth A, Rick J, Balain S. A conceptual framework for implementation fidelity. *Implement Sci* 2007;2.

70. Underhill K, Mayo-Wison E, Gardner F, Operario D, Montgomery P. A new tool to incorporate implementation data into systematic reviews: applying the Oxford Implementation Index [abstract]. In: *14th Cochrane Colloquium*; 2006 Oct 23-26; Dublin, Ireland.

71. Ogilvie D, Fayter D, Petticrew M, Sowden A, Thomas S, Whitehead M, et al. The harvest plot: a method for synthesising evidence about the differential effects of interventions. *BMC Med Res Methodol* 2008;8:8.

72. Pope C, Mays N, Popay J. *Synthesizing qualitative and quantitative health evidence: a guide to methods*. Maidenhead: Open University Press; 2007.

73. Mulrow C, Langhorne P, Grimshaw J. Integrating heterogeneous pieces of evidence in systematic reviews. *Ann Intern Med.* 1997;127:989-95.

74. Herbert RD, Bø K. Analysis of quality of interventions in systematic reviews. *BMJ* 2005;331:507-9.

75. Armstrong R, Waters E, Jackson N, Oliver S, Popay J, Shepherd J, et al. *Guidelines for systematic reviews of health promotion and public health interventions.* Version 2. Melbourne University: Australia. October 2007.

76. Deeks JJ, Dinnes J, D'Amico R, Sowden AJ, Sakarovitch C, Song F, et al. Evaluating non-randomised intervention studies. *Health Technol Assess* 2003;7:1-173.

77. Wang S, Moss JR, Hiller JE. Applicability and transferability of interventions in evidence-based public health. *Health Promot Int* 2006;21:76-83.

CHAPTER 4

SYSTEMATIC REVIEWS OF ADVERSE EFFECTS

4.1 INTRODUCTION **179**

4.2 DEFINING THE REVIEW QUESTION **180**

4.2.1 Population **180**

4.2.2 Intervention **180**

4.2.3 Comparators **181**

4.2.4 Outcomes **181**

4.2.5 Study design **182**

4.2.5.1 RCTs 182

4.2.5.2 Observational studies 182

4.2.5.3 Case reports 183

4.2.5.4 Databases 183

4.3 IDENTIFYING ADVERSE EFFECTS EVIDENCE **183**

4.3.1 Search strategy **184**

4.3.2 Potentially useful sources **184**

4.4 DATA EXTRACTION **186**

4.5 QUALITY ASSESSMENT **186**

4.5.1 Quality assessment criteria **186**

4.5.1.1 Is there an adequate explanation of how adverse effects were identified? 187

4.5.1.2 Was a standardised or validated measurement instrument used? 187

4.5.1.3 How was the adverse effect(s) attributed to the intervention? 187

4.5.1.4 Are the terms clearly explained? 188

4.5.2 The quality of the reporting of adverse effects in primary studies **188**

4.5.3 Generalisability **189**

4.6 DATA SYNTHESIS **190**

4.6.1 Consideration of potential sources of heterogeneity **190**

4.6.2 Methods of data synthesis **190**

4.6.2.1 Meta-analysis techniques 190

4.7 REPORTING **191**

REFERENCES **192**

4.1 INTRODUCTION

Therapeutic interventions can have negative (adverse) as well as beneficial effects. Many adverse effects can be explained by the mode of action of the intervention and can therefore be anticipated and explained. Others may be unexpected, occurring only where unique combinations of genetic factors or personality and environment combine. Some adverse effects may be extensions of the expected response to an intervention; for example severe constipation following the use of an anti-diarrhoeal (loperamide). Others may occur where the response to an intervention is unexpectedly negative; for example phocomelia (limb abnormalities) following the use of thalidomide for pregnancy induced nausea.

Health care professionals and patients need information about both intended and unintended effects of an intervention in order to make an informed decision about its adoption.[1-3] Even in the presence of reliable information regarding benefit and harm, decision-making is seldom straightforward.[4]

Most reviews focus on the beneficial effect or clinical effectiveness of an intervention without adequately addressing adverse effects.[5-7] This imbalance may lead to interventions being prescribed or used inappropriately, or patients being harmed by potentially avoidable adverse consequences. Systematic review of adverse effects and consideration of adverse effects within reviews of effectiveness therefore needs to be encouraged. However, this is a relatively new area and methods are still evolving.[8, 9]

Some of the methodological issues have been reported[10, 11] and a survey of 256 reviews of adverse effects identified particular difficulties with search strategies and the evaluation of diverse data sources.[12] One issue of particular importance is that reviews focusing on adverse effects often include studies where harms were of secondary interest. Another issue is the selection of adverse effects on which to focus. Many systematic reviews have attempted to review all adverse effects of an intervention,[10] but this can be problematic given the often large numbers of different associated adverse effects/events some of which may be poorly documented. It is important to balance comprehensiveness against clinical relevance and in practical terms the outcomes chosen should be those that are important in guiding decisions related to the intervention.

The basic principles for carrying out a systematic review, as described in *Chapter 1* also apply to reviews of adverse effects. This chapter focuses on the differences in approach and specific issues related to assessment of the safety and tolerability of an intervention. Given that, to date, much development has been around adverse effects of pharmacological interventions, this chapter reflects this emphasis. However, the principles also apply to other types of intervention, such as surgical procedures and medical devices.

4.2 DEFINING THE REVIEW QUESTION

4.2.1 Population

Interventions are sometimes used to treat a number of different conditions. Any resulting adverse effects may be experienced across conditions or may be population specific. Decisions will need to be made about whether the review will focus on a specific population with a particular diagnosis or whether all patient populations who have received the intervention will be included. For example, a review assessing the effect of statins on cancer risk did not specify why the patients in the studies were taking statins.[19] This was probably not important in this context, but might have been for other adverse effects.

Overall, broader inclusion criteria make the findings more generalisable, whereas narrower inclusion criteria are likely to produce more homogenous results. It is important to consider the trade-off between the two approaches.

4.2.2 Intervention

For pharmacological interventions, reviews may focus on a single drug, all drugs in a class, or even include similar classes of drug. Boundaries will in part depend on the type of intervention and the type of adverse effects being investigated. However it is important to recognise that there can be problems with grouping drugs together in a class; even within a class there can be differences between drugs, and assumptions should be avoided. For example, arising from the clinical problem that selective COX2 inhibitor drugs were suspected of causing cardiovascular adverse effects, a systematic review considered all drugs of this class, together with some nonselective COX2 inhibitors.[13] Importantly, drugs were not all treated as a single intervention. The review found that of the selective COX2 inhibitors, only rofecoxib was associated with an increased risk of cardiovascular events and that one of the older nonselective COX2 inhibitors, diclofenac, may also increase risk.[13] This highlights two important points. Firstly, if all selective COX2 inhibitors had been treated as a single intervention, then differences between the specific drugs would have been missed. Secondly, if only one selective COX2 inhibitor had been included (making the assumption that it was representative of its class) then the wrong conclusions would have been reached.

As for reviews of effectiveness, defining nonpharmacological interventions can be difficult. Surgical procedures and medical devices are frequently modified by surgeons/manufacturers and the researcher has to decide when such modifications might constitute a separate intervention.[14] With surgical techniques, there is the added complexity that even a standardised technique can be performed differently by different surgeons or even by the same surgeon as his/her experience of the technique increases. Complex interventions may be even more difficult to define in the context of potential adverse effects.

4.2.3 Comparators

The appropriate comparator will ideally be placebo or no treatment in order to identify and evaluate those effects which are truly related to the intervention. However, sometimes it is important to explore differences between active treatments. This applies to pharmacological interventions such as selective COX2s or statins and to nonpharmacological treatments such as catheter ablation with or without anticoagulation. A reliable investigation of the differences between active treatments can only be undertaken for specific adverse effects within well-defined populations.

4.2.4 Outcomes

There is sometimes an assumption that all adverse effects should be included. However, such a broad approach can be resource intensive. An analysis of three systematic reviews indicated that two included a broad range of events, but generated a large volume of work and yielded little useful information for decision making.[15] A fully comprehensive review will not always be necessary or feasible and narrowing the focus to a specific adverse effect or class of effects enables researchers to undertake analyses in a systematic, manageable and useful way.[16] This can be achieved by investigating only those adverse effects that are defined by patients or clinicians as the most serious and/or severe and/or troublesome; or are commonly reported, or lead to participant withdrawal from treatment.[16] The important point is that the scope of a particular review should be specified and justified in the protocol. It is also important to consider how to detect adverse effects (i.e. unwanted harms of the intervention) rather than adverse events that have been recorded in clinical studies, but which may not be causally related to the intervention of interest. This is considered further in *Section 4.6 Data synthesis.*

Primary studies that report on adverse events may differ, for example in the definitions of a specific adverse event, severity (or intensity); the reporting (or not) of events of differing degrees of severity; the terminology used to describe similar events (e.g. fatigue, tiredness, aesthenia and lethargy) and for continuous outcomes, the threshold for an 'abnormal' result. The review team will need to decide whether outcomes from different studies are similar enough to group together in the review. If outcomes are grouped it is important to justify the decision.

Attempts have been made to standardise terminology used to describe adverse events by for example, the National Cancer Institute and the World Health Organisation (WHO). The WHO criteria (WHO Adverse Reaction Terminology or WHO-ART; see MedDRA www.umc-products.com) have been widely adopted by regulatory bodies and the pharmaceutical industry. Serious adverse events are defined by the WHO as those that lead to significant medical consequences such as death, disability or hospitalisation. However, primary studies may define serious adverse events differently, for example those that the investigator (or the patient) considers serious. An adverse event may be severe in intensity (as opposed to mild or moderate) without necessarily being serious.[17] Different adverse events may be grouped under a broader classification and it may not be clear which adverse effects are included, for example the term 'minor haemorrhage' recorded in studies of anticoagulants includes nose bleeds, bruises, gum bleeding, and other forms of minor bleeding.

The identification of rare effects can be specified as an objective.[18] However, causality and incidence are unlikely to be adequately addressed by reviewing the published literature. Rare events almost never occur in controlled trials, with their limited participant numbers and follow-up duration. A rate may be estimated from cohort studies, and, if the population is unselected, the rate may be a better estimate than that arising from an RCT on a highly selected population. However, even published observational studies are seldom large enough to provide definitive estimates of incidence. The lack of evidence of a rare adverse effect is therefore not proof that such an adverse effect is not associated with the intervention of interest.

It may be helpful when formulating the review question to think in terms of 'hypothesis generating' or 'hypothesis testing'. Taking a broad approach in specifying the review question, for example by including all adverse effects, could be considered hypothesis generating, whereas hypothesis testing should seek to clarify the statistical nature of the risk, and/or better define the characteristics of the adverse effect by having a more focussed question.

4.2.5 Study design

A range of different study designs can provide useful adverse effects data. For example, RCTs may be appropriate for common, anticipated adverse effects, observational studies may be particularly useful for long-term or rare adverse effects, and post-marketing monitoring data may be useful in detecting previously unknown adverse effects.

4.2.5.1 RCTs

It is generally recognised that RCTs have major limitations as sources of adverse effects/adverse events data. They may not be generalisable having excluded patients at high or even medium risk of experiencing certain adverse effects, or may have only short-term follow-up and relatively small sample sizes.

The way that adverse effects data are collected in RCTs and how they are reported is also a limitation to their usefulness in systematic reviews. A survey found that of 185 RCTs of drug therapy, 14% made no mention of adverse effects, and data in a further 32% could not be fully evaluated.[20]

4.2.5.2 Observational studies

Observational studies, such as controlled or uncontrolled cohort studies or case-control studies or large case series, are likely to provide more participants, longer follow-up and more generalisable data than RCTs. For example, a meta-analysis of RCTs identified only 340 cardiovascular events related to Cox 2 inhibitors,[21] while a review of observational studies identified 60,251 cardiovascular events in nine case-control studies.[13] In another review of the incidence of thrombotic thrombocytopaenic purpura associated with ticlopidine plus aspirin, the only data found were in a single large observational study of 43,322 participants.[7] Post-marketing surveillance studies may be useful sources of adverse events/effects data for pharmaceuticals.

4.2.5.3 Case reports

Published case reports (reports of individual spontaneous occurrences of an adverse effect in clinical practice), may be a useful source as information about adverse effects are frequently reported.[6,22,23] However, just because case reports are abundant and widely used, does not necessarily render them good sources of evidence.

Published case reports have significant limitations,[24] for example they frequently fail to provide important information, including an assessment of likely causal relationship to the intervention being evaluated, and some investigations have concluded that stricter criteria and guidelines for reporting are required.[25, 26] Importantly, case reports cannot provide conclusive evidence; they cannot provide an estimate of incidence, and are usually only hypothesis generating. Although any indication that a treatment might have a significant harmful effect should not be ignored, a balance has to be struck between responding to each report of an adverse effect and trying to eliminate uncertainty before acting.

4.2.5.4 Databases

Arguments have been made for reports of adverse events from drug surveillance databases, hospital databases or general practice databases to be incorporated into systematic reviews.[8] However, it should be noted that analysis of this type of information is more akin to primary research than systematic review; using such sources will require a properly designed study to yield information, see for example the study on the role of paroxitine in suicide.[27]

4.3 IDENTIFYING ADVERSE EFFECTS EVIDENCE

Search methods may vary depending on whether the review focuses on the adverse effects of an intervention or focuses primarily on effectiveness but also addresses adverse effects.

If adverse effects are a secondary outcome, then the way that searches are conducted will depend largely on how the effectiveness searches were carried out. For example, if a search for effectiveness studies included only terms for the population and intervention with no search filters for study design, no terms for the outcomes, and the definition of the population has not changed, then it may be sufficient to scan the results of the effectiveness searches for information on adverse effects. Only a few additional searches may be required in additional specialist sources.

If, for example, the effectiveness searches were limited to RCTs and the adverse effects sought are long-term, rare or unanticipated, then additional searches will be required. These searches would be carried out in the sources already searched, as well as in additional sources specific to adverse effects.

4.3.1 Search strategy

If information on a specific named adverse effect is required, the approach used will be similar to that for a systematic review of effectiveness, using adverse effects as the outcome (see *Appendix 4: Searching for specific adverse effects*). It should be noted, however, that due to the poor reporting and poor indexing of adverse effect data this method will not necessarily retrieve all the relevant papers.[28-30] Alternatively, if all adverse effects for a given intervention are required then a different approach is needed. This is outlined in *Appendix 4: Searching with generic adverse effects terms*. Consideration should be given to whether the adverse effect(s) of interest are common or rare, short or long-term, known or unknown as this will have implications for the choice of study design and therefore the most relevant sources of information and search filters.

4.3.2 Potentially useful sources

Textbooks and bulletins can be a useful starting point when searching for information on adverse effects, particularly of pharmaceuticals. The European Public Assessment Report (EPAR) provides a useful summary of adverse effects of drugs licensed by the European Medicine's Agency (EMEA) as does the Summary of Product Characteristics. The Physician's Desk Reference, Meyler's Side Effects of Drugs, Martindale: The Complete Drug Reference and AHFS (American Hospital Formulary Service) Drug Information. Problems in Pharmacovigilance the Australian Adverse Drug Reactions Bulletin, or Reactions Weekly (via PharmaNewsFeed) are also useful sources.

The main electronic sources for adverse effects data are listed in *Table 4.1*. Potential sources of unpublished information on adverse effects are listed in *Table 4.2.*

Very few evaluations have been carried out of the comparative usefulness of these sources in terms of yield of relevant information. The available evaluations focus on case studies of drug interventions,[30-33] and all indicate that EMBASE retrieves more relevant references than MEDLINE. Some studies suggest there is potential value in searching databases such as Derwent Drug File, [32, 34, 35] and TOXLINE. [30, 31, 36] Searching full-text articles may be particularly advantageous in identifying adverse effects due to the poor reporting of adverse effects in the title and abstracts of articles and therefore the indexing.[28, 37-39] Full-text databases provide access to the full-text of articles and some enable searches of full-text articles.

Table 4.1: Summary of information sources

Electronic sources	Examples
**Full-text databases**<br>Content varies from generic coverage to specialisation in adverse effects or drug information	Iowa Drug Information Service (IDIS), PharmaNewsFeed
**Bibliographic databases**<br>These can be divided into:<br>• those specifically related to adverse effects<br>• those containing a large section of adverse effects information<br>• generic databases that contain information on adverse effects	 <br>• TOXLINE<br>• Derwent Drug File, International Pharmaceutical Abstracts (IPA), Pharmline<br>• MEDLINE, EMBASE
**Referenced summary databases**<br>Most specialise in drug information	Drugdex, Reprorisk, Poisindex, XPharm
**Internet reference collections**<br>Most are topic related, and contain useful bibliographies or reference lists	Organization of Teratology Information Specialists, Motherisk (safety of drugs during pregnancy)
**Spontaneous reporting systems**<br>Some information is free on the internet, most useful data are available for a fee through databases or requests services	Canada's Adverse Drug Reaction Database, UK Drug Analysis Prints (DAPS), DIOGENES, Vigibase Services

Table 4.2: Unpublished data sources

Unpublished information	Examples
**Authors**<br>May be authors of efficacy studies or adverse effects studies	Trialists, researchers
**Industry**<br>Drug companies or manufacturers of medical devices<br><br>In many countries, manufacturers of drugs have a regulatory requirement to monitor the adverse effects of their drugs. However such data may be classed as commercially sensitive and therefore not accessible, and even if obtained, can be extremely detailed and difficult to navigate	GlaxoSmithKline www.gsk-clinicalstudyregister.com/<br>International Federation of Pharmaceutical Manufacturers and Associations (IFPMA) clinical trials portal (http://clinicaltrials.ifpma.org/no_cashe/en/myportal/index.htm)<br>Food and Drug Administration (FDA) www.fda.gov/ [40]<br><br>Comprehensive clinical trials reports (CCTRs) produced by or for manufacturers

4.4 DATA EXTRACTION

Data extraction should include frequency, severity, seriousness of the event and withdrawals from treatment because of adverse events.

The way in which adverse events were monitored or recorded may affect the reported frequency and so should also, if possible, be extracted. For example, noting whether the data were collected at follow-up (and if so how frequently), and whether collected by patient diary or checklist, or relied on spontaneous reporting. In studies using patient diaries, adverse effects were found to be significantly more common in the active treatment than control groups.[41] However, when adverse effects were assessed by direct questioning, spontaneous reporting, or where the method was not reported, there were no significant differences between groups or methods of assessment.

Withdrawals and drop-outs should be extracted where possible, together with the reason if known. However, withdrawals and drop-outs are not reliable surrogates for safety or tolerability; withdrawals may be for other reasons than adverse events, for example unpleasant or inconvenient study procedures, lack of improvement or earlier than expected recovery.[42] Alternatively, investigators often make considerable efforts to keep withdrawals low[43] and this can result in withdrawal rates lower than the 'true' rate that would be seen in clinical practice.

Data extraction is frequently complicated by poor reporting. For example, some studies only report the number of adverse events, not the number of patients with adverse events. This can lead to problems at the data synthesis stage. It is possible to derive the number of patients from the number of adverse events or use the number of adverse events.[44] However this is controversial as it may over or underestimate the rate of adverse events.[45]

4.5 QUALITY ASSESSMENT

In studies assessing both effectiveness and adverse effects it is important to recognise that there might be differences in the quality of data relating to each type of outcome. Such differences are commonly related to the sample size and study duration, which may be adequate for the primary efficacy/effectiveness variable but not for adverse effects.[15, 42] Also the collection of data may be different: for example the efficacy of an intervention may be studied in a placebo controlled RCT but the adverse effects data may be collected retrospectively when the assigned treatment is known.[46]

4.5.1 Quality assessment criteria

Criteria for assessing the quality of adverse effects data are being developed[8, 11] and some checklists already exist such as the checklist for assessing quality of RCTs, cohort studies and uncontrolled surgical series that reported adverse effects.[68] The majority of these have not been validated[11, 42, 49, 59] and none are suitable for all purposes. However, different criteria should be used for different study designs, and the criteria should have been validated by empirical evidence wherever possible.[11]

Efforts have been made to develop a standard scale for assessing the quality of different types of study design,[69-72] but there is currently no consensus on how to incorporate information about quality from a range of study designs within a systematic review.[15] Researchers need to be clear about what they require from quality assessment and perform the assessment accordingly.[15]

4.5.1.1 Is there an adequate explanation of how adverse effects were identified?

A variety of measurement instruments may be used to identify adverse effects. Examples include active or passive surveillance, questionnaire derived data, clinical laboratory and pharmacokinetic and pharmacodynamic data. The choice of instrument can significantly affect the identification, measurement and reporting of adverse effects.[22, 47] For example, active surveillance may provide more reliable estimates than passive surveillance and even with active surveillance, a prospective method may yield more accurate information than a retrospective method.[48] The strengths and limitations of these methods have been described elsewhere.[11, 49] In assessing study quality, it is important to consider how adverse effects were identified and reported for each data source used. For example, were the adverse effects assessed independently by someone other than the surgeon performing the procedure? Are the measurement instruments described? Is the timing and duration of follow-up reported?

4.5.1.2 Was a standardised or validated measurement instrument used?

There are a number of standardised instruments for reporting adverse effects.[50-58] For example the National Cancer Institute[54] and the National Institute of Allergy and Infectious Diseases[59] have introduced guidance on assessing the severity of adverse effects. Similarly, the Brighton Collaboration has developed guidelines for reporting adverse effects following immunisation.[52] The use of nonstandardised and nonvalidated scales is also very common,[42] and it is important to specify whether included studies used a standardised or validated scale to report adverse effects.

4.5.1.3 How was the adverse effect(s) attributed to the intervention?

Studies should make clear how they identified that the harm was related to the intervention, who made the attribution (e.g. investigators, participants, sponsors or a combination of these) and whether the process was blinded to assigned treatment.[42] Establishing the causal link between the intervention and harms may be particularly difficult in case reports, and the following criteria may be helpful for establishing causality: temporal relationship, lack of alternative causes, and for interventions that are given repeatedly over time, response to discontinuation (dechallenge), dose-response relationship and response to repeat exposure (rechallenge).[11] For drug interventions the presence of toxic concentrations of a drug may also indicate a causal relationship.

4.5.1.4 Are the terms clearly explained?

A variety of terms are used to identify adverse effects, with some of the terms remaining ill-defined and the boundaries between them not clearly described.[42, 49] The lack of standard and well-constructed terms and a set of definitions has led to real difficulties in comparing adverse effects between studies.[20] To make accurate and reliable comparisons between, or synthesis of, studies it is important that clear and well-constructed terms and definitions are used. In particular the severity of adverse effects should be adequately defined by either detailed description of severity or reference to a known scale of severity.[60]

4.5.2 The quality of the reporting of adverse effects in primary studies

Currently there is no agreed standard about what and how information is recorded and reported,[61] and hence no consistency in the data available for extraction.[62] The CONSORT statement has been extended in an effort to improve reporting of harms from RCTs,[42] but this is likely to be of limited value as data on adverse events are often derived from studies other than RCTs. Some of the criteria in the extension to the CONSORT statement are relevant to absolute quality and others are important for enabling adverse effects information to be synthesised and compared across studies.

Ideally, study results should be reported according to the methods section of the study protocol.[63] The number of each type of adverse effect should be reported for each study arm. The timing of events should also be reported, particularly when the follow-up period is prolonged. Many RCTs are large enough to evaluate the beneficial effects of the intervention, but the majority are not adequately powered to detect statistically significant differences for most adverse effects, except very common ones.[6, 48] This may lead to overinterpreting the absence of adverse effects especially when the sample size is small.[48] Serious and life-threatening adverse effects in particular should be described separately for each type of event.[48] If no adverse effect of a specific type or severity occurred this should be stated.

The report should specify the number of patients withdrawn from the study because of adverse effects by study arm and by type of adverse effects and detail who decided to withdraw (participant or physician) and whether attribution was blinded to the assigned treatment.[42, 48] Such information is often not well reported. A systematic review of published RCTs on treatments for HIV infection found that although 82% of trials reported how many patients discontinued the study treatment, reasons were stated in only 38%.[64] A further systematic review found that 75% of trials in seven medical areas reported the number of discontinuations due to toxicity per study arm, but specific reasons were given in only 46%.[60] Studies with prolonged follow-up should report the timing of withdrawals as the causes of early withdrawals may differ from late withdrawals.

Sometimes more than one adverse effect may occur in a patient but this is not always reported clearly. It is most helpful when both the number of affected participants and the total number of adverse effects are reported, with the denominators and incidence or prevalence rates.[42]

Intention-to-treat is usually the recommended method of analysis (see *Chapter 1 Section 1.3.5 Data synthesis*). It may however, underestimate adverse effects particularly when there is a high rate of nonadherence with allocated treatment.[42] 'On treatment' or 'per protocol' analysis, in which adverse effects are only considered in those patients who have received the intervention, are probably more appropriate.

There are a number of factors associated with bias in the reporting of adverse effects data. It is important to be clear about whether a paper includes all adverse effects that occurred or just a selected sample.[42] If a subset is reported it should be clear how, why and who made the selection. It should also be clear whether the selection was made on the basis of frequency (e.g. common events), severity, seriousness, or biological relevance to the intervention.[65] Clinical trials have been found to report adverse effects without distinction of severity and this may hinder accurate comparison of adverse effects between studies.[60] If severity and seriousness are not considered then the synthesis may be flawed. Where critical or otherwise significant adverse effects have been reported they should ideally have been investigated and the findings included in the report.[8]

Which adverse effects are critical or important is a clinical judgement and depends on the purpose of the study. For example if the study is of healthy individuals and the main focus is prevention, even minor harms might be important in the balance of harms and benefits. Alternatively, if the main study outcome is survival, only major or life-threatening harms might be relevant.[42]

Some studies with poor reporting of adverse effects may be methodologically sound and contact with authors may retrieve additional information.[66] However, contact with authors may retrieve little information, even when studies are recently completed and targeting a very common condition.[67]

4.5.3 Generalisability

A combination of factors such as patient characteristics (e.g. age), type and severity of disease, co-morbidities and clinical setting may contribute to the occurrence of adverse effects. Likely effects of confounding factors should be considered, particularly when using data from case series and observational studies. Interpreting adverse effects data from RCTs can also be problematic; in clinical trials most participants have the disease of interest but are otherwise healthy, but once the intervention is licensed it is often used in individuals with co-morbidities who are taking several other drugs.[42] These confounding factors may affect the generalisability of the study and should be clearly described.

4.6 DATA SYNTHESIS

4.6.1 Consideration of potential sources of heterogeneity

Systematic reviews of adverse effects often include evidence from a variety of sources including RCTs, observational studies, case reports and case series. There are difficulties in synthesising disparate data sets and differences between studies have to be considered as a source of heterogeneity (whether narrative or quantitative). In observational studies the extent of drug exposure is not as certain as in RCTs. For example, in cohort studies many patients might have received an incomplete course of medication which may lead to the underestimation of the true rate or severity of adverse effects. Patients in the control group may have procured the medication during a generally prolonged follow-up period and this may lead to overestimation of the rate of adverse effects in the control group.[73]

4.6.2 Methods of data synthesis

Whether narrative or quantitative synthesis is used, researchers should try to explore any patterns identified across the results and discuss the possible factors that might explain variations in study findings (e.g. rate and severity of adverse effects). Attempts should be made to explore possible relationships between characteristics of included studies and their reported findings and also between the findings of different studies. Researchers should clearly indicate the populations addressed by the included studies and carefully assess the applicability to other populations.[11]

Exploring heterogeneity in study findings is especially important.[74] Variations may be due to methodological differences and/or differences in the characteristics of the included studies. The possible effects of individual study quality indicators (e.g. follow-up period, methods used to identify adverse effects), study design, study size and funding sources in the analysis should be investigated and discussed.[11] Subgroup, sensitivity or regression analyses may be helpful for explaining some of these variations and generating functional hypotheses.

Researchers should provide a detailed description of cases of unusual or not previously recorded adverse effects.[48]

4.6.2.1 Meta-analysis techniques

There is little guidance about when and how to perform meta-analysis of adverse effects data. It is important, but not always easy to determine when and what data from multiple studies should be combined.[75] No standard technique is available for meta-analysis of diverse and heterogeneous data, and selection of techniques depends on different factors including the aim of the review, characteristics of selected studies and type of outcomes.[73] Although data from both observational studies and RCTs has been combined, for example to present a single estimate of mortality associated with chronic usage of non-steroidal anti-inflammatory drugs (NSAIDs),[73] in some reviews it may only be appropriate to quantitatively combine results from one or some study designs

(e.g. RCTs and cohort studies) and synthesise data from other types of studies (e.g. case series and case reports) using a narrative approach. As with efficacy data it may be appropriate to conduct subgroup analyses or, where data allow, use meta-regression to further explore the risk of adverse effects. For example the risk of bowel perforation with the cancer drug bevacizumab is thought to be higher in patients with ovarian cancer than in other cancers.[76]

Various Bayesian approaches to meta-analysis have been used[77-80] and when and how to use Bayesian approaches in reviews of adverse effects is a developing field. For example, a Bayesian approach has been used to combine evidence from case-control and prospective studies to estimate the absolute risk of developing ovarian cancer.[81]

4.7 REPORTING

The guidance for report writing presented in *Chapter 1, Section 1.3.6 Report writing*, is relevant to reviews of adverse effects. However, when reporting reviews of adverse effects it is important that detailed cross referencing to related reviews of the intended effects of the intervention are provided for the reader.

Summary: Systematic reviews of adverse effects

- The number of different reported adverse effects/events can be large and comprehensiveness needs to be balanced against clinical relevance. The outcomes selected should be those that are important in guiding decisions related to the intervention.
- Observational studies, such as controlled or uncontrolled cohort studies or case-control studies or large case series, are likely to provide more patients, longer follow-up and more generalisable data than RCTs.
- Search methods may vary depending on whether adverse effects are the main focus of the review or a secondary focus.
- Both the quality of the primary studies and the quality of the reporting of adverse events within the primary studies should be considered.
- Meta-analysis may be more challenging and problematic than when applied to effectiveness data. Whatever the method of synthesis, researchers should try to illustrate the patterns identified across the results and discuss the possible factors that might explain variations in study findings.

REFERENCES

1. Loke YK. Assessing the benefit-harm balance at the bedside. *BMJ* 2004;329:7-8.

2. Asscher AW, Parr GD, Whitmarsh VB. Towards the safer use of medicines. *BMJ* 1995;311:1003-6.

3. Cuervo GL, Clarke M. Balancing benefits and harms in health care. *BMJ* 2003;327:65-6.

4. Greenhalgh T, Kostopoulou O, Harries C. Making decisions about benefits and harms of medicines. *BMJ* 2004;329:47-50.

5. Ernst E, Pittler MH. Assessment of therapeutic safety in systematic reviews: literature review. *BMJ* 2001;323:546.

6. Aronson JK, Derry S, Loke YK. Adverse drug reactions: keeping up to date. *Fundam Clin Pharmacol* 2002;16:49-56.

7. Cosmi B, Castelvetri C, Milandri M, Rubboli A, Confoti A. The evaluation of rare adverse drug events in Cochrane reviews: the incidence of thrombotic thrombocytopenic purpura after ticlopidine plus aspirin for coronary stenting. [Abstract] In: *8th Cochrane Colloquium*; 2000 Oct 25-29; Cape Town, South Africa.

8. Loke YK, Price D, Herxheimer A. Chapter 14: Adverse effects. In: Higgins JPT, Green S, editors. *Cochrane Handbook for Systematic Reviews of Interventions Version 5.0.0 (updated February 2008)*: The Cochrane Collaboration; 2008. Available from: www.cochrane-handbook.org

9. Loke YK, Price D, Herxheimer A, Cochrane Adverse Effects Methods Group. Systematic reviews of adverse effects: framework for a structured approach. *BMC Med Res Methodol* 2007;7:32.

10. McIntosh H, Woolacott N, Bagnall A. Comparison of methods used to assess adverse events in three CRD reviews [abstract]. In: *11th Cochrane Colloquium*; 2003 Oct 26-31; Barcelona, Spain.

11. Chou R, Helfand M. Challenges in systematic reviews that assess treatment harms. *Ann Intern Med* 2005;142:1090-9.

12. Golder S, Loke Y, McIntosh HM. Room for improvement? A survey of the methods used in systematic reviews of adverse effects. *BMC Med Res Methodol* 2006;6:3.

13. McGettigan P, Henry D. Cardiovascular risk and inhibition of cyclooxygenase: a systematic review of the observational studies of selective and nonselective inhibitors of cyclooxygenase 2. *JAMA* 2006;296:1633-44.

14. Rodgers M, McKenna C, Palmer S, Chambers D, Van Hout S, Golder S, et al. Curative catheter ablation in atrial fibrillation and typical atrial flutter: systematic review and economic evaluation [in press]. *Health Technol Assess* 2008.

15. McIntosh HM, Woolacott NF, Bagnall AM. Assessing harmful effects in systematic reviews. *BMC Med Res Methodol* 2004;4:19.

16. Higgins JPT, Green S, (editors). *Cochrane Handbook for Systematic Reviews of Interventions. Version 5.0.0 [updated February 2008]*: The Cochrane Collaboration; 2008. Available from: www.cochrane-handbook.org

17. Aronson JK, Ferner RE. Clarification of terminology in drug safety. *Drug Saf* 2005;28:851-70.

18. Bagnall A-M, Jones L, Ginnelly L, Lewis R, Glanville J, Gilbody S, et al. A systematic review of atypical antipsychotic drugs in schizophrenia. *Health Technol Assess* 2003;7:1-193.

19. Dale KM, Coleman CI, Henyan NN, Kluger J, White CM. Statins and cancer risk: a meta-analysis. *JAMA* 2006;295:74-80.

20. Loke YK, Derry S. Reporting of adverse drug reactions in randomised controlled trials - a systematic survey. *BMC Clin Pharmacol* 2001;1:3.

21. Kearney PM, Baigent C, Godwin J, Halls H, Emberson JR, Patrono C. Do selective cyclo-oxygenase-2 inhibitors and traditional non-steroidal anti-inflammatory drugs increase the risk of atherothrombosis? Meta-analysis of randomised trials. *BMJ* 2006;332:1302-8.

22. Loke YK, Derry S, Aronson JK. A comparison of three different sources of data in assessing the frequencies of adverse reactions to amiodarone. *Br J Clin Pharmacol* 2004;57:616-21.

23. Arnaiz JA, Carne X, Riba N, Codina C, Ribas J, Trilla A. The use of evidence in pharmacovigilance. Case reports as the reference source for drug withdrawals. *Eur J Clin Pharmacol* 2001;57:89-91.

24. Loke YK, Price D, Derry S, Aronson JK. Case reports of suspected adverse drug reactions: systematic literature survey of follow-up. *BMJ* 2006;332:335-9.

25. Aronson JK. Anecdotes as evidence: we need guidelines for reporting anecdotes of suspected adverse drug reactions. *BMJ* 2003;326:1346.

26. Kelly WN. The quality of published adverse drug event reports. *Ann Pharmacother* 2003;37:1774-8.

27. Medawar C, Herxheimer A, Bell A, Jofre S. Paroxetine, Panorama and user reporting of ADRs: consumer intelligence matters in clinical practice and post-marketing drug surveillance. *Int J Risk Saf Med* 2002;15:161-9.

28. Derry S, Loke YK, Aronson JK. Incomplete evidence: the inadequacy of databases in tracing published adverse drug reactions in clinical trials. *BMC Med Res Methodol* 2001;1:7.

29. Wieland S, Dickersin K. Selective exposure reporting and Medline indexing limited the search sensitivity for observational studies of the adverse effects of oral contraceptives. *J Clin Epidemiol* 2005;58:560-7.

30. Golder S, McIntosh HM, Duffy S, Glanville J. Developing efficient search strategies to identify reports of adverse effects in MEDLINE and EMBASE. *Health Info Libr J* 2006;23:3-12.

31. Biarez O, Sarrut B, Doreau CG, Etienne J. Comparison and evaluation of nine bibliographic databases concerning adverse drug reactions. *DICP* 1991;25:1062-5.

32. Thomson Scientific. *Derwent Drug File: Definitive drug journal and conference information*. Thomson Scientific; 2004. [cited 2008 20 Oct]. Available from: http://scientific.thomson.com/media/dw/productpdfs/ddf-compare.pdf

33. Bagnall AM, Jones L, Glanville J, Kleijnen J. Assessing adverse events in a systematic review of atypical antipsychotics for schizophrenia. In: *4th Symposium on Systematic Reviews: Pushing the Boundaries*; 2002 Jul; Oxford. 2002.

34. Sodha RV, Van Amelsvoort T. Multi-database searches in biomedicine: citation duplication and novelty assessment using carbamazepine as an example. *J Inf Sci* 1994;20:139-41.

35. Van Putte N. A comparison of four biomedical databases for the retrieval of drug literature. *Health Inf Lib* 1991;3:119-27.

36. Madden M, MacDonald A. An evaluation and comparison of nine drug information retrieval services. *Drug Inf J* 1977;11:47-59.

37. Windsor DA. Adverse-reactions literature: a bibliometric analysis. *Methods Inf Med* 1977;16:52-4.

38. Nuovo J, Sather C. Reporting adverse events in randomized controlled trials. *Pharmacoepidemiol Drug Saf* 2007;16:349-51.

39. Bernal-Delgado E, Fisher ES. Abstracts in high profile journals often fail to report harm. *BMC Med Res Methodol* 2008;8:14.

40. MacLean CH, Morton SC, Ofman JJ, Roth EA, Shekelle PG, Southern California Evidence-Based Practice Center. How useful are unpublished data from the Food and Drug Administration in meta-analysis? *J Clin Epidemiol* 2003;56:44-51.

41. Edwards JE, McQuay HJ, Moore RA, Collins SL. Reporting of adverse effects in clinical trials should be improved: lessons from acute postoperative pain. *J Pain Symptom Manage* 1999;18:427-37.

42. Ioannidis JP, Evans SJ, Gøtzsche PC, O'Neill RT, Altman DG, Schulz K, et al. Better reporting of harms in randomized trials: an extension of the CONSORT statement. *Ann Intern Med* 2004;141:781-8.

43. Aitken L, Gallagher R, Madronio C. Principles of recruitment and retention in clinical trials. *Int J Nurs Pract* 2003;9:338-46.

44. Lazarou J, Pomeranz BH, Corey PN. Incidence of adverse drug reactions in hospitalized patients: a meta-analysis of prospective studies. *JAMA* 1998;279:1200-5.

45. Ross SD. Drug-related adverse events: a readers' guide to assessing literature reviews and meta-analyses. *Arch Intern Med* 2001;161:1041-6.

46. Henry D, Moxey A, O'Connell D. Agreement between randomized and non-randomized studies: the effects of bias and confounding [abstract]. In: *9th Cochrane Colloquium*; 2001 Oct 9-13; Lyon, France.

47. Olsen H, Klemetsrud T, Stokke HP, Tretli S, Westheim A. Adverse drug reactions in current antihypertensive therapy: a general practice survey of 2586 patients in Norway. *Blood Press* 1999;8:94-101.

48. Ioannidis JPA, Lau J. Improving safety reporting from randomised trials. *Drug Saf* 2002;25:77-84.

49. Baba-Akbari A. *Study of the scale, nature and causes of adverse events and methods to identify them* [PhD]. York: University of York; 2006.

50. Bonhoeffer J, Kohl K, Chen R, Duclos P, Heijbel H, Heininger U, et al. The Brighton Collaboration: addressing the need for standardized case definitions of adverse events following immunization (AEFI). *Vaccine* 2002;21:298-302.

51. Trotti A, Bentzen SM. The need for adverse effects reporting standards in oncology clinical trials. *J Clin Oncol* 2004;22:19-22.

52. Kohl KS, Bonhoeffer J, Chen R, Duclos P, Heijbel H, Heininger U, et al. The Brighton Collaboration: enhancing comparability of vaccine safety data. *Pharmacoepidemiol Drug Saf* 2003;12:335-40.

53. National Institute of Allergy and Infectious Diseases. *Division of AIDS table for grading the severity of adult and pediatric adverse events [monograph online]*. Bethesda, MD: National Institute of Allergy and Infectious Diseases; 2004. Available from: www3.niaid.nih.gov/research/resources/DAIDSClinRsrch/

54. National Cancer Institute. *Common Toxicity Criteria v2.0 (CTC) [monograph online]*. Bethesda, MD: National Cancer Institute; 1999. Available from: http://ctep.cancer.gov/reporting/ctc_archive.html

55. Peloso PM, Wright JG, Bombardier C. A critical appraisal of toxicity indexes in rheumatology. *J Rheumatol* 1995;22:989-94.

56. Vitiello B, Riddle MA, Greenhill LL, March JS, Levine J, Schachar RJ, et al. How can we improve the assessment of safety in child and adolescent psychopharmacology? *J Am Acad Child Adolesc Psychiatry* 2003;42:634-41.

57. Miller AB, Hoogstraten B, Staquet M, Winkler A. Reporting results of cancer treatment. *Cancer* 1981;47:207-14.

58. Corso DM, Pucino F, DeLeo JM, Calis KA, Gallelli JF. Development of a questionnaire for detecting potential adverse drug reactions. *Ann Pharmacother* 1992;26:890-6.

59. Woloshynowych M, Rogers S, Taylor-Adams S, Vincent C. The investigation and analysis of critical incidents and adverse events in healthcare. *Health Technol Assess* 2005;9:1-158.

60. Ioannidis JP, Lau J. Completeness of safety reporting in randomized trials: an evaluation of 7 medical areas. *JAMA* 2001;285:437-43.

61. MacLehose HG, Klaes D, Garner P. What methods do trials use to collect adverse data? [abstract]. In: *11th Cochrane Colloquium*; 2003 Oct 26-31; Barcelona, Spain.

62. Price D, Jefferson T. Methodological problems in the interpretation of adverse event data included in a systematic review of adverse events following measles-mumps-rubella (MMR) immuniziation [abstract]. In: *4th Symposium on Systematic Reviews: Pushing the Boundaries*; 2002 Jul; Oxford.

63. Chan AW, Hrobjartsson A, Haahr MT, Gøtzsche PC, Altman DG. Empirical evidence for selective reporting of outcomes in randomized trials: comparison of protocols to published articles. *JAMA* 2004;291:2457-65.

64. Ioannidis JP, Contopoulos-Ioannidis DG. Reporting of safety data from randomised trials. *Lancet* 1998;352:1752-3.

65. Keech AC, Wonders SW, Cook DI, Gebski VJ. Balancing the outcomes: reporting adverse events. *Med J Aust* 2004;181:215-8.

66. Price D, Jefferson T, Demicheli V. Methodological issues arising from systematic reviews of the evidence of safety of vaccines. *Vaccine* 2004;22:2080-4.

67. Ioannidis JP, Chew P, Lau J. Standardized retrieval of side effects data for meta-analysis of safety outcomes. A feasibility study in acute sinusitis. *J Clin Epidemiol* 2002;55:619-26.

68. Meenan RT, Saha S, Chou R, Swarztrauber K, Pyle Krages K, O'Keefe-Rosetti M, et al. *Effectiveness and cost-effectiveness of echocardiography and carotid imaging in the management of stroke*. Rockville, MD: Agency for Healthcare Research and Quality (AHRQ); 2002.

69. Downs SH, Black N. The feasibility of creating a checklist for the assessment of the methodological quality both of randomised and non-randomised studies of health care interventions. *J Epidemiol Community Health* 1998;52:377-84.

70. Slim K, Nini E, Forestier D, Kwiatkowski F, Panis Y, Chipponi J. Methodological index for non-randomized studies (minors): development and validation of a new instrument. *ANZ J Surg* 2003;73:712-6.

71. Wells GA, Shea B, O'Connell D, Peterson J, Welch V, Tugwell P. The Newcastle-Ottawa Scale (NOS) for assessing the quality of nonrandomised studies in meta-analyses [abstract]. In: *3rd Symposium on Systematic Reviews: Beyond the Basics*; 2000 Jul; Oxford.

72. Pope C, Mays N, Popay J. *Synthesizing qualitative and quantitative health evidence: a guide to methods*. Maidenhead: Open University Press; 2007.

73. Tramer MR, Moore RA, Reynolds DJM, McQuay HJ. Quantitative estimation of rare adverse events which follow a biological progression: a new model applied to chronic NSAID use. *Pain* 2000;85:169-82.

74. Egger M, Schneider M, Davey Smith G. Spurious precision? Meta-analysis of observational studies. *BMJ* 1998;316:140-4.

75. Naylor CD. The case for failed meta-analyses. *J Eval Clin Pract* 1995;1:127-30.

76. Han ES, Monk BJ. What is the risk of bowel perforation associated with bevacizumab therapy in ovarian cancer? *Gynecol Oncol* 2007;105:3-6.

77. Wang J, Donnan PT, MacDonald TM. An approximate Bayesian risk analysis of the gastro-intestinal safety of ibuprufen. *Pharmacoepidemiol Drug Saf* 2002;11:695-701.

78. Babapulle MN, Joseph L, Belisle P, Brophy JM, Eisenberg MJ. A hierarchical Bayesian meta-analysis of randomised clinical trials of drug-eluting stents. *Lancet* 2004;364:583-91.

79. Stricker BH, Psaty BM. Detection, verification, and quantification of adverse drug reactions. *BMJ* 2004;329:44-7.

80. Zucker DR, Schmid CH, McIntosh MW, D'Agostino RB, Selker HP, Lau J. Combining single patient (N-of-1) trials to estimate population treatment effects and to evaluate individual patient responses to treatment. *J Clin Epidemiol* 1997;50:401-10.

81. Muller P, Parmigiani G, Schildkraut J, Tardella L. A Bayesian hierarchical approach for combining case-control and prospective studies. *Biometrics* 1999;55:858-66.

CHAPTER 5

SYSTEMATIC REVIEWS OF ECONOMIC EVALUATIONS

5.1 INTRODUCTION **201**
5.1.1 What is an economic evaluation? **202**
5.2 CONDUCTING A REVIEW OF ECONOMIC EVALUATIONS **203**
5.2.1 The review question **203**
5.2.2 Inclusion criteria **203**
5.3 IDENTIFYING ECONOMIC EVALUATIONS **204**
5.4 DATA EXTRACTION **204**
5.5 QUALITY ASSESSMENT **206**
5.5.1 Methods of deriving effectiveness data **206**
5.5.2 Cost analysis **207**
5.5.2.1 Cost categories 207
5.5.3 Measurement and valuation of health benefits (utilities) **208**
5.5.4 Methods of synthesising costs and effects **208**
5.5.5 Analysis of uncertainty **208**
5.5.5.1 Statistical comparisons 209
5.5.5.2 Bootstrapping 209
5.5.5.3 Sensitivity analysis - parameter uncertainty 209
5.5.5.4 Sensitivity analysis - methodological uncertainty 209
5.5.5.5 Probabilistic sensitivity analyses 211
5.5.6 Generalisability of the results **212**
5.5.7 Use of checklists to assess the quality of economic evaluations **212**
5.5.8 Quality scoring systems **212**
5.6 DATA SYNTHESIS **212**
5.7 REPORTING **214**
REFERENCES **215**

5.1 INTRODUCTION

Where resources are limited, decision-makers need to consider not only whether an intervention is effective but whether it is also cost-effective. If a new intervention requires more resource than current practice, then this will have to be found from elsewhere within the health system, and adoption may displace other treatments or services. Considering economic aspects alongside clinical effectiveness can make reviews more useful to health care decision-makers.[1] There are three main options for addressing economic issues within or alongside a systematic review of effectiveness.

First, at a basic level any cost or resource information reported in the effectiveness studies can be extracted and presented as an additional outcome (see *Chapter 1 Section 1.2.2.2 Review question and inclusion criteria*). Although this may not constitute a formal economic evaluation, it can provide useful additional information that may be of value in a decision-making context.

The second option involves undertaking two mutually dependent components: a review of all available evidence and an economic evaluation, which is often achieved through the use of decision modelling.[2] The findings of the review are used to help develop and populate a decision model. This is a common approach, used primarily in health technology assessment. Many such examples have been undertaken for NICE.[3-5] However, decision modelling is outside the remit of this chapter as it involves specialist skills, but good introductory texts are available.[6]

The third option is to carry out a systematic review of existing economic evaluation studies that have focused on the intervention in question. This is often done alongside the clinical effectiveness review. However, the exact role for this type of review is unclear and questions remain as to whether it is actually useful to undertake reviews of existing economic evaluations.[7] A fundamental reason for undertaking a review of any kind is that the collation and synthesis of evidence will be more useful than that available from any individual study. But, given the disparity in methods used across existing economic evaluations it is extremely difficult to synthesise such studies into a coherent whole. Studies need to be adjusted to achieve standardised results, but in reality this is rarely achievable given the diverse nature of the elements considered, including differences in perspectives, health care systems (which use different resources) and time horizons.

Although some health economists have expressed concerns about the value of systematic reviews of economic evaluation[7, 8] methods are available to guide their conduct[9] and a large number have been undertaken.[10, 11] Reasons for undertaking this type of review include i) to inform development of a decision model; ii) to identify the most relevant economic evaluation to inform a particular question; and iii) to identify the key economic trade-offs implicit in a particular treatment choice.[8] Whether or not these are the only viable reasons is still open to debate, but when considering undertaking such a review it is important to have a clear rationale for doing so and that the objectives set are attainable.

This chapter assumes that, for whatever reason, a systematic review of economic evaluation is appropriate. The aim of the chapter is to guide non-health economists through some of the main issues to be considered when undertaking a systematic review of economic evaluation, and should be read in conjunction with the general

guidance presented in *Chapter 1*. The same basic principles apply and only issues specific to reviews of economic evaluation are described here.

5.1.1 What is an economic evaluation?

An economic evaluation is essentially a tool to allow comparative health interventions to be evaluated in order to address the issue of efficient resource allocation. It is widely accepted that on their own, economic evaluations are not sufficient to inform decision-making, but that they are a necessary component of the decision-making process.

An economic evaluation is a study in which both the cost and health outcomes of comparative technologies or interventions have been assessed. The aim is to identify, measure, value and compare the cost and consequences of the alternative interventions being considered. Primary economic evaluation can either be undertaken as an integral part of a single clinical study, often a trial, or can be based on more than one source of effectiveness data derived from expert opinion, authors' assumptions or reviews of clinical effectiveness - in this way economic evaluations can both use and be used in systematic review. Either approach may employ modelling techniques.

Full economic evaluation provides a framework for structuring specific decision problems and considers both the effectiveness and cost data for two or more interventions being compared within the analysis. A partial economic evaluation makes no comparison and simply describes a single intervention or service through consideration of costs or consequences alone.[12] There are three generic types of full economic evaluation, each defined on the basis of the outcomes measured. See *Box 5.1.*

Box 5.1 Generic types of full economic evaluation

Cost-effectiveness analysis (CEA)
A single clinical outcome expressed in natural units (e.g. postoperative infections prevented or life years gained) is used. If multiple clinical outcomes are used, cost-consequences analysis (CCA) may be reported which includes all clinical outcomes and costs for each alternative. Clinical outcomes may vary in direction and magnitude of effect.

Cost-utility analysis (CUA)
Clinical outcomes (health states) are converted into utility scores using a utility measurement instrument such as the SF-6D or the EuroQol (EQ-5D) to estimate quality-adjusted-life-years (QALYs). Alternatively other utility measures such as healthy-years-equivalent (HYE) may be used.

Cost-benefit analysis (CBA)
Clinical outcomes are converted into monetary units so that a net benefit (or cost) can be estimated. Methods used to convert health benefits to monetary values include willingness to pay (WTP) and the human capital approach (HCA).

As with other types of evaluation, a wide range of methods are used in economic evaluation, and therefore the methodological rigour of studies can vary; methods can be used inappropriately and decisions about the appropriateness of methods can impact on the quality and validity of a review. It is therefore important that the researcher understands at least some basic methodological concepts prior to undertaking a review of economic evaluations. More complex issues can be dealt with in consultation with the health economist that should be included in the review team.

5.2 CONDUCTING A REVIEW OF ECONOMIC EVALUATIONS

A protocol for a review of economic evaluations should be developed as for a review of effectiveness (see *Chapter 1, Section 1.2 The review protocol*) and be used to establish in advance the methods that will be used throughout. Early discussion with a health economist is recommended.

5.2.1 The review question

An economic review may consider questions relating to the cost-effectiveness of differing interventions, outcomes, populations and settings, or may explore the effect of other factors, such as change in patient adherence, on the cost-effectiveness of interventions.

The scope may need to be broader than that set for clinical effectiveness to capture all relevant costs and consequences. For example, resources such as further treatments consumed or avoided downstream of the intervention will need to be included in the economic evaluation. Working through the care pathway for relevant interventions may be helpful in identifying relevant costs and benefits, and in formulating the review question.

5.2.2 Inclusion criteria

As described in *Chapter 1, Section 1.2 The review protocol*, PICOS may be helpful in defining clear inclusion and exclusion criteria.

A basic consideration will be whether to include only full economic evaluations as under certain circumstances and for specific review questions, it might be appropriate to include partial economic evaluations. For example, if the review question involves incremental costs, then cost analyses may be relevant. Alternatively, if the question is about relative benefits (measured using methods such as QALYs or WTP) studies comparing these outcomes might be considered appropriate even if they are not full economic evaluations. Where partial evaluations are included, as for all reviews, a systematic and transparent approach must be taken.

Reviews of economic evaluations are more likely to require a time horizon of 'till death' which may necessitate including types of study that differ from the clinical review, and require additional inclusion criteria being specified.

The review may be limited to include only those economic studies that incorporated a high quality source of clinical evidence, from an RCT or a systematic review.

Other considerations around the study type may include, for example, whether to restrict inclusion to economic evaluations from a societal perspective.

Outcomes may also differ from those of primary interest in the clinical review. For example, quality-adjusted-life-years are more likely to be relevant to economic evaluation.

5.3 IDENTIFYING ECONOMIC EVALUATIONS

Literature searches can utilise both the sources used routinely for effectiveness reviews (see *Chapter 1, Section 1.3.1 Identifying research evidence for systematic reviews*) and resources that focus on economic studies. These include specialised databases such as CRD's NHS Economic Evaluation Database (NHS EED)[13] which contains critical abstracts of full economic evaluations, alongside bibliographic details of partial evaluations, and the Health Economic Evaluations Database (HEED),[14] produced by John Wiley and Sons, which contains summaries of economic evaluations (full and partial). As both NHS EED and HEED solely contain records of economic evaluation the strategy used to search these databases need only contain terms related to the subject area. In theory these databases should capture all health economic evaluations. However, if it is essential that results are as current as possible, additional searches of the most recent updates of MEDLINE and EMBASE could be carried out. If this is done the search strategy should contain terms for both the subject topic area as well as relevant economics terms. Similarly, if other subject specific databases are used, the search strategy should combine economic terms with subject terms.

Search filters are available for a range of study types including economic evaluations. The Hedges Project, based at McMaster University and funded by the US National Library of Medicine, develops search strategies ('hedges') to improve retrieval of study reports from large biomedical bibliographic databases such as MEDLINE, EMBASE, and CINAHL (http://hiru.mcmaster.ca/hiru/HIRU_Hedges_home.aspx). A range of filters, including economic and cost filters, is available, together with research papers providing information about how they were developed. The InterTASC Information Specialists' Subgroup Search filter resource provides a comprehensive source of filters, including a section on economics (www.york.ac.uk/inst/crd/intertasc/econ.htm). The search strategies used for the NHS EED database can be found at www.crd.york.ac.uk/crdweb/html/help.htm

5.4 DATA EXTRACTION

Data extraction should capture the key methodological elements that can impact on the results of an economic evaluation. These include: the perspective, the population, time horizon, outcomes, discounting, and the techniques employed in conducting the economic analysis. Consideration of the care pathway can be helpful in structuring data extraction.

Data extraction requirements are specific to each review question but will need to include the following areas:

- Study question, included population, intervention, comparator and setting
- Modelling techniques
- Sources and quality of clinical data
- Sources and quality of cost data
- Study outcomes in terms of health benefits and costs, and the methods used to synthesise them
- Methods for dealing with uncertainty
- Study results, including results of analyses of uncertainty

Additional study-specific issues may also need to be included. For example, whether adverse effects were considered in the cost analysis or if certain health states were included in a Markov model.

If, following the systematic review of economic evaluations, a new primary economic analysis is planned, then additional data may need to be extracted to inform the design or conduct of that analysis.

An example data extraction form is presented in Box 5.2.

Box 5.2: Example data extraction template

Type of economic evaluation	
Study Objective	
Interventions	
Location/Setting	
Methods	Analytical approach:<br>Effectiveness data:<br>Monetary benefit and utility valuations:<br>Measure of benefit:<br>Cost data:<br>Analysis of uncertainty:
Results	
Authors' conclusions	

5.5 QUALITY ASSESSMENT

Quality assessment is likely to focus on the following elements of the economic evaluation, each of which can have an important impact on the validity of the overall results of that study:

- Methods of deriving the effectiveness data
- Measurement of resource data
- Valuation of resource data
- Measurement and valuation of health benefits (utilities)
- Method of synthesising the costs and effects
- Analysis of uncertainty
- Generalisability of the results

This is not an exhaustive list, but an understanding of these issues, which are discussed in more detail below, will provide insight into the quality assessment of economic evaluations. Quality assessment of decision models is not covered in detail here due to the technical nature of the material. It is recommended that more detailed information on good practice in decision modelling be consulted.[15]

5.5.1 Methods of deriving effectiveness data

There is a hierarchy of sources of evidence ranging from a formal systematic review to expert opinion and authors' assumptions.[16] Where possible economic evaluation should use effectiveness data obtained from a systematic review. However, non-systematic synthesis of effectiveness data may be justifiable when it is the only available source of evidence.

The type of effectiveness data included in an economic evaluation can vary from a single efficacy parameter obtained from a meta-analysis of RCTs to epidemiological data mapping the natural history of disease. Quality assessment of the clinical effectiveness data incorporated in an economic evaluation will depend on the type of clinical data used; whether the data were obtained from a single study or from the literature or from expert opinion; and whether modelling techniques were used.

When the effectiveness data has been derived from a single study, quality assessment should be undertaken as described in *Chapter 1*. However, additional elements will also need to be assessed. For example, whether the study time horizon is adequate to capture all the relevant health outcomes required and, if statistical modelling techniques have been used to extrapolate the data, whether the extrapolation methods and assumptions used were appropriate.[17]

When the effectiveness data has been synthesised from a variety of sources assessment should focus on the quality of the literature review and the methods used to synthesise the data including:

- Whether a search strategy was used
- Which databases were searched

- Whether there were clear inclusion and exclusion criteria
- Whether sufficient information was given about the quality of the included studies

5.5.2 Cost analysis

Quality assessment of cost analysis should consider which costs were evaluated in the study, the measurement of the associated resource quantities, and the valuation (cost) of those resources. Some of the issues that need to be assessed are common to all economic evaluations, while others are specific to the type of approach used.

5.5.2.1 Cost categories

For any economic evaluation all costs relevant to the study question and the perspective adopted or viewpoint from which the analysis has been undertaken should have been included. For example patient travel costs are a cost from the patient's perspective and a cost from society's perspective, but not a cost from the hospital's perspective.

Measurement of resources data

Resource use is measured in physical units such as equipment, staff, dressings and drugs. Issues to consider are as follows:

- The sources used to collect resource utilisation data should be reported clearly (e.g. clinical trials, administrative databases, clinical databases, medical records and published literature)
- Resource quantities should be reported independently from the costs, so that assessment of the measurement method is facilitated
- Any assumptions in the measurement of resources should be explicitly reported and justified
- If an expert was consulted to estimate some of the resources, the methods used should be described

For trial-based economic evaluations, the most valid resource estimates are considered to be those collected prospectively alongside effectiveness data, utilising the robust infrastructure established for the trial.[18]

If resources utilized were identified through a review of the literature, details of the process employed to identify and select the patterns of resource utilisation and the quantities used should have been given.

Valuation of resource data

For the valuation of resources, the relevant issues to consider are as follows:

- All the sources used to obtain unit costs should be reported and be relevant for the specific study setting
- All costs should be adjusted to a specific price year so that the effects of inflation are removed from the cost estimation

- If the time horizon for estimating costs was longer than one year, discounting should have been performed in order to reflect time preferences[19]
- If prices were used instead of costs and cost-to-charge ratios calculated these should reflect the true opportunity costs of the strategies compared[20]

5.5.3 Measurement and valuation of health benefits (utilities)

Utilities may be measured using either a generic valuation tool, such as the SF-6D or the EQ-5D, or a disease specific tool which may have been obtained using either standard gamble or time trade off techniques. Tools differ considerably (a full discussion is given in the books by Drummond[12] and Brazier[21]) and choice of tool can impact on the results obtained and on their usefulness in priority setting. As a minimum assessment should consider who provided the scores (patients, clinicians, general public, etc.), which tool was used (EQ-5D, SF-6D, etc.) and when the scores were elicited (at baseline, during treatment, after treatment, etc.). A useful overview and comparison of the impact of different measures in rheumatoid arthritis is available.[22]

5.5.4 Methods of synthesising costs and effects

The true economic value of an intervention compared to another depends on the additional costs and benefits. Incremental cost-effectiveness ratios are the ratios that capture this relative value. Unless a treatment is clearly dominant (both cheaper and more effective), incremental cost-effectiveness ratios (ICERs) should have been calculated as this is the only appropriate way of capturing the true economic value.[12] A paper should report sufficient data to ascertain dominance from the figures given, rather than relying on a statement from the authors which can be made in error and be potentially misleading. Cost-effectiveness results should have been reported in both a disaggregated and an aggregated way. That is, undiscounted and discounted health benefit and cost results should have been reported both separately and as part of the ICERs. It is also appropriate to report the net benefit statistic, which is sometimes used to overcome the statistical issues raised when dealing with a ratio, like the ICER.

5.5.5 Analysis of uncertainty

A well-conducted economic evaluation should investigate as thoroughly as possible, the following sources of uncertainty:

- Parameter uncertainty, which occurs because parameters are estimated from samples and their true value is unknown
- Methodological uncertainty, which arises from the analytical methods used in the evaluation, particularly where there is disagreement around the methods used (e.g. the inclusion of indirect costs, discounting of health benefits, discount rate)
- Modelling uncertainty which can arise due to the simplifying assumptions that are often required to facilitate modelling

Methods of evaluating uncertainty include statistical comparisons, bootstrapping, sensitivity analyses (one-way or multi-way sensitivity analyses, threshold analyses and analyses of extremes or worst/best case analysis) and probabilistic sensitivity analyses. The method(s) employed will vary depending on what is being assessed and the types of data that were used as input parameters in the economic evaluation.

5.5.5.1 Statistical comparisons

Statistical tests comparing effects, costs or cost-effectiveness are appropriate for studies that have derived their effectiveness and costs from patient level data. The quality assessment of the statistical comparisons performed should focus on the appropriateness of the type of tests used and the results reported (e.g. 95% confidence intervals; p-values).

5.5.5.2 Bootstrapping

Bootstrapping is a statistical method that can be applied to capture uncertainty where patient level data are used.[23] Due to the fact that the ICER is a ratio, normal parametric statistical methods based on the standard error cannot be used. Non-parametric bootstrapping is an alternative method which allows a comparison of the arithmetic means without making any assumptions about the sampling distribution. However, it should be noted that economic evaluations can use a net benefit statistic rather than an ICER to overcome the statistical problems associated with a ratio.[24]

5.5.5.3 Sensitivity analysis - parameter uncertainty

Sensitivity analyses of parameter uncertainty are usual in economic evaluations that obtain their data from systematic or other reviews. The aim of the sensitivity analyses is to evaluate the sensitivity of the results to changes in the parameter estimates. N-way sensitivity analyses and threshold analysis can only vary a few parameters at the same time in practice. In contrast, probabilistic sensitivity analysis (PSA) (see below) can vary all parameters at the same time, subject to data availability.

The following issues should be assessed:

- Whether the parameters chosen were justified
- Whether variations were performed across meaningful ranges of values
- Whether the robustness of the results was assessed according to a previously agreed level of 'acceptable variation'

5.5.5.4 Sensitivity analysis - methodological uncertainty

Uncertainty around analytical methods is also assessed through the use of sensitivity analysis. For example, the impact of different discount rates and the use of discounting (or not) on health benefits should have been assessed in studies with a long time horizon.

Box 5.3: Checklist for assessing economic evaluations

Study design

1. Was the research question stated?
2. Was the economic importance of the research question stated?
3. Was/were the viewpoint(s) of the analysis clearly stated and justified?
4. Was a rationale reported for the choice of the alternative programmes or interventions compared?
5. Were the alternatives being compared clearly described?
6. Was the form of economic evaluation stated?
7. Was the choice of form of economic evaluation justified in relation to the questions addressed?

Data collection

8. Was/were the source(s) of effectiveness estimates used stated?
9. Were details of the design and results of the effectiveness study given (if based on a single study)?
10. Were details of the methods of synthesis or meta-analysis of estimates given (if based on an overview of several effectiveness studies)?
11. Were the primary outcome measure(s) for the economic evaluation clearly stated?
12. Were the methods used to value health states and other benefits stated?
13. Were the details of the subjects from whom valuations were obtained given?
14. Were productivity changes (if included) reported separately?
15. Was the relevance of productivity changes to the study question discussed?
16. Were quantities of resources reported separately from their unit cost?
17. Were the methods for the estimation of quantities and unit costs described?
18. Were currency and price data recorded?
19. Were details of price adjustments for inflation or currency conversion given?
20. Were details of any model used given?
21. Was there a justification for the choice of model used and the key parameters on which it was based?

(Continued)

Analysis and interpretation of results

22. Was time horizon of cost and benefits stated?
23. Was the discount rate stated?
24. Was the choice of rate justified?
25. Was an explanation given if cost or benefits were not discounted?
26. Were the details of statistical test(s) and confidence intervals given for stochastic data?
27. Was the approach to sensitivity analysis described?
28. Was the choice of variables for sensitivity analysis justified?
29. Were the ranges over which the parameters were varied stated?
30. Were relevant alternatives compared? (i.e. Were appropriate comparisons made when conducting the incremental analysis?)
31. Was an incremental analysis reported?
32. Were major outcomes presented in a disaggregated as well as aggregated form?
33. Was the answer to the study question given?
34. Did conclusions follow from the data reported?
35. Were conclusions accompanied by the appropriate caveats?
36. Were generalisability issues addressed?

Based on Drummond's checklist[27]

5.5.5.5 Probabilistic sensitivity analyses

This method can only be used to deal with parameter uncertainty in modelling-based economic evaluations. PSA, also referred to as second-order uncertainty, considers the uncertainty surrounding the value of a parameter. This is achieved by assigning a probability distribution rather than a point estimate to each parameter. The quality assessment in this case should focus on whether:

- Appropriate distributions were assigned to the model parameters[6, 25]
- Relevant assumptions were tested. For example, assumptions about model structure or interpretation of the available evidence[12]

5.5.6 Generalisability of the results

Generalisability refers to the extent to which the results obtained can be applied to different settings. The relevance of the intervention, the patient population and the resources which have been included in the economic evaluation will determine whether the results can be generalised. Uncertainty regarding the generalisability of the results to the relevant study setting would usually be assessed through sensitivity analyses. A useful discussion on this issue is available.[26]

5.5.7 Use of checklists to assess the quality of economic evaluations

Several reliable, comprehensive, and easy to use checklists are available to guide the quality assessment of economic evaluations. The most widely used is the BMJ checklist.[27] Both a 10-item version and an expanded 35-item version are available. In addition, a 36th item relating to generalisability may be added if it is relevant to the review (see *Box 5.3*). Although, this checklist does not provide detailed coverage of some issues relevant to modelling studies, it can be augmented using specific items such as model type, structural assumptions, time horizon, cycle length and health states. Alternatively, a checklist developed to assess the quality of the models used in economic evaluations can be used as a complement to the BMJ checklist.[15]

In some cases the validity of an economic evaluation may be difficult to assess due to limitations in reporting, an issue common to many studies and covered in *Chapter 1*.

5.5.8 Quality scoring systems

Several quality scoring systems have been devised for use in assessing the methodological quality of economic evaluations. These are generally based on completing checklists, assigning values to the different items considered, and summing these values to obtain a final score, which is intended to reflect the quality of the appraised study.

Six published quality scoring systems for economic evaluations have been identified, but none of these are considered to be sufficiently valid and reliable for use as a method of quality assessment.[28] Given the limitations presented by quality scoring systems, their use is not recommended. Rather, it is preferable to present a checklist or a descriptive critical assessment based on appropriate guidelines or checklists, which should describe the methods and results, strengths and weaknesses and the implications of the strengths and weaknesses on the reliability of the conclusions.

5.6 DATA SYNTHESIS

Synthesis should begin with descriptive comparisons of the study question, methods and results. It may be useful to include summary tables which present key information relating to population, country, perspective, comparison of interventions, measure of benefit and incremental cost-effectiveness ratios. An example is given in *Table 5.1*. The range of incremental cost-effectiveness ratios should be presented and the reliability

(internal validity) and relevance (generalisability) of the estimates should be explored. The analytical approaches used in the studies should be compared and their robustness discussed, for example, whether the studies used the same type of modelling technique and the same model structure.

This can be developed further and the results of the original cost-effectiveness studies adjusted to conform to standard methods specified for a given setting, in order that study results can be compared more readily. In its simplest form, this may involve converting the currency of the cost estimates. However, if adequate detail is reported, local unit costs appropriate to the review could be substituted for the unit costs used in the individual studies.[29] This would depend on the amount of disaggregated data reported in the individual economic evaluations and the heterogeneity across studies. The more disaggregated and homogenous the data, the easier and more feasible it would be to adjust the results. Theoretically, a meta-analysis of economic evaluations could be done. However, this is not straightforward and would require input from an experienced health economist.[30, 31] In practice, economic evaluations addressing a particular question are often heterogeneous and published reports do not generally present sufficient detail to permit such adjustments.

Table 5.1: Example summary table

Author	Population	Country	Perspective	Interventions	Measure of benefit	Results
Bolin K.[32]	Smokers aged 35 or older	Sweden	Societal	Bupropion<br>Nicotine replacement therapy	QALY	SEK23,400/ QALY gained for men for bupropion<br>SEK16,600/ QALY gained for women for bupropion
Cornuz J.[33]	Smokers aged 45, 20 cigs per day	Canada	Third-party payer	Bupropion<br>Counselling<br>Replacement therapies	Life years	CA$792/life year gained for men for bupropion
Nielsen K.[34]	Smokers in cessation programme	USA	Employer	Sustained-release bupropion<br>Nicotine patch<br>Both	Quit rate	30.3% quit rate and $163.49/ person for bupropion<br>16.4% quit rate and $245.22/ person for nicotine patch<br>35.5% quit rate and $408.71/ person for both

SEK = Swedish Kroner CA$ = Canadian Dollars $ = US dollars

5.7 REPORTING

Readers of the systematic review may well include non-economists so the results of the cost-effectiveness analyses need to be presented as clearly as possible. For example, it may be useful to report the absolute and incremental costs and effectiveness as well as the incremental cost-effectiveness ratios as these may be more readily understood. In *Table 5.1*, the absolute quit rates (where reported) are given alongside the incremental cost-effectiveness ratios.

Summary: Systematic reviews of economic evaluations

- The inclusion criteria for a review of effectiveness and a review of economic evaluations are likely to overlap, however, additional specific criteria may be needed ranging from the source of cost and clinical evidence to the use of a modelling approach.
- Preference based values of health outcomes as well as disease-specific measures should be considered when forming the inclusion criteria.
- Sources searched for economic evaluations should include specialised databases of economic evaluations such as NHS EED and HEED.
- The BMJ checklist is the most general quality assessment tool. However, the study question and inclusion criteria may well require a more specific quality assessment checklist.
- Identified economic evaluations are usually too heterogeneous and are unlikely to report adequate details for a meta-analysis of results. A narrative synthesis is usual.
- Disaggregated results should be reported as well as incremental cost-effectiveness ratios in order to facilitate the interpretation of the results.

REFERENCES

1. Lavis J, Davies H, Oxman A, Denis JL, Golden-Biddle K, Ferlie E. Towards systematic reviews that inform health care management and policy-making. *J Health Serv Res Policy* 2005;10 Suppl 1:35-48.

2. National Institute for Health and Clinical Excellence. *Guide to the methods of technology appraisal. June 2008*. London: National Institute for Health and Clinical Excellence; 2008.

3. Rodgers M, McKenna C, Palmer S, Chambers D, Van Hout S, Golder S, et al. Curative catheter ablation in atrial fibrillation and typical atrial flutter: systematic review and economic evaluation [in press]. *Health Technol Assess* 2008.

4. Woolacott NF, Jones L, Forbes CA, Mather LC, Sowdon AJ, Song FJ, et al. The clinical effectiveness and cost-effectiveness of bupropion and nicotine replacement therapy for smoking cessation: a systematic review and economic evaluation. *Health Technol Assess* 2002;6:1-245.

5. Wilby J, Kainth A, Hawkins N, Epstein D, McIntosh H, McDaid C, et al. Clinical effectiveness, tolerability and cost-effectiveness of newer drugs for epilepsy in adults: a systematic review and economic evaluation. *Health Technol Assess* 2005;9:1-172.

6. Briggs A, Claxton K, Sculpher M. *Decision modelling for health economic evaluation*. Oxford: Oxford University Press; 2006.

7. Donaldson C, Mugford M, Vale L, editors. *Evidence-based health economics from effectiveness to efficiency in systematic review*. London: BMJ Books, 2002.

8. Anderson R. Systematic reviews of economic evaluations: utility or futility? [submitted].

9. Carande-Kulis VG, Maciosek MV, Briss PA, Teutsch SM, Zaza S, Truman BI, et al. Methods for systematic reviews of economic evaluations for the Guide to Community Preventive Services. Task Force on Community Preventive Services. *Am J Prev Med* 2000;18:75-91.

10. Schwappach DL, Boluarte TA, Suhrcke M. The economics of primary prevention of cardiovascular disease - a systematic review of economic evaluations. *Cost Eff Resour Alloc* 2007;5:5.

11. Barham L, Lewis D, Latimer N. One to one interventions to reduce sexually transmitted infections and under the age of 18 conceptions: a systematic review of the economic evaluations. *Sex Transm Infect* 2007;83:441-6.

12. Drummond MF, Sculpher MJ, Torrance GW, O'Brien BJ, Stoddart GL. *Methods for economic health evaluation of health care programmes*. 3rd ed. Oxford: Oxford University Press; 2005.

13. Centre for Reviews and Dissemination. *NHS EED (NHS Economic Evaluation Database) [internet]*. York: Centre for Reviews and Dissemination, University of York; [cited 2008 9 Sep]. Available from: www.crd.york.ac.uk/crdweb/

14. *HEED: Health Economic Evaluations Database [internet]*. Chichester: John Wiley & Sons Ltd; [cited 2008 22 Apr]. Available from: www3.interscience.wiley.com/cgi-bin/mrwhome/114130635/HOME

15. Philips Z, Ginnelly L, Sculpher M, Claxton K, Golder S, Riemsma R, et al. Review of guidelines for good practice in decision-analytic modelling in health technology assessment. *Health Technol Assess* 2004;8:1-158.

16. Phillips B, Ball C, Sackett D, Badenoch D, Straus S, Haynes B, et al. *Oxford Centre for Evidence-based Medicine Levels of Evidence (May 2001)*; 2001. Available from: www.cebm.net/index.aspx?o=1025

17. Collett D. *Modelling survival data in medical research*. 2nd ed. Boca Raton, FL: CRC Press; 2003.

18. Glick H, Podolsky D, Schulamn K. Trial-based economic evaluations: an overview of design and analysis. In: Drummond MF, McGuire A, editors. *Economic evaluation in health care: merging theory with practice*. Oxford: Oxford University Press; 2001. p. 113-40.

19. Krahn M, Gafni A. Discounting in the economic evaluation of health care interventions. *Med Care* 1993;31:403-18.

20. Finkler SA. The distinction between costs and charges. *Ann Intern Med* 1982;96:102-9.

21. Brazier J, Ratcliffe J, Salomon J, Tsuchiya A. *Measuring and valuing health benefits for economic evaluation*. Oxford: Oxford University Press; 2008.

22. Marra CA, Woolcott JC, Kopec JA, Shojania K, Offer R, Brazier JE, et al. A comparison of generic, indirect utility measures (the HUI2, HUI3, SF-6D, and the EQ-5D) and disease-specific instruments (the RAQoL and the HAQ) in rheumatoid arthritis. *Soc Sci Med* 2005;60:1571-82.

23. Briggs AH, Wonderling DE, Mooney CZ. Pulling cost-effectiveness analysis up by its bootstraps: a non-parametric approach to confidence interval estimation. *Health Econ* 1998;6:327-40

24. Briggs AH, O'Brien BJ, Blackhouse G. Thinking outside the box: recent advances in the analysis and presentation of uncertainty in cost-effectiveness studies. *Annu Rev Public Health* 2002;23:377-401.

25. Thomson SG, Nixon RM. How sensitive are cost-effectiveness analyses to choice of parametric distributions? *Med Decis Making* 2005;25:416-23.

26. Boulenger S, Nixon J, Drummond M, Ulmann P, Rice S, de Pouvourville G. Can economic evaluations be made more transferable? *Eur J Health Econ* 2005;6:334-46.

27. Drummond MF, Jefferson TO. Guidelines for authors and peer reviewers of economic submissions to the BMJ. The BMJ Economic Evaluation Working Party. *BMJ* 1996;313:275-83.

28. Thurston SJ, Craig D, Wilson P, Drummond MF. Increasing decision-makers' access to economic evaluations: alternative methods of communicating the information. *Int J Technol Assess Health Care* 2008;24:151-7.

29. Jefferson T, Demicheli V, Vale L. Quality of systematic reviews of economic evaluations in health care. *JAMA* 2002;287:2809-12.

30. Shemilt I, Mugford M, Byford S, Drummond M, Eisenstein E, Knapp M, et al. *The Campbell Collaboration economics methods policy brief. Version 1.0 - April 2008*: The Campbell Collaboration; 2008. Available from: www.campbellcollaboration.org/resources/methods_policy_briefs.php

31. Shemilt I, Mugford M, Byford S, Drummond M, Eisenstein E, Knapp M, et al. Chapter 15: Incorporating economics evidence. In: Higgins JPT, Green S, editors. *Cochrane Handbook for Systematic Reviews of Interventions Version 5.0.0 (updated February 2008)*: The Cochrane Collaboration; 2008. Available from: www.cochrane-handbook.org

32. Bolin K LB, Willers S. The cost utility of bupropion in smoking cessation health programs: simulation model results for Sweden. *Chest* 2006;129:651-60.

33. Cornuz J, Gilbert A, Pinget C, McDonald P, Slama K, Salto E, et al. Cost-effectiveness of pharmacotherapies for nicotine dependence in primary care settings: a multinational comparison. *Tob Control* 2006;15:152-9.

34. Nielsen K, Fiore MC. Cost-benefit analysis of sustained-release bupropion, nicotine patch, or both for smoking cessation. *Prev Med* 2000;30:209-16.

CHAPTER 6

INCORPORATING QUALITATIVE EVIDENCE IN OR ALONGSIDE EFFECTIVENESS REVIEWS

6.1 INTRODUCTION 221

6.2 WHAT IS QUALITATIVE RESEARCH? 221
- **6.2.1 Options for utilising qualitative research** 222

6.3 IDENTIFICATION OF QUALITATIVE STUDIES 223
- **6.3.1 Using bibliographic databases** 223
- **6.3.2 Other sources** 224
- **6.3.3 Sampling methods** 224

6.4 ASSESSMENT OF QUALITATIVE RESEARCH 225
- **6.4.1 How should quality be assessed?** 225
- **6.4.2 How should quality assessment be used?** 227
- **6.4.3 When should quality assessment be carried out?** 227

6.5 SYNTHESIS OF QUALITATIVE RESEARCH 228
- **6.5.1 Methods of synthesis** 229

6.6 USING QUALITATIVE FINDINGS TO HELP EXPLAIN, INTERPRET AND IMPLEMENT FINDINGS FROM EFFECTIVENESS REVIEWS 230
- **6.6.1 Findings from one or more qualitative studies** 230
- **6.6.2 Findings from a synthesis of qualitative studies** 230
- **6.6.3 Combining qualitative and quantitative syntheses** 231

6.7 FUTURE DEVELOPMENTS 232

REFERENCES 233

6.1 INTRODUCTION

This chapter focuses on the identification, assessment and synthesis of qualitative studies to help explain, interpret and implement the findings from effectiveness reviews. There is growing recognition of the contribution that qualitative research can make to reviews of effectiveness, particularly in relation to understanding the what, how and why.[1] This includes shaping questions of importance to end users, understanding the mechanisms behind effectiveness or ineffectiveness, understanding heterogeneous results, identifying factors that impact on the implementation of an intervention, describing the experience of people receiving the interventions, and providing participants' subjective evaluations of outcomes. For example, what is it about the workings of a stroke unit that result in better survival rates? What elements of a community-based programme to prevent falls enable older people to retain their independence? How was the process of care perceived and what counts as a successful outcome for those receiving the intervention? An approach that uses qualitative research to address questions such as these helps to ensure that reviews are of maximum value in the decision-making process.

Despite recognition of the importance of qualitative research to effectiveness reviews, so far the number of available examples is relatively small. Poor availability may reflect a relative lack of interest in applying review methods to qualitative research and/or lack of consensus about whether it is appropriate to do so. In recent years new approaches and techniques for reviewing qualitative studies have emerged, although debates about appropriateness continue. Because review methods are not well-developed or tested we outline various options for consideration, and provide references which should be consulted where more detailed information is required. We also include worked examples; where possible these have been selected because they are directly linked or related to reviews of effectiveness. But because these are few in number, other types of example are included, such as stand-alone reviews of qualitative research.

The process for reviewing qualitative studies has been argued to be an iterative one which might not proceed in a linear way.[2, 3] So, although we have chosen to structure the chapter according to the recognised stages in a systematic review (study identification, quality assessment and synthesis), in practice the process may deviate.

6.2 WHAT IS QUALITATIVE RESEARCH?

Qualitative research is concerned with the subjective world and offers insight into social, emotional, and experiential phenomena.[4] The aim is to draw out understandings and perceptions, to explore the features of settings and culture and to understand the linkages between process and outcomes. Most qualitative studies are small scale, focusing on a single or small number of cases, and they provide depth and contextualised detail. Qualitative research is not a single method but includes a range of designs such as interviews, direct observation, analysis of texts/documents or of audio/video recorded speech or behaviour.[2, 5] Choice of method is often determined by a particular theoretical perspective, such as phenomenology which provides a framework for the research.[2]

More recently, the relevance of qualitative research to the assessment of health interventions, especially those that are complex, has been recognised. As a result qualitative and quantitative methods are increasingly being used together in primary evaluative research (mixed-method). For example qualitative methods have been used to understand participants' experiences in a trial evaluating a computerised decision support tool for patients with atrial fibrillation being considered for anti-coagulation treatment.[6] The main reason for the adoption of mixed-methods in primary research is to enhance relevance in the decision-making process.

Important to note is that some primarily quantitative studies provide information of a qualitative form such as observations or quotes, which are unlikely to be the result of a formal research process. Sometimes no detail is reported of how these 'data' have been collected or analysed and researchers need to be cautious if using such information. This issue has been discussed with respect to the implementation of community-based interventions to reduce unintentional injuries in children and young people.[7]

6.2.1 Options for utilising qualitative research

The approach to utilising qualitative research needs to be decided at an early stage, as it will impact on subsequent stages of the review, especially searching and synthesis. Outlined below are three options for including qualitative evidence in/alongside quantitative effectiveness reviews; the first offers a more informal approach; the second involves a formal synthesis of the qualitative findings. Both options treat qualitative and quantitative evidence as complementary with the qualitative evidence offering an explanation for, and interpretation of, the quantitative findings; the third combines the findings from the quantitative and qualitative syntheses. Used in this way the qualitative evidence does not contribute directly to the effectiveness data. If researchers are interested in including both quantitative and qualitative research to address questions of effectiveness they might consider using techniques or approaches capable of combining different types of research evidence such as Bayesian meta-analysis,[8] critical interpretive synthesis[9] or realist synthesis.[10, 11] There are also a number of useful guides discussing various methods for synthesising complex bodies of evidence.[2, 12]

Option 1. Use the findings from one or more qualitative studies in the discussion and interpretation of the results of the quantitative studies to help make sense of, or place the review findings in context. Usually (although not always) the qualitative evidence will be linked to the quantitative studies included in the effectiveness review. Qualitative and quantitative evidence might be included in the same publication or in separate but associated publications.

Option 2. Undertake a review of qualitative studies alongside the review of quantitative studies and use the formal qualitative synthesis to interpret the findings of the quantitative synthesis (sometimes referred to as parallel synthesis). Researchers might choose to include qualitative research embedded within the quantitative studies or stand-alone qualitative studies that address the question of interest.

Option 3. Where reviews of both quantitative and qualitative evidence are undertaken there is also the option to combine the results of the two syntheses. This approach is sometimes referred to as multi-level, sequenced, cross-design or meta-synthesis.[2, 13]

6.3 IDENTIFICATION OF QUALITATIVE STUDIES

In line with the three options discussed in *Section 6.2.1 Options for utilising qualitative research,* researchers can choose to include qualitative evidence that is embedded within quantitative studies (i.e. mixed-method) or associated with the quantitative evaluation, or choose to include qualitative studies that address issues of direct relevance, but are not linked or associated with the quantitative evaluation.

A search for quantitative studies will often identify associated or linked studies using qualitative methods. However relying solely on this approach is questionable, as the studies are identified by chance rather than in a structured systematic way.[14] A very broad approach can be used, where the search strategy consists solely of subject and topic terms without specifying the study type(s) of interest. Both quantitative and qualitative studies would be identified, but this method is likely to result in large numbers of records being retrieved. This approach is routinely used at the EPPI-Centre[15] when carrying out linked systematic reviews of qualitative and quantitative studies.

6.3.1 Using bibliographic databases

The methods and tools available to identify qualitative studies, especially from electronic databases are much less well developed than those available for identifying quantitative studies, especially RCTs.[16] There are as yet no registers of qualitative studies, nor do existing RCT registers record whether qualitative data were also collected.

Qualitative research may be given a descriptive or creative title that makes retrieval using standard search techniques difficult. Database abstracts, where included, are often not structured and can have variable content, which further complicates their identification.[17] Many studies lack an abstract; for example a search for qualitative studies on support for breastfeeding found that 23 per cent of the records screened did not include an abstract.[18] This means that many full papers will need to be retrieved to make decisions about inclusion, as in a review of adherence to tuberculosis treatment where over 600 full text articles were screened.[19]

There are differences in how qualitative research is, and has been, indexed in electronic databases that have implications for searching. While MEDLINE introduced the subject heading 'qualitative research' in 2003, CINAHL has had a wide range of detailed subject headings suitable for indexing qualitative research for much longer. For example, the headings 'qualitative studies' and 'grounded theory' were both introduced in 1988. Assuming that the topic of interest falls within their scope, then searching both MEDLINE and CINAHL is likely to be important. For CINAHL using broad free text terms – 'qualitative', 'findings', 'interviews' – in conjunction with topic specific thesaurus terms may be adequate to identify qualitative studies.[20]

When searching electronic databases it is important to be aware that uncertainty remains about how consistently the term 'qualitative research' is being used.[21]

The indexing of qualitative papers is generally viewed as less accurate than the indexing of quantitative studies, both in terms of whether suitable index terms are available and whether they have been applied correctly.[22] In a comparison of three strategies across six databases – using thesaurus terms, free-text terms and broad-based terms – only

four per cent of records were actually relevant, and all three strategies had to be used in combination to avoid missing potentially relevant records.[22] The identification of a high proportion of irrelevant studies is likely to be due in part to poor indexing.

Search filters for identifying qualitative research are available for use in a number of electronic databases. For researchers wishing to use existing search filters, two resources are particularly helpful. The Hedges Project, based at McMaster University and funded by the US National Library of Medicine, develops search strategies ('hedges') to improve retrieval of study reports from large biomedical bibliographic databases such as MEDLINE, EMBASE, PsycINFO, and CINAHL. A range of filters is available for each database, together with information about the research papers underlying the development of the filter (http://hiru.mcmaster.ca/hiru/HIRU_Hedges_home.aspx). The webpages are arranged by database so, for example, on the pages about MEDLINE a number of qualitative filters of varying degrees of sensitivity, specificity, accuracy and precision are presented in a tabular format. Where the aim is to comprehensively identify all papers on a topic, a filter with high sensitivity should be selected. If it is of less importance to identify all papers, a filter with high precision will usually be appropriate. Reading the accompanying research paper that describes the development of each filter can help researchers to choose the filter that is most appropriate.

The InterTASC Information Specialists' Sub-Group (ISSG) Search Filter Resource offers another useful website (www.york.ac.uk/inst/crd/intertasc/). The ISSG is a group of information professionals supporting research groups within England and Scotland producing technology assessments for NICE. This resource includes a wider range of filters than the Hedges Project, but in some cases the filter is not displayed in full on the website, although details of where it is published are given.

6.3.2 Other sources

Given the identified deficiencies in the indexing of studies and in the study filters currently available, additional techniques such as Internet searching, personal contact with researchers, handsearching and reference checking are important (see *Chapter 1*).

6.3.3 Sampling methods

There is currently no consensus as to whether the searches undertaken to identify qualitative studies need to be as comprehensive in their coverage as those undertaken to identify quantitative studies, although they should be as systematic, explicit and reproducible as possible.[23]

Where the number of available studies may be simply too large to work through, researchers may decide to adopt a strategy for limiting the number of included studies. Options include purposive and/or theoretical sampling where papers are selected for inclusion on the basis of particular criteria such as rich description or conceptual clarity. Alternatively, random sampling can be used. Examples of purposive sampling are provided in reviews of caring[24] and access to health care.[9]

The search strategy and, where used, the methods for sampling need to be documented clearly. It is important to outline the steps taken and discuss the potential impact of

any limitations. Proposed standards for reporting literature searches are available, and provide a useful resource.[25]

6.4 ASSESSMENT OF QUALITATIVE RESEARCH

The application of quality criteria to qualitative research is widely debated, although many accept the need for clear and transparent approaches for judging the quality or credibility of research. For example, it has been noted that the distinguishing mark of all 'good' research is the awareness and acknowledgement of error and, that what flows from this is the necessity of establishing procedures which will minimize the effect such errors may have on what counts as knowledge.[26] It is less clear whether consensus can be reached over an agreed set of principles for judging quality. Qualitative researchers from different disciplines and from different theoretical backgrounds may have different criteria for assessing the quality of a study.[27] Some argue that quality cannot be determined by following prescribed formulas[28] or that it is fruitless to try to set standards for qualitative research as such.[29] Others, accepting the need for structured procedures, argue for more rigorous use and reporting of analytical approaches which improve reliability and validity.[30] Others have suggested there are general questions that can be asked to judge validity and reliability in qualitative research, but that these are not readily codified.[31] It has also been argued that quality assessment should take account of theory in the design of the research, analysis, and interpretation of the data.[32]

6.4.1 How should quality be assessed?

Despite lack of consensus about quality assessment a number of different tools and techniques are now available. Over one hundred sets of proposals on quality in qualitative research have been identified,[33] a subset of which have been reviewed,[34] including five that were developed specifically for use in systematic reviews.[35-39] The majority of tools available are generic, and to date there have been few attempts to develop method specific approaches. This is despite arguments that different qualitative methods need to be appraised in different ways.[40]

Some issues in using structured approaches were illustrated in a recent study.[41] Two structured methods – the Critical Appraisal Skills Programme (CASP) tool,[42] and the Quality Framework[34] – were systematically compared with an approach based on unprompted judgement (where experienced qualitative researchers relied on their own expertise to make judgements of quality). Each approach was used to assess twelve qualitative studies investigating support for breastfeeding. Agreement between researchers and between methods was slight, and importantly researchers disagreed on the quality of the studies, whether papers were actually reporting qualitative research and whether the study was relevant to the review question. Because answering questions about quality is largely a subjective process involving judgement, it may lead to differences both between researchers and methods.

In addition, the Quality Framework was criticised for its length and complexity, which is likely to impact on its use in future systematic reviews. The authors identified a need for continued debate and empirical research into the use of quality assessment. Similarly, the authors of a recent review who attempted to apply two different quality frameworks, concluded that further methodological work is needed to produce clear guidance about how quality appraisal should be undertaken.[32]

An innovative approach, developed to appraise qualitative studies for inclusion in a set of reviews focusing on peoples' experiences and perspectives, uses generic methodological quality criteria tailored to the specific review question.[43] It is designed to help researchers assess to what extent studies may have distorted, misrepresented or simply missed people's experiences and perspectives. The authors have published a series of reports that outline how the approach has been applied in practice.[44, 45]

Box 6.1: Appraisal tools

Popay, Rogers & Williams (1998)[39]	Primary question relates to the appropriateness of the methods used. This is followed by a detailed assessment of methodological soundness.
Critical Appraisal Skills Programme (1998)[42]	10 questions relating to rigour, credibility and relevance.
Quality Framework (2003)[34]	18 questions relating to 9 key areas: findings; design; sample; data collection; analysis; reporting; reflexivity and neutrality; ethics and auditability.
Prompts for appraising qualitative research (2004)[33]	Generic set of prompts relating to aspects of reporting and aspects of study design and execution.
Long & Godfrey (2004)[38]	A tool to explore descriptive and evaluative elements of a study. 34 questions across 4 key areas: phenomenon studied and context; ethics; data collection, analysis and potential researcher bias; policy and practice implications.
Walsh & Downe (2006)[48]	Set of prompts relating to 8 key areas: scope and purpose; design; sampling strategy; analysis; interpretation; reflexivity; ethical dimensions; relevance and transferability.

A structured review of reports published between 1988 and 2004, appraising and synthesising qualitative studies in health and health care is available.[46] The authors found that over 60% of the 42 reviews included either explicitly stated that quality appraisal was not carried out or failed to report any appraisal of studies. Interestingly where quality appraisal was used, in all but one case the instrument or criteria were modified, suggesting that available methods are difficult to apply in practice. Others have opted to construct their own criteria for assessing rigor as part of the review process.[47]

Box 6.1 lists some of the different appraisal tools that have been developed explicitly for use in systematic reviews and/or have been used for that purpose (this is not a comprehensive list). Researchers interested in carrying out quality assessment, might consider using one or more of these tools.

6.4.2 How should quality assessment be used?

Quality assessment has been used to establish a quality threshold below which studies will be excluded, or to distinguish between studies in terms of overall contribution.[32, 36, 49] There is no consensus as to which approach is preferable. Quality assessment can also be used to gain an understanding of the relative strengths and weaknesses of the body of evidence and taken into account during the process of synthesis.

Some have reported that better quality studies appear to make stronger contributions to the synthesis[19, 49] or that weaker studies contribute nothing substantially different from the stronger studies.[32] Sensitivity analysis has been used to explore the relationship between the quality of qualitative studies and contribution to review findings.[50] The analysis was based on 62 primary studies from five reviews, and suggested that studies judged to be of low quality contributed little to the overall review findings. This appears to be the first attempt to apply sensitivity analysis to the question of quality in qualitative research and further assessment is required. However, the findings are consistent with the more descriptive accounts offered about study quality and overall contribution to synthesis.[19, 32, 49]

6.4.3 When should quality assessment be carried out?

The use of quality assessment is further complicated by debate around when it should be carried out. The need for appraisal of studies before the synthesis has been queried.[51] The authors of one qualitative synthesis reported that the necessity of prior quality appraisal was a moot point.[36] They did go on to comment however that the appraisal process was a useful prelude to the synthesis because it helped to screen out inappropriate and poor quality studies. Clearly, if quality assessment is to be used to establish a quality threshold then assessment will need to take place before the synthesis.

6.5 SYNTHESIS OF QUALITATIVE RESEARCH

This section focuses on the formal synthesis of qualitative research. General debate about the appropriateness of combining qualitative studies continues, and more specifically whether different types of qualitative research, based on different theoretical assumptions and methods should be combined. Sometimes authors' claims about the theoretical underpinnings of their work are not always closely related to the methods actually used. A recent investigation suggested it is very difficult to draw firm boundaries around what is, and is not, a particular type of qualitative research as many authors failed to give any definition.[52] Despite these problems this same investigation also found that it is possible to synthesise across different traditions[52] and indeed some review teams consider the combining of data from multiple theoretical and methodological traditions a strength of the review.[24]

Box 6.2: Methods proposed for the synthesis of qualitative evidence

Synthesis method	Description
Meta-ethnography[51]	A set of techniques for synthesising qualitative studies. It involves the selection, comparison and analysis of studies to create new interpretations or concepts. Key stages include the reading and re-reading of studies; determining how the studies are related by listing key concepts and comparing and contrasting them; translating the studies into one another and synthesising the translations to identify concepts which go beyond individual accounts and can be used to produce a new interpretation.<br><br>It has been used to address questions about the meanings of medicines,[54 55] the barriers and facilitators of adherence to TB treatment,[56 19] lay experiences of diabetes and diabetes care,[36] children's perspectives of growing up in disadvantage[57] and teenage mothers' experiences of their lives.[58]
Thematic analysis/ synthesis[43]	The identification of important or recurrent themes. Findings are summarised under thematic headings. Information is tabulated allowing identification of prominent themes and offering structured ways of dealing with the data in each theme. More recently the method has been refined, such that a new method – thematic synthesis – has emerged.[43, 59]<br><br>It has been used to address questions about barriers to and facilitators of healthy eating amongst children;[60] young people, pregnancy and social exclusion[44] and accidental injury, risk taking behaviour and the social circumstances in which young people live.[61]

(Continued)

Synthesis method	Description
Grounded theory (constant comparative method)[62]	Grounded theory was originally developed for use with primary studies and describes methods for sampling, data collection and analysis. Its potential application to the synthesis of multiple qualitative studies has recently been reported.[2, 63]<br><br>One particular element – the constant comparative method – has received most attention as it offers a set of procedures for analysing qualitative evidence.[64]<br><br>It has been used to address questions about women's experiences of domestic violence,[65] living with HIV infection[66] and caring in nursing.[24]
Qualitative research synthesis: Qualitative meta-synthesis[3]	A set of techniques for the interpretive integration of qualitative research findings.
Content analysis[67]	A systematic technique for categorising data into themes and counting frequency of themes.
Case survey[68]	Formal process for coding data from qualitative cases into a quantitative form.
Qualitative comparative analysis[69]	A method for summarising and comparing data from case studies using Boolean logic.
Qualitative research synthesis: Qualitative meta-summary[3]	A set of techniques for the quantitative aggregation of qualitative research findings.
Narrative synthesis[70]	A general framework and specific tools and techniques that help to increase transparency and trustworthiness. Can be applied to reviews of quantitative or qualitative research as individual tools and techniques can be selected according to the type of study design and data included in the review.<br><br>The framework has been applied to questions relating to the implementation of domestic smoke alarms.[70]

6.5.1 Methods of synthesis

A number of different methods have been proposed for the synthesis of qualitative findings, many based on approaches used in primary research.[2, 3, 12]

Some of the methods maintain the qualitative form of the evidence such as meta-ethnography and some involve converting qualitative findings into a quantitative form such as content analysis. It has been argued that perhaps the least useful way of dealing with qualitative data is to turn them into quantitative data.[53] *Box 6.2* outlines some of the methods proposed. This is not a comprehensive list. Choice of method will

be influenced by a number of factors including the question posed, likely number of relevant studies and undoubtedly the knowledge and expertise of the team undertaking the review.

Many of the methods are still at a developmental stage and there are relatively few examples of their application. An overview of syntheses of qualitative research published between 1988 and 2004 identified 42 such syntheses of which meta-ethnography was the method most commonly used.[46] Consequently there is as yet little evaluation of the relative strengths and weaknesses of available methods and few guidelines exist for judging quality. However, the recent development of a comprehensive framework for good practice offers potential, both for researchers wanting to carry out a synthesis and for those wishing to use the findings of a synthesis.[52]

It is important to note that there are many different terms used to describe the various methods, some of which have been applied inconsistently. Some terms such as 'qualitative meta-analysis', 'meta-study' and 'meta-synthesis' appear to have been used in an over-arching way to describe any synthesis of qualitative research. The use of the term meta-synthesis to describe any synthesis of qualitative research has been criticised on the grounds that it is not specific to qualitative research, and is frequently technically incorrect, since what is being attempted is not at the meta-level of the synthesis but at the meta-level of the included studies (Mary Dixon-Woods, personal communication).

6.6 USING QUALITATIVE FINDINGS TO HELP EXPLAIN, INTERPRET AND IMPLEMENT FINDINGS FROM EFFECTIVENESS REVIEWS

The approach for integrating the findings from qualitative studies with the effectiveness review will depend on which of the options outlined in *Section 6.2.1 Options for utilising qualitative research* is selected.

6.6.1 Findings from one or more qualitative studies

Using the findings from one or more qualitative studies in the narrative discussion of the results of the quantitative studies is possibly the most straightforward approach, as it does not involve a separate synthesis of the qualitative studies. This method was used in a review of population tobacco control interventions.[71] Relevant qualitative studies were searched for, identified and quality assessed as part of the overall review process and used to help interpret the quantitative findings. The qualitative findings were helpful in understanding the experience of staff subjected to smoking restrictions in the workplace. Staff viewed restrictions as divisive, as they impacted differentially across staff grades.

6.6.2 Findings from a synthesis of qualitative studies

Findings from a synthesis of qualitative studies can be used to interpret the findings of the quantitative synthesis. The best available examples are two reviews of qualitative studies, which address questions of adherence with tuberculosis treatment.[32, 56] Both are

linked to a Cochrane review of directly observed therapy (DOT) for treating tuberculosis which found no evidence that direct observation of people taking their medication was better than self administered treatment.[72] The reviews of qualitative studies aimed to help explain these findings as well as helping to understand the factors influencing (negatively or positively) adherence with tuberculosis treatment. Both reviews identified a number of factors that provide fresh insights into the reasons for poor adherence. In turn these factors can be used to inform the development of new interventions as well as inform the implementation of specific measures to improve adherence. With regard to interpreting the quantitative findings, the qualitative synthesis offered huge insight. For example, in helping to understand that patient experience was frequently negative and dehumanizing in hospital or clinic based DOT where participants had less choice or flexibility over treatment, often having to travel during work hours and not being in control of their drug supply. The authors suggest that costs to the patient in terms of time and resources and the dehumanizing nature of the intervention may help to explain why clinic based DOT tended to be less successful than the alternatives.[32]

6.6.3 Combining qualitative and quantitative syntheses

The synthesis of qualitative studies can be brought together in a formal way with the synthesis of quantitative studies. Because the model mixes methods at the review level, it preserves the integrity of the findings from each study type. This approach developed by the EPPI-Centre has been used in a series of reviews focusing on young people and healthy eating[73] as well as reviews in other topic areas such as HIV health promotion[45] and young people, pregnancy and social exclusion.[44]

The review focusing on promotion of healthy eating is used to illustrate the approach.[73] The findings from each synthesis were juxtaposed. This was done by using a matrix that enabled the qualitative findings relating to young peoples' views about healthy eating (and implied recommendations) to be compared and contrasted against the actual interventions evaluated in the quantitative studies. The comparative analysis was guided by three questions, relating to which interventions matched the recommendations derived from young peoples' views; which recommendations had yet to be evaluated using rigorous methods and whether interventions that matched recommendations demonstrated larger effects. One theme emerged relating to the promotion of fruit and vegetables as tasty rather than healthy, with minimal emphasis on health messages. Five evaluation studies matched this theme and subgroup analysis suggested that the biggest increase in vegetable consumption was in the studies with minimal focus on health messages. A research gap was identified relating to the theme that fruit and vegetables should not be promoted in the same way.

In bringing together the results from the two sets of studies the review team were able to demonstrate added value both in helping to understand why certain types of intervention might be more effective than others, and in guiding recommendations for future research. This approach has the potential to involve any number of linked syntheses, addressing different questions that are important to the effectiveness of an intervention.

6.7 FUTURE DEVELOPMENTS

Despite recognition of the importance of qualitative research to effectiveness reviews, the methods available are not fully developed or well established. Teams undertaking reviews of qualitative studies can usefully add to knowledge by publishing their own experiences of the process.

Summary: Incorporating qualitative evidence in or alongside systematic reviews of effect

- Qualitative research can enhance the utility of reviews of effectiveness in a variety of ways such as shaping questions of importance to end users, understanding the mechanisms behind effectiveness or ineffectiveness, understanding heterogeneous results, identifying factors that impact on the implementation of an intervention, describing the experience of people receiving the interventions, and providing participants' subjective evaluations of outcomes.
- Qualitative research is not a single method but includes a range of designs such as interviews, direct observation, analysis of texts/documents or of audio/video recorded speech or behaviour. Choice of method is often determined by a particular theoretical perspective, such as phenomenology.
- Whichever approach guides the search for qualitative research, it is important to be transparent and to document clearly the steps taken and discuss the potential impact of any limitations.
- Despite lack of consensus over the value of quality assessment, structured approaches for judging validity and reliability in qualitative research have been developed, used in practice and compared empirically.
- A number of methods have been proposed for the synthesis of qualitative studies, but little evaluation undertaken.
- There are three main approaches to integrating the findings from qualitative studies within an effectiveness review. Use the findings from one or more qualitative studies in the narrative discussion of the quantitative findings; use the findings from the synthesis of qualitative studies to interpret the findings of the quantitative synthesis or bring together in a formal way the synthesis of qualitative studies with the synthesis of quantitative studies.

REFERENCES

1. Sheldon TA. Making evidence synthesis more useful for management and policy-making. *J Health Serv Res Policy* 2005;10 Suppl 1:1-5.

2. Pope C, Mays N, Popay J. *Synthesizing qualitative and quantitative health evidence: a guide to methods*. Maidenhead: Open University Press; 2007.

3. Sandelowski M, Barroso J. *Handbook for synthesizing qualitative research*. New York, NY: Springer; 2006.

4. Giacomini MK, Cook DJ, for the Evidence-Based Medicine Working Group. Users' guide to the medical literature. XXIII. Qualitative research in health care. A. Are the results of the study valid? *JAMA* 2000;284:357-62.

5. Pope C, Mays N, editors. *Qualitative research in health care*. 3rd ed. Oxford: Blackwell 2006.

6. Murtagh MJ, Thomson RG, May CR, Rapley T, Heaven BR, Graham RH, et al. Qualitative methods in a randomised controlled trial: the role of an integrated qualitative process evaluation in providing evidence to discontinue the intervention in one arm of a trial of a decision support tool. *Qual Saf Health Care* 2007;16:224-9.

7. Roen K, Arai L, Roberts H, Popay J. Extending systematic reviews to include evidence on implementation: methodological work on a review of community-based initiatives to prevent injuries. *Soc Sci Med* 2006;63:1060-71.

8. Roberts KA, Dixon-Woods M, Fitzpatrick R, Abrams KR, Jones DR. Factors affecting uptake of childhood immunisation: a Bayesian synthesis of qualitative and quantitative evidence. *Lancet* 2002;360 1596-9.

9. Dixon-Woods M, Cavers D, Agarwal S, Annandale E, Arthur A, Harvey J, et al. Conducting a critical interpretive synthesis of the literature on access to healthcare by vulnerable groups. *BMC Med Res Methodol* 2006;6:35.

10. Pawson R, Greenhalgh T, Harvey G, Walshe K. *Realist synthesis: an introduction*. Manchester: ESRC Research Methods Programme; 2004.

11. Pawson R, Greenhalgh T, Harvey G, Walshe K. Realist review: a new method of systematic review designed for complex policy interventions. *J Health Serv Res Policy* 2005;10 Suppl 1:21-34.

12. Dixon-Woods M, Agarwal S, Young B, Jones D, Sutton A. *Integrative approaches to qualitative and quantitative evidence*. London: NHS Health Development Agency; 2004.

13. Oliver S, Harden A, Rees R, Shepherd J, Brunton G, Garcia J, et al. An emerging framework for including different types of evidence in systematic reviews for public policy. *Evaluation* 2005;11:428-46.

14. Higgins JPT, Green S, (editors). *Cochrane Handbook for Systematic Reviews of Interventions. Version 5.0.0 [updated February 2008]*: The Cochrane Collaboration; 2008. Available from: www.cochrane-handbook.org

15. *EPPI-Centre [Internet]*. London: The Evidence for Policy and Practice Information, Social Science Research Unit, Institute of Education, University of London; c2008. [cited 2008 Apr 17]. Available from: http://eppi.ioe.ac.uk/cms/

16. Dixon-Woods M, Fitzpatrick R. Qualitative research in systematic reviews. Has established a place for itself. *BMJ* 2001;323:765-6.

17. Evans D. Database searches for qualitative research. *J Med Libr Assoc* 2002;90:290–3.

18. Dixon-Woods M, Bonas S, Booth A, Jones DR, Miller T, Sutton AJ, et al. How can systematic reviews incorporate qualitative research? A critical perspective. *Qualitative Research* 2006;6:27-44.

19. Atkins S, Lewin S, Smith H, Engel M, Fretheim A, Volmink J. Conducting a meta-ethnography of qualitative literature: lessons learnt. *BMC Med Res Methodol* 2008;8:21

20. Flemming K, Briggs M. Electronic searching to locate qualitative research: evaluation of three strategies. *J Adv Nurs* 2007;57:95–100.

21. Grant MJ. How does your searching grow? A survey of search preferences and the use of optimal search strategies in the identification of qualitative research. *Health Info Libr J* 2004;21:21-32.

22. Shaw RL, Booth A, Sutton AJ, Miller T, Smith JA, Young B, et al. Finding qualitative research: an evaluation of search strategies. *BMC Med Res Methodol* 2004;4:5.

23. Booth A. Cochrane or cock-eyed? How should we conduct systematic reviews of qualitative research? [monograph online]. In: *Qualitative Evidence-Based Practice Conference*; 2001 May 14-16; Coventry University.

24. Finfgeld-Connett D. Meta-synthesis of caring in nursing. *J Clin Nurs* 2008;17:196-204.

25. Booth A. "Brimful of STARLITE": toward standards for reporting literature searches. *J Med Libr Assoc* 2006;94:421-9.

26. Oakley A. *Experiments in knowing: gender and method in social sciences*. Cambridge: Policy Press; 2000.

27. Sandelowski M, Docherty S, Emden C. Focus on qualitative methods. Qualitative metasynthesis: issues and techniques. *Res Nurs Health* 1997;20:365-71.

28. Buchanan DR. An uneasy alliance: combining qualitative and quantitative research methods. *Health Educ Q* 1992;19:117-35.

29. Howe K, Eisenhart M. Standards for qualitative (and quantitative) research: a prolegomenon. *Educational Researcher* 1990;19:2-9.

30. Seale S, Silverman D. Ensuring rigour in qualitative research. *Eur J Pub Health* 1997;7:389-4.

31. Murphy E, Dingwall R, Greatbatch D, Parker S, Watson P. Qualitative research methods in health technology assessment: a review of the literature. *Health Technol Assess* 1998;2:1-274.

32. Noyes J, Popay J. Directly observed therapy and tuberculosis: how can a systematic review of qualitative research contribute to improving services? A qualitative meta-synthesis. *J Adv Nurs* 2007;57:227-43.

33. Dixon-Woods M, Shaw RL, Agarwal S, Smith JA. The problem of appraising qualitative research. *Qual Saf Health Care* 2004;13:223-5.

34. Spencer L, Ritchie J, Lewis J, Dillon L. *Quality in qualitative evaluation: a framework for assessing research evidence [monograph online]*. London: Cabinet Office; 2003. Available from: www.gsr.gov.uk/evaluating_policy/era_papers/qual_eval.asp

35. Boulton M, Fitzpatrick R, Swinburn C. Qualitative research in health care: II. A structured review and evaluation of studies. *J Eval Clin Pract* 1996;2:171-9.

36. Campbell R, Pound P, Pope C, Britten N, Pill R, Morgan M, et al. Evaluating meta-ethnography: a synthesis of qualitative research on lay experiences of diabetes and diabetes care. *Soc Sci Med* 2003;56:671-84.

37. Hoddinott P, Pill R. A review of recently published qualitative research in general practice. More methodological questions than answers? *Fam Pract* 1997;14:313-9.

38. Long AF, Godfrey M. An evaluation tool to assess the quality of qualitative research studies. *Int J Soc Res Meth* 2004;7:181-96.

39. Popay J, Rogers A, Williams G. Rationale and standards for the systematic review of qualitative literature in health services research. *Qual Health Res* 1998;8:341-51.

40. Kuper A, Reeves S, Levinson W. An introduction to reading and appraising qualitative research. *BMJ* 2008;337:a288.

41. Dixon-Woods M, Sutton A, Shaw R, Miller T, Smith J, Young B, et al. Appraising qualitative research for inclusion in systematic reviews: a quantitative and qualitative comparison of three methods. *J Health Serv Res Policy* 2007;12:42-7.

42. Critical Appraisal Skills Programme (CASP). *Qualitative research: appraisal tool. 10 questions to help you make sense of qualitative research*. In. Oxford: Public Health Resource Unit; 2006. p. 1-4. Available from: www.phru.nhs.uk/Doc_Links/Qualitative Appraisal Tool.pdf

43. Thomas J, Harden A. Methods for the thematic synthesis of qualitative research in systematic reviews. *BMC Med Res Methodol* 2008;8:45.

44. Harden A, Brunton G, Fletcher A, Oakley A. *Young people, pregnancy and social exclusion: a systematic synthesis of research evidence to identify effective, appropriate and promising approaches for prevention and support*. London: EPPI-Centre, Social Science Research Unit, Institute of Education, University of London; 2006.

45. Rees R, Kavanagh J, Burchett H, Shepherd J, Brunton G, Harden A, et al. *HIV health promotion and men who have sex with men (MSM): a systematic review of research relevant to the development and implementation of effective and appropriate interventions*. London EPPI-Centre, Social Science Research Unit, Institute of Education, University of London; 2004.

46. Dixon-Woods M, Booth A, Sutton AJ. Synthesizing qualitative research: a review of published reports. *Qual Res* 2007;7:375-422.

47. Nicholas DB, Globerman J, Antle BJ, McNeill T, Lach LM. Processes of metastudy: a study of psychosocial adaptation to childhood chronic health conditions. *International Journal of Qualitative Methods* 2006;5:Article 5. Available from: www.ualberta.ca/~iiqm/backissues/5_1/pdf/nicholas.pdf

48. Walsh D, Downe S. Appraising the quality of qualitative research. *Midwifery* 2006;22:108-19.

49. Miller T, Bonas S, Dixon Woods M. Qualitative research on breastfeeding in the UK: a narrative review and methodological reflection. *Evidence & Policy* 2007;3:197-230.

50. Harden A. Critical appraisal and qualitative research: exploring sensitivity analysis [abstract]. In: *ESRC Research Methods Festival*; 2008 Jun 30-Jul 3; St Catherine's College, Oxford.

51. Noblit GW, Hare RD. *Meta-ethnography: synthesizing qualitative studies*. London: Sage; 1988.

52. Garside R. *A comparison of methods for the systematic review of qualitative research: two examples using meta-ethnography and meta-study* [PhD]. Exeter: Peninsula Postgraduate Health Institute, Universities of Exeter and Plymouth; 2008.

53. Petticrew M, Roberts H. *Systematic reviews in the social sciences: a practical guide*. Malden, MA: Blackwell Publishing; 2006.

54. Britten N, Campbell R, Pope C, Donovan J, Morgan M, Pill R. Using meta ethnography to synthesise qualitative research: a worked example. *J Health Serv Res Policy* 2002;7:209-15.

55. Pound P, Britten N, Morgan M, Yardley L, Pope C, Daker-White G, et al. Resisting medicines: a synthesis of qualitative studies of medicine taking. *Soc Sci Med* 2005;61:133-55.

56. Munro SA, Lewin SA, Smith HJ, Engel ME, Fretheim A, Volmink J. Patient adherence to tuberculosis treatment: a systematic review of qualitative research. *PLoS Med* 2007;4:e238.

57. Attree P. Growing up in disadvantage: a systematic review of the qualitative evidence. *Child Care Health Dev* 2004;30:679-89.

58. Graham H, McDermott E. Qualitative research and the evidence base of policy: insights from studies of teenage mothers in the UK. *J Soc Policy* 2005;35:21-37.

59. Thomas J, Harden A. *Methods for the thematic synthesis of qualitative research in systematic reviews*. London: ESRC National Centre for Research Methods; 2007. Report No.: NCRM Working Paper Series Number (10/07).

60. Thomas J, Sutcliffe K, Harden A, Oakley A, Oliver S, Rees R, et al. *Children and healthy eating: a systematic review of barriers and facilitators*. London: EPPI-Centre, Social Science Research Unit, Institute of Education, University of London; 2003.

61. Thomas J, Kavanagh J, Tucker H, Burchett H, Tripney J, Oakley A. *Accidental injury, risk-taking behaviour and the social circumstances in which young people live: a systematic review*. London: EPPI-Centre, Social Science Research Unit, Institute of Education, University of London; 2007.

62. Glaser BG, Strauss AL. *The discovery of grounded theory*. Chicago, IL: Aldine; 1967.

63. Dixon-Woods M, Agarwal S, Jones D, Young B, Sutton A. Synthesising qualitative and quantitative evidence: a review of possible methods. *J Health Serv Res Policy* 2005;10:45-53.

64. Strauss A, Corbin J. *Basics of qualitative research: techniques and procedures for developing grounded theory*. 2nd ed. Thousand Oaks, CA: Sage; 1998.

65. Kearney MH. Enduring love: a grounded formal theory of women's experience of domestic violence. *Res Nurs Health* 2001;24:270-82.

66. Barroso J, Powell-Cope GM. Metasynthesis of qualitative research on living with HIV infection. *Qual Health Res* 2000;10:340-53.

67. Hodson R. *Analyzing documentary accounts*. London: Sage; 1999.

68. Yin RK. *Case study research: design and methods*. 2nd ed. Thousand Oaks, CA: Sage; 1994.

69. Ragin CC. *The comparative method: moving beyond qualitative and quantitative strategies*. Berkeley, CA: University of California Press; 1987.

70. Popay J, Roberts H, Sowden A, Petticrew M, Arai L, Rodgers M, et al. *Guidance on the conduct of narrative synthesis in systematic reviews*. Lancaster: ESRC Research Methods Programme; 2006.

71. Thomas S, Fayter D, Misso K, Ogilvie D, Petticrew M, Sowden A, et al. Population tobacco control interventions and their effects on social inequalities in smoking: systematic review. *Tob Control* 2008;17:230-7.

72. Volmink J, Garner P. Directly observed therapy for treating tuberculosis *Cochrane Database of Systematic Reviews* 2007, Issue 4. Art. No.: CD003343. DOI: 10.1002/14651858.CD003343.pub3.

73. Thomas J, Harden A, Oakley A, Oliver S, Sutcliffe K, Rees R, et al. Integrating qualitative research with trials in systematic reviews. *BMJ* 2004;328:1010-2.

APPENDIX 1: OTHER REVIEW APPROACHES

This guide describes the process of conducting a systematic review of the effectiveness of a health intervention using aggregate data presented in reports and publications. Issues specific to undertaking reviews of clinical tests, adverse events, public health interventions, economic evaluations and the identification, assessment and synthesis of qualitative studies to help explain, interpret and implement the findings from effectiveness reviews are also covered. However, different types of evidence synthesis have emerged in order to meet objectives not met by standard systematic review methods. These approaches are beyond the scope of this guide but are described briefly here.

INDIVIDUAL PATIENT DATA (IPD) META-ANALYSIS

Individual patient data (IPD) meta-analysis is a specific method of systematic review. Instead of extracting data from study publications, the original research data for each participant is obtained directly from the researchers responsible for each included study. These data can then be collated and re-analysed centrally and, if appropriate, combined in meta-analyses. The approach has been used extensively in cancer[1] and cardiovascular disease and is becoming used more frequently in many other areas of health.[2]

Although IPD meta-analysis usually takes longer and costs more than conventional systematic reviews of published aggregate data, they offer a number of benefits, related particularly to the quality of data and the type of analyses that can be done.[3, 4] The IPD provided can be checked in detail and analyses carried out in a consistent way across studies. The reviewer does not have to interpret information or analyses presented in published reports, or have to consider combining differing outcome measures or summary statistics. If outcomes or patient characteristics have been measured, scored or defined differently, it is often possible to use the data supplied to redefine these according to an agreed review definition.[5] Problems with the original analyses can also often be avoided, for example if data are obtained from all randomised patients then intention to treat (ITT) analyses (see *Chapter 1 Section 1.3.5 Data synthesis*) may be possible even if the original study analyses failed to use ITT methods.[6] The IPD approach to systematic review can also be helpful in circumventing publication and associated biases.[7] IPD reviews should therefore be considered in circumstances where the published information does not permit a good quality review, or where particular types of analyses are required that are not feasible using standard approaches. Although obtaining IPD can help avoid problems associated with the analyses and reporting of studies, as with other types of systematic review, it cannot, generally, help avoid bias associated with study design or conduct.

IPD reviews are usually carried out as collaborative projects whereby all researchers contributing information from their studies, together with those managing or providing expert advice to the project, become part of an active collaboration. Detailed discussion

of how such projects are operationalised is outside the scope of this report. However, the rationale for IPD meta-analysis is the same as for any other systematic review, and many of the approaches and methods used are the same, with substantial differences occurring only with respect to data collection, checking and analysis. Some aspects of analysis, relating particularly to time-to-event analysis and sub-group analysis are discussed briefly in the synthesis section of *Chapter 1*. Their relevance to systematic reviews of clinical tests is briefly discussed in *Chapter 2*. Further details on organisation of IPD meta-analysis and data collection and checking can be found elsewhere.[3, 8]

PROSPECTIVE META-ANALYSIS

Prospective meta-analysis involves selecting a group of studies for inclusion in a meta-analysis before the results of those studies are known. Because decisions about relevant outcomes and subgroups are made in advance, there is no opportunity for selecting studies on the basis of their findings, thereby preventing publication and selection biases. It may also mean that investigators can agree on consistent study methods and data structures that will facilitate the subsequent meta-analysis. Analyses are generally done using IPD. However, as with IPD meta-analysis, prospective meta-analyses may require the close collaboration of several independent research groups, which can present various logistical challenges.

REVIEWS OF REVIEWS

This describes a systematic review that includes only other systematic reviews. In theory the systematic reviews included in the review should have covered most of the primary studies available. Reviews of reviews are likely to be helpful when a review question is very broad and a number of systematic reviews have already been conducted in the topic area. However, the different inclusion criteria adopted by the various reviews can make synthesis and interpretation problematic.

SCOPING REVIEWS

A scoping review determines the size and nature of the evidence base for a particular topic area, which can in turn be used to identify gaps in the literature and make recommendations for future primary research. The literature search should be as extensive as possible, including a range of relevant databases, handsearching and attempts to identify unpublished literature. Scoping reviews differ from standard systematic reviews in that they do not attempt to synthesise the evidence. A scoping review might be useful to research bodies that are planning a primary study, or to assess the feasibility of a full systematic review. It is not appropriate to use a scoping review to answer a clinical question.

RAPID EVIDENCE ASSESSMENTS

Rapid evidence assessments are used to summarise the available research evidence within the constraints of a given timetable, typically three months or less. Rapid evidence assessments differ from full systematic reviews in terms of the time constraints and consequently there are limitations on the extent of the literature searches and other review activities. Whilst attempting to be as comprehensive as possible, rapid evidence assessments usually make compromises to meet their tight deadlines; therefore they may fail to identify potentially relevant studies. They are useful to policy-makers who need to make decisions quickly, but should be viewed as provisional appraisals, rather than full systematic reviews.

REFERENCES

1. Clarke M, Godwin J. Systematic reviews using individual patient data: a map for the minefields? *Ann Oncol* 1998;9:827-33.

2. Simmonds MC, Higgins JP, Stewart LA, Tierney JF, Clarke MJ, Thompson SG. Meta-analysis of individual patient data from randomized trials: a review of methods used in practice. *Clin Trials* 2005;2:209-17.

3. Stewart LA, Clarke MJ. Practical methodology of meta-analyses (overviews) using updated individual patient data. Cochrane Working Group. *Stat Med* 1995;14:2057-79.

4. Stewart LA, Tierney JF. To IPD or not to IPD? Advantages and disadvantages of systematic reviews using individual patient data. *Eval Health Prof* 2002;25:76-97.

5. Askie L, Duley L, Henderson-Smart D, Stewart L. Antiplatelet agents for prevention of pre-eclampsia: a meta-analysis of individual patient data. *Lancet* 2007;369:1791-8.

6. Advanced Bladder Cancer (ABC) Meta-analysis Collaboration. Adjuvant chemotherapy in invasive bladder cancer: a systematic review and meta-analysis of individual patient data Advanced Bladder Cancer (ABC) Meta-analysis Collaboration. *Eur Urol* 2005;48:189-99.

7. Stewart L, Tierney J, Burdett S. Do systematic reviews based on individual patient data offer a means of circumventing biases associated with trial publications? In: Rothstein HR, Sutton AJ, Borenstein M, editors. *Publication bias in meta-analysis: prevention, assessment and adjustments*. Chichester: Wiley; 2005. p. 261-86.

8. Stewart LA, Tierney JF, Clarke M. Chapter 19: Reviews of individual patient data. In: Higgins JPT, Green S, editors. *Cochrane Handbook for Systematic Reviews of Interventions. Version 5.0.0 (updated February 2008)*: The Cochrane Collaboration; 2008. Available from: www.cochrane-handbook.org

APPENDIX 2: EXAMPLE SEARCH STRATEGY TO IDENTIFY STUDIES FROM ELECTRONIC DATABASES

The development of a search strategy is an iterative process: one attempt will rarely produce the final strategy. Strategies are usually built up from a series of test searches and discussions of the results of those searches among the review team.

The first step is to break down the review question to help guide the development of search terms, using a structure such as PICOS.

For example:

Population(s)/Patient(s)	Patients undergoing hip replacement
Intervention(s)/Treatment(s)	Antimicrobial prophylaxis
Comparator(s)	No prophylaxis
Outcome(s)	Postoperative infection
Study Design	Any type of study design

It is not necessary to include all of the PICOS concepts in the search strategy. It is preferable to search for those concepts that can be clearly defined and translated into search terms. Concepts that are poorly defined, not likely to be included in journal abstracts, or not indexed in a consistent way will be difficult to identify from database searches. If this is the case, using a broader search and then sifting through the identified studies may be preferable. This may apply particularly to the outcome(s) of studies as these are frequently not referred to in either the title or abstract of a database record.

Search filters are tested and in some cases validated strategies that can be used in a named database to identify specific types of study. They usually consist of a series of database index terms relating to study type combined with free text terms describing the methods used in conducting that type of research. There are filters available that will, for example, reliably identify RCTs in MEDLINE and in EMBASE, but filters for use in other databases or to identify other study types are limited. The development and validation of filters to identify other study types, such as diagnostic accuracy studies and qualitative research, is ongoing.[1-4] A useful source of information about search filters is the website maintained by the InterTASC Information Specialists' Subgroup www.york.ac.uk/inst/crd/intertasc/ which lists both published search filters and research on their development and use.

Once the concepts of the search have been determined, the next stage is to produce a list of synonyms, abbreviations and spelling variants which may be used by authors. Similar research is often described using very different terms. To reflect this variation, a search strategy will usually comprise both indexing terms (if the database has a thesaurus or controlled vocabulary) and 'free text' terms and synonyms (from the database record's title and abstract) to ensure that as many relevant papers

are retrieved as possible. For example, when searching MEDLINE for studies about myocardial infarction, the free text term “heart attack” should be used as well as the Medical Subject Heading (MeSH) term “Myocardial Infarction”. Identifying appropriate indexing terms can be done by searching for key papers and checking how they have been indexed, consulting clinical experts in the review team and advisory group, as well as by scanning the thesaurus for relevant terms.

When selecting free text terms to use in the strategy it is important to take account of alternative spellings (including US and British English variants), abbreviations, synonyms, geographical variation, and changes in terminology over time. Sometimes it can also be useful to search for common mis-spellings, for example “asprin” when you want to retrieve studies of aspirin.

It is important to compile imaginatively and to check the indexing terms used in known relevant publications. Once a list of potential search terms has been compiled for each of the concepts, the next stage is to identify relevant subject headings which have been used to describe the topic in the databases you plan to search. For example with postoperative infection the following Medical Subject Headings (MeSH) are available for use in MEDLINE:

- Bacterial Infections
- Postoperative Complications
- Surgical Wound Infection
- Prosthesis-Related Infections
- Sepsis
- Infection Control.

Some of these terms are “high level” that encompass narrower or more specific terms. To capture these narrower terms, in those databases that offer the facility, it is possible to ‘explode’ the high level term and so search for many terms at once. The explosion facility within a database makes use of the hierarchical thesaurus. Using the command “exp Bacterial Infections/” in the OvidSP interface to MEDLINE will retrieve papers indexed with that term but will also automatically retrieve papers indexed with the narrower terms Bacteramia, Hemorrhagic Septicaemia, Central Nervous System Bacterial Infections, etc. as displayed in the section of the MeSH below.

Bacterial Infections/

- Bacteraemia
    - Hemorrhagic Septicaemia
- Central Nervous System Bacterial Infections
- Endocarditis, Bacterial
- Eye Infections, Bacterial
- Fournier Gangrene

The subject headings should be added to the concept list relating to the postoperative infection concept so that a first test search strategy for MEDLINE includes a mixture of text terms and MeSH headings.

bacterial adj infect$.ti,ab.
(postoperative adj complication$ or post adj operative adj complication$).ti,ab.
surgical adj wound adj infection$.ti,ab.
prosthesis-related adj infection$.ti,ab.
hip adj replacement adj3 infection$.ti,ab.
sepsis.ti,ab.
Septic?emia.ti,ab.
infection adj control.ti,ab.
bacterial adj contamination.ti,ab.
Exp Bacterial Infections/
Exp Postoperative Complications/
Surgical Wound Infection/
Prosthesis-Related Infections/
Sepsis/
Exp Infection Control/

The search has been written for the OvidSP search interface to MEDLINE and has commands specific to that interface:

adj	Words have to appear next to each other. Also retrieves hyphenated words.
adj3	Words have to appear within 3 words of each other. Other numbers can be used as required.
$	Truncation symbol, for example ‘complication$’ retrieves ‘complications’ as well as ‘complication’.
.ti,ab	Restricts the search to title and abstract fields, to avoid retrieving unexpected results from the subject headings.
EXP	Explode the subject heading, to retrieve more specific terms
/	MeSH heading.
?	O*ptional wild card* character used within, or at the end of, a search term to substitute for one or no characters. Useful for retrieving documents with British and American word variants.

Each database interface has its own unique set of commands and, information about these will be on the database help pages.

Once a series of concepts that reflect the PICOS elements have been compiled they are then combined using Boolean logic (AND, OR, NOT) to create a set of results which should contain articles relating to the topic in question. The AND operator is used to ensure that all the search terms must appear in the record, for example searching for “prostate AND cancer” retrieves all records which contain both the term prostate and the term cancer. AND is used to narrow down or focus a search.

OR is used to accumulate similar search terms and thus makes searches larger. Searching for "heparin OR warfarin" retrieves all records where either heparin or warfarin or both are found. It is best to use the OR operator to combine terms relating to the same concept (e.g. all the postoperative infection terms in the example above) before narrowing down a search using the AND operator with another set of terms.

NOT is used to exclude records from a search. For example, "acupuncture NOT asthma" will retrieve all records which contain the term acupuncture, but not those which also contain the word asthma. NOT should be used with great care because it may have a larger effect than anticipated; a record may well discuss both the concept of interest and the one to be excluded.

The combination of concepts using the Boolean operators might develop as follows (for MEDLINE using the OVIDSP interface):

1 Hip Joint/
2 Hip Prosthesis/
3 Acetabulum/
4 hip replacement$.ti,ab.
5 total-hip replacement$.ti,ab.
6 total joint replacement$.ti,ab.
7 hip surgery.ti,ab.
8 hip operation$.ti,ab.
9 (hip adj3 prosthe$).ti,ab.
10 (hip adj3 arthroplasty).ti,ab.
11 1 or 2 or 3 or 4 or 5 or 6 or 7 or 8 or 9 or 10
12 exp Bacterial Infections/
13 exp Postoperative Complications/
14 Surgical Wound Infection/
14 Prosthesis-Related Infections/
16 Sepsis/
17 exp Anti-Infective Agents/
18 exp Infection Control/
19 exp Antibiotics/
20 Antibiotic Prophylaxis/
21 ((bacteri$ or wound$) adj2 (infect$ or contamin$)).ti,ab.
22 sepsis.ti,ab.
23 antibiotic$.ti,ab.
24 antimicrobial$.ti,ab.
25 anti-microbial$.ti,ab.
26 (anti$ adj infect$).ti,ab.
27 ultraclean.ti,ab.

28 hypersterile.ti,ab.
29 or/12-28
30 11 and 29

Sets 1 to 10 capture the concepts of hip replacement or hip surgery and are combined using OR to produce result set 11. Sets 12 to 28 capture the concepts of infection and infection prevention and are combined using OR to produce result set 29. The two sets of concepts are then combined to find the records which contain both concepts using AND to produce set 30.

The draft strategy can be tested on one database and the results checked by whether it retrieves papers that are already known to the team but were not used to develop the draft strategy. In addition, a small sample of the results of the test or scoping search can be examined by the review team to identify additional search terms (text words and indexing) or highlight potential limitations. The sample records need to be representative so bear in mind that the search results as output from the database will be listed in either alphabetical order by authors name, or by publication date or by date added to the database. Depending upon the complexity of the review topic, and consequently the search to be undertaken, this process may need to be repeated several times until an agreed strategy is formulated. If at all possible, the final search strategy should be peer reviewed to check for errors (spelling mistakes, incorrect use of operators, or failure to include relevant MeSH) that could reduce the recall of papers.[5]

At this point, the searches using other databases and resources can begin. However, this does not mean that search iterations should necessarily stop. If new search terms are identified during the review process they should be incorporated into the strategy or supplementary searches should be carried out.

Converting a final strategy for use in other databases requires care. While free text terms can usually be re-used in other databases you will need to identify one or possibly more matching relevant thesaurus terms used by the other databases. Each database thesaurus is unique so this procedure should be undertaken for each database being searched. For example, if you are searching MEDLINE for papers about "pressure sores" you would use the MeSH term "pressure ulcer" while if you were searching EMBASE you would need to use the EMTREE term "decubitus".

If the search interface is also different you will need to make appropriate changes to the search operators used in the strategy. For example, some database providers use '$' as the truncation symbol, while other database providers use '*'.

Not all databases include an abstract in the record. Where this is the case the search strategy can be made more sensitive given the reliance solely upon terms being identified in the title (and any indexing fields). This can be achieved by using more synonyms and broader terms.

In some cases databases with web interfaces have a restricted range of search options and if this is the case searchers need to adopt pragmatic approaches and use very simple searches. For example, if there are limited options for combining terms using Boolean operators such as AND an alternative approach may be to run a number of separate searches on the database in place of one longer search.

REFERENCES

1. Leeflang M, McDonald S, Scholten RJ, Rutjes A, Reitsma JJB. Search strategies to identify diagnostic accuracy studies in MEDLINE and EMBASE (Protocol). *Cochrane Database of Systematic Reviews* 2007, Issue 2. Art. No.: MR000022. DOI: 10.1002/14651858.MR000022.pub2.

2. Flemming K, Briggs M. Electronic searching to locate qualitative research: evaluation of three strategies. *J Adv Nurs* 2007;57:95–100.

3. Leeflang MMG, Scholten RJPM, Rutjes AWS, Reitsma JB, Bossuyt PMM. Use of methodological search filters to identify diagnostic accuracy studies can lead to the omission of relevant studies. *J Clin Epidemiol* 2006;59:234-40.

4. Shaw RL, Booth A, Sutton AJ, Miller T, Smith JA, Young B, et al. Finding qualitative research: an evaluation of search strategies. *BMC Med Res Methodol* 2004;4:5.

5. Sampson M, McGowan J. Errors in search strategies were identified by type and frequency. *J Clin Epidemiol* 2006;59:1057-63.

APPENDIX 3: DOCUMENTING THE SEARCH PROCESS

The search should be described briefly in the methods section of the review. The detailed description can be made available as a web document or as an appendix in a report, where space allows.

DESCRIBING ELECTRONIC DATABASE SEARCHES

The description should include:

- The name of the database searched
- The name of the database provider/system used
- The date when the search was run
- The years covered by the search
- The full strategy used. It is preferable to reproduce the strategy with the minimum of editing. Removing the number of hits identified, for example, can introduce errors and hide genuine mistakes.

For example:

MEDLINE was searched using the Ovid interface on 06/11/07 for the period 1996 to October Week 4 2007

1 (smoking or antismoking or anti-smoking).ti,ab. (53347)
2 Smoking/(39157)
3 (smoker or smokers or tobacco or nicotine or cigar$).ti,ab. (53978)
4 Tobacco/or Tobacco, Smokeless/(9039)
5 "Tobacco Use Disorder"/(3192)
6 Nicotine/(6990)
7 or/1-6 (91596)
8 exp Mass Media/or Cellular Phone/(13165)
9 Electronic Mail/or Radio/or Television/or Telephone/(6855)
10 Advertising/or Hotlines/or Nonverbal Communication/(6927)
11 Multimedia/or Communications Media/or Pamphlets/(2296)
12 Health Education/or Internet/or Health Promotion/(55884)
13 Telecommunications/(1517)
14 (mass adj media).ti,ab. (949)
15 ((advert$ or campaign$ or program$) adj3 (tv or television or cable or cinema or cinemas or theatre or theatres or theater or theaters or movies or media or newspaper$ or journal$ or magazine$)).ti,ab. (1466)

16 ((advert$ or campaign$ or program$) adj3 (broadcast$ or televised)).ti,ab. (67)
17 (internet adj3 (advert$ or campaign$ or information or program$)).ti,ab. (1349)
18 (sms or text messag$ or texting).ti,ab. (754)
19 ((pod adj cast$) or podcast$).ti,ab. (28)
20 (smoking adj day$).ti,ab. (22)
21 (selfhelp or (self adj help) or (counter adj marketing) or (consumer adj advocacy)).ti,ab. (1495)
22 ((quit adj3 win) or smokeout or (smoke adj out)).ti,ab. (54)
23 ((advert$ or campaign$ or program$ or intervention$) adj3 (nationwide or statewide or countrywide or citywide or national or nation wide or state wide or country wide or city wide)).ti,ab. (7031)
24 or/8-23 (88367)
25 7 and 24 (5364)

DESCRIBING JOURNAL HANDSEARCHES

Provide a list of journal full titles in alphabetical order. State the earliest month and year searched, together with the latest month and year searched, and any missing journal issues which were not searched.

DESCRIBING SEARCHES OF CONFERENCE PROCEEDINGS

Provide details of the conference proceedings searched, for example:

Proceedings with a title in addition to the conference name:

> Substance use: individual behaviour, social interactions, markets, and politics. 24th Arne Ryde Symposium; 2004, Aug 13-14; Lund.

Proceedings without a separate title:

> International Meeting on the Economic, Social, and Health Issues in Tobacco Control; 2001 Dec 3-4; Kobe City

Proceedings in a language other than English:

> Arrêt de la consommation du tabac: Conférence de consensus [Consensus conference on smoking cessation]; 1998 Oct 8-9; Charcot.

Proceedings also published as part of a journal:

> Proceedings of the 6th Scientific Conference: Tobacco or health, eternal problem, new challenges; 2005 Nov 17-18; Poznan. (Przeglad Lekarski. 2005;62(10):947-1226)

DESCRIBING THE METHODS USED TO SEARCH RELEVANT INTERNET SOURCES

Report the website, the URL, the date searched, any specific sections searched and the search terms used. For example:

> The Action for Smoking and Health website (www.ash.org.uk/) was searched on 12/10/2006 using the on-site search engine with single search terms: 'mass media', 'radio', 'television', 'film', 'advertising'. The section of the website labelled 'Tobacco Industry Documents' was scanned in detail.

DESCRIBING OTHER SEARCHES

Provide a brief summary of other sources searched, for example:

> The reference lists of studies selected for inclusion were scanned for relevant studies.
>
> The manufacturing company United Tobacco Substitutes International was contacted for further information (15/11/06).
>
> Citation searches in Science Citation Index (15/11/06) were carried out for papers citing the recent key paper: Smith P and Jones L. Innovative mass media smoking campaigns. Journal of Tobacco Control Innovation, 2004;23:560-569.

DESCRIBING THE SEARCH PROCESS

The following example shows how a search can be described briefly in a final report when it is possible to put the full searches in an appendix.

> The following databases were searched for relevant studies: ASSIA (via CSA 1990 to 06/March/06); CENTRAL (via Cochrane Library issue 2/2006, 12/April/06, 12/April/06); CINAHL (via WebSPIRS 1990 to 2006 wk 4, 01/April/06); DH-Data (via Datastar 1994 to 14/March/06, 14/March/06); EMBASE (via Dialog 1974 to 2006 week 8, 16/March/06); MEDLINE (via Ovid, 1966 to week 10 2006, 15/March/06); PsycINFO (via Datastar 1806 to 2006 week 2, 17/March/06). The search strategies used text words and relevant indexing to capture the concept of preventing the uptake of smoking in young people. The full strategies are shown in the appendix. The reference lists of included papers were assessed for additional relevant studies and the journal *Tobacco Control* was handsearched for the period January 2000-June 2006. The following websites were searched: National Guideline Clearinghouse (www.guideline.gov/15/Oct/06) and Action on Smoking and Health (ASH) (www.ash.org.uk/16/Oct/06).

The following example shows how a search can be described briefly for a journal article where high levels of detail may not be permitted in the body of the paper:

> The following databases were searched from inception to March/April 2006 for relevant studies: ASSIA, CENTRAL, CINAHL, DH-Data, EMBASE, MEDLINE, PsycINFO. The literature search used the following terms (with synonyms and

closely related words): "smoking" combined with "prevention" and "young people". The searches were not limited by study design or language of publication. Further studies were identified by examining the reference lists of all included articles, handsearching the journal *Tobacco Control* from 2000 to 2006, and searching relevant websites. The full list of sources and the search strategy are available from the authors.

A detailed search description included as an appendix to a report, or as a web document, or available from the authors should include the detail described above, plus all the search strategies for each database and resource, as shown in Q.1 to Q.5 above.

The following are examples of the use of appendices for search strategies in CRD Reports:

1. Prostate biopsy methods is an example of a narrow, well-defined topic where brief details of the search strategy are given in the report methods section (pages 23-4), then a clear, well set out and very detailed appendix itemises exactly what was done (pages 87-97).

   CRD Report 29 – Diagnostic value of systematic prostate biopsy methods in the investigation for prostate cancer: a systematic review. 2005. www.york.ac.uk/inst/crd/pdf/report29.pdf

2. Fuller information about the searches is given in the methods section of this report (pages 4-5) with additional detail in the appendix (pages 25-30).

   CRD Report 32 – Systematic review of interventions to increase participation of cancer patients in randomised controlled trials. 2006. www.york.ac.uk/inst/crd/pdf/report32.pdf

3. This is an example of how an updated search looking at a broad subject area has been presented. Brief details are given in the review methods section (page 1) and readers are referred to the full details in the appendix (pages 41-50).

   CRD Report 35 – The treatment and management of chronic fatigue syndrome (CFS)/myalgic encephalomyelitis (ME) in adults and children: update of CRD Report 22. 2007. www.york.ac.uk/inst/crd/pdf/report35.pdf

Please note that in the three examples above the search strategies have been reported without including the numbers of records identified by individual search statements. As stated earlier in this section, CRD would now recommend the strategies being reproduced with a minimum of editing so there is less opportunity for errors to be introduced.

APPENDIX 4: SEARCHING FOR ADVERSE EFFECTS

Named adverse effects can be searched for as both indexing terms and text words. For example in MEDLINE OvidSP sudden death can be searched using the following approach;

#1 exp Death, Sudden/

#2 (sudden adj2 death$).ti,ab.

#3 #1 or #2

KEY

exp denotes exploding the succeeding indexing term

adj is the proximity operator in OvidSP, and adj2 denotes within 2 words

$ is the truncation symbol in OvidSP

ti, ab denotes searching in the title and abstract

In some instances subheadings may be available to focus the search on the outcome as an adverse effect (rather than, for example, a consequence of the disease). For example in MEDLINE the subheading 'chemically induced' is available and a search for vision disorders as an adverse effect may be carried out as follows:

#1 exp Vision Disorders/ci [chemically induced]

It should be noted, however, that for truly sensitive searches suitable text words will need to be added (for example, vision disorder$.ti,ab), this could be in combination with adverse effects terms (for example, vision disorder$.ti,ab and complication$.ti,ab etc).

In addition to terms for named adverse effects it may also be appropriate (due to poor reporting in papers and indexing in databases) to add generic adverse effect search terms as for a general search for all adverse effects associated with an intervention (see below).

SEARCHING WITH GENERIC ADVERSE EFFECTS TERMS

If none of the adverse effects are known at the time of searching then a sensitive search strategy in MEDLINE OvidSP would include a combination of MeSH floating subheadings and text words for generic adverse effects terms for example;

#1 ae.fs. OR co.fs. OR de.fs.

#2 (safe OR safety OR side effect$ OR undesirable effect$ OR treatment emergent OR tolerability OR toxicity OR adrs OR (adverse adj2 (effect or effects or reaction or reactions or event or events or outcome or outcomes))).ti,ab

#1 OR #2

KEY

ae denotes the subheading 'adverse effects'

co denotes the subheading 'complications'

de denotes the subheading 'drug effects'

.fs denotes 'floating' the preceding subheading (ie searching for the subheading attached to any indexing term).

Many papers on adverse effects will not contain any generic adverse effects terms in the title, abstract or indexing. In instances where the adverse effects are unknown at the time of searching, it may be possible to identify potential adverse effects to use as search terms from tertiary sources such as the BNF and Meyler's Side Effects of Drugs.

Abbreviations and acronyms

AcroMine	Text mining tool dealing with term extraction and variation
AgeInfo	Bibliographic database (specific population group)
Ageline	Bibliographic database (specific population group)
Agricola	Bibliographic database (agriculture)
AHRQ	Agency for Healthcare Research and Quality
AMED	Bibliographic database (complementary medicine)
ASSIA	Bibliographic database (Applied Social Sciences Index & Abstracts)
AUC	Area Under the Curve
BIOSIS Previews	Bibliographic database (life sciences)
CASP	Critical Appraisal Skills Programme
CBA	Cost-benefit Analysis
CCA	Cost-consequences Analysis
CCTRs	Comprehensive Clinical Trials Reports
CDSR	Cochrane Database of Systematic Reviews
CEA	Cost-effectiveness Analysis
CENTRAL	Cochrane Central Register of Controlled Trials
ChildData	Bibliographic database (specific population group)
CI	Confidence Interval
CINAHL	Bibliographic database (nursing and allied health)
CONSORT	A set of recommendations for the reporting of RCTs
CRD	Centre for Reviews and Dissemination
CSA	Bibliographic database (sociological & social services abstracts)
CUA	Cost-utility Analysis
DAPS	Drug Analysis Prints
DARE	Database of Abstracts of Reviews of Effects
DE	Design Effect
DFS	Disease free survival
DH	Department of Health

Dialog	Database provider
DoPHER	Database of Promoting Health Effectiveness Reviews
DOR	Diagnostic Odds Ratio
DOT	Directly Observed Therapy
DSA	Digital Subtraction Angiography
DUETs	Database of Uncertainties about the Effects of Treatments
EE	Effect Estimates
EI Compendex	Bibliographic database (engineering)
EMBASE	Bibliographic database (biomedicine)
EMTREE	Thesaurus used in EMBASE database
Enviroline	Bibliographic database (environment)
EPAR	European Public Assessment Report
EPICOT	Evidence, Population(s), Intervention(s), Comparison(s), Outcome(s), Time stamp
EPOC	Cochrane Effective Practice and Organisation of Care Group
EPPI	Evidence for Policy and Practice Information
EQUATOR	Enhancing the QUAlity and Transparency Of health Research
EQ-5D	An instrument for measuring utility in economic evaluations
ERIC	Bibliographic database (education)
ESRC	Economic and Social Research Council
FACTA	Text mining tool that finds associated concepts using text analysis
FDA	Federal Drug Administration
FN	False Negative
FP	False Positive
GEOBASE	Bibliographic database (earth sciences and human geography)
HCA	Human Capital Approach
HEED	Health Economic Evaluations Database
HMIC	Health Management Information Consortium. A bibliographic database
HR	Hazard Ratio
HSROC	Hierachical SROC
HTA	Health Technology Assessment
HYE	Healthy-years-equivalent
ICC	Intracluster Correlation Coefficient

ICER	Incremental Cost-effectiveness Ratios
ICMJE	International Committee of Medical Journal Editors
ICONDA	Bibliographic database (housing and architecture)
IDIS	Iowa Drug Information Service
InterTASC	Inter Technology Assessment Services Collaboration
IPA	International Pharmaceutical Abstracts. A bibliographic database
IPD	Individual Patient Data
ISSG	Information Specialists' Sub-Group of InterTASC
ITT	Intention To Treat
KLEIO	Text mining tool that provides advanced searching facilities across Medline
LILACS	Bibliographic database (Latin American and Caribbean Health Sciences Literature)
LMWH	Low-molecular-weight heparin
MANTIS	Bibliographic database (osteopathy and chiropractic)
MCUG	Micturating cystourethrography
MD	Mean Difference
MEDLINE	Bibliographic database (medicine)
MeSH	Medical Subject Headings used in MEDLINE database
MMR	Measles Mumps and Rubella
MRA	Magnetic Resonance Angiography
MTC	Mixed Treatment Comparisons
NGC	National Guidelines Clearinghouse
NHS	National Health Service
NHS EED	NHS Economic Evaluation Database
NICE	National Institute for Health and Clinical Excellence
NIHR	National Institute for Health Research
NRSMG	Cochrane Non Randomised Studies Methods Group
NSAIDs	Non-Steroidal Anti-Inflammatory Drugs
NTIS	National Technical Information Service
NTT	Number Needed To Treat
O – E	Observed–Expected
OR	Odds Ratio
PAIS	Public Affairs Information Service. A bibliographic database

PDR	Physician's Desk Reference
PHL	Public Health Language
PHO	Public Health Observatories
PICOCS	Population, Interventions, Comparators, Outcomes, Context, Study design
PICOS	Population, Interventions, Comparators, Outcomes and Study Designs
PRISMA	Preferred Reporting Items for Systematic Reviews and Meta-Analyses
PROGRESS	Place of Residence, Race/ethnicity, Occupation, Gender, Religion, Education, Socioeconomic status and Social capital.
PSA	Probabilistic Sensitivity Analysis
PsycINFO	Bibliographic database (psychology and psychiatry)
QALYs	Quality-Adjusted-Life-Years
QUADAS	Quality Assessment of Diagnostic Accuracy Studies included in Systematic Reviews
QUOROM	Quality of Reporting of Meta-analyses
RCT	Randomised Controlled Trial
REMARK	REporting recommendations for tumour MARKer prognostic studies
ROC	Receiver Operating Characteristic
RR	Risk Ratios
RRR	Relative Risk Reduction
RSS	Really Simple Syndication
SAS	Data analysis and statistical software
SD	Standard Deviation
SE	Standard Error
SF-6D	An instrument for measuring utility in economic evaluations
SIGN	Scottish Intercollegiate Guidelines Network
SMD	Standardised Mean Difference
SportDiscus	Bibliographic database (sport, health, fitness & sports medicine)
SROC	Summary ROC
STARD	Standards For The Reporting Of Diagnostic Accuracy Studies
STATA	Data analysis and statistical software
STROBE	An initiative to improve reporting of observational studies
TB	Tuberculosis

TerMine	Text mining tool dealing with term extraction and variation
TN	True Negative
TP	True Positive
TRANSPORT	Bibliographic database (transport)
TREND	Guidelines for the reporting of nonrandomized evaluations of behavioural and public health interventions
SRS	Systematic review software
TSH	Thyroid Stimulating Hormone
UTI	Urinary Tract Infection
VUR	Vesicoureteric Reflux
Wolters Kluwer	Database provider
Waternet	Bibliographic database (water supply)
WHO	World Health Organisation
WHO-ART	WHO Adverse Reaction Terminology
WINBUGS	Data analysis and statistical software
WTP	Willingness To Pay
ZETOC	Database of journal contents pages provided by the British Library and the Mimas servic

Glossary

Term	Definition
Absolute risk reduction	See Risk difference.
Ad hoc	A solution designed for a specific problem or task that is not generalisable to other situations (Latin).
Adverse effect	An adverse event for which the causal relation between the drug/intervention and the event is at least a reasonable possibility.
Adverse event	An adverse outcome that occurs during or after exposure to a drug or other intervention and which may or may not be caused by the intervention.
Allocation bias	Bias resulting from a systematic difference (other than the intervention) between experimental and control groups in a clinical trial. Allocation bias can be avoided by randomisation.
Allocation concealment	See Concealment of allocation.
A priori	Formed or conceived beforehand (Latin).
Archive	Collection of material made at the end of a project and preserved to assist in answering queries about the review and to facilitate any update.
Area under the curve (AUC)	A graphical summary of overall diagnostic accuracy; the closer the curve is to the upper left hand corner of the graph, the better the diagnostic performance. The AUC ranges from 0 to 1, with 0.5 indicating a poor test where the accuracy is equivalent to chance.
Attrition bias	Bias resulting from systematic differences between comparison groups as a result of differential withdrawals or exclusions of participants.
Bayesian analysis	An approach to statistical analysis that can be used in single studies or meta-analysis. A prior probability distribution based on objective evidence and subjective opinion is defined at the outset. Bayes' theorem is then used to update the prior distribution in light of the results of a study, producing a posterior distribution from which point estimates of effect and credible intervals (equivalent to confidence intervals) can be defined.
Baseline characteristics	Participant characteristics that are collected at the beginning of a study prior to receiving the intervention. Characteristics may include demographic details such as age and gender and clinical characteristics such as stage of disease or presence of co-morbidites.

Before-and-after study	A study design where a group is studied before and after an intervention.
Bias	A systematic error or deviation in results or inferences from the underlying 'truth'. See also selection bias; performance bias; attrition bias; detection bias and reporting bias.
Bibliographic databases	Databases that provide descriptive records of items such as books and articles.
Bibliographic software	Computer software that assists with the organisation of bibliographic references. There are many different packages (e.g. EndNote, Reference Manager), but most will allow for the import of references from bibliographic databases and the automated production of reference lists.
Blinding	Keeping knowledge of which comparison group a participant belongs (e.g. to intervention or control) from the study participants, investigators or outcome assessors. This reduces the risk of bias.
Boolean operator	Boolean operators are used to combine terms when conducting electronic searches. Examples include "AND" (used to narrow a search), "OR" (used to broaden a search) and "NOT" (used to exclude terms from a search).
Bootstrapping	A statistical approach for examining the uncertainty in cost-effectiveness analysis. It involves drawing many random sub-samples from the original data set and computing the statistic of interest from each in the same way. After sampling, each sub-sample is returned to the data set, a process known as sampling with replacement.
Case-control study	An observational study that compares people with a specific disease or outcome of interest (cases) with a suitable control group of people without that disease or outcome, and which seeks to find associations between the outcome and prior exposure to particular risk factors.
Case series	A study reporting observations on a series of individuals, usually all receiving the same intervention, with no control group.
Case survey	Formal process for coding data from qualitative cases into a quantitative form for statistical analysis.
Clinical heterogeneity	See Heterogeneity.
Cluster randomised trial	A trial where randomisation is of clusters of people (e.g. general practices, schools) rather than individuals themselves.
Cochrane Collaboration	An international organisation that aims to help people make well-informed decisions about health care by preparing, maintaining, updating and ensuring the accessibility of systematic reviews of the effects of healthcare interventions.
Cohort study	An observational study in which a defined group of participants is observed over time and a comparison made between those who did and those who did not receive the intervention.

Co-intervention	An additional diagnostic or therapeutic procedure given to people receiving a particular intervention.
Commissioning brief	Information provided by an organisation wishing to commission a systematic review to assist researchers in preparing proposals to undertake the work.
Communication channel	Any medium used to convey a message to an audience or audiences.
Co-morbidity	The presence of one or more diseases or conditions other than those of primary interest.
Comparator	In a controlled trial, the intervention (which could include placebo, usual care, another intervention or no treatment) with which the intervention of interest is compared.
Complex intervention	An intervention involving a number of separate elements that seem essential to the proper functioning of the intervention although the active ingredient of the intervention that is effective is difficult to specify.
Concealment of allocation	The process used to prevent foreknowledge of which comparison group an individual will be assigned to in a randomised controlled trial. Inadequate concealment of allocation may lead to selection bias.
Conceptual mapping	In narrative synthesis, the use of visual methods to help to construct groupings of, and relationships between, ideas and/or concepts. Closely related to idea webbing.
Conceptual triangulation	In narrative synthesis, the use of a combination of different perspectives and/or methods to study a particular concept.
Confidence interval	A measure of uncertainty around the results of a statistical analysis that describes the range of values within which we can be reasonably sure that the true effect lies.<br>For example a 95% confidence interval is based on the notion that if a study were repeated many times in other samples from the same population, 95% of the confidence intervals from those studies would include the true value of the effect being measured.<br>Wider intervals indicate lower precision; narrow intervals, greater precision.
Confounding	A situation in which a measure of the effect is distorted because of an association between the intervention (or exposure) with other factor(s) that influence the outcome under investigation. For example, if the control group includes patients with more advanced stages of cancer than in the intervention group, then an analysis of survival will be confounded by tumour stage.
Content analysis	A set of procedures for collecting and organizing non-structured information. This approach makes it easier to systematically and objectively analyze the data and make inferences about the population of interest.

Continuous outcomes	Outcomes related to variables with a potentially infinite number of possible values within a given range, for example weight and blood pressure.
Contributorship	A system of publication credit in which all those who contributed to a publication are listed with details of their contribution, including those who did not meet the standard criteria to be listed as authors.
Control group	The group that acts as a comparator for one or more experimental interventions in a controlled trial.<br>The group without the disease or outcome of interest in a case control study.
Controlled trial	A clinical trial with a control group.
Cost-effectiveness analysis	An economic analysis that converts effects into health terms and describes the costs for some additional health gain (e.g. cost per additional stroke prevented).
Cost-to-charge ratios	In economic analyses, an adjustment applied to charges in order to better reflect the true costs of the technology being evaluated.
Critical interpretive synthesis	A form of review which, while sensitive to the issues involved in conducting reviews that conventional systematic review methodology has identified, draws on a distinctive tradition of qualitative inquiry, including interpretive approaches to review, enabling the generation of theory with strong explanatory power.
Cross-over trial	A type of clinical trial comparing two or more interventions in which all the participants receive all the interventions but the order of receipt is determined by randomisation.
Cross-sectional study	A study that examines the relationship between diseases (or other health related characteristics) and other variables of interest as they exist in a defined population at a particular time.
Custom field	In bibliographic software, a field for which the type of content is not pre-specified by the software and which can therefore be customised by the individual. Often used in systematic reviews for keeping track of decisions or articles ordered.
Cutpoint	The threshold or value at which continuous data are divided into dichotomous categories. If used it is important that the cutpoint is not determined by a data dependent process.
Database provider	The specific platform through which a database is accessed. Examples include OVID and Dialog. Many databases are available via more than one provider. See also Search interface.
Decision modelling	A theoretical construct (often using a mathematical framework) that allows the comparison of the relationship between costs and outcomes of alternative health care interventions by incorporating evidence from a variety of sources.

Detection bias	Bias caused by systematic differences between comparison groups in how outcomes are ascertained, diagnosed or verified.
Diagnostic odds ratio	An overall indicator of diagnostic performance, calculated as the odds of a positive test result among those with the target condition, divided by the odds of a positive test result among those without the condition.
Diagnostic threshold	Point at which diagnostic test results are classified as positive or negative.
Dichotomous data	Data that can take one of two possible values for example dead or alive, myocardial infarction or no myocardial infarction. Also known as binary data.
Differential verification bias	A type of bias that occurs when a diagnosis is verified using different reference standards, depending upon the result of the index test.
Discounting	In health economics, a reduction applied to future costs and benefits to reflect the fact that costs and benefits available today have a higher value than those occurring in the future.
Double-blind	In a controlled trial, this is the process by which the participants and the investigators (outcome assessors) are prevented from knowing which intervention the participants have been given. See also Blinding.
Effectiveness	The extent to which a specific intervention, applied under usual circumstances, does what it is intended to do.
Efficacy	The extent to which an intervention produces a beneficial effect under ideal conditions.
Estimate of effect	The observed relationship between an intervention and an outcome expressed as, for example odds ratio, risk difference, risk ratio, hazard ratio, standardised mean difference, weighted mean difference, number needed to treat.
Evidence-based practice	An approach to the practice of medicine that involves integrating individual clinical expertise with the best available external clinical evidence from systematic research.
Experimental intervention	An intervention under evaluation.
Experimental study	A study in which investigators determine (by randomisation or another method) to which intervention group an individual will be allocated.
External validity	The degree to which the results of a study hold true in other settings (generalisability). See also Validity.
Fixed-effect model	A method of calculating a pooled effect that assumes all variation of estimates of effect between studies is assumed to be due to random error (the play of chance). See also Random-effects model.

Forest plot	A graphical representation of the individual results of each study included in a meta-analysis together with or without the pooled meta-analysis result. The plot provides an 'at a glance' indication of the variability between studies and highlights any studies with outlying results.<br>Each study is shown as squares centred on its estimate of effect with a horizontal line indicating the confidence interval. A vertical line is drawn through the value that indicates no difference between the interventions being compared (e.g. 1.0 for an odds ratio). If shown, the overall pooled estimate is represented as a diamond at the bottom of the plot. The centre of the diamond represents the pooled point estimate, and its horizontal extremities represent the confidence interval.
Free text terms	In literature searching, the use of everyday words and phrases, as opposed to index terms, to search bibliographic databases.
Funnel plot	A graphical display of study precision such as the standard error plotted against effect size that can be used to investigate biases associated with small trials (including publication bias).
Generalisability	See External validity.
Grey literature	A general term for the kind of material that is not published in an easily accessible form or listed in standard bibliographic databases, for example conference proceedings, internal reports, theses and some books.
Grounded theory	A set of methods for sampling, data collection and analysis.
Handsearching	The process of searching a journal page by page to identify relevant articles.
Hazard ratio	A measure of effect calculated by time-to-event analyses that represents how many times more or less one group is likely to experience the outcome of interest.
Health technology	A broad term that covers any method used by health professionals to promote health, to prevent and treat disease, or to foster and improve rehabilitation and long-term care.
Heterogeneity	In systematic reviews heterogeneity refers to variability or differences between studies. A distinction is sometimes made between:<br>statistical heterogeneity – differences in the effect estimates<br>methodological heterogeneity – differences in study design<br>clinical heterogeneity – differences in participants, interventions or outcome measures<br>See also Homogeneity.
Hierarchy of evidence	A hierarchy of study designs based on their internal validity, or risk of bias, with well-designed systematic reviews and randomised trials at the top and observational studies and case series lower down.
Homogeneous	The degree of similarity between the studies included in a review.

Idea webbing	In narrative synthesis, the use of visual methods to help to construct groupings of, and relationships between, ideas and/or concepts. Closely related to Conceptual mapping.
Incorporation bias	A type of bias that occurs when the result of the index test is used in establishing the final diagnosis (i.e. it forms part of the reference standard).
Incremental cost-effectiveness ratio (ICER)	The difference in the mean costs of two interventions in the population of interest divided by the difference in the mean outcomes in the population of interest.
Index test	The test whose performance is being evaluated.
Indexing term(s)	A word or words used to describe the subject of, for example, a journal article. They are designed to make searching easier and more effective. Ideally these terms will be assigned from a controlled vocabulary, for example MeSH. See also MeSH.
Individual patient data (IPD) meta-analysis	A specific type of systematic review that uses the original individual participant data obtained from those responsible for included studies. Data are centrally collected, checked and re-analysed.
Intention to treat (ITT) analysis	True intention to treat analysis captures two criteria: (i) participants should be analysed irrespective of whether or not they received their allocated intervention and irrespective of what occurred subsequently, for example, participants with protocol violations or those subsequently judged ineligible should be included in the analysis; (ii) all participants should be included irrespective of whether outcomes were collected. Although the first criterion is generally accepted, there is no clear consensus on the second as it involves including participants in the analyses whose outcomes are unknown, and therefore requires imputation of data. Many authors describe their analyses as ITT when only the first criterion has been met.
Internal validity	See Validity.
Inter-rater agreement	The degree of agreement exhibited when a measurement is repeated under identical conditions by different raters.
Interrupted time series	A quasi-experimental study design involving multiple observations over time that are 'interrupted', usually by an intervention, to permit separation of real intervention effects from other long-term trends.
Intervention group	A group of participants in a study receiving a particular intervention.
Inverse variance method	A widely used and easy to implement method of pooling study results which is very flexible and can be used to combine any type of effect measure provided that standard errors are available. A fixed effect meta-analysis using the generic inverse variance method calculates a weighted average of study effect estimates by summating individual effect estimates and weighting these by the reciprocal of their squared standard errors.

Investigator triangulation	In narrative synthesis, a method of exploring the extent to which heterogeneity in study results may be attributable to the diverse approaches taken by different researchers.
Kappa statistic	A measure of inter-rater agreement.
Knowledge transfer	Interactions between decision-makers and researchers through the process of planning, disseminating and applying existing or new research in decision-making.
L'Abbé plot	A scatter plot of the risk in the experimental group against the risk in the control group, used to explore possible heterogeneity in systematic reviews.
Language bias	Bias in a systematic review resulting from limiting inclusion to the exclusion of items not written in a particular language or languages.
Language restrictions	The deliberate restriction of search results to particular languages. Results in language bias.
Likelihood ratio (LR)	A measure of accuracy of a diagnostic test. The likelihood ratio expresses the odds that a diagnostic test will give the correct result in a patient with the target disorder.
Linkage and exchange	Two way communications and partnerships between producers and users of research.
Literature search	For a systematic review this should be a systematic search for information on a given topic. Can include searches of bibliographic databases, websites, handsearching of journals and books, citation searching and reference checking.
Mantel-Haenszel	A method for combining studies that uses an alternative weighting scheme to the inverse variance method. It has better statistical properties than the inverse variance method when events are few.
Markov model	An analytical method particularly suited to modelling repeated events (e.g. headache) or the progression of a chronic disease (e.g. dementia) over time.
Masking	See Blinding.
Mean difference	A standard statistic that measures the absolute difference between the mean value of two groups, estimating the amount by which on average the intervention changes the outcome compared to the control.
Medical Subject Heading (MeSH)	MeSH is the controlled vocabulary indexing system used by the National Library of Medicine for indexing articles on Medline. It is also used in some other electronic bibliographic databases.<br>See also Indexing term; Keyword.
Meta-analysis	Statistical techniques used to combine the results of two or more studies and obtain a combined estimate of effect.
Meta-ethnography	A set of techniques for synthesising qualitative studies. It involves the selection, comparison and analysis of studies to create new interpretations or concepts.

Meta-regression	A statistical technique used to explore the relationship between study characteristics and study results in a systematic review.
Meta-synthesis	See Multi-level synthesis.
Mixed-method	The use of qualitative and quantitative methods together.
Moderator variable	A variable that alters the effect of an explanatory variable on a dependent variable.
Multivariable prediction modelling	A method of quantifying the contribution of each of a series of tests to the diagnostic process by modelling the occurrence of the target condition as a function of the different test results.
Multi-level synthesis	Where a review of qualitative studies is undertaken alongside a review of quantitative studies and the results of the two syntheses are combined. (Also referred to as meta-synthesis, sequenced, or cross-design synthesis).
Narrative synthesis	Predominantly a textual approach that provides an analysis of the relationships within and between studies and an overall assessment of the robustness of the evidence.
Negative predictive value	The probability of non-disease among persons with a negative test result.
Number needed to treat/harm (NNT/NNH)	An estimate of how many people need to receive an intevention before one more person would experience a beneficial or a harmful outcome, respectively. Also referred to as number needed to treat for benefit (NNT_B) and number needed to treat for harm (NNT_H).
Observational study	A study in which the investigators observe and measure but do not seek to intervene.
Odds	Odds describe the ratio of the probability that an event will happen to the probability that it will not happen.
Odds ratio	The ratio of the odds of an event in one group (e.g. the experimental (intervention) group) to the odds of an event in another (e.g. the control group).
One-way or multi-way sensitivity analyses	In economic evaluations, one-way simple sensitivity analysis varies each parameter individually in order to isolate the consequences of each parameter on the results of the study. Multi-way simple sensitivity analysis varies two or more parameters at the same time and the overall effect on the results is evaluated.
Opportunity costs	The cost of foregone outcomes that could have been achieved through alternative investments.
Outcome	An aspect of a participant's clinical or functional status that we seek to change through intervention, for example survival, tumour recurrence, conception, live birth, level of anxiety, frequency of asthma attacks. See also Primary outcome and Secondary outcome.
Overall accuracy	The proportion of people correctly classified by the test.

Parallel synthesis	Where a review of qualitative studies is undertaken alongside a review of quantitative studies and the formal qualitative synthesis is used to interpret the findings of the quantitative synthesis.
Partial verification bias	A type of bias that occurs when only a selected sample of participants undergoing the index test also receive the reference standard (e.g. only those who had a positive index test result).
Per protocol analysis	An analysis restricted to those participants in a study who followed the trial protocol closely enough to ensure that their data would be likely to show an effect of treatment if it existed. Per protocol analysis may be subject to bias because the reasons for not following the protocol may be related to treatment.
Performance bias	Bias resulting from systematic differences in care provided to those in each intervention group (other than the intervention being evaluated) that arise because carers or participants act differently because they know which intervention is being delivered.
Peto odds ratio	Calculates odds based on the difference between the observed number of events and the number of events that would be expected if there was no difference between experimental and control interventions. The method performs better than alternative approaches when events are very rare. It can also be used to combine time to event data by pooling log rank observed–expected (O – E) events and associated variance. It can give biased estimates when treatment effects are very large, or where there is a lack of balance in treatment allocation within the individual studies.
Piloting	The process of testing a procedure on a small scale before introducing it into practice, e.g. testing a data extraction form on a small sample of studies to identify any problems and inconsistencies between reviewers.
Placebo	An intervention without specific biological activity in the condition being treated, usually administered to compare its effects with those of an active intervention. Placebos are used because the act of intervention (rather than the intervention itself) may bring about some benefit for psychological or other reasons.
Pooling	See Meta-analysis.
Positive predictive value	The probability of disease among persons with a positive test result.
Precision	A measure of the likelihood of random errors in the results of a study, meta-analysis or measurement.<br>The proportion of articles identified by a search strategy that are relevant.
Primary outcome	The main or outcome of greatest importance. See also Outcome and Secondary outcome.

Primary study	The original study in which data were collected. The term is sometimes used to distinguish such studies from secondary studies that re-examine previously collected data (e.g. systematic reviews).
Probabilistic sensitivity analyses	In economic evaluations, probabilistic sensitivity analysis attributes distributions of probabilities to the uncertain variables which are incorporated into evaluation models based on decision analytical techniques (e.g. Monte-Carlo simulation). This method can only be used to deal with uncertainty in data input.
Prognostic markers (biomarkers)	Characteristics that help to identify or categorise people with different risks of specific future outcomes. They may be simple clinical measures such as body mass index, but are more often pathological, biochemical, molecular or genetic measures or attributes.
Prognostic tests	Tests conducted to assess a patient's risk of a particular outcome.
Prospective study	A study in which participants are identified and then followed forward in time to observe whether particular outcomes do or do not occur.
Publication bias	Bias arising from the fact that studies with statistically significant results are more likely to be published than those with inconclusive results. As a result, systematic reviews that fail to include unpublished studies may omit relevant research and are likely to be biased towards the positive and overestimate the effect of an intervention.
Pull strategy	Facilitating demand (user)-led access to research findings.
Push strategy	Researcher-led distribution of new research findings.
p-value	The probability of obtaining the observed effect (or larger) under the null hypothesis which for systematic reviews will commonly be the assumption that there is no effect of the experimental intervention. A very small p-value means that it is very unlikely that the observed effect has arisen purely by chance and provides evidence against the null hypothesis.
Q*	The point on the ROC curve that intersects with the line of symmetry (where sensitivity is equal to specificity). Sometimes used as an indicator of overall test performance where there is no clinical preference for maximising either sensitivity or specificity.
Qualitative comparative analysis	A method for summarising and comparing data from case studies using Boolean logic.
Qualitative meta-summary	A set of techniques for aggregating qualitative research findings.
Qualitative meta-synthesis	A set of techniques for the interpretive integration of qualitative findings.
Qualitative research	Research that adopts an interpretive, naturalistic approach and studies things in their natural settings.

Quality of life	An individual's emotional, social, and physical well-being, and their ability to function in the ordinary tasks of living.
Quality threshold	In systematic reviews, restricting inclusion to studies that meet predefined criteria related to quality (validity).
Quality-adjusted life year (QALY)	In economic evaluations, a measure of health gain in which survival duration is weighted or adjusted by the patient's (health-related) quality of life during the survival period.
Quantitative research	Research that concentrates on describing and analysing phenomena by using numerical data and empirical models.
Quantitative synthesis	See Meta-analysis
Randomisation	The process of allocating participants to one of the groups of a randomised controlled trial using (i) a means of generating a random sequence and (ii) a means of concealing the sequence, such that those entering participants to a trial are unaware of which intervention a participant will receive. This should ensure that intervention groups are balanced for both known and unknown factors.
Randomised controlled trial (RCT)	An experiment in which investigators use randomisation to allocate participants into the groups that are being compared. Usually allocation is made at the level of individuals, but sometimes it is done at group level e.g. by schools or clinics.
Receiver Operating Characteristic (ROC) curve	A graph used to display the trade-offs between sensitivity and specificity as a result of varying the diagnostic threshold.
Reference standard	The best currently available diagnostic test, against which the index test is compared.
Regression analysis	A statistical modelling technique used to estimate or predict the influence of one or more independent variables on a dependent variable e.g. the influence of stage of disease and tumour size on survival.
Regression to the mean	A statistical phenomenon by which extreme examples from any set of data are likely to be followed by examples which are less extreme; a tendency towards the average of any sample. For example, the offspring of two very tall individuals tend to be tall, but closer to the average (mean) than either of their parents.
Relationship marketing	Developing long-term relationships with customers in order to retain them; relationship marketing techniques focus on customer retention and satisfaction.
Relative risk	See Risk ratio.
Reporting bias	A bias caused by only a subset of all relevant data being available for inclusion. For example through not all trials being published or not all outcomes being reported.
Research synthesis	The combination and evaluation of separate studies to provide a coherent overall understanding to a research question.

Resources	A general term covering the staff, time, money, equipment and consumables required to, for example, implement an intervention or conduct a systematic review.
Retrospective study	A study in which the outcomes have occurred to the participants before the study commenced.
Risk	The probability with which an outcome (usually adverse) will occur. For example, if out of 100 participants 20 have a myocardial infarction, the risk of infarction is 0.2.
Risk difference	The difference in size of risk between two groups. For example, if the control group has a 30% risk of experiencing a particular event and the intervention group has a 20% risk of experiencing the event, the risk different is 10%. Also known as Absolute risk reduction.
Risk factor	An aspect of an individual's genetic, physiological, environmental, or socioeconomic state that affects the probability of them experiencing a particular disease or outcome. For example people with high body mass index are at increased risk of developing diabetes.
Risk ratio	The ratio of the risk of an event in one group (e.g. the experimental (intervention) group) to the risk of an event in another (e.g. the control group).
Sample size calculation	A calculation performed when planning a clinical study to determine the number of participants needed to ensure a given probability of detecting an effect of a given magnitude if it exists.
Screening test	A test used to detect possible disease in people without symptoms.
Search interface	The means by which a user can interrogate a database. Interfaces vary in complexity. Some consist merely of a text box in which a limited number of words can be entered. Others are more complex and allow the searcher to create complex searches. See also Database host.
Search operator	A term used to combine words within a search. For example, many search interfaces allow searches for terms occurring within so many words of each other (known as adjacency searching). See also Boolean operator.
Search strategy	The exact terms and their combinations used to search a bibliographic database.
Secondary outcome	An outcome of lesser importance than the primary outcome. See also Outcome and Primary outcome.
Secular trend	A relatively long-term trend in a community or country.

Selection bias	1. Bias caused by systematic differences between comparison groups in prognosis or responsiveness to treatment.<br>2. Bias caused by systematic differences between those who are selected for a study and those who are not. This affects the generalisability (external validity) of a study but not its (internal) validity or risk of bias.<br>3. Bias arising from the way in which studies were selected for inclusion in a systematic review, for example, publication bias.
Sensitivity	In diagnostic/screening tests, a measure of a test's ability to correctly identify people with the disease or condition of interest.<br>In literature searching, the proportion of relevant articles that are retrieved using a specific search strategy.
Sensitivity analysis	An analysis used to test the robustness of findings and determine how sensitive results are to the data that were included and/or the way that analyses were done.
Sham (surgery/device)	An activity that makes the recipient believe they have received the actual intervention when they have not; e.g. sham surgery involves an anaesthetic, an incision and suturing, but without the actual surgical intervention being performed.
Specificity	In diagnostic/screening tests, a measure of a test's ability to correctly identify people who do not have the disease or condition of interest.<br>In literature searching, the proportion of non relevant articles that are not retrieved.
Stakeholder	In systematic reviews a person or group with an interest in or potentially affected by the results of the review.
Standardised mean difference	The difference between two estimated means divided by an estimate of the within-group standard deviation. It is used to standardise and combine results from studies that have used different ways of measuring the same concept, e.g. mental health.
Statistical heterogeneity	See Heterogeneity.
Statistical power	The probability of rejecting the null hypothesis when a specific alternative hypothesis is true. In comparative studies the chance of detecting a real effect as statistically significant, given that the effect actually exists. For a given size of effect, studies with more participants have greater power. Studies with a given number of participants have more power to detect large effects than to detect small effect.
Stochastic	An adjective describing a random or probabilistic event or process.
Sub-group analysis	In a clinical study or systematic review, an analysis in which the effect of the intervention is evaluated in a defined subset or subsets of participants.
Summary data	Data that have been aggregated for presentation or analysis, for example numbers of events in each group in a clinical trial.

Surrogate outcome	An outcome measure that is not of direct practical importance but is believed to be an indicator or predictor of outcomes that are clinically important. These are often physiological or biochemical markers that can be obtained much more quickly compared to the clinical outcome of interest. To be valid, a surrogate outcome must have been shown to correlate with and accurately predict the outcome of interest.
Test accuracy study	A one-sided comparison between the results of an index test and those of a reference standard. Any discrepancy is assumed to arise from error in the index test.
Thematic analysis/synthesis	A method used in the analysis of qualitative data to systematically identify the main, recurrent and/or most important themes and/or concepts across multiple responses.
Threshold analyses	In economic evaluations, threshold analysis identifies the critical values of the parameters above or below which the results of a study vary. This method is usually used together with simple sensitivity analysis.
Time horizon	The time span that reflects the period over which the main differences between interventions in health effects and use of health care resources are expected to be experienced.
Time preferences	The predilection of an individual (or a society) for the use of resources in the present rather than in the future.
Time-to-event data	Data that reflect not just whether an event occurs but the time at which it happens. For example time to death or survival analysis. Each data item is represented by a state variable indicating whether or not an event has occurred and an elapsed time at which the state was assessed. Individuals who have not (yet) experienced the event at a particular point in time are censored and contribute their event-free time to the analysis.
Treatment received analysis	See Per-protocol analysis.
Triangulation	A research strategy in which the researcher observes the same variable or phenomenon with multiple sources, measures, and methods.
Truncation symbol	A symbol used when searching electronic databases to retrieve all words that begin with a particular stem. For example, a search for 'child$' on Ovid MEDLINE will find any words that begin with 'child', including 'child', 'children' and 'childhood'.
Undiscounted	See Discounting.
Update searching	The re-running of a literature search to capture material that has become available since the original search was conducted. May involve re-writing search strategies to take account of changes in terminology and database indexing.
Validity (of a measurement)	The degree to which a measurement truly measures what it purports to measure.

Validity (of a study)	The degree to which a result of a study is likely to be true and free of bias (systematic errors), and hence the degree to which inferences drawn from the study are likely to be justified. Validity in this sense is synonymous with internal validity. See also External validity.
Weighting	In meta-analysis, the relative contribution of each individual study to the overall result and/or the method used to determine this. Studies are often weighted by the inverse of the variance of their measure of effect so that studies with more statistical information make a relatively greater contribution.
Worst/best case analysis	In economic evaluations, a sensitivity analysis using extreme values for the input data to investigate the outcome of the economic evaluation in the extreme case. A pessimistic or optimistic outcome is generated. Also known as Analysis of extremes.
2x2 contingency table	A table presenting the results of a test accuracy study, showing the number of true positive, false positive, false negative and true negative results.

Index

A
absolute effects 64-65, 75
abstracts
    in identifying evidence 16-18, 223
    in inclusion criteria 12-13
    in report writing 80
    in study selection 13, 23, 25
accountability 79
adverse effects 9, 177-197, 253-254
advisory group 5-6, 9, 19, 24, 159, 161-162
allocation concealment 35, 42
analysis (see also meta-analysis) 36-41, 45-76
    clinical tests 124, 127, 136, 146
    public health 169
    adverse effects 183, 185, 190-191
    economic evaluations 202, 211-213
    qualitative evidence 225-226, 229-230
analysis of uncertainty 208-211
applicability, see generalisability
archiving 80, 84
attrition bias 36
authorship 24-25, 79
available case analysis 71-72

B
Bayesian methods 74-75, 191
before-and-after studies 11, 39
best evidence synthesis 164
bias 3-4, 6, 15, 33-44
    assessment 3-4, 43, 125-128, 136, 138, 186-187
    attrition 36
    detection 35, 39
    disease progression 127
    language 12, 16-17
    methodological 33-44, 66, 70, 124-125, 135-137, 139
    minimizing 16, 23, 28-29, 32, 34-35, 40, 53
    performance 35
    publication 12, 16, 25, 32, 69-71, 120, 136-137, 166
    reporting 41
    risk of 6, 10-11, 23, 32-40, 43-45
    selection / allocation 11, 23, 35, 38-44
    sources of 33, 44, 53, 124-128
bibliographic databases 3, 16-22
    clinical tests 119-120
    public health 164-166
    adverse effects 183-185
    economic evaluations 204
    qualitative evidence 223-224
binary outcomes 57-59
blinding / masking
    in studies 35-37, 39-44, 187-188
    in the review 21, 24, 30

C
case-control study 11
checklist 3, 43, 47, 77, 125, 139, 167-168, 170, 186, 210-212, 214
chi-squared 66, 67
citation searching 16, 19, 119, 166, 251
clinical trials (see also randomised controlled trials) 9-11, 189, 207
cluster randomised trials 11, 38, 72, 164
cohort studies 7, 11, 34, 40, 118-119, 135, 139-140, 182, 186, 190-191
complex interventions 10, 42, 60, 83, 159-161, 167-168, 180, 222
concealment of allocation 34-35, 42
conceptual mapping 49
conclusions, formulation of 75, 78, 80-83, 88, 211-212
conference
    abstracts 12, 13, 16, 18, 30, 84
    presentations 85
    proceedings 16-18, 250
confidence intervals 55, 62, 65-67, 72, 74-76, 133-134, 209, 211
conflict of interests 80
confounding 35, 39-40, 141, 143, 189
content analysis 51, 229
continuous
    data 31, 59, 61, 69, 72, 136-137, 142
    outcomes 59-60
contributorship 78-79
controlled trials 7, 17, 39, 120
cost-benefit analysis (CBA) 202-203
cost-effectiveness analysis (CEA) 202
cost-utility analysis (CUA) 202
counts and rates 61-62

critical appraisal (see also quality assessment) 3, 33, 225-226
cross-over trials 11, 37-38, 73-74
cutpoint 136-137, 140

D

data collection, see data extraction
databases, bibliographic 3, 16-22
clinical tests 119-120
public health 164-166
adverse effects 183-185
economic evaluations 204
qualitative evidence 223-224
data extraction 13, 28-32
clinical tests 121-124, 137
public health 166-167
adverse effects 186
economic evaluations 204-205
data synthesis 14, 45-76
clinical tests 129-133, 142-143
public health 169
adverse effects 190
economic evaluations 212-213
qualitative evidence 228-229
descriptive synthesis 45
design effect (DE) 73
detection bias 35, 39
diagnostic odds ratio (DOR) 116, 120, 130-132
dichotomous
data 31, 57-69
outcomes 57-59
discussion, formulation of 81
dissemination 14, 85-90
via websites 19
framework 87
dose-response 142
double-blinding 36, 42
drop-outs 37, 186

E

economic evaluations 201-214
effect
estimates 9, 12, 32, 42, 54-59, 62, 66-70, 73, 75, 189-190
measures 14, 54, 59, 187
modifiers 68-69
electronic databases 16-19
clinical tests 120
public health 166
adverse effects 184
qualitative evidence 223
equity 68, 162-163
excluded studies 7, 24-26, 36
executive summary 80
experimental studies, see randomised controlled trials, cluster randomised trials
external validity, see generalisability

F

fixed effect model 14, 55, 58, 63, 142
forest plots 52, 62-63, 66-67, 130, 133
funnel plots 52, 69-71, 120

G

generalisability 10, 33-34, 42, 44, 53, 80, 82
clinical tests 139
public health 170
adverse effects 180, 182, 189
economic evaluations 211-213
generic inverse variance method 55-56, 58, 60, 62, 73, 142
graphical presentations 52, 62, 129, 133-134, 169
grey literature 16, 18-19, 89, 166

H

handsearching 17-18, 22-23, 166, 224, 240, 250-252
harvest plot 169
hazard ratio (HR) 60-62, 64, 142
health promotion 72, 160, 163-164, 167, 231
heterogeneity 14, 51-52, 54, 66-71
clinical tests 120, 129-130, 132, 139
public health 164, 168-169
economic evaluations 213-214
qualitative evidence 221
hierarchy of evidence 10, 34, 40, 163, 206
homogeneity 180, 213
hypothesis-generating 20, 40, 69, 182-183
hypothesis-testing 54, 182

I

I^2 statistic 67
idea webbing 49, 52
identifying research evidence (see also search) 13, 16-22
clinical tests 119-120, 137
public health 164-166
adverse effects 183-185
economic evaluations 204
qualitative evidence 223-224
implications 81-83
implementation
of intervention 35-36, 41-42, 53,
public health 162-164, 167-170
qualitative evidence 221-222, 231-232
of dissemination 85-86, 88

imputation 36, 71-72, 74
inclusion / exclusion criteria 6-10, 13, 19, 23-27, 30
clinical tests 113, 119, 135, 138-139
public health 160-164
adverse effects 180
economic evaluations 203-204
qualitative evidence 223-224
index test 109, 113-115, 119-124, 126-129, 133-134
indirect comparison 68, 74
individual patient data (IPD) meta-analysis 61, 68, 137, 142, 239-240
intention to treat (ITT) analysis 36-37, 71-72
internal validity 34, 42, 44, 139-140, 213
interrupted time series 11, 39, 43, 164
interventions
complex 10, 42, 60, 83, 159-161, 167-168, 180, 222
public health 3, 41-42, 157, 159-165, 168-170, 239

K
Kaplan Meier analysis 60
knowledge transfer/translation 85

L
language bias 12, 16-17
literature searching (see also search) 13, 16-22
clinical tests 119-120, 137
public health 164-166
adverse effects 183-185
economic evaluations 204
qualitative evidence 223-224

M
managing references 21
Mantel-Haenszel odds ratio 58
masking / blinding
in studies 35-37, 39-44, 187-188
in the review 21, 24, 30, 66
mean difference 59-60
meta-analysis (see also analysis) 14, 25, 33, 41, 45, 54-63, 71-74, 76-77
clinical tests 130-137, 142, 146-147
adverse effects 182, 190-191
economic evaluations 206, 210, 213-214
meta-ethnography 228, 230
meta-regression 69, 191
mixed treatment comparisons 74
moderator variables 52
multiple regression analysis 143

N
narrative synthesis 14, 44-53, 76, 129, 190, 214, 229
negative likelihood ratio 116, 130-131
negative predictive value 116
number needed to treat (NNT) 64-65

O
observational studies 11, 40-41, 43, 59, 182, 190-191
obtaining documents 21
odds ratio 56-59, 62, 64
ordinal outcomes 61
outcomes 7-11, 13-14, 19, 31, 33-44, 75-76, 162-163, 181-182, 202-205, 221
adverse effects 181-182
binary 57-59
continuous 59-60
dichotomous / binary 57-59
measures 118, 142
ordinal 61
primary 9, 115
public health 162-163
secondary (other) 9, 183
surrogate 9, 162-163
time to event 60-61

P
paired analysis 73-74
participants (see also population) 8-9, 11, 30-31, 33-40, 42, 71-73
peer review 80
performance bias 35
per protocol / treatment received / on treatment analysis 71, 189
Peto odds ratio 58-59
PICOCS 160
PICOS 4, 7-9, 15, 19, 160, 170
piloting 24, 28-29, 32, 125
population (see also participants) 8, 113, 135, 160, 180
positive likelihood ratio 116
positive predictive value 115
primary outcome 9, 115, 163
process evaluation 168
prognostic
studies 135, 138-139
tests 135
project websites 19, 89
prospective meta-analysis 240
protocol (see also review protocol)
amendments 15
writing 6-8, 10, 13-15, 132-133, 161

public health intervention 3, 41-42, 157, 159-165, 168-170, 239
publication bias 12, 16, 25, 32, 69-71, 120, 136-137, 143, 146-147, 166
publication, multiple / duplicate 25

Q

Q-statistic 66, 67
qualitative
analysis 229-231
studies / research 9, 164, 167, 169, 221-232
quality assessment 3, 4, 6, 10, 14, 33-44
clinical tests 124-128, 138-141
public health 167-168
adverse effects 186-189
economic evaluations 206-212
qualitative evidence 225-227
quality of reporting 25, 33, 41, 51-52, 59, 61, 68, 77
clinical tests 120-122, 125, 129, 135-140
public health 161, 167-168
adverse effects 184, 186-189
economic evaluations 207-209, 212-213
qualitative evidence 227
quality of the intervention 33, 41, 167
quantitative synthesis 54-76
quasi-experimental studies 9, 11, 39

R

recommendations 81-83
random-effects model 55-56, 63, 75
randomisation 11, 34-35
randomised controlled trials (RCTs) 11, 34-39, 41, 45, 59, 68, 75
clinical tests 111-112, 142-143
public health 163, 168
adverse effects 182, 186, 188-191
economic evaluations 204, 206
qualitative evidence 223
rapid evidence assessments 241
rate ratios 62
receiver operating characteristic (ROC) curve 117-118, 129-134
reference management 20-21, 25
reference standard 114
registers of trials 18, 71, 223
regression analysis 69, 143
relative and absolute effects 64-65, 75
relative risk 57-58, 64
report writing 77-83
adverse effects 191
data extraction 28
economic evaluations 214
protocol amendments 15
public health 170
qualitative evidence 230-232
quality assessment 44
quality of reporting 41
quantitative results 62-65, 75
search strategies 21-22
study selection 25-26
results, describing 75
reviews of reviews 240
review protocol 6-15, 23-24, 28, 45, 54, 68, 84
clinical tests 121, 132-133, 135-136
public health 160-161
adverse effects 181
economic evaluations 203
risk of bias 6, 10-11, 23, 32-40, 43-45
risk ratios 58-59, 62, 64
robustness of synthesis 48-49, 53-54, 65-66, 76

S

scoping review 240
screening
papers for inclusion 10, 13, 20, 84
interventions 39, 112-113, 118, 120, 132
search
filters 204, 224, 243
literature 20, 23
strategies 16, 19, 22, 78, 137, 179, 204, 224, 251-252
terms 13, 19, 165, 243-247, 251, 253-254
web / internet resources 16, 18, 20, 165-166, 251
secondary (other) outcome 9, 183
sensitivity 115-119, 122-123, 127, 129-134
analysis 10, 12, 24, 65-66, 143, 146, 190, 209, 211, 227
single-gate studies 118-119, 132
social interventions 160
software 21, 29, 133
specificity 115-119, 129-134
standardised mean difference 59-60
statistical analysis 14, 33, 36, 38, 41, 45, 54-75
clinical tests 130-131, 136-137, 142-143
public health 169
adverse effects 189
economic evaluations 208-209

statistical heterogeneity (see also heterogeneity) 66-68, 71, 109, 129
study selection 1, 6, 13, 23-27
    public health 167
    adverse effects 179
subgroup analysis 8, 14, 31, 44, 50-52, 67-69
    clinical tests 132, 137, 139
    public health 169
    adverse effects 190-191
surrogate outcomes 9, 162-163
surveys 164, 229
synthesis 14, 45-76
    clinical tests 129-133, 142-143
    public health 169
    adverse effects 190
    economic evaluations 212-213
    qualitative evidence 228-229

T
test accuracy 111-115, 118-121, 124-129, 131-132, 134
text mining 20
thematic analysis 51, 228
threshold
    analysis 209
    diagnostic 114-115, 117-118, 121-122, 127
    effect 129-131
triangulation 52
two-gate studies 118-119, 132
time-to-event analysis 60-61, 142

U
unpublished papers 12, 16-19, 32, 69, 84, 120, 136-137, 166, 184-185
updating literature searches 1, 20

V
validity
    assessment (see also bias, risk of) 43, 53, 114, 139-140, 206, 212-213, 225, 232
    external, see generalisability
    internal 34, 42, 44, 139-140, 213
variability (see also heterogeneity) 54-55, 60, 67, 73, 130
vote-counting 49, 51

W
web searching, see search
website, dedicated review 19, 24, 77, 89
